# Genome-scale reconstruction and *in silico *analysis of the *Ralstonia eutropha *H16 for polyhydroxyalkanoate synthesis, lithoautotrophic growth, and 2-methyl citric acid production

**DOI:** 10.1186/1752-0509-5-101

**Published:** 2011-06-28

**Authors:** Jong Myoung Park, Tae Yong Kim, Sang Yup Lee

**Affiliations:** 1 Metabolic and Biomolecular Engineering National Research Laboratory, Department of Chemical and Biomolecular Engineering (BK21 program), KAIST, 291 Daehak-ro, Yuseong-gu, Daejeon 305-701, Republic of Korea; 2 BioProcess Engineering Research Center, and Center for Systems and Synthetic Biotechnology, Institute for the BioCentury, KAIST, 291 Daehak-ro, Yuseong-gu, Daejeon 305-701, Republic of Korea; 3 Bioinformatics Research Center, KAIST, 291 Daehak-ro, Yuseong-gu, Daejeon 305-701, Republic of Korea

## Abstract

**Background:**

*Ralstonia eutropha *H16, found in both soil and water, is a Gram-negative lithoautotrophic bacterium that can utillize CO_2 _and H_2 _as its sources of carbon and energy in the absence of organic substrates. *R. eutropha *H16 can reach high cell densities either under lithoautotrophic or heterotrophic conditions, which makes it suitable for a number of biotechnological applications. It is the best known and most promising producer of polyhydroxyalkanoates (PHAs) from various carbon substrates and is an environmentally important bacterium that can degrade aromatic compounds. In order to make *R. eutropha *H16 a more efficient and robust biofactory, system-wide metabolic engineering to improve its metabolic performance is essential. Thus, it is necessary to analyze its metabolic characteristics systematically and optimize the entire metabolic network at systems level.

**Results:**

We present the lithoautotrophic genome-scale metabolic model of *R. eutropha *H16 based on the annotated genome with biochemical and physiological information. The stoichiometic model, RehMBEL1391, is composed of 1391 reactions including 229 transport reactions and 1171 metabolites. Constraints-based flux analyses were performed to refine and validate the genome-scale metabolic model under environmental and genetic perturbations. First, the lithoautotrophic growth characteristics of *R. eutropha *H16 were investigated under varying feeding ratios of gas mixture. Second, the genome-scale metabolic model was used to design the strategies for the production of poly[R-(-)-3hydroxybutyrate] (PHB) under different pH values and carbon/nitrogen source uptake ratios. It was also used to analyze the metabolic characteristics of *R. eutropha *when the phosphofructokinase gene was expressed. Finally, *in silico *gene knockout simulations were performed to identify targets for metabolic engineering essential for the production of 2-methylcitric acid in *R. eutropha *H16.

**Conclusion:**

The genome-scale metabolic model, RehMBEL1391, successfully represented metabolic characteristics of *R. eutropha *H16 at systems level. The reconstructed genome-scale metabolic model can be employed as an useful tool for understanding its metabolic capabilities, predicting its physiological consequences in response to various environmental and genetic changes, and developing strategies for systems metabolic engineering to improve its metabolic performance.

## Background

*Ralstonia eutropha *H16, also known as *Wautersia eutropha *H16 or *Cupriavidus necator *H16, is a nonpathogenic Gram-negative bacterium, found in both soil and water, belonging to the *Betaproteobacteria*. It is a lithoautotrophic bacterium that utilizes CO_2 _and H_2 _as sources of carbon and energy in the absence of organic substrates, and is an environmentally important bacterium that can degrade various aromatic compounds [1]. It is also the best known polyhydroxyalkanoates (PHAs) producer [1-3]. The genome of *R. eutropha *H16 consists of two chromosomes and one plasmid, which have been fully sequenced and annotated: chromosome1 (4,052,032 bp), chromosome 2 (2,912,490 bp) and megaplasmid pHG1 (452,156 bp) [1].

One of the interesting characteristics of *R. eutropha *H16 is its ability to switch between heterotrophic growth (utilizing organic compounds as energy sources) and autotrophic CO_2 _fixation (utilizing H_2 _as an energy source). In the absence of organic substrates, *R. eutropha *operates its autotrophic metabolism by fixing CO_2 _*via *Calvin-Benson-Bassham cycle (CBB) and oxidixing H_2_, which is calatalyzed by two NiFe dehydrogenases [4]. Another significant characteristic of *R. eutropha *is that it can accumulate large amounts of PHA, such as poly[R-(-)-3hydroxybutyrate] (PHB) as intracellular granules of up to 90% of dry cell weight under unbalanced growth conditions [1,2,5]. For this reason, *R. eutropha *H16 has attracted biotechnological interest as an excellent producer of biodegradable plastics and served as a source of the PHA biosynthetic machinary for other recombinant microorganisms [6]. Furthermore, *R. eutropha *H16 can play an important role in microbial degradation of aromatic compounds such as benzoate, phenol and biphenyl in our environment [7].

In order to improve the industrial applicability of *R. eutropha *H16 by metabolic engineering, it is necessary to understand the characteristics of its metabolism and optimize the entire metabolic network at the systems level. Having the complete genome sequence and annotation of *R. eutropha *H16 available, genome-scale modeling and simulation can be employed for such systematic analyses and design of metabolic engineering strategies. Here we report the reconstruction of the *in silico *genome-scale model of *R. eutropha *H16, RehMBEL1391, composed of 1391 reactions including 229 transport reactions and 1171 metabolites (Additional file 1 and 2 and table 1).

**Table 1:** Features of the *in silico *metabolic model of *R.eutropha *H16

Features	Number
Genome feature	
Genome size (base pairs, bp)	7,416,678
Chromosome 1	4,052,032
Chromosome 2	2,912,490
Megaplasmid pHG1	452,156
No. of open reading frames (ORFs)	6,626 (1,678)^a^
Chromosome 1	3,651 (841)
Chromosome 2	2,555 (680)
Megaplasmid pHG1	420 (157)
*In silico *metabolic model	
No. of reactions (redundant) included in the model	1391
No. of biochemical reactions	1162
No. of transport reactions	229
No. of reactions (unique) included in the model^b^	1367
No. of metabolites	1171
No. of ORFs assigned in metabolic network	1256
ORF coverage^c ^(%)	18.96

^a ^The parenthesis contains the number of the ORFs with unknown function.

^b^The same redundant reactions catalyzed by an isozyme is counted as one.

^c^The number of ORFs incorporated in the genome-scale model divided by the total number of ORFs in the genome of *R. eutropha*.

## Methods

### Reconstruction of the metabolic network

A metabolic network of *R. eutropha *H16 was constructed by combining information from various sources, such as public databases, literature, and experiments. The genome annotation information was obtained from NCBI http://www.ncbi.nlm.nih.gov/. The biochemical reactions were collected and analyzed from various public databases, including Kyoto Encyclopedia of Genes and Genomes (KEGG, [8]), BioSilico [9], Metacyc [10] and the University of Minnesota Biocatalyst/Biodegradation Database (UMBBD; http://umbbd.msi.umn.edu/). The transport reactions were analyzed based on TransportDB [11] and literature [1,12] as they are not sufficiently described in typical metabolic pathway databases. The individual reactions were balanced with respect to element and charge for pH 6, 7, and 8. The charges of metabolites at different pH values were estimated based on their p*K_a _*values using the MarvinBeans software developed by ChemAxon http://www.chemaxon.com/download/.

### Fermentation

*R. eutropha *H16 (KCTC 22469; Korean Collection for Type Cultures, Daejeon, Korea) was used in this study. Cells were stored as a glycerol stock at -70°C before use. A seed culture was prepared in a 250 mL Erlenmeyer flask containing 100 mL of MR medium plus 20 g∙L^-1 ^of D-fructose and cultured at 30°C and 250 rpm (JEIO Tech. Co. SI-900R). The MR medium contains per liter: (NH_4_)_2_HPO_4_, 4 g; KH_2_PO_4_, 6.67 g; citric acid, 0.8 g; MgSO_4_∙3H_2_O, 0.8 g, trace metal solution, 5 mL [13]. Batch culture was carried out in a 7.5 L Bioflo 310 fermentor (New Brunswick Scientific Co., Edison, NJ) containing 2 L of MR medium plus 20 g∙L^-1 ^of D-fructose. Seed culture (200 mL) was used to inoculate the fermentor to give the initial OD_600 _of 0.5. For nitrogen-limited cultivation, (NH_4_)_2_HPO_4 _was replaced with 4 g∙L^-1 ^of Na_2_HPO_4 _and 1.8 g∙L^-1 ^of NH_4_Cl. Chemostat cultures were carried out at the dilution rates of 0.05, 0.07, and 0.10 h^-1 ^by continuous feeding of the sterilized MR medium plus 20 g∙L^-1 ^of D-fructose and simultaneous removal of culture broth using peristaltic pumps at the same rates (Cole-Parmer, Vernon Hills, IL). The steady states were determined by monitoring the constant concentrations of biomass, D-fructose, and organic acids in the fermentor for 5 consecutive samples taken at 1-3 h intervals. The pH was controlled at 7.0 by automatic feeding of 2 M KOH. The dissolved oxygen concentration was maintained above 40% of air saturation by supplying air at 1vvm (air volume working volume^-1 ^minute^-1^) and by automatically controlling the agitation speed up to 1,000 rpm. Foaming was controlled by the addition of Antifoam 289 (Sigma, St. Louis, MO). To investigate the effects of pH on PHB synthesis, cells were cultured in 250 mL Erlenmeyer flask containing 100 mL of nitrogen-limited MR medium plus 20 g∙L^-1 ^of D-fructose at pH 6, 7, or 8 (30°C and 250 rpm). The pH was adjusted to 6, 7, or 8 with 2 M KOH solution.

### Analytical procedures

The concentrations of D-fructose and organic acids were determined by high-performance liquid chromatography (Varian ProStar 210, Palo Alto, CA) equipped with UV/VIS (Varian ProStar 320, Palo Alto, CA) and RI (Shodex RI-71, Tokyo, Japan) detectors. A MetaCarb 87 H column (300 × 7.8 mm, Varian) was isocratically eluted with 0.01 N H_2_SO_4 _at 60°C and a flow rate of 0.6 ML∙min^-1^. The OD_600 _was measured using an Ultrospec 3000 spectrophotometer (Pharmacia Biotech., Uppsala, Sweden) to monitor the cell concentration. Cell concentration defined as gram dry cell weight (gDCW) per liter of culture broth was calculated from the pre-determined standard curve relating the OD_600 _to DCW (1 OD_600 _= 0.448 gDCW∙L^-1^) [14].

The content and monomer composition of the PHA were determined by gas chromatography (GC) [15]. Liquid cultures were centrifuged at 4,000 × *g *for 20 min, and then the cells were washed twice with distilled water, and dried overnight at 100°C. The dried cell pellet was subjected to methanolysis in the presence of 15% (v∙v^-1^) sulfuric acid with benzoic acid as an internal standard. The resulting methyl esters of 3-hydroxybutyrate (3HB) were assayed by GC according to the method reported previously [15]. GC analysis was performed by injecting 1 μL of sample into an Agilent 6890N GC system (Agilent Technologies, Palo Alto, CA) equipped with Agilent 7683 automatic injector, flame ionization detector, and a fused silica capillary column (ATTM-Wax; 30 m, ID of 0.53 mm, film thickness of 1.20 mm; Alltech, Deerfield, IL). The GC oven temperature was initially maintained at 80°C for 5 min and ramped to 230°C at 7.5°C min^-1^. It was then increased at a rate of 10°C min^-1 ^until 260°C was reached and held for 5 min. Helium was used as a carrier gas. The injector and detector were maintained at 250 and 300°C, respectively. The PHB content (wt%) was defined as the percentage of PHB concentration (g∙L^-1^) to cell concentration (g∙L^-1^).

### Biomass composition

Some unknown yet important biomass components were experimentally quantified to make the biomass model equation more accurate. The compositions of carbohydrates and amino acids of *R. eutropha *H16 were determined using the cells cultured in MR medium (Korea Basic Science Institute, Daejeon, Korea). The neutral sugars from cell extracts were analyzed using CarboPac PA1 (4.5 × 250 mm) and CarboPac PA1 cartridge (4.5 × 50 mm) with Bio-LC DX-600 (Dionex, Sunnyvale, CA). A mobile phase of 16 mM NaOH was used at the flow rate of 1.0 mL min^-1^. Data were then analyzed with PeakNet on-line software. Amino acid compositions were determined by Waters HPLC systems (Waters, Milford, MA) that consists of two 510 HPLC pumps, gradient controller, 717 automatic sampler, 2487 UV detector, and empower 2 software together with Waters pico-tag column (3.9 × 300 mm). Data for other components were either adopted from the literature or reasonably assumed as described in Additional file 3.

### Energetic parameter calculations

Two energetic parameters, growth associated maintenance energy (GAME) and non-growth associated maintenance energy (NGAME), were calculated as follows. GAME (g gDCW^-1^) is represented as the ATP term in the biomass equation and NGAME (mmol gDCW^-1 ^h^-1^) is represented in an independent ATP consumption reaction in which the flux is fixed to the value calculated for this parameter. After determining the oxygen uptake and D-fructose uptake rates from experiments, GAME and NGAME were set by chemostat data [16,17]. The GAME and NGAME values were calculated based on the finding that the D-fructose uptake rate and cell growth rate are linearly dependent. GAME was calculated from the slope obtained from the plot of D-fructose uptake rate *versus *dilution rate from chemostat data. After determining the GAME value, an arbitrary value of NGAME was then repeatedly changed until the correct y-intercept value (in the plot of D-fructose uptake rate *versus *dilution rate) is obtained. The oxygen and D-fructose uptake rates of 4.6 and 2.6 mmol gDCW^-1 ^h^-1^, respectively, were used as constraints at the growth rate of 0.25 h^-1 ^(Additional file 4). The GAME and NGAME values for *R. eutropha *H16 in MR medium were 15.30 g gDCW^-1 ^and 3.00 mmol gDCW^-1 ^h^-1^; the GAME is generally larger than NGAME [18-20].

### Constraints-based flux analysis

In order to perform constraints-based flux analysis, internal metabolites are first balanced under the assumption of pseudo-steady state [21,22]. This results in a stoichiometric model *S_ij_· v_j _= 0*, in which *S_ij _*is a stoichiometric coefficient of a metabolite *i *in the *j*^th ^reaction and *v_j _*is the flux of the *j*^th ^reaction given in mmol gDCW^-1 ^h^-1^. The resultant balanced reaction model is, however, almost always underdetermined in calculating the flux distribution due to that the number of reactions is greater than that of metabolites. Thus, linear programming (LP)-based optimization, subject to the constraints of mass conservation, reaction thermodynamics and metabolic capacity, was carried out to determine the fluxes as described previously [22-24]. For all the simulations throughout this study, the uptake and secretion constraints for major organic acids, including acetic acid, lactic acid, pyruvic acid, and ethanol, whose transporters are available in the network model of *R. eutropha*, were constrained to zero in order to reflect this organism's growth characteristics, in which no organic acids are produced during batch fermentation (Additional file 4 and 5, [25]). It should be noted that this organism's inability to produce organic acids makes it amenable to high cell density cultivation as it does not require acid tolerance.

The metabolic flux distribution and the changes of metabolic fluxes under several perturbed conditions were examined by flux variability analysis (FVA) [26,27] that calculates minimal and maximal flux values of each reaction for an objective function of maximum cell growth rate. These flux values were compared with the control flux solution space to examine the changes of flux solution space for each reaction under genetic and environmental perturbations [26,28,29]. In addition, in order to identify the gene knockout targets for the enhanced production of 2-methylcitric acid, the method of minimization of metabolic adjustment (MOMA) using quadratic programming (QP) was employed [30].

## Results and Discussion

### Genome-scale reconstruction and general features of *R. eutropha *H16 metabolic network

**Figure 1:**
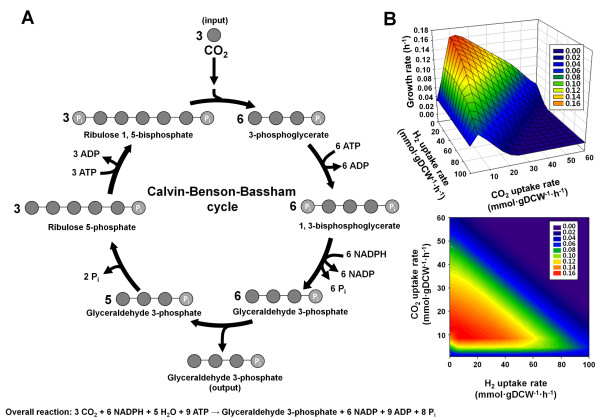
**Sensitivity for gaseous uptake rates of H_2 _and CO_2 _on lithoautotrophic growth of *R. eutropha***. (A) Overview of the CBB cycle for carbon fixation and its overall reaction. (B) Mesh plots graph 3D data as a continuous surface for H_2 _uptake rate, CO_2 _uptake rate, and growth rate when the O_2 _uptake rate is 10 mmol gDCW^-1 ^h^-1^. For simulation of lithoautotrophic growth, the D-fructose uptake rate was constrained to zero and reactions related with CBB cycle were activated.

**Figure 2:**
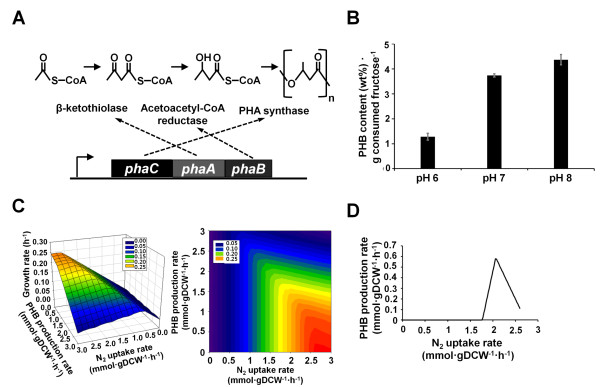
**Effects on poly[R-(-)-3hydroxybutyrate] (PHB) production for different pH values and carbon/nitrogen source uptake ratios**. (A) Genes, enzymes, and reactions for the biosynthesis of polyhydroxyalkanoate (PHA). (B) PHB production yield for different pH values, which are pH 6, 7, and 8. The PHB content (%wt) is defined as the percentage of PHB concentration (g∙L^-1^) to cell concentration (g∙L^-1^). The culture medium is the MR minimal medium with D-fructose. (C) Mesh plots graph 3D data as a continuous surface for nitrogen uptake rate, PHB production rate, and growth rate. (D) Predicted PHB production rate by limiting nitrogen uptake rate for the maximization of growth rate.

The initial metabolic network of *R. eutropha *H16 was constructed by compiling all the enzymatic reactions based on the KEGG database [8]. All these reactions in the metabolic network were elementally and charge balanced at pH 6, 7, and 8. Finally, the extensively curated genome-scale metabolic model of *R. eutropha *H16, RehMBEL1391, consisting of 1391 metabolic reactions and 1171 metabolites was constructed (Additional file 1 and 2 and table 1). The number of transport reactions is 229 and the number of intracellular metabolic reactions is 1162. In this model, 1256 genes out of 6626 open reading frames (ORFs), corresponding to 18.96% of the total ORFs, were incorporated (Table 1). The percentage of reactions assigned to ORFs was 75.4%, while the remaining 24.6% of reactions were also included in the model in the form of lumped multi-step reactions, reactions added to fill missing links, or several transport reactions. The metabolic reactions in RehMBEL1391 were classified into 93 different subsystems. The metabolic network contains several sub-metabolisms, including carbohydrate metabolism, energy meatbolism, lipid metabolism, nucleotide metabolism, amino acid metabolism, cofactor and vitamin meatbolism, and xenobiotics biodegradation and metabolism. The model includes CO_2_-fixing and H_2_-oxidizing reactions for lithoautotrophic growth, including a lumped reaction for the CBB cycle and formate-oxidizing reactions for organoautotrophic growth catalyzed by formate dehydrogenase (E.C. 1.2.1.2, E.C. 1.2.2.1) (Figure 1). The model also contains PHB biosynthesis reactions catalyzed by β-ketothiolase (E.C. 2.3.1.9), acetoacetyl-CoA reductase (E.C. 1.1.1.36), and PHA synthase (E.C. 2.3.1.-) (Figure 2A). Under anaerobic conditions the *in silico *cell can grow by utilizing nitrate reductase (E.C. 1.7.99.4) and nitric oxide reductase (E.C. 1.7.99.7), which are part of the denitrification pathway using NO_3_^- ^and NO_2_^- ^as alternative electron acceptors, respectively. *R. eutropha *cannot grow anaerobically without the alternative electron acceptors [1], which agrees with the *in silico *metabolic simulation. Consequently, *R. eutropha *relies on a strictly respiratory energy metabolism and cannot grow in the absence of final electron acceptors such as O_2_, NO_3_^-^, and NO_2_^- ^.

**Figure 3:**
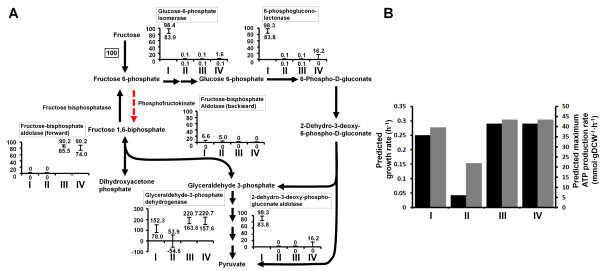
**Effects of expression of phosphofructokinate reaction in *R. eutropha***. It was investigated for wild-type strain (I), *eda*- deficient strain (II), *eda*- deficient and *pfk*-expressing strain (III), and both *edd*- and *pfk*-expressing strain (IV) of *R. eutropha*. (A) Pathway and flux distribution of the central metabolism for each strain. The flux distribution was investigated by flux variability analysis (FVA). The y axis in each graph indicates the relative flux (%) that is normalized to the carbon source uptake rate, which is D-fructose uptake rate. The upper and lower values in each graph indicate the maximum and minimum values, respectively, in flux variability. (B) Predicted growth rate and maximum ATP production rate for each strain. The black bar denotes the predicted growth rate and the grey bar denotes the predicted maximum ATP production rate.

*R. eutropha *H16 is missing a phosphofructokinase enzyme (E.C. 2.7.1.11) encoded by *pfk *converting fructose 6-phosphate to fructose 1,6-bisphosphate in the Embden-Meyerhof-Parnas (EMP) pathway. For the metabolism of hexoses, Entner-Doudoroff (ED) pathway is utilized rather than the EMP pathway [31,32]. Thus, the most fluxes in *R. eutropha *pass through 6-phosphogluconate and enter the glycolytic pathway at glyceraldehyde-3-phosphate and pyruvate nodes. The simulation results suggested that knocking out the essential genes in the ED pathway retards cell growth (Figure 3) when using D-fructose, which agrees with experimental results [31].

### Refinement of the genome-scale metabolic network of *R. eutropha *H16

#### Determination of biomass composition

The reconstructed metabolic network of *R. eutropha *H16 was further refined by incorporating experimentally determined biomass formation reactions to represent cellular growth. The amino acid and carbohydrate compositions of *R. eutropha *H16 were experimentally measured using the cells at the exponential growth phase during aerobic batch cultivation on a minimal medium containing D-fructose. The compositions of other components were adopted from either literature or available information. The fatty acid compositions in phospholipids of the cells grown in normal and nitrogen-limited minimal medium containing D-fructose were found to differ significantly [33]. The major (over 95%) fatty acids in the cells cultured in normal medium were myristic acid (C14:0), palmitic acid (C16:0), palmitoleic acid (C16:1), and oleic acid (C18:1). Under the condition of active PHB biosynthesis in nitrogen-limited medium, the content of palmitoleic acid (C16:1) decreased, while the content of heptadecanoic acid (C17:0) increased from 0.6% to 11.2%. Considerable amounts of myristoleic acid (C14:1) and nonadecanoic acid (C19:0) were also detected when grown in nitrogen-limited medium (Additional file 3). These lipid compositions were incorporated into the biomass formation equations to describe the physiology of *R. eutropha *for each condition (Additional file 1 and 3).

#### Carbon source utilization

The genome-scale metabolic model of *R. eutropha *H16, RehMBEL1391, was further improved by incorporating the information on its growth capabilities using 131 different carbon sources reported in literature (see Additional file 6 for the relevant references). Based on this information, transport reactions and missing reactions were also appropriately updated to the model. For example, although butyrate-CoA ligase (E.C. 6.2.1.2) essential for the utilization of butyrate was not initially annotated, *R. eutropha *appears to utilize butyrate as a carbon source [32,34]. Therefore, the reaction catalyzed by butyrate-CoA ligase was accordingly incorporated into the model. Likewise, the capability of *R. eutropha *to degrade and utilize many aromatic compounds as carbon sources that are not beneficial to the ecosystem was properly described in RehMBEL1391, including benzoate, phenol, and cresol. During the course of model refinement, it was found that these aromatic compounds are degraded and converged to the key common intermediates, namely protocatechuate and catechol, which are further metabolized to acetyl-CoA and succinyl-CoA, and fed into central carbon metabolism.

### Effects of varying feeding ratios of gas mixture for the lithoautotrophic growth of *R. eutropha*

One important capability of *R. eutropha *is the lithoautotrophic growth utilizing CO_2 _as a carbon source and H_2 _as an energy source [4,35]. In the absence of organic compounds, *R. eutropha *operates autotrophic CO_2 _fixation *via *CBB cycle and NiFe hydrogenases to oxidize H_2_. The lumped CBB reaction was added into the model to describe the lithoautotrophic growth of *R. eutropha*. Microbial growth rate under lithoautotrophic growth condition is controlled by regulating the supply rate of the gaseous substrates, namely H_2_, O_2_, and CO_2_. More precisely, the feeding ratio of gas mixture (H_2_, O_2_, and CO_2_) has been considered to exert significant effects on growth characteristics of *R. eutropha *under this lithoautotrophic condition [35]. Because of the importance of the feeding ratio of gas mixture, RehMBEL1391 was again employed to study the relationship between growth rate and gas composition of three gaseous substrates (Figure 1). The predicted results show that the growth rate of *R. eutropha *is more sensitive to CO_2_/O_2 _ratio than H_2_/O_2 _ratio. These results agree well with the reported experimental data that examined several ratios of CO_2_/O_2 _and H_2_/O_2 _in the feeding gas mixture on the lithoautotrophic growth of *R. eutropha *[35].

### Strategies for PHB production

*R. eutropha *is capable of accumulating short-chain length PHA (SCL-PHA) under nutrient-limited condition in the presence of excess carbon sources. PHB is synthesized from acetyl-CoA by three sequential enzymes encoded by the *phaA, phaB*, and *phaC *genes, and their respective reactions for PHB biosynthesis are reflected in RehMBEL1391. First, two acetyl-CoAs are condensed to form acetoacetyl-CoA catalyzed by β-ketothiolase (*phaA*). Next, acetoacetyl-CoA is reduced to (R)-3-hydroxybutyryl-CoA by acetoacetyl-CoA reductase (*phaB*). PHA synthase (*phaC*) finally links (R)-3-hydroxybutyryl-CoA to the growing chain of PHB (Figure 2A, [1,36]). RehMBEL1391 with this set of PHB biosynthetic reactions was then employed for further in depth study on PHB biosynthesis under varying environmental conditions.

For PHB production in *R. eutropha*, pH is known to play an important role [37]. In order to gain better insight into the effects of pH on the capability of *R. eutropha *to produce PHB, RehMBEL1391 whose metabolic reactions are elementally and charge balanced for pH 6, 7, and 8 was systematically simulated (see Additional file 7 for the relevant mol files to estimate the charges of metabolites at different pH values using MarvinBeans software). For each pH value of 6, 7, and 8, PHB production rate under nitrogen-limited condition was predicted for identical D-fructose consumption rate through constraints-based flux analysis of RehMBEL1391; nitrogen uptake rate was constrained to be less than 1 mmol gDCW^-1 ^h^-1 ^to describe nitrogen-limited condition. As a result, the predicted yield (Y_PHB_) obtained at pH 8 was superior to the yields predicted at pH 6 and 7. The model predicted that the Y_PHB _increased as pH increases, which well agrees with our experimental data shown in Figure 2B. This observation was also supported by previous report, in which the yield of poly[(3-hydroxybutyrate)-co-(3-mercaptopropionate)] from *R. eutropha *was greatest at pH 8 in the range between pH 6 and 8 [37]. Taken together, use of RehMBEL1391 further provides *in silico *confirmation that pH value of fermentation medium significantly influences the polymer yield and its yield is optimal under mild basic condition. Thus, the RehMBEL1391 model can be used to systematically investigate the effects of other environmental changes on the physiology of *R. eutropha *as well.

Likewise, the relationships among the growth rate of *R. eutropha*, PHB production rate and nitrogen uptake rate, an important factor for PHB biosynthesis, were investigated by using RehMBEL1391 in order to find the optimal level of nitrogen limitation that maximizes PHB biosynthesis (Figure 2C and 2D). For this, the uptake rate of D-fructose, a carbon source in this case, was constrained to be no greater than 2.6 mmol gDCW^-1 ^h^-1^, while the nitrogen uptake rate was intentionally varied from 0 to 3 mmol gDCW^-1 ^h^-1^. Cell growth rate was maximized as an objective function using constraints-based flux analysis. As a result, a positive correlation was observed between the PHB production rate and the C/N uptake ratio (mmol D-fructose mmol nitrogen^-1^) up to a certain level. The predicted PHB production rate increased with increasing C/N uptake ratio of up to 1.26 mmol D-fructose mmol nitrogen^-1^. However, the predicted PHB production rate started to decrease once the C/N uptake ratio exceeds 1.26 mmol D-fructose mmol nitrogen^-1^. This observation was supported by previous reports, in which the optimal C/N ratio existed on the production of PHB and its copolymers in *R. eutropha *[38-40].

Nitrogen sources are important precursors of amino acids, which in turn are used as precursors of other metabolites or building blocks for biomass formation. Thus, nitrogen limitation causes primarily the retardation of cell growth due to the insufficiency of amino acids. Additionally, acetyl-CoA is not only a precursor for PHB production, but also an important precursor for biomass formation. Thus, cell growth and PHB production compete each other for the utilization of acetyl-CoA. Based on the genome-scale simulation using RehMBEL1391, the limitation of nitrogen uptake rate had negative effects on the formation of precursors of biomass components. However, the excess carbon source (D-fructose) was able to maintain the pool of acetyl-CoA for retarded growth and the excess acetyl-CoA was redirected to PHB production at levels below the optimal C/N uptake ratio. At the C/N uptake ratios higher than the optimal level, the increased limitation of nitrogen negatively affected the formation of biomass components and thus cell growth. Such extreme limitation of nitrogen caused a decrease in the biosynthesis of aspartate, which is also an important precursor for CoA biosynthesis. This causes the decrease in the biosynthesis of acetyl-CoA, and thereby affecting cellular growth and PHB production. Thus, when the C/N uptake ratio increases beyond the optimal level, both cell growth rate and PHB production rate decrease (Figure 2C and 2D). In summary, the PHB production was found to have a positive correlation with the C/N uptake ratio up to an optimal value, and further increase of the C/N uptake ratio resulted in the decrease of PHB production due to the decrease of acetyl-CoA pool (Figure 2C and 2D). Thus, RehMBEL1391 again proved to provide predictions consistent with the considered experimental results regarding the presence of optimal C/N uptake ratio on PHB production, thereby further validating the robustness of this network model.

### Effects of the expression of phosphofructokinase in *R. eutropha*

*R. eutropha *H16 has an incomplete glycolytic pathway that lacks in phosphofructokinase (E.C. 2.7.1.11) encoded by *pfk*, which converts fructose 6-phosphate to fructose 1,6-bisphosphate, but instead utilizes the ED pathway to catabolize D-fructose and other hexoses [31,32]. FVA of RehMBEL1391 also showed that metabolic fluxes from hexoses go through 6-phosphogluconate in the ED pathway and go into the glycolytic pathway at glyceraldehyde-3-phosphate and pyruvate nodes (Figure 3). This unique central carbon metabolic pathway motivated us to examine the catabolism of carbon sources upon specific manipulation of this pathway. As a result of simulation with RehMBEL1391, its growth retardation predicted by *in silico *knockout of 2-dehydro-3-deoxy-phosphogluconate aldolase (E.C. 4.1.2.14) encoded by *eda *supports the importance of ED pathway for the utilization of hexoses, such as D-fructose, in *R. eutropha *(Figure 3). The predicted maximum ATP production rate of *eda*-deficient mutant also decreased, compared with that of the wild-type strain. Furthermore, *in silico *addition of the phosphofructokinase reaction in the *eda*-deficient model recovered the growth rate and ATP production rate (Figure 3). This is explainable by the utilization of a complete glycolytic pathway, which produces ATP two times more than the ED pathway. However, *in silico *co-presence of reactions encoded by *eda *and *pfk *genes did not lead to synergetic effects on the growth rate and ATP production rate (Figure 3). This overall observation was consistent with the previous study [31], which reported the retarded growth of *eda*-deficient *R. eutropha *mutant, the growth recovery of *eda*-deficient but *pfk*-expressing strain, and no synergetic effects of the co-expression of *eda *and *pfk *genes in *R. eutropha *for growth.

### 2-methyl citric acid production in *R. eutropha*

**Figure 4:**
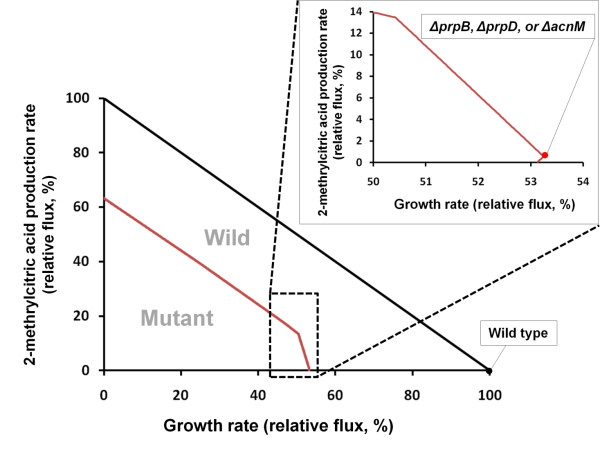
**Flux solution space of wild-type and mutants (*ΔprpB, ΔprpD*, or *ΔacnM*) of *R. eutropha***. Based on the flux solution space of wild-type, 2-methylcitric acid is non-growth associated metabolite when the cell growth rate is maximized. After the *in silico *single knockout of each gene, 2-methylcitric acid is changed to the growth-associated metabolite according to the flux solution space of the mutants. The x and y axes indicate the relative flux (%) of 2-methylcitric acid production rate and cell growth rate that are normalized to the maximum values of 2-methylcitric acid production rate and cell growth rate in the wild-type of *R. eutropha*, respectively.

As a proof-of-concept biotechnological application of *R. eutropha *as a production host, RehMBEL1391 was used to predict production of an important industrial product 2-methylcitric acid, which appears in propionic acid metabolism in many organisms across prokaryotes and eukaryotes, including *R. eutropha *[41-43], yeasts [44], and fungi [45]. 2-methylcitric acid and its derivatives have been considered for potential pharmaceutical applications, such as growth inhibitor agent in cancer treatment and antiperspirants [43]. For the overproduction of 2-methylcitric acid in *R. eutropha, in silico *single gene knockout simulation was conducted using flux balance analysis (FBA) and MOMA in order to redesign the metabolic network and redirect metabolic fluxes towards production of 2-methylcitric acid [30]. As a result, three knockout candidates were predicted with identical top priority in terms of the maximal production rate of 2-methylcitric acid, including methylisocitric acid lyase (E.C. 4.1.3.30) encoded by *prpB*, 2-methylcitric acid dehydratase (E.C. 4.2.1.79) encoded by *prpD*, and 2-methylisocitric acid dehydratase (E.C. 4.2.1.99) encoded by *acnM*, from both FBA and MOMA (Figure 4, [30]). These enzymes are involved in the consumption of 2-methylcitric acid. In addition, when simulating with grouping reaction constraints developed recently for accurate predictions of fluxes by the grouping of functionally related reactions [27], the same three candidates were identified as FBA and MOMA, suggesting the validity of these knockout targets. Inactivation of these target genes altered the solution space, compared with the wild-type strain as shown in Figure 4. The flux values of 2-methylcitric acid production rate and cell growth rate represented in Figure 4 were relative fluxes that were normalized to the maximum values of 2-methylcitric acid production rate and cell growth rate in the wild-type, respectively. In the wild-type strain, production of 2-methylcitric acid was not predicted at optimal growth rate. However, if any one of these predicted target genes was inactivated, the solution space was altered so that RehMBEL1391 is enabled to produce 2-methylcitric acid even at the optimal growth rate, which decreased to about 53% (see the enlarged panel in Figure 4) because metabolic fluxes were redirected to the product without being consumed by these three enzymes. These results are consistent with Ewering's study (2006) on the improvement of *R. eutropha *for the biotechnological production of 2-methylcitric acid [43]. They also disrupted the 2-methylisocitric acid dehydratase encoded by *acnM *in *R. eutropha *and confirmed the enhanced capability of the mutant for producing 2-methylcitric acid.

## Conclusion

*R. eutropha *is a versatile microorganism in many aspects, including PHA production, utilization of diverse carbon sources, degradation of aromatic compounds, lithoautotrophic growth and high cell density growth. To better appreciate this organism and explore potential applications, we reconstructed the genome-scale metabolic model of *R. eutropha*, RehMBEL1391, consisting of 1391 reactions and 1171 metabolites based on the information obtained from genome annotation data, literature and validation experiments. Using this metabolic model, *in silico *metabolic flux analyses were performed to investigate the effects of various environmental and genetic variables. First, the effects of relative feeding ratio of gas mixture, consisting of H_2_, O_2_, and CO_2_, on the lithoautotrophic growth of *R. eutropha *were successfully confirmed. Also, to design strategies for better PHB production, *in silico *metabolic flux analyses of RehMBEL1391 were performed to examine the effects of pH values ranging from 6 to 8 and different C/N uptake ratios on *R. eutropha*'s capability of producing PHB. Furthermore, the effects of expression of phosphofructokinase and the importance of ED pathway for the utilization of hexoses as a carbon source were investigated and compared with the reported experimental data. Finally, RehMBEL1391 was successfully used to identify gene knockout targets for metabolic engineering through *in silico *gene knockout simulation for the production of 2-methylcitric acid. From an industrial perspective, there exist many hurdles to overcome for successful application of *R. eutropha*, particularly in the field of industrial biotechnology. Although this study presented only the limited use of the genome-scale metabolic model, it managed to thoroughly validate the network model in comparison with various experimental data. Hence, it is expected that this network model will serve as a knowledge-base that contributes to tackling problems more systematically and expanding our insight on this organism in return.

## List of abbreviations

PHA: polyhydroxyalkanoate; CBB: Calvin-Benson-Bassham; PHB: poly[R-(-)-3hydroxybutyrate]; ORF: open reading frame; ED: Entner-Doudoroff; 3MP: 3-mercaptopropionate; C/N ratio: carbon/nitrogen source ratio; FBA: flux balance analysis; MOMA: method of minimization of metabolic adjustment; KEGG: Kyoto encyclopedia of genes and genomes; UMBBD: university of Minnesota biocatalyst/biodegradation database; gDCW: gram dry cell weight; GC: gas chromatography; 3HB: 3-hydroxybutyrate; GAME: growth associated maintenance energy; NGAME: non growth associated maintenance energy; LP: linear programming; FVA: flux variability analysis

## Authors' contributions

JMP, TYK, and SYL generated ideas. JMP and TYK, and SYL designed research. JMP performed research. JMP performed analytical experiments. JMP analyzed data. JMP, TYK, and SYL wrote the paper. All authors read and approved the final manuscript.

## Supplementary Material

Additional file 1 List of metabolic reactions in the genome-scale metabolic model of *Ralstonia eutropha *H16 Click here for file

Additional file 2 List of metabolites in the genome-scale metabolic model of *Ralstonia eutropha *H16 Click here for file

Additional file 3 Biomass composition of *Ralstonia eutropha *H16 Click here for file

Additional file 4 Batch and chemostat culture profile of *Ralstonia eutropha *H16 in minimal medium Click here for file

Additional file 5 HPLC and GC analysis results in culture of *Ralstonia eutropha *H16 in minimal medium Click here for file

Additional file 6 Carbon source utilization of *Ralstonia eutropha *H16 Click here for file

Additional file 7 mol files for each metabolite to estimate the charges of metabolites for pH 6, 7, and 8 using MarvinBeans software Click here for file
