# Supplementary material for: Genome-scale reconstruction and in silico analysis of the Ralstonia eutropha H16 for polyhydroxyalkanoate synthesis, lithoautotrophic growth, and 2-methyl citric acid production
Source: BMC Syst Biol. 2011 Jun 28;5:101. doi: 10.1186/1752-0509-5-101 (PMC3154180; doi:10.1186/1752-0509-5-101)
Supplement: Additional file 1 — List of metabolic reactions in the genome-scale metabolic model of Ralstonia eutropha H16 [file 1752-0509-5-101-S1.PDF]

Additional file 1. List of metabolic reactions in the genome-scale metabolic model of *Ralstonia eutropha* H16

Reaction name	Metabolism	Enzyme	E.C. number	Locus(reh)	Gene(reh)	reaction with marvin charges (pH6)	reaction with marvin charges (pH7)	reaction with marvin charges (pH8)
1MNAPTH	1- and 2-Methylnaphthalene degradation	1-methylnaphthalene hydroxylase	1.14.13.-	H16_A1145/H16_B0495/H16_B1480/H16_B2135	unknown/unknown/unknown/unknown	mnapth + o2 + nadh + h -> hmnapth + nad + h2o	mnapth + o2 + nadh + h -> hmnapth + nad + h2o	mnapth + o2 + nadh + h -> hmnapth + nad + h2o
ACLDn	1- and 2-Methylnaphthalene degradation	alcohol dehydrogenase	1.1.1.1	H16_A0757/H16_A3330/H16_B0517/H16_B1433/H16_B1699/H16_B1745/H16_B1834/H16_B2470	adh/unknown/unknown/adhP/unknown/unknown/unknown/unknown	hmnapth + nad -> napthah + nadh + h	hmnapth + nad -> napthah + nadh + h	hmnapth + nad -> napthah + nadh + h
2H3CBZP	1- and 2-Methylnaphthalene degradation	2-hydroxy-3-carboxy-benzalpyruvate hydratase-aldolase	4.2.1.-	H16_A1069/H16_A1070/H16_A1289/H16_A2151/H16_A3307/H16_B0359/H16_B0706	unknown/unknown/unknown/unknown/unknown/unknown/unknown/	2h3cbzpyr + h2o -> fsalac + pyr	2h3cbzpyr + h2o -> fsalac + pyr	2h3cbzpyr + h2o -> fsalac + pyr
2H3MBZP	1- and 2-Methylnaphthalene degradation	2-hydroxy-3-methylbenzalpyruvate hydratase-aldolase	4.2.1.-	H16_A1069/H16_A1070/H16_A1289/H16_A2151/H16_A3307/H16_B0359/H16_B0706	unknown/unknown/unknown/unknown/unknown/unknown/unknown/	2h3mbzpyr + h2o -> msalcah + pyr	2h3mbzpyr + h2o -> msalcah + pyr	2h3mbzpyr + h2o -> msalcah + pyr
SALCH3	1- and 2-Methylnaphthalene degradation	salicylate hydroxylase	1.14.13.1	H16_A0578/H16_A0922/H16_A1785/H16_B0750/H16_B0876	unknown/unknown/unknown/unknown/unknown	msalc + o2 + nadh + 2 h -> dhtolen + nad + co2 + h2o	msalc + o2 + nadh + 2 h -> dhtolen + nad + co2 + h2o	msalc + o2 + nadh + 2 h -> dhtolen + nad + co2 + h2o
2HISOPD	1- and 2-Methylnaphthalene degradation	2-hydroxyisophthalate decarboxylase	4.1.1.-	H16_B2447	unknown	hxsp + h -> salcyl + co2	hxsp + h -> salcyl + co2	hxsp + h -> salcyl + co2
SALCH4	1- and 2-Methylnaphthalene degradation	salicylate hydroxylase	1.14.13.1	H16_A0578/H16_A0922/H16_A1785/H16_B0750/H16_B0876	unknown/unknown/unknown/unknown/unknown	msalc4 + o2 + nadh + 2 h -> 4mctch + nad + h2o + co2	msalc4 + o2 + nadh + 2 h -> 4mctch + nad + h2o + co2	msalc4 + o2 + nadh + 2 h -> 4mctch + nad + h2o + co2
2MNAPTH	1- and 2-Methylnaphthalene degradation	2-methylnaphthalene hydroxylase	1.14.13.-	H16_A1145/H16_B0495/H16_B1480/H16_B2135	unknown/unknown/unknown/unknown	2mnapth + o2 + nadh + h -> 2napthm + nad + h2o	2mnapth + o2 + nadh + h -> 2napthm + nad + h2o	2mnapth + o2 + nadh + 2 h -> 2napthm + nad + h2o
SCCNAPS	1- and 2-Methylnaphthalene degradation	succinyl-CoA:naphthyl-2-methyl-succinyl-CoA transferase	2.8.3.-	H16_B0355/H16_B0367/H16_B0488/H16_B0655/H16_B0656/H16_B0847/H16_B0914	unknown/unknown/unknown/unknown/unknown/unknown/unknown/	napth2ms + succoa -> napmsccoa + succ	napth2ms + succoa -> napmsccoa + succ	napth2ms + succoa -> napmsccoa + succ
NAPMSCD	1- and 2-Methylnaphthalene degradation	naphthyl-2-methyl-succinyl-CoA dehydrogenase	1.3.99.-	H16_A2143/H16_A2149/H16_A2808/H16_B0675/H16_B0699	unknown/unknown/unknown/unknown/unknown/	napmsccoa -> nap2msuccoa + h2	napmsccoa -> nap2msuccoa + h2	napmsccoa -> nap2msuccoa + h2
HYDRTS	1- and 2-Methylnaphthalene degradation	hydratase	4.2.1.-	H16_A1069/H16_A1070/H16_A1289/H16_A2151/H16_A3307/H16_B0359/H16_B0706	unknown/unknown/unknown/unknown/unknown/unknown/unknown/	nap2msuccoa + h2o -> naphmsuccoa	nap2msuccoa + h2o -> naphmsuccoa	nap2msuccoa + h2o -> naphmsuccoa
THIOLS	1- and 2-Methylnaphthalene degradation	thiolase	2.3.1.-	H16_A0039/H16_A0240/H16_A0269/H16_A0699/H16_A1315/H16_A1564/H16_A1683/H16_A1802/H16_A2759/H16_A3071/H16_A3093/H16_A3221/H16_A3529/H16_A3586/H16_B0018/H16_B0021/H16_B0032/H16_B0219/H16_B1278/H16_B1292/H16_B1407/H16_B1663/H16_B1899/H16_B2397/	unknown/unknown/unknown/unknown/unknown/unknown/unknown/unknown/unknown/unknown/unknown/unknown/unknown/pat/unknown/unknown/wbpD/unknown/unknown/phnT/unknown/unknown/unknown/unknown/	napomsuccoa + coa -> 2naptcoa + succoa	napomsuccoa + coa -> 2naptcoa + succoa	napomsuccoa + coa -> 2naptcoa + succoa
2NAPTCT	1- and 2-Methylnaphthalene degradation	2-naphthoate CoA-transferase	2.8.3.-	H16_B0355/H16_B0367/H16_B0488/H16_B0655/H16_B0656/H16_B0847/H16_B0914	unknown/unknown/unknown/unknown/unknown/unknown/unknown/	2naptcoa + h2o -> 2naptha + coa + h	2naptcoa + h2o -> 2naptha + coa + h	2naptcoa + h2o -> 2naptha + coa + h
ACLDnp	1- and 2-Methylnaphthalene degradation	alcohol dehydrogenase	1.1.1.1	H16_A0757/H16_A3330/H16_B0517/H16_B1433/H16_B1699/H16_B1745/H16_B1834/H16_B2470	adh/unknown/unknown/adhP/unknown/unknown/unknown/unknown	2napthm + nad -> 2napald + nadh + h	2napthm + nad -> 2napald + nadh + h	2napthm + nad -> 2napald + nadh + h
2H4HMBZP	1- and 2-Methylnaphthalene degradation	2-hydroxy-4-hydroxymethylbenzalpyruvate hydratase-aldolase	4.2.1.-	H16_A1069/H16_A1070/H16_A1289/H16_A2151/H16_A3307/H16_B0359/H16_B0706	unknown/unknown/unknown/unknown/unknown/unknown/unknown/	2h4hmbnpyr + h2o -> 4hmscald + pyr	2h4hmbnpyr + h2o -> 4hmscald + pyr	2h4hmbnpyr + h2o -> 4hmscald + pyr
SALCH5	1- and 2-Methylnaphthalene degradation	salicylate hydroxylase	1.14.13.1	H16_A0578/H16_A0922/H16_A1785/H16_B0750/H16_B0876	unknown/unknown/unknown/unknown/unknown	4hmsalc + o2 + nadh + 2 h -> 4hmcatech + nad + h2o + co2	4hmsalc + o2 + nadh + 2 h -> 4hmcatech + nad + h2o + co2	4hmsalc + o2 + nadh + 2 h -> 4hmcatech + nad + h2o + co2
C23DDT1	1,1,1-Trichloro-2,2-Bis-(4'-Chlorophenyl)Ethane (DDT) degradation	cis-2,3-dihydrodiol DDT dehydrogenase	1.3.1.-	H16_B0731/H16_B0734	unknown/unknown	c23dhddt + nad -> 23doddt + nadh + h	c23dhddt + nad -> 23doddt + nadh + h	c23dhddt + nad -> 23doddt + nadh + h
C23DDTp	1,1,1-Trichloro-2,2-Bis-(4'-Chlorophenyl)Ethane (DDT) degradation	cis-2,3-dihydrodiol DDT dehydrogenase	1.3.1.-	H16_B0731/H16_B0734	unknown/unknown	c23dhddt + nadp -> 23doddt + nadph + 2 h	c23dhddt + nadp -> 23doddt + nadph + h	c23dhddt + nadp -> 23doddt + nadph + h
NTPPD2	1,1,1-Trichloro-2,2-Bis-(4'-Chlorophenyl)Ethane (DDT) degradation	2-nitropropane dioxygenase	1.13.11.-	H16_A0633/H16_B0223/H16_B0757/H16_B1109/H16_B1420/H16_B1836	pcaH2/unknown/unknown/unknown/unknown/unknown	23doddt + o2 -> ohtchod + h	23doddt + o2 -> ohtchod + h	23doddt + o2 -> ohtchod + h
OTO4BZ	1,1,1-Trichloro-2,2-Bis-(4'-Chlorophenyl)Ethane (DDT) degradation	multi-step reaction				ohtchod + o.5 h2 -> 2 4chbenz + h	ohtchod + 0.5 h2 -> 2 4chbenz + h	ohtchod + o.5 h2 -> 2 4chbenz + h
NTPPD3	1,1,1-Trichloro-2,2-Bis-(4'-Chlorophenyl)Ethane (DDT) degradation	2-nitropropane dioxygenase	1.13.11.-	H16_A0633/H16_B0223/H16_B0757/H16_B1109/H16_B1420/H16_B1836	pcaH2/unknown/unknown/unknown/unknown/unknown	dchdchche + o2 -> ohchtcht + h	dchdchche + o2 -> ohchtcht + h	dchdchche + o2 -> ohchtcht
PAAD8	1,1,1-Trichloro-2,2-Bis-(4'-Chlorophenyl)Ethane (DDT) degradation	phenylacrylic acid decarboxylase	4.1.1.-	H16_B2447	unknown	dda + h -> ddm + co2	dda + h -> ddm + co2	dda + h -> ddm + co2
DTO4CH	1,1,1-Trichloro-2,2-Bis-(4'-Chlorophenyl)Ethane (DDT) degradation	multi-step reaction				ddm + 3 co2 + 4 h2 -> 2 4chphac + 2 h2o + 2 h	ddm + 3 co2 + 4 h2 -> 2 4chphac + 2 h2o + 2 h	ddm + 3 co2 + 4 h2 -> 2 4chphac + 2 h2o + 2 h
ALHD15	1,2-Dichloroethane degradation	aldehyde dehydrogenase (NAD+)	1.2.1.3	H16_A0232/H16_A0745/H16_A1114/H16_A1495/H16_B0212/H16_B0421/H16_B0737/H16_B0833/H16_B1534/H16_B1735/H16_B1751/H16_B1835/H16_B1960/H16_B2444	unknown/unknown/unknown/unknown/unknown/unknown/unknown/unknown/unknown/unknown/unknown/unknown/exaC/unknown/	chacald + nad + h2o -> chac + nadh + 2 h	chacald + nad + h2o -> chac + nadh + 2 h	chacald + nad + h2o -> chac + nadh + 2 h
MACR2	1,4-Dichlorobenzene degradation	maleylacetate reductase	1.3.1.32	H16_A1786/H16_B0970	unknown/pcpE	2bromoac + nadh -> 2mac + nad + br	2bromoac + nadh -> 2mac + nad + br	2bromoac + nadh -> 2mac + nad + br
CMBLD3	1,4-Dichlorobenzene degradation	carboxymethylenebutenolidase	3.1.1.45	H16_A2215/H16_A2739/H16_A3488	unknown/unknown/unknown	c2ch4cmo + h2o -> 2chmac + h	c2ch4cmo + h2o -> 2chmac + h	c2ch4cmo + h2o -> 2chmac + h
MACR3	1,4-Dichlorobenzene degradation	maleylacetate reductase	1.3.1.32	H16_A1786/H16_B0970	unknown/pcpE	2chmac + nadh -> 2mac + nad + cl	2chmac + nadh -> 2mac + nad + cl	2chmac + nadh -> 2mac + nad + cl
FBMO10	1,4-Dichlorobenzene degradation	chlorophenol 4-monooxygenase	1.14.13.-	H16_A1145/H16_B0495/H16_B1480/H16_B2135	unknown/unknown/unknown/unknown	246tchph + 2 nadh + o2 + 2 h -> 26dchhq + 2 nad + h2o + cl	246tchph + 2 nadh + o2 + h -> 26dchhq + 2 nad + h2o + cl	246tchph + 2 nadh + o2 + h -> 26dchhq + 2 nad + h2o + cl

FBMO11	1,4-Dichlorobenzene degradation	chlorophenol 4-monooxygenase	1.14.13.-	H16_A1145/H16_B0495/H16_B1480/H16_B2135	unknown/unknown/unknown/unknown	26dchp + o2 + nadh + h -> 26dchhq + nad + h2o	26dchp + o2 + nadh + h -> 26dchhq + nad + h2o	26dchp + o2 + nadh + h -> 26dchhq + nad + h2o
CMBLD4	1,4-Dichlorobenzene degradation	carboxymethylenebutenolidase	3.1.1.45	H16_A2215/H16_A2739/H16_A3488	unknown/unknown/unknown	c4cmbo + h2o -> 2mac + h	c4cmbo + h2o -> 2mac + h	c4cmbo + h2o -> 2mac + h
CMBLD5	1,4-Dichlorobenzene degradation	carboxymethylenebutenolidase	3.1.1.45	H16_A2215/H16_A2739/H16_A3488	unknown/unknown/unknown	protmn + h2o -> cacac + h	protmn + h2o -> cacac + h	protmn + h2o -> cacac + h
MCCIS2	1,4-Dichlorobenzene degradation	muconate cycloisomerase	5.5.1.1	H16_A1966/H16_B0536	catB3/catB4	3chccm + h -> protmn + hcl + co2	3chccm + h -> protmn + hcl + co2	3chccm + h -> protmn + hcl + co2
CATCHDG4	1,4-Dichlorobenzene degradation	catechol 1,2-dioxygenase	1.13.11.1	H16_A1964/H16_B0968	catA/pcpA	4chcat + o2 -> 3chccm + 2 h	4chcat + o2 -> 3chccm + 2 h	4chcat + o2 -> 3chccm + 2 h
CATCHD4	1,4-Dichlorobenzene degradation	catechol 2,3-dioxygenase	1.13.11.2	H16_B0546	unknown	4chcat + o2 -> 5ch2hmcs + h	4chcat + o2 -> 5ch2hmcs + h	4chcat + o2 -> 5ch2hmcs + h
6CHQD	1,4-Dichlorobenzene degradation	6-Chlorobenzene-1,2,4-triol:oxygen 7S,8S-oxidoreductase				6chq + o2 -> 2chmac + 2 h	6chq + o2 -> 2chmac + 2 h	6chq + o2 -> 2chmac + 2 h
26DICH	1,4-Dichlorobenzene degradation	2,6-DiCH chlorohydolase				26dchhq + h2o -> 6chq + hcl + h	26dchhq + h2o -> 6chq + hcl	26dchhq + h2o -> 6chq + hcl
4HBZCT	2,4-Dichlorobenzoate degradation	4-hydroxybenzoyl-CoA thioesterase	3.1.2.23	H16_A1529/H16_A2456	unknown/unknown	4hbzcoa + h2o -> 4hb + coa + h	4hbzcoa + h2o -> 4hb + coa + h	4hbzcoa + h2o -> 4hb + coa + h
NTPPD4	2,4-Dichlorobenzoate degradation	2-nitropropane dioxygenase	1.13.11.-	H16_A0633/H16_B0223/H16_B0757/H16_B1109/H16_B1420/H16_B1836	pcaH2/unknown/unknown/unknown/unknown/unknown	4sulcat + o2 -> 3sulmc + 2 h	4sulcat + o2 -> 3sulmc + 2 h	4sulcat + o2 -> 3sulmc + 2 h
ESTLP	2,4-Dichlorobenzoate degradation	esterase / lipase	3.1.1.-	H16_A1422/H16_A1554/H16_A3634/H16_B0287/H16_B0633/H16_B1090/H16_B1112/H16_B1249/H16_B1475/H16_B2049/H16_B2068	unknown/unknown/unknown/unknown/unknown/unknown/unknown/unknown/unknown/cheB2/unknown	4sullac + h2o -> so3 + 2mac + h	4sullac + h2o -> so3 + 2mac + h	4sullac + h2o -> so3 + 2mac + h
ALCDt	3-Chloroacrylic acid degradation	alcohol dehydrogenase	1.1.1.1	H16_A0757/H16_A3330/H16_B0517/H16_B1433/H16_B1699/H16_B1745/H16_B1834/H16_B2470	adh/unknown/unknown/adhP/unknown/unknown/unknown/unknown	t3chp -> 3chroald + h2	t3chp -> 3chroald + h2	t3chp -> 3chroald + h2
ALHD13	3-Chloroacrylic acid degradation	aldehyde dehydrogenase (NAD+)	1.2.1.3	H16_A0232/H16_A0745/H16_A1114/H16_A1495/H16_B0212/H16_B0421/H16_B0737/H16_B0833/H16_B1534/H16_B1735/H16_B1751/H16_B1835/H16_B1960/H16_B2444	unknown/unknown/unknown/unknown/unknown/unknown/unknown/unknown/unknown/unknown/unknown/unknown/exaC/unknown/	3chroald + h2o -> t3chc + h + h2	3chroald + h2o -> t3chc + h + h2	3chroald + h2o -> t3chc + h + h2
ACLDc	3-Chloroacrylic acid degradation	alcohol dehydrogenase	1.1.1.1	H16_A0757/H16_A3330/H16_B0517/H16_B1433/H16_B1699/H16_B1745/H16_B1834/H16_B2470	adh/unknown/unknown/adhP/unknown/unknown/unknown/unknown	c3chp -> c3chroald + h2	c3chp -> c3chroald + h2	c3chp -> c3chroald + h2
ALHD14	3-Chloroacrylic acid degradation	aldehyde dehydrogenase (NAD+)	1.2.1.3	H16_A0232/H16_A0745/H16_A1114/H16_A1495/H16_B0212/H16_B0421/H16_B0737/H16_B0833/H16_B1534/H16_B1735/H16_B1751/H16_B1835/H16_B1960/H16_B2444	unknown/unknown/unknown/unknown/unknown/unknown/unknown/unknown/unknown/unknown/unknown/unknown/exaC/unknown/	c3chroald + h2o -> c3chroc + h2 + h	c3chroald + h2o -> c3chroc + h2 + h	c3chroald + h2o -> c3chroc + h2 + h
PAAD7	3-Chloroacrylic acid degradation	phenylacrylic acid decarboxylase	4.1.1.-	H16_B2447	unknown	3opp + h -> acal + co2	3opp + h -> acal + co2	3opp + h -> acal + co2
DADH	Alanine and Aspartate Metabolism	D-Amino acid dehydrogenase	1.4.99.1	H16_A0770/H16_A0817/H16_A1505/H16_B0508/H16_B1893	dadA2/dadA1/dadA5/dadA6/dadA7	dala + fad + h2o -> fadh2 + nh4 + pyr	dala + fad + h2o + h -> fadh2 + nh4 + pyr	dala + fad + h2o + h -> fadh2 + nh4 + pyr
ASPTA	Alanine and Aspartate Metabolism	aspartate transaminase	2.6.1.1	H16_A2857	unknown	akg + asp <-> glu + oaa	akg + asp <-> glu + oaa	akg + asp <-> glu + oaa
ASNN	Alanine and Aspartate Metabolism	L-asparaginase	3.5.1.1	H16_A2102	ansB	asn + h2o -> asp + nh4	asn + h2o -> asp + nh4	asn + h2o -> asp + nh4
GLNAS	Alanine and Aspartate Metabolism	glutamin-(asparagin-)ase	3.5.1.38	H16_A1910	ansA	asn + h2o -> asp + nh4	asn + h2o -> asp + nh4	asn + h2o -> asp + nh4
ALAR	Alanine and Aspartate Metabolism	alanine racemase	5.1.1.1	H16_A2113	dadX	ala <-> dala	ala <-> dala	ala <-> dala
ASNS	Alanine and Aspartate Metabolism	asparagine synthase (glutamine-hydrolysing)	6.3.5.4	H16_A3223	unknown	asp + atp + gln + h2o -> amp + asn + glu + ppi	asp + atp + gln + h2o -> amp + asn + glu + ppi + 2 h	asp + atp + gln + h2o -> amp + asn + glu + ppi + 2 h
ASP4D	Alanine and Aspartate Metabolism	aspartate 4-decarboxylase	4.1.1.12	H16_A3009	asdA	asp + h -> ala + co2	asp + h -> ala + co2	asp + h -> ala + co2
ARGSUCS	Alanine and Aspartate Metabolism	argininosuccinate synthase	6.3.4.5	H16_B2531	argG	asp + atp + citr -> amp + argsucc + ppi	asp + atp + citr -> amp + argsucc + ppi + 2 h	asp + atp + citr -> amp + argsucc + ppi + 2 h
4AMBUAT	Alanine and Aspartate Metabolism	4-aminobutyrate aminotransferase	2.6.1.19	H16_B0981	gabT	bala + akg <-> 3opp + glu	bala + akg <-> 3opp + glu	bala + akg <-> 3opp + glu
BAPYRT	Alanine and Aspartate Metabolism	beta-alanine--pyruvate transaminase	2.6.1.18	H16_A0272	aptA	ala + 3opp <-> pyr + bala	ala + 3opp <-> pyr + bala	ala + 3opp <-> pyr + bala
ASPR	Alanine and Aspartate Metabolism	aspartate racemase	5.1.1.13	H16_B2421	racX	asp <-> asp-D	asp <-> asp-D	asp <-> asp-D
LAAO1	Alanine and Aspartate Metabolism	L-amino-acid oxidase	1.4.3.2	H16_A0845/H16_A0856	lao1/lao2	asp + h2o + o2 <-> oaa + nh4 + h2o2	asp + h2o + o2 <-> oaa + nh4 + h2o2	asp + h2o + o2 <-> oaa + nh4 + h2o2
ASPOX1	Alanine and Aspartate Metabolism	L-aspartate oxidase	1.4.3.16	H16_A3036	nadB	asp + h2o + o2 <-> oaa + nh4 + h2o2	asp + h2o + o2 <-> oaa + nh4 + h2o2	asp + h2o + o2 <-> oaa + nh4 + h2o2
SUCSD1	Alanine and Aspartate Metabolism	succinate-semialdehyde dehydrogenase (NADP)	1.2.1.16	H16_B0982/H16_B1179/H16_B1537/H16_B2057	gabD1/gabD2/gabD3/gabD4	h2o + nadp + sucsal <-> 3 h + nadph + succ	h2o + nadp + sucsal <-> 2 h + nadph + succ	h2o + nadp + sucsal <-> 2 h + nadph + succ
ASP1DCB	Alanine and Aspartate Metabolism	aspartate 1-decarboxylase	4.1.1.11	H16_A3129	panD	asp + h -> bala + co2	asp + h -> bala + co2	asp + h -> bala + co2
GTADT2	Alanine and aspartate metabolism	aspartyl-tRNA(Asn)/glutamyl-tRNA (Gln) amidotransferase	6.3.5.6	H16_A0108&H16_A0111&H16_A0112&H16_A1509&H16_A1882	gatB&gatA1&gatC&gatA3&unknown	asptrna + gln + atp + h2o -> asntrna + glu + pi + adp + h	asptrna + gln + atp + h2o -> asntrna + glu + pi + adp + 2 h	asptrna + gln + atp + h2o -> asntrna + glu + pi + adp + 2 h
METTRFT	Aminoacyl-tRNA Biosynthesis	Methionyl-tRNA formyltransferase	2.1.2.9	H16_A3699	fmt	fthf + mettrna -> fmettrna + h + thf	fthf + mettrna -> fmettrna + h + thf	fthf + mettrna -> fmettrna + h + thf
TYRTRS	Aminoacyl-tRNA Biosynthesis	tyrosyl-tRNA synthetase	6.1.1.1	H16_A0490	tyrS	atp + h + trnatyr + tyr -> amp + ppi + tyrtrna	atp + trnatyr + tyr -> amp + ppi + tyrtrna + h	atp + trnatyr + tyr -> amp + ppi + tyrtrna + h
METTRS	Aminoacyl-tRNA Biosynthesis	Methionyl-tRNA synthetase	6.1.1.10	H16_A2945	metG	atp + h + met + trnamet -> amp + mettrna + ppi	atp + met + trnamet -> amp + mettrna + ppi + h	atp + met + trnamet -> amp + mettrna + ppi + h
SERTRS	Aminoacyl-tRNA Biosynthesis	Seryl-tRNA synthetase	6.1.1.11	H16_A0764/H16_B0015	serS1/serS2	atp + ser + trnaser -> amp + ppi + sertrna	atp + ser + trnaser -> amp + ppi + sertrna + 2 h	atp + ser + trnaser -> amp + ppi + sertrna + 2 h
ASPTRS	Aminoacyl-tRNA Biosynthesis	Aspartyl-tRNA synthetase	6.1.1.12	H16_A0453	aspS	asp + atp + h + trnaasp -> amp + asptrna + ppi	asp + atp + trnaasp -> amp + asptrna + ppi + h	asp + atp + trnaasp -> amp + asptrna + ppi + h
GLYTRS	Aminoacyl-tRNA Biosynthesis	Glycyl-tRNA synthetase	6.1.1.14	H16_A0521/H16_A0523	glyS/glyQ	atp + gly + h + trnagly -> amp + glytrna + ppi	atp + gly + trnagly -> amp + glytrna + ppi + h	atp + gly + trnagly -> amp + glytrna + ppi + h

PROTRS	Aminoacyl-tRNA Biosynthesis	Prolyl-tRNA synthetase	6.1.1.15	H16_A3246	proS	atp + h + pro + trnapro -> amp + ppi + protrna	atp + pro + trnapro -> amp + ppi + protrna + h	atp + pro + trnapro -> amp + ppi + protrna + h
CYSTRS	Aminoacyl-tRNA Biosynthesis	Cysteinyl-tRNA synthetase	6.1.1.16	H16_A1221	cysS	atp + cys + h + trnacys -> amp + cystrna + ppi	atp + cys + trnacys -> amp + cystrna + ppi + h	atp + cys + trnacys -> amp + cystrna + ppi + h
GLNTRS	Aminoacyl-tRNA Biosynthesis	Glutaminyl-tRNA synthetase	6.1.1.18	H16_A2784	glnS	atp + gln + h + trnagln -> amp + glntrna + ppi	atp + gln + trnagln -> amp + glntrna + ppi + h	atp + gln + trnagln -> amp + glntrna + ppi + h
ARGTRS	Aminoacyl-tRNA Biosynthesis	Arginyl-tRNA synthetase	6.1.1.19	H16_A0159	agrS	arg + atp + h + trnaarg -> amp + argtrna + ppi	arg + atp + trnaarg -> amp + argtrna + ppi + h	arg + atp + trnaarg -> amp + argtrna + ppi + h
TRPTRS	Aminoacyl-tRNA Biosynthesis	Tryptophanyl-tRNA synthetase	6.1.1.2	H16_A0515	trpS	atp + h + trnatrp + trp -> amp + ppi + trptrna	atp + trnatrp + trp -> amp + ppi + trptrna + h	atp + trnatrp + trp -> amp + ppi + trptrna + h
PHETRS	Aminoacyl-tRNA Biosynthesis	Phenylalanyl-tRNA synthetase	6.1.1.20	H16_A1343&H16_A1344	pheS&pheT	atp + h + phe + trnaphe -> amp + phetrna + ppi	atp + phe + trnaphe -> amp + phetrna + ppi + h	atp + phe + trnaphe -> amp + phetrna + ppi + h
HISTRS	Aminoacyl-tRNA Biosynthesis	Histidyl-tRNA synthetase	6.1.1.21	H16_A2363	hisS	atp + his + trnahis -> amp + histrna + ppi	atp + his + trnahis -> amp + histrna + ppi + h	atp + his + trnahis -> amp + histrna + ppi + h
THRTRS	Aminoacyl-tRNA Biosynthesis	Threonyl-tRNA synthetase	6.1.1.3	H16_A1339	thrS	atp + thr + trnathr -> amp + ppi + thrtrna	atp + thr + trnathr -> amp + ppi + thrtrna + 2 h	atp + thr + trnathr -> amp + ppi + thrtrna + 2 h
LEUTRS	Aminoacyl-tRNA Biosynthesis	Leucyl-tRNA synthetase	6.1.1.4	H16_A3139	leuS	atp + h + leu + trnaleu -> amp + leutrna + ppi	atp + leu + trnaleu -> amp + leutrna + ppi + h	atp + leu + trnaleu -> amp + leutrna + ppi + h
ILETRS	Aminoacyl-tRNA Biosynthesis	Isoleucyl-tRNA synthetase	6.1.1.5	H16_A3046	ileS	atp + h + ile + trnaile -> amp + iletrna + ppi	atp + ile + trnaile -> amp + iletrna + ppi + h	atp + ile + trnaile -> amp + iletrna + ppi + h
LYSTRS	Aminoacyl-tRNA Biosynthesis	Lysyl-tRNA synthetase	6.1.1.6	H16_A1167	lysU	atp + h + lys + trnalys -> amp + lystrna + ppi	atp + lys + trnalys -> amp + lystrna + ppi + h	atp + lys + trnalys -> amp + lystrna + ppi + h
ALATRS	Aminoacyl-tRNA Biosynthesis	Alanyl-tRNA synthetase	6.1.1.7	H16_A2769	alaS	ala + atp + h + trnaala -> alatrna + amp + ppi	ala + atp + trnaala -> alatrna + amp + ppi + h	ala + atp + trnaala -> alatrna + amp + ppi + h
VALTRS	Aminoacyl-tRNA Biosynthesis	Valyl-tRNA synthetase	6.1.1.9	H16_A2751	valS	atp + h + trnaval + val -> amp + ppi + valtrna	atp + trnaval + val -> amp + ppi + valtrna + h	atp + trnaval + val -> amp + ppi + valtrna + h
ACG6PD	Aminosugars metabolism	N-acetylglucosamine-6-phosphate deacetylase	3.5.1.25	H16_A0314	nagA	naga6p + h2o -> ac + ga6p	naga6p + h2o -> ac + ga6p	naga6p + h2o -> ac + ga6p
GM6PD	Aminosugars metabolism	glucosamine-6-phosphate deaminase	3.5.99.6	H16_A0315	nagB	ga6p + h2o -> f6p + nh4	ga6p + h2o -> f6p + nh4	ga6p + h2o -> f6p + nh4
UAEPGR	Aminosugars metabolism	UDP-N-acetylenolpyruvoylglucosamine reductase	1.1.1.158	H16_A3061	murB	2 h + nadph + uaccg -> nadp + udpnam	h + nadph + uaccg -> nadp + udpnam	h + nadph + uaccg -> nadp + udpnam
GA1PACT	Aminosugars metabolism	glucosamine-1-phosphate N-acetyltransferase	2.3.1.157	H16_A0262	glmU	accoa + ga1p -> naga1p + coa + h	accoa + ga1p -> naga1p + coa + h	accoa + ga1p -> naga1p + coa
UNAGCVT	Aminosugars metabolism	UDP-N-acetylglucosamine 1-carboxyvinyltransferase	2.5.1.7	H16_A3418	murA	pep + udpnag -> pi + uaccg	pep + udpnag -> h + pi + uaccg	pep + udpnag -> h + pi + uaccg
GF6PT	Aminosugars metabolism	glutamine-fructose-6-phosphate transaminase	2.6.1.16	H16_A0263	glmS	f6p + gln -> ga6p + glu	f6p + gln -> ga6p + glu	f6p + gln -> ga6p + glu
UNAGDP	Aminosugars metabolism	UDP-N-acetylglucosamine diphosphorylase	2.7.7.23	H16_A0262	glmU	naga1p + utp -> ppi + udpnag	naga1p + utp -> ppi + udpnag	naga1p + utp -> ppi + udpnag
PGAMT	Aminosugars metabolism	phosphoglucosamine mutase	5.4.2.10	H16_A2445	manB3	ga1p <-> ga6p	ga1p <-> ga6p	ga1p + h <-> ga6p
ACMANAPP	Aminosugars metabolism	predicted acid phosphatase (N-Acetyl-D-mannosamine)	3.1.3.-	H16_A0168/H16_A0520/H16_B0594/H16_B1063	aceK/unknown/unknown/unknown	nadma + pi <-> nadma6p + h2o	nadma + pi <-> nadma6p + h2o	nadma + pi <-> nadma6p + h2o
UDPACG	Aminosugars metabolism	UDP-N-acetylglucosamine 4-epimerase	5.1.3.7			udpnag <-> udpacgal	udpnag <-> udpacgal	udpnag <-> udpacgal
ME1	Anaplerotic Reactions	malic enzyme (NAD)	1.1.1.38	H16_A3153	maeA	mal + nad -> co2 + nadh + pyr	mal + nad -> co2 + nadh + pyr	mal + nad -> co2 + nadh + pyr
ME2	Anaplerotic Reactions	malic enzyme (NADP)	1.1.1.40	H16_A1002	maeB	mal + nadp -> co2 + h + nadph + pyr	mal + nadp -> co2 + nadph + pyr	mal + nadp -> co2 + nadph + pyr
PPA1	Anaplerotic Reactions	inorganic diphosphatase	3.6.1.1	H16_A0746	ppa	h2o + ppi -> 2 pi	h2o + ppi -> 2 pi + h	h2o + ppi -> 2 pi
PPA2	Anaplerotic Reactions	inorganic triphosphatase	3.6.1.25			h2o + pppi -> pi + ppi	h2o + pppi -> 2 h + pi + ppi	h2o + pppi -> h + pi + ppi
PPC	Anaplerotic Reactions	phosphoenolpyruvate carboxylase	4.1.1.31	H16_A2921	ppc	co2 + h2o + pep -> h + oaa + pi	h2o + pep + co2 -> 2 h + oaa + pi	co2 + h2o + pep -> 2 h + oaa + pi
PPCK	Anaplerotic Reactions	phosphoenolpyruvate carboxykinase	4.1.1.32	H16_A3711	pepck	atp + h + oaa -> adp + co2 + pep	atp + h + oaa -> adp + co2 + pep	atp + h + oaa -> adp + co2 + pep
ICL	Anaplerotic Reactions	Isocitrate lyase	4.1.3.1	H16_A2211/H16_A2227	iclA/iclB	icit -> glx + succ	icit -> glx + succ	icit -> glx + succ
MALS	Anaplerotic Reactions	malate synthase	4.1.3.2 (2.3.3.9 in kegg)	H16_A2217	aceB	accoa + glx + h2o -> coa + h + mal	accoa + glx + h2o -> coa + h + mal	accoa + glx + h2o -> coa + h + mal
P5CD1	Arginine and Proline Metabolism	1-pyrroline-5-carboxylate dehydrogenase	1.5.1.12	H16_A3631	putA	p5c + 2 h2o + nad -> glu + 2 h + nadh	p5c + 2 h2o + nad -> glu + h + nadh	p5c + 2 h2o + nad -> glu + h + nadh
P5CR1	Arginine and Proline Metabolism	pyrroline-5-carboxylate reductase	1.5.1.2	H16_A3106	proC	p5c + 2 h + nadph -> nadp + pro	p5c + 2 h + nadph -> nadp + pro	p5c + 2 h + nadph -> nadp + pro
PROD2	Arginine and Proline Metabolism	Proline dehydrogenase	1.5.99.8	H16_A3631	putA	fad + pro -> p5c + fadh2	fad + pro -> p5c + fadh2	fad + pro -> p5c + fadh2
ORNCBT	Arginine and Proline Metabolism	ornithine carbamoyltransferase	2.1.3.3	H16_A3063	argF	cap + orn <-> citr + h + pi	cap + orn <-> citr + h + pi	cap + orn <-> citr + h + pi
ARGSCL	Arginine and Proline Metabolism	argininosuccinate lyase	4.3.2.1	H16_A2925	argH	argsucc <-> arg + fum	argsucc <-> arg + fum	argsucc <-> arg + fum
GLU5SDT	Arginine and Proline Metabolism	L-glutamate 5-semialdehyde dehydratase (spontaneous)	spontaneous	spontaneous	spontaneous	glugsal -> p5c + h2o	glugsal -> p5c + h + h2o	glugsal -> p5c + h + h2o
GLUDH4	Arginine and Proline metabolism	glutamate dehydrogenase	1.4.1.3	H16_A0471	gdhA1	glu + nad + h2o <-> akg + nh4 + nadh + h	glu + nad + h2o <-> akg + nh4 + nadh + h	glu + nad + h2o <-> akg + nh4 + nadh + h
P5CD2	Arginine and Proline metabolism	1-pyrroline-5-carboxylate dehydrogenase	1.5.1.12	H16_A3631	putA	glugsal + nad + h2o <-> glu + nadh + 2 h	glugsal + nad + h2o <-> glu + nadh + 2 h	glugsal + nad + h2o <-> glu + nadh + 2 h
ORNC	Arginine and Proline metabolism	ornithine cyclodeaminase	4.3.1.12	H16_A0689/H16_A1394/H16_A3673/H16_B1881	unknown/arcB/unknown/unknown	orn <-> pro + nh4	orn <-> pro + nh4	orn <-> pro + nh4
P4HX	Arginine and Proline metabolism	prolyl 4-hydroxylase	1.14.11.2	H16_A3244	phy	pro + akg + o2 -> 4hpro + succ + co2	pro + akg + o2 -> 4hpro + succ + co2	pro + akg + o2 -> 4hpro + succ + co2
P5CD3	Arginine and Proline metabolism	1-pyrroline-5-carboxylate dehydrogenase	1.5.1.12	H16_A3631	putA	4hpro + fad -> l1p3h5c + fadh2 + h	4hpro + fad -> l1p3h5c + fadh2	4hpro + fad -> l1p3h5c + fadh2
PROD3	Arginine and Proline metabolism	Proline dehydrogenase	1.5.99.8	H16_A3631	putA	4hpro + fad -> l1p3h5c + fadh2 + h	4hpro + fad -> l1p3h5c + fadh2	4hpro + fad -> l1p3h5c + fadh2
P5CR2	Arginine and Proline metabolism	pyrroline-5-carboxylate reductase	1.5.1.2	H16_A3106	proC	l1p3h5c + nadh + 2 h -> 4hpro + nad	l1p3h5c + nadh + 2 h -> 4hpro + nad	l1p3h5c + nadh + 2 h -> 4hpro + nad
P5CD4	Arginine and Proline metabolism	1-pyrroline-5-carboxylate dehydrogenase	1.5.1.12	H16_A3631	putA	l1p3h5c + nad + 2 h2o -> e4hglu + nadh + h	l1p3h5c + nad + 2 h2o -> e4hglu + nadh + h	l1p3h5c + nad + 2 h2o -> e4hglu + nadh + h
PTO4H	Arginine and Proline metabolism					l1p3h5c + h2o + h <-> 4hglusa	l1p3h5c + h2o + h <-> 4hglusa	l1p3h5c + h2o + h <-> 4hglusa
P5CD5	Arginine and Proline metabolism	1-pyrroline-5-carboxylate dehydrogenase	1.5.1.12	H16_A3631	putA	e4hglu + nad <-> 4hglusa + nadh + h2o	e4hglu + nad <-> 4hglusa + nadh + h2o	e4hglu + nad <-> 4hglusa + nadh + h2o

PROD4	Arginine and Proline metabolism	Proline dehydrogenase	1.5.99.8	H16_A3631	putA	e4hglu + nad <-> 4hglusa + nadh + h2o	e4hglu + nad <-> 4hglusa + nadh + h2o	e4hglu + nad <-> 4hglusa + nadh + h2o
DATA2	Arginine and Proline metabolism	D-alanine transaminase	2.6.1.21	H16_A2521	dat	e4hglu + oaa -> asp-D + hydroxyakg	e4hglu + oaa -> asp-D + hydroxyakg	e4hglu + oaa -> asp-D + hydroxyakg
ASPAM4	Arginine and Proline metabolism	aspartate aminotransferase	2.6.1.1	H16_A2857	unknown	e4hglu + akg -> hydroxyakg + glu	e4hglu + akg -> hydroxyakg + glu	e4hglu + akg -> hydroxyakg + glu
HOGAD	Arginine and Proline metabolism	4-hydroxy-2-oxoglutarate aldolase	4.1.2.14	H16_B1213	eda	hydroxyakg <-> pyr + glx	hydroxyakg <-> pyr + glx	hydroxyakg <-> pyr + glx
CREAH	Arginine and Proline metabolism	creatinine amidohydrolase	3.5.2.10	H16_A1736	unknown	cretn + h2o -> creatine + h	cretn + h2o -> creatine	cretn + h2o -> creatine
CARSA	Arginine and Proline metabolism	N-carbamoylsarcosine amidase	3.5.1.59	H16_A0926	unknown	carbs + h2o + 2 h -> sarcs + co2 + nh4	carbs + h2o + 2 h -> sarcs + co2 + nh4	carbs + h2o + 2 h -> sarcs + co2 + nh4
P5CD1p	Arginine and Proline Metabolism	1-pyrroline-5-carboxylate dehydrogenase	1.5.1.12	H16_A3631	putA	p5c + 2 h2o + nadp -> glu + 3 h + nadph	p5c + 2 h2o + nadp -> glu + h + nadph	p5c + 2 h2o + nadp -> glu + h + nadph
P5CR2p	Arginine and Proline metabolism	pyrroline-5-carboxylate reductase	1.5.1.2	H16_A3106	proC	l1p3h5c + nadph + 3 h -> 4hpro + nadp	l1p3h5c + nadph + 2 h -> 4hpro + nadp	l1p3h5c + nadph + 2 h -> 4hpro + nadp
GLTN1	Ascorbate and Aldarate metabolism	gluconolactonase	3.1.1.17	H16_A3012/H16_B0345/ H16_B1441	gnl1/gnl2/gnl3	g14l + h2o <-> guln + h	g14l + h2o <-> guln + h	g14l + h2o <-> guln + h
GLCRDH	Ascorbate and Aldarate metabolism	glucarate dehydratase	4.2.1.40	H16_B0127	unknown	dgluca -> d4dg + h2o	dgluca -> d4dg + h2o	dgluca -> d4dg + h2o
GALCTDH	Ascorbate and Aldarate metabolism	galactarate dehydratase	4.2.1.42	H16_A1258/H16_A1259/ H16_B0965	unknown/unknown/ unknown	dgal -> d4dg + h2o	dgal -> d4dg + h2o	dgal -> d4dg + h2o
ADLD	Ascorbate and Aldarate metabolism	aldehyde dehydrogenase (NAD+)	1.2.1.3	H16_A0232/H16_A0745/ H16_A1114/H16_A1495/ H16_B0212/H16_B0421/ H16_B0737/H16_B0833/ H16_B1534/H16_B1735/ H16_B1751/H16_B1835/ H16_B1960/H16_B2444	unknown/unknown/ unknown/unknown/ unknown/unknown/ unknown/unknown/ unknown/unknown/ unknown/unknown/ exaC/unknown/	dglucl + nad + 2 h2o <-> dgluca + nadh + 3 h	dglucl + nad + 2 h2o <-> dgluca + nadh + 3 h	dglucl + nad + 2 h2o <-> dgluca + nadh + 3 h
GLCRD2	Ascorbate and Aldarate metabolism	glucarate dehydratase	4.2.1.40	H16_B0127	unknown	dgluca <-> d3dg + h2o	dgluca <-> d3dg + h2o	dgluca <-> d3dg + h2o
DDGD	Ascorbate and Aldarate metabolism	5-dehydro-4-deoxyglucarate dehydratase	4.2.1.41	H16_B0131	unknown	d4dg <-> 25dop + h2o + co2	d4dg <-> 25dop + h2o + co2	d4dg <-> 25dop + h2o + co2
CCPPMD	Atrazine degradation	N-cyclopropylmelamine deaminase	3.5.4.-	H16_B0862	unknown	cyromz + h2o -> ccpam + nh4	cyromz + h2o + h -> ccpam + nh4	cyromz + h2o + h -> ccpam + nh4
CCPPAMD	Atrazine degradation	N-cyclopropylammeline deaminase	3.5.4.-	H16_B0862	unknown	ccpam + h2o + h -> ccpamd + nh4	ccpam + h2o + h -> ccpamd + nh4	ccpam + h2o + h -> ccpamd + nh4
CCPAMAH	Atrazine degradation	N-cyclopropylammelide alkylamino hydrolase	3.5.4.-	H16_B0862	unknown	ccpamd + h2o + h -> cyanr + ccppam	ccpamd + h2o + h -> cyanr + ccppam	ccpamd + h2o + h -> cyanr + ccppam
HATRAZE1	Atrazine degradation	hydroxyatrazine ethylaminohydrolase	3.5.99.3	H16_A1363	unknown	hatraz + h2o + h -> isoppam + ethlam	hatraz + h2o + h -> isoppam + ethlam	hatraz + h2o + h -> isoppam + ethlam
HATRAZE2	Atrazine degradation	hydroxyatrazine ethylaminohydrolase	3.5.99.3	H16_A1363	unknown	2c4h6at + h2o -> ammld + hcl + h	2c4h6at + h2o -> ammld + hcl + h	2c4h6at + h2o -> ammld + hcl + h
4HBZDC	Benzoate degradation via CoA ligation	4-hydroxybenzoate decarboxylase	4.1.1.61	H16_B2446	unknown	phenol + co2 -> 4hb + h	phenol + co2 -> 4hb + h	phenol + co2 -> 4hb + h
NITL5	Benzoate degradation via CoA ligation	nitrilase	3.5.5.1	H16_A1125	nit	bzonit + 2 h2o -> benzot + nh4	bzonit + 2 h2o -> benzot + nh4	bzonit + 2 h2o -> benzot + nh4
AMDS6	Benzoate degradation via CoA ligation	amidase	3.5.1.4	H16_A1469/H16_B1874/ H16_B2459	unknown/unknown/ aimE	bzamid + h2o -> benzot + nh4	bzamid + h2o -> benzot + nh4	bzamid + h2o -> benzot + nh4
BZOTD1	Benzoate degradation via CoA ligation	benzoate 1,2-dioxygenase	1.14.12.10	H16_A1961&H16_A196 2&H16_A1963	benC&benB&benA	benzot + nadh + h + o2 -> 16dh24checc + nad	benzot + nadh + h + o2 -> 16dh24checc + nad	benzot + nadh + h + o2 -> 16dh24checc + nad
BZOTD1p	Benzoate degradation via CoA ligation	benzoate 1,2-dioxygenase	1.14.12.10	H16_A1961&H16_A196 2&H16_A1963	benC&benB&benA	benzot + nadph + 2 h + o2 -> 16dh24checc + nadp	benzot + nadph + h + o2 -> 16dh24checc + nadp	benzot + nadph + h + o2 -> 16dh24checc + nadp
16DCDC1	Benzoate degradation via CoA ligation	1,6-dihydroxycyclohexa-2,4-diene-1-carboxylate dehydrogenase	1.3.1.25	H16_A1960	benD	16dh24checc + nad -> catech + nadh + co2	16dh24checc + nad -> catech + nadh + co2	16dh24checc + nad -> catech + nadh + co2
APPS3	Benzoate degradation via CoA ligation	acylphosphatase	3.6.1.7	H16_A3325	acyP	bzop + h2o -> benzot + pi	bzop + h2o -> benzot + pi + h	bzop + h2o -> benzot + pi + h
BZOTCOA	Benzoate degradation via CoA ligation	benzoate-CoA ligase	6.2.1.25	H16_A1412/H16_B1918	unknown/unknown	benzot + atp + coa + h -> amp + ppi + bzocoa	benzot + atp + coa -> amp + ppi + bzocoa + h	benzot + atp + coa -> amp + ppi + bzocoa + h
ACDH5	Benzoate degradation via CoA ligation	acyl dehydratase	4.2.1.-	H16_A1069/H16_A1070/ H16_A1289/H16_A2151/ H16_A3307/H16_B0359/ H16_B0706	unknown/unknown/ unknown/unknown/ unknown/unknown/ unknown/	6hcecoa + h2o -> 26dhccoa	6hcecoa + h2o -> 26dhccoa	6hcecoa + h2o -> 26dhccoa
2KCHCCH	Benzoate degradation via CoA ligation	2-ketocyclohexanecarboxyl-CoA hydrolase	3.1.2.-	H16_A3190/H16_B1690/ H16_B1691/H16_B1864	unknown/unknown/ unknown/unknown	2kchccoa + h2o -> pmcoa	2kchccoa + h2o -> pmcoa	2kchccoa + h2o -> pmcoa + h
CCHCCOAL	Benzoate degradation via CoA ligation	cyclohexanecarboxylate-CoA ligase	6.2.1.-	H16_B1693	unknown	cchc + coa + atp + h -> cchccoa + amp + ppi	cchc + coa + atp -> cchccoa + amp + ppi + h	cchc + coa + atp -> cchccoa + amp + ppi + h
2HCHCCOA	Benzoate degradation via CoA ligation	2-hydroxycyclohexanecarboxyl-CoA dehydrogenase	1.1.1.-	H16_B1696	unknown	hcchccoa + nad -> 2kchccoa + nadh + h	hcchccoa + nad -> 2kchccoa + nadh + h	hcchccoa + nad -> 2kchccoa + nadh + h
PMCOAD	Benzoate degradation via CoA ligation	pimeloyl-CoA dehydrogenase	1.3.1.62	H16_B0371&H16_B0372	pimC&pimD	pmcoa + nad -> 6checoa + nadh + 2 h	pmcoa + nad -> 6checoa + nadh + 2 h	pmcoa + nad -> 6checoa + nadh + h
ACDH6	Benzoate degradation via CoA ligation	acyl dehydratase	4.2.1.-	H16_A1069/H16_A1070/ H16_A1289/H16_A2151/ H16_A3307/H16_B0359/ H16_B0706	unknown/unknown/ unknown/unknown/ unknown/unknown/ unknown/	6checoa + h2o -> hpimcoa	6checoa + h2o -> hpimcoa	6checoa + h2o -> hpimcoa
ACTF4	Benzoate degradation via CoA ligation	acetyltransferase	2.3.1.-	H16_A0039/H16_A0240/ H16_A0269/H16_A0699/ H16_A1315/H16_A1564/ H16_A1683/H16_A1802/ H16_A2759/H16_A3071/ H16_A3093/H16_A3221/ H16_A3529/H16_A3586/ H16_B0018/H16_B0021/ H16_B0032/H16_B0219/ H16_B1278/H16_B1292/ H16_B1407/H16_B1663/ H16_B1899/H16_B2397/	unknown/unknown/ unknown/unknown/ unknown/unknown/ unknown/unknown/ unknown/unknown/ unknown/unknown/ unknown/pat/unkno wn/unknown/wbpD/ unknown/unknown/ phnT/unknown/unkn own/unknown/unkn own/	opimcoa + coa -> glutcoa + accoa	opimcoa + coa -> glutcoa + accoa	opimcoa + coa -> glutcoa + accoa
GLUCD2	Benzoate degradation via CoA ligation	glutaryl-CoA dehydrogenase	1.3.99.7	H16_A2818	gcdH	glutcoa + fad -> gl1coa + fadh2	glutcoa + fad + h -> gl1coa + fadh2	glutcoa + fad + h -> gl1coa + fadh2

CCPPCCOAL	Benzoate degradation via CoA ligation	cyclopropanecarboxylate:CoA ligase	6.2.1.-	H16_A0866/H16_A0871/H16_A1230/H16_A1519/H16_A1700/H16_A1718/H16_A2252/H16_A2794/H16_A2807/H16_A2978/H16_B0174/H16_B0677/H16_B0910/H16_B1148/H16_B1264/H16_B1335/H16_B1662/H16_B1709/H16_B2522	unknown/unknown/unknown/unknown/unknown/unknown/unknown/unknown/unknown/unknown/unknown/unknown/unknown/unknown/unknown/unknown/unknown/unknown/unknown/	ccppc + coa + atp + h -> ccppccoa + adp + pi	ccppc + coa + atp -> ccppccoa + adp + pi	ccppc + coa + atp -> ccppccoa + adp + pi
3HBCDH	Benzoate degradation via CoA ligation	3-hydroxybutyryl-CoA dehydrogenase	1.1.1.157	H16_A1102	paaH2	3hbcoa + nadp -> aacoa + nadph + 2 h	3hbcoa + nadp -> aacoa + nadph + h	3hbcoa + nadp -> aacoa + nadph + h
ACTF5	Benzoate degradation via CoA ligation	acetyltransferase	2.3.1.-	H16_A0039/H16_A0240/H16_A0269/H16_A0699/H16_A1315/H16_A1564/H16_A1683/H16_A1802/H16_A2759/H16_A3071/H16_A3093/H16_A3221/H16_A3529/H16_A3586/H16_B0018/H16_B0021/H16_B0032/H16_B0219/H16_B1278/H16_B1292/H16_B1407/H16_B1663/H16_B1899/H16_B2397/	unknown/unknown/unknown/unknown/unknown/unknown/unknown/unknown/unknown/unknown/unknown/unknown/unknown/pat/unknown/unknown/wbpD/unknown/unknown/phnT/unknown/unknown/unknown/unknown/	3h5ohcoa + coa -> 3hbcoa + accoa	3h5ohcoa + coa -> 3hbcoa + accoa	3h5ohcoa + coa -> 3hbcoa + accoa
BZALDD6	Benzoate degradation via Hydroxylation	benzaldehyde dehydrogenase (NAD)	1.2.1.28	H16_A1772	unknown	4hbzald + nad + h2o <-> 4hb + nadh + 2 h	4hbzald + nad + h2o <-> 4hb + nadh + 2 h	4hbzald + nad + h2o <-> 4hb + nadh + h
PHBZMN	Benzoate degradation via Hydroxylation	p-hydroxybenzoate 3-monooxygenase	1.14.13.2	H16_B2286	pobA	4hb + o2 + nadph + 2 h -> 34dhb + nadp + h2o	4hb + o2 + nadph + h -> 34dhb + nadp + h2o	4hb + o2 + nadph + h -> 34dhb + nadp + h2o
PROTCC1	Benzoate degradation via Hydroxylation	protocatechuate 3,4-dioxygenase	1.13.11.3	H16_B0795/(H16_B2290&H16_B2291)	unknown/(pcaG&pcaH1)	34dhb + o2 -> carccm + 2 h	34dhb + o2 -> carccm + 2 h	34dhb + o2 -> carccm + 2 h
PAAD9	Benzoate degradation via Hydroxylation	phenylacrylic acid decarboxylase	4.1.1.-	H16_B2447	unknown	zoe + h2o + h -> hopt + co2	zoe + h2o + h -> hopt + co2	zoe + h2o + h -> hopt + co2
OXCTD2	Benzoate degradation via Hydroxylation	4-oxalocrotonate decarboxylase	4.1.1.77	H16_B0549	unknown	zoe + h -> op4en + co2	zoe + h -> op4en + co2	zoe + h -> op4en + co2
4OXCTT	Benzoate degradation via Hydroxylation	4-oxalocrotonate tautomerase	5.3.2.-	H16_A2763/H16_A3184/H16_B0550	unknown/unknown/unknown	2hmuc -> zoe	2hmuc -> zoe	2hmuc -> zoe
CATCHDG5	Benzoate degradation via Hydroxylation	catechol 1,2-dioxygenase	1.13.11.1	H16_A1964/H16_B0968	catA/pcpA	catech + o2 -> ccmuc + 2 h	catech + o2 -> ccmuc + 2 h	catech + o2 -> ccmuc + 2 h
CATCHD5	Benzoate degradation via Hydroxylation	catechol 2,3-dioxygenase	1.13.11.2	H16_B0546	unknown	catech + o2 -> 2hmucsald + h	catech + o2 -> 2hmucsald + h	catech + o2 -> 2hmucsald + h
MCCIS3	Benzoate degradation via Hydroxylation	muconate cycloisomerase	5.5.1.1	H16_A1966/H16_B0536	catB3/catB4	ccmuc + h <-> mclact	ccmuc + h <-> mclact	ccmuc + h <-> mclact
MCLACi	Benzoate degradation via Hydroxylation	muconolactone D-isomerase	5.3.3.4	H16_A1967/H16_B1584	catC3/catC2	mclact <-> 2odhfac	mclact <-> 2odhfac	mclact <-> 2odhfac
3OXAPLC	Benzoate degradation via Hydroxylation	3-oxoadipate enol-lactonase	3.1.1.24	H16_A0147/H16_A1968/H16_B1583	unknown/catD1/catD2	2odhfac + h2o -> oadip + h	2odhfac + h2o -> oadip + h	2odhfac + h2o -> oadip + h
3OXAPT	Benzoate degradation via Hydroxylation	3-oxoadipate CoA-transferase	2.8.3.6	H16_B0198/H16_B0199	pcaI/pcaJ	succoa + oadip -> succ + ooadpcoa	succoa + oadip -> succ + ooadpcoa	succoa + oadip -> succ + ooadpcoa
3OXADCT	Benzoate degradation via Hydroxylation	3-oxoadipyl-CoA thiolase	2.3.1.16	H16_A0462/H16_A1290/H16_B0200	unknown/unknown/pcaF	ooadpcoa + coa -> succoa + accoa	ooadpcoa + coa -> succoa + accoa	ooadpcoa + coa -> succoa + accoa
HMSALD3	Benzoate degradation via Hydroxylation	2-hydroxymuconic semialdehyde dehydrogenase	1.2.1.32	H16_B0547	unknown	2hmucsald + nad + h2o -> 2hmuc + nadh + 2 h	2hmucsald + nad + h2o -> 2hmuc + nadh + 2 h	2hmucsald + nad + h2o -> 2hmuc + nadh + 2 h
CATCHD6	Benzoate degradation via Hydroxylation	catechol 2,3-dioxygenase	1.13.11.2	H16_B0546	unknown	sulcatech + o2 + h2o -> 2hmuc + so3 + 2 h	sulcatech + o2 + h2o -> 2hmuc + so3 + 2 h	sulcatech + o2 + h2o -> 2hmuc + so3 + 2 h
3CMUCC	Benzoate degradation via Hydroxylation	3-carboxy-cis,cis-muconate cycloisomerase	5.5.1.2	H16_A2422/H16_B2289	pcaB2/pcaB1	carccm + h <-> gcarmclc	carccm + h <-> gcarmclc	carccm + h <-> gcarmclc
4CBMCLC	Benzoate degradation via Hydroxylation	4-carboxymuconolactone decarboxylase	4.1.1.44	H16_A0535/H16_B2288	unknown/pcaCD	gcarmclc + h <-> 2odhfac + co2	gcarmclc + h <-> 2odhfac + co2	gcarmclc + h <-> 2odhfac + co2
PROTCD	Benzoate degradation via Hydroxylation	protocatechuate 3,4-dioxygenase	1.13.11.-	H16_A0633/H16_B0223/H16_B0757/H16_B1109/H16_B1420/H16_B1836	pcaH2/unknown/unknown/unknown/unknown/unknown	34dhb + o2 + nadh + 2 h -> thbn + co2 + nad + h2o	34dhb + o2 + nadh + 2 h -> thbn + co2 + nad + h2o	34dhb + o2 + nadh + 2 h -> thbn + co2 + nad + h2o
MACR4	Benzoate degradation via Hydroxylation	maleylacetate reductase	1.3.1.32	H16_A1786/H16_B0970	unknown/pcpE	2mac + nadh + h -> oadip + nad	2mac + nadh + h -> oadip + nad	2mac + nadh + h -> oadip + nad
BZFORCL2	Benzoate degradation via Hydroxylation	benzoylformate carboxy-lyase	4.1.1.7	H16_A1113	unknown	4hbzald + co2 -> 4hpheglyx + h	4hbzald + co2 -> 4hpheglyx + h	4hbzald + co2 -> 4hpheglyx + h
MCCIS4	Benzoate degradation via Hydroxylation	muconate cycloisomerase	5.5.1.1	H16_A1966/H16_B0536	catB3/catB4	4c2hmucsah -> 2h2hpd	4c2hmucsah -> 2h2hpd	4c2hmucsah -> 2h2hpd
PROTCC2	Benzoate degradation via Hydroxylation	protocatechuate 3,4-dioxygenase	1.13.11.3	H16_B0795/(H16_B2290&H16_B2291)	unknown/(pcaG&pcaH1)	gallate + o2 -> 2py46dc + h2o + h	gallate + o2 -> 2py46dc + h2o + h	gallate + o2 -> 2py46dc + h2o + h
CARHM2	Benzoate degradation via Hydroxylation	5-carboxymethyl-2-hydroxymuconate isomerase	5.3.3.10	H16_A0624/H16_B1250	hpaF/unknown	4c2hhd <-> 4obtc	4c2hhd <-> 4obtc	4c2hhd <-> 4obtc
ACDH7	Benzoate degradation via Hydroxylation	acyl dehydratase	4.2.1.-	H16_A1069/H16_A1070/H16_A1289/H16_A2151/H16_A3307/H16_B0359/H16_B0706	unknown/unknown/unknown/unknown/unknown/unknown/unknown/	4c2hmucsah + h2o -> 4c4h2oadip	4c2hmucsah + h2o -> 4c4h2oadip	4c2hmucsah + h2o -> 4c4h2oadip
ACDH8	Benzoate degradation via Hydroxylation	acyl dehydratase	4.2.1.-	H16_A1069/H16_A1070/H16_A1289/H16_A2151/H16_A3307/H16_B0359/H16_B0706	unknown/unknown/unknown/unknown/unknown/unknown/unknown/	4c2o4pent + h2o -> ppyr	4c2o4pent + h2o -> ppyr	4c2o4pent + h2o -> ppyr
3HBZOR	Benzoate degradation via hydroxylation	3-hydroxybenzoate,NADH:oxygen oxidoreductase	1.14.13.24			3hbenzot + o2 + nadh + h -> gensa + nad + h2o	3hbenzot + o2 + nadh + h -> gensa + nad + h2o	3hbenzot + o2 + nadh + h -> gensa + nad + h2o
PROTCDp	Benzoate degradation via Hydroxylation	protocatechuate 3,4-dioxygenase	1.13.11.-	H16_A0633/H16_B0223/H16_B0757/H16_B1109/H16_B1420/H16_B1836	pcaH2/unknown/unknown/unknown/unknown/unknown	34dhb + o2 + nadph + 3 h -> thbn + co2 + nadp + h2o	34dhb + o2 + nadph + 2 h -> thbn + co2 + nadp + h2o	34dhb + o2 + nadph + 2 h -> thbn + co2 + nadp + h2o
MACR4p	Benzoate degradation via Hydroxylation	maleylacetate reductase	1.3.1.32	H16_A1786/H16_B0970	unknown/pcpE	2mac + nadph + 2 h -> oadip + nadp	2mac + nadph + h -> oadip + nadp	2mac + nadph + h -> oadip + nadp
ANDO	Benzoate degradation via hydroxylation	anthranilate 1,2-dioxygenase	1.14.12.1			an + 1.25 o2 + 3 h + nadh -> catech + nh4 + co2 + nad + 0.5 h2o	an + 1.25 o2 + 3 h + nadh -> catech + nh4 + co2 + nad + 0.5 h2o	an + 1.25 o2 + 3 h + nadh -> catech + nh4 + co2 + nad + 0.5 h2o
DMRATT	Biosynthesis of steroids	geranyltranstransferase	2.5.1.10	H16_A2733	ispa	dmpp + ipp -> gpp + ppi	dmpp + ipp -> gpp + ppi + h	dmpp + ipp -> gpp + ppi + h
GRATT	Biosynthesis of steroids	geranyltranstransferase	2.5.1.10	H16_A2733	ispa	gpp + ipp -> frdp + ppi	gpp + ipp -> frdp + ppi + h	gpp + ipp -> frdp + ppi + h
CDPMDEK	Biosynthesis of steroids	4-(cytidine 5'-diphospho)-2-C-methyl-D-erythritol kinase	2.7.1.148	H16_A0374	unknown	cdpmde + atp -> 2pcdpmde + adp	cdpmde + atp -> 2pcdpmde + adp + h	cdpmde + atp -> 2pcdpmde + adp + h

HMB4PPR	Biosynthesis of steroids	1-hydroxy-2-methyl-2-(E)-butenyl 4-diphosphate reductase (dmpp)	1.17.1.2	H16_A3031/H16_B2169	ispH	h + hmb4pp + nadh -> dmpp + h2o + nad	h + hmb4pp + nadh -> dmpp + h2o + nad	h + hmb4pp + nadh -> dmpp + h2o + nad
DOXRTI	Biosynthesis of steroids	1-deoxy-D-xylulose reductoisomerase	1.1.1.267	H16_A2049	dxp	dx5p + 2 h + nadph -> mde4p + nadp	dx5p + h + nadph -> mde4p + nadp	dx5p + h + nadph -> mde4p + nadp
DOXPS	Biosynthesis of steroids	1-deoxy-D-xylulose 5-phosphate synthase	2.2.1.7	H16_A2732	dxs	g3p + h + pyr -> co2 + dx5p	g3p + h + pyr -> co2 + dx5p	g3p + h + pyr -> co2 + dx5p
HMB4DPR	Biosynthesis of steroids	1-hydroxy-2-methyl-2-(E)-butenyl 4-diphosphate reductase	1.17.1.2	H16_A3031/H16_B2169	ispH/ispH	h + hmb4pp + nadh -> h2o + ipp + nad	h + hmb4pp + nadh -> h2o + ipp + nad	h + hmb4pp + nadh -> h2o + ipp + nad
MECDPDHT	Biosynthesis of steroids	2C-methyl-D-erythritol 2,4 cyclodiphosphate dehydratase	1.17.4.3	H16_A2364	ispG	mdecpp + h + nadh -> hmb4pp + h2o + nad	mdecpp + h + nadh -> hmb4pp + h2o + nad	mdecpp + nadh -> hmb4pp + h2o + nad
MECDPS	Biosynthesis of steroids	2-C-methyl-D-erythritol 2,4-cyclodiphosphate synthase	4.6.1.12	H16_A1457	unknown	2pcdpmde -> mdecpp + cmp	2pcdpmde -> mdecpp + cmp	2pcdpmde -> mdecpp + cmp
ME4PCT	Biosynthesis of steroids	2-C-methyl-D-erythritol 4-phosphate cytidylyltransferase	2.7.7.60	H16_A1456	unknown	mde4p + ctp -> cdpmde + ppi	mde4p + ctp -> cdpmde + ppi	mde4p + ctp -> cdpmde + ppi
OCTPPS	Biosynthesis of steroids	Octaprenyl pyrophosphate synthase	2.5.1.29/2.5.1.33/2.5.1.30	multi step reaction	multi step reaction	frdp + 5 ipp -> opp + 5 ppi	frdp + 5 ipp -> opp + 5 ppi + 5 h	frdp + 5 ipp -> opp + 5 ppi + 5 h
FDFT1	Biosynthesis of steroids	farnesyl-diphosphate farnesyltransferase	2.5.1.21	H16_B0208	unknown	2 frdp -> ppi + pqppi	2 frdp -> ppi + pqppi + h	2 frdp -> ppi + pqppi + h
FDFT2	Biosynthesis of steroids	farnesyl-diphosphate farnesyltransferase	2.5.1.21	H16_B0208	unknown	pqppi + nadph + 2 h -> ppi + sql + nadp	pqppi + nadph -> ppi + sql + nadp	pqppi + nadph -> ppi + sql + nadp
THPT	Biosynthesis of steroids	trans-hexaprenyltranstransferase	2.5.1.30	H16_A3253	grcC	hppp + ipp -> heppp + ppi	hppp + ipp -> heppp + ppi + h	hppp + ipp -> heppp + ppi + h
AOXNS	Biotin Metabolism	8-amino-7-oxononanoate synthase	2.3.1.47	H16_A0181	bioF	ala + pmcoa <-> aona + co2 + coa	ala + pmcoa <-> aona + co2 + coa	ala + h + pmcoa <-> aona + co2 + coa
AMAOXNT	Biotin Metabolism	adenosylmethionine-8-amino-7-oxononanoate transaminase	2.6.1.62	H16_A0180/H16_B2123	bioA/unknown	aona + sam <-> samob + danna + h	aona + sam <-> samob + danna + h	aona + sam <-> samob + danna + h
BIOTS	Biotin Metabolism	Biotin synthase	2.8.1.6	H16_A0183	bioB	sam + dtb + s -> bt + da-5 + met + h	sam + dtb + s -> bt + da-5 + met + h	sam + dtb + s -> bt + da-5 + met
DTBTS	Biotin Metabolism	dethiobiotin synthase	6.3.3.3	H16_A0182	bioD	atp + co2 + danna -> adp + dtb + 2 h + pi	atp + co2 + danna -> adp + dtb + 3 h + pi	atp + co2 + danna -> adp + dtb + 2 h + pi
6CARHC	Biotin metabolism	6-carboxyhexanoate--CoA ligase	6.2.1.14	H16_B0928/H16_B1438	unknown/unknown	pimlt + atp + coa + 2 h -> amp + ppi + pmcoa	pimlt + atp + coa -> amp + ppi + pmcoa	pimlt + atp + coa -> amp + ppi + pmcoa + h
BTNACL	Biotin metabolism	biotin-[acetyl-CoA-carboxylase] ligase	6.3.4.15	H16_A0135/H16_A2946	bitA/unknown	atp + bt + h -> ppi + b5amp	atp + bt -> ppi + b5amp	atp + bt -> ppi + b5amp
BPHEDO1	Biphenyl degradation	biphenyl-2,3-diol 1,2-dioxygenase	1.13.11.39	H16_B0654	bphC	bp23d + o2 -> hophd + h	bp23d + o2 -> hophd + h	bp23d + o2 -> hophd + h
BIPHED1	Biphenyl degradation	2,6-Dioxo-6-phenylhexa-3-enoate benzoylhydrolase	3.7.1.8	H16_B0600	bphD	hophd + h2o -> benzot + op4en + h	hophd + h2o -> benzot + op4en + h	hophd + h2o -> benzot + op4en + h
BPHEDO2	Biphenyl degradation	biphenyl-2,3-diol 1,2-dioxygenase	1.13.11.39	H16_B0654	bphC	dhchph + o2 -> hochphhd + h	dhchph + o2 -> hochphhd + h	dhchph + o2 -> hochphhd + h
BIPHED2	Biphenyl degradation	2,6-Dioxo-6-phenylhexa-3-enoate benzoylhydrolase	3.7.1.8	H16_B0600	bphD	hochphhd + h2o -> 4chbenz + op4en + h	hochphhd + h2o -> 4chbenz + op4en + h	hochphhd + h2o -> 4chbenz + op4en + h
BPHEHB	Bisphenol A degradation	bisphenol A hydroxylase B	1.14.13.-	H16_A1145/H16_B0495/H16_B1480/H16_B2135	unknown/unknown/unknown/unknown	bisphenol + nadh + h + o2 -> 12bhpp + nad + h2o	bisphenol + nadh + h + o2 -> 12bhpp + nad + h2o	bisphenol + nadh + h + o2 -> 12bhpp + nad + h2o
BPHEHA	Bisphenol A degradation	bisphenol A hydroxylase A	1.14.13.-	H16_A1145/H16_B0495/H16_B1480/H16_B2135	unknown/unknown/unknown/unknown	bisphenol + nadh + h + o2 -> 22bhpp + nad + h2o	bisphenol + nadh + h + o2 -> 22bhpp + nad + h2o	bisphenol + nadh + h + o2 -> 22bhpp + nad + h2o
2BHPPPH	Bisphenol A degradation	2,2-bis(4-hydroxyphenyl)-1-propanol hydroxylase	1.14.13.-	H16_A1145/H16_B0495/H16_B1480/H16_B2135	unknown/unknown/unknown/unknown	22bhpp + nadh + h + o2 -> 23bhpp + nad + h2o	22bhpp + nadh + h + o2 -> 23bhpp + nad + h2o	22bhpp + nadh + h + o2 -> 23bhpp + nad + h2o
4ETHPMH	Bisphenol A degradation	4-ethylphenol methylenehydroxylase	1.14.13.-	H16_A1145/H16_B0495/H16_B1480/H16_B2135	unknown/unknown/unknown/unknown	4ethp + nadph + 2 h + o2 -> 14hpeth + nadp + h2o	4ethp + nadph + h + o2 -> 14hpeth + nadp + h2o	4ethp + nadph + h + o2 -> 14hpeth + nadp + h2o
14HPED	Bisphenol A degradation	1-(4'-hydroxyphenyl)ethanol dehydrogenase	1.1.1.-	H16_A0679/H16_A0893/H16_A1256/H16_A1828/H16_A2460/H16_A2586/H16_B0034/H16_B0572/H16_B0663/H16_B0831/H16_B1417/H16_B2561	unknown/unknown/unknown/unknown/abmB/unknown/wecC/unknown/unknown/unknown/unknown/unknown/	14hpeth + nad -> 4hactph + nadh + h	14hpeth + nad -> 4hactph + nadh + h	14hpeth + nad -> 4hactph + nadh + 2 h
MALDO	Butanoate metabolism	malate decarboxylating oxidoreductase (decarboxylating)	1.1.1.83	H16_A0477	leuB1	dmal + nad + 2 h -> co2 + nadh + pyr	dmal + nad + 3 h -> co2 + nadh + pyr	dmal + nad + 3 h -> co2 + nadh + pyr
ALHD4	Butanoate metabolism	aldehyde dehydrogenase (butanal, NAD)	1.1.1.-	H16_A0478/H16_A0679/H16_A0861/H16_A0893/H16_A1256/H16_A1562/H16_A1702/H16_A1828/H16_A2460/H16_A2474/H16_A2586/H16_B0034/H16_B0572/H16_B0663/H16_B0713/H16_B0831/H16_B0941/H16_B0942/H16_B1417/H16_B2168/H16_B2561/	ldhA/unknown/unknown/unknown/unknown/unknown/unknown/unknown/abmB/unknown/unknown/wecC/unknown/unknown/unknown/unknown/unknown/unknown/unknown/unknown/unknown/	butanal + h2o + nad -> 1boh + 2 h + nadh	butanal + h2o + nad -> 1boh + 2 h + nadh	butanal + h2o + nad -> 1boh + 2 h + nadh
MALEAI	Butanoate metabolism	maleate isomerase	5.2.1.1	H16_A0923/H16_B0811	unknown/unknown	malte <-> fum	malte <-> fum	malte <-> fum
ACALDb	Butanoate metabolism	acetaldehyde dehydrogenase	1.2.1.10	H16_A1806/H16_A2747/H16_B0551/H16_B0596	unknown/unknown/mhpF/unknown	butanal + coa + nad -> c040coa + nadh + h	butanal + coa + nad -> c040coa + nadh + h	butanal + coa + nad -> c040coa + nadh + h
4HBTD	Butanoate metabolism	4-hydroxybutyrate dehydrogenase	1.1.1.61	H16_A1553	gbd	4hbt + nad <-> sucsal + nadh + h	4hbt + nad <-> sucsal + nadh + h	4hbt + nad <-> sucsal + nadh + h
AC4HB	Butanoate metabolism	acetyl-CoA: 4-hydroxybutanoate CoA transferase (two-step reaction, unclear reaction)				4hbt + accoa <-> 3btecoa + ac + h2o	4hbt + accoa <-> 3btecoa + ac + h2o	4hbt + accoa <-> 3btecoa + ac + h2o
3HBCD	Butanoate metabolism	3-hydroxybutyryl-CoA dehydratase	4.2.1.55	H16_B1843/H16_B2534	unknown/unknown	r3hbcoa <-> ccoa + h2o	r3hbcoa <-> ccoa + h2o	r3hbcoa <-> ccoa + h2o
PHAS	Butanoate metabolism	polyhydroxyalkanoate synthase	2.3.1.-	H16_A1437/H16_A2003	phaC1/phaC2	r3hbcoa <-> pbhb + coa	r3hbcoa <-> pbhb + coa	r3hbcoa <-> pbhb + coa
PHBD	Butanoate metabolism	poly(3-hydroxybutyrate) depolymerase	3.1.1.75	H16_A1150/H16_A2862/H16_B0339/H16_B1014/H16_B2073/H16_B2401	phaZ1/phaZ2/phaZ3/phaZ5/phaZ6/phaZ7	pbhb + h2o <-> r3hbn2 + r3rhbb + h	pbhb + h2o <-> r3hbn2 + r3rhbb + h	pbhb + h2o <-> r3hbn2 + r3rhbb + h
HBDH	Butanoate metabolism	hydroxybutyrate-dimer hydrolase	3.1.1.22	H16_A2251	phaY1	r3rhbb + h2o <-> 2 r3hbn + h	r3rhbb + h2o <-> 2 r3hbn + h	r3rhbb + h2o <-> 2 r3hbn + h
3HBD	Butanoate metabolism	3-hydroxybutyrate dehydrogenase	1.1.1.30	H16_A1334/H16_A1814	unknown/unknown	r3hbn + nad <-> acac + nadh + h	r3hbn + nad <-> acac + nadh + h	r3hbn + nad <-> acac + nadh + h
ALHD5	Butanoate metabolism	aldehyde dehydrogenase (NAD+)	1.2.1.3	H16_A0232/H16_A0745/H16_A1114/H16_A1495/H16_B0212/H16_B0421/H16_B0737/H16_B0833/H16_B1534/H16_B1735/H16_B1751/H16_B1835/H16_B1960/H16_B2444	unknown/unknown/unknown/unknown/unknown/unknown/unknown/unknown/unknown/unknown/unknown/unknown/exaC/unknown/	3b1a + nad + h2o -> 3but + nadh + 2 h	3b1a + nad + h2o -> 3but + nadh + 2 h	3b1a + nad + h2o -> 3but + nadh + 2 h

AACOAR	Butanoate metabolism	acetoacetyl-CoA reductase	1.1.1.36	H16_A1439/H16_A2002/H16_A2171	phaB1/phaB2/phaB3	r3hbcoa + nadp <-> aacoa + nadph + 2 h	r3hbcoa + nadp <-> aacoa + nadph + h	r3hbcoa + nadp <-> aacoa + nadph + h
AACOAS	Butanoate metabolism	acetoacetyl-CoA synthetase	6.2.1.16	H16_A2860	unknown	atp + acac + coa + h -> amp + ppi + aacoa	atp + acac + coa -> amp + ppi + aacoa + h	atp + acac + coa -> amp + ppi + aacoa + h
3OACT	Butanoate metabolism	3-oxoacid CoA-transferase	2.8.3.5	H16_A1331/H16_A1332	unknown/unknown	succoa + acac <-> succ + aacoa	succoa + acac <-> succ + aacoa	succoa + acac <-> succ + aacoa
HXMGL	Butanoate metabolism	hydroxymethylglutaryl-CoA lyase	4.1.3.4	H16_A0186/H16_A1235/H16_A1547/H16_A2385/H16_B2494	unknown/mvaB/unknown/hmgL1/hmgL2	3h3mgcoa -> accoa + acac	3h3mgcoa -> accoa + acac	3h3mgcoa -> accoa + acac
ACDH1	Butanoate metabolism	acyl dehydratase	4.2.1.-	H16_A1069/H16_A1070/H16_A1289/H16_A2151/H16_A3307/H16_B0359/H16_B0706	unknown/unknown/unknown/unknown/unknown/unknown/unknown/	gl1coa + h2o <-> hgcoa	gl1coa + h2o <-> hgcoa	gl1coa + h2o <-> hgcoa
BTCAL	Butanoate metabolism	butyrate-CoA ligase/short-chain acyl-CoA synthetase	6.2.1.2			butn + atp + coa -> c040coa + amp +ppi	butn + atp + coa -> c040coa + amp +ppi	butn + atp + coa -> c040coa + amp +ppi
SUCCS	C5-Branched Dibasic acid metabolism	succinyl-CoA synthetase	6.2.1.5	H16_A0547/H16_A0548	sucC/sucD	atp + itcn + coa + h <-> adp + pi + itcncoa	atp + itcn + coa <-> adp + pi + itcncoa	atp + itcn + coa <-> adp + pi + itcncoa
CBBCYC	Calvin-Benson-Bassham cycle					3 co2 + 5 h2o + 9 atp + 6 nadph + 12 h -> 9 adp + 8 pi + g3p + 6 nadp	3 co2 + 5 h2o + 9 atp + 6 nadph -> 9 adp + 8 pi + g3p + 6 nadp + 3 h	3 co2 + 5 h2o + 9 atp + 6 nadph -> 9 adp + 8 pi + g3p + 6 nadp + 3 h
2AMBZC	Carbazole degradation	2-aminobenzoate-CoA ligase	6.2.1.32	H16_A2457	abmG	atp + an + coa + h -> amp + ppi + anthcoa	atp + an + coa -> amp + ppi + anthcoa + h	atp + an + coa -> amp + ppi + anthcoa + h
ANTHMN1	Carbazole degradation	anthraniloyl-CoA monooxygenase	1.14.13.40	H16_A2461	abmA	anthcoa + o2 + 2 nadh + 2 h -> 2amoeccoa + h2o + 2 nad	anthcoa + o2 + 2 nadh + 2 h -> 2amoeccoa + h2o + 2 nad	anthcoa + o2 + 2 nadh + 2 h -> 2amoeccoa + h2o + 2 nad
ANTHMN2	Carbazole degradation	anthraniloyl-CoA monooxygenase	1.14.13.40	H16_A2461	abmA	anthcoa + o2 + 2 nadph + 4 h -> 2amoeccoa + h2o + 2 nadp	anthcoa + o2 + 2 nadph + 2 h -> 2amoeccoa + h2o + 2 nadp	anthcoa + o2 + 2 nadph + 2 h -> 2amoeccoa + h2o + 2 nadp
FTOLAM	Carprolactam degradation	Hydrolase	3.5.1.-	H16_A1465/H16_A1546/H16_A1732/H16_A1734/H16_A2086/H16_A3386/H16_B0814/H16_B1172/H16_B1643/H16_B1666/H16_B2013/H16_B2014/H16_B2069/H16_B2126	unknown/unknown/unknown/unknown/unknown/unknown/unknown/unknown/unknown/unknown/unknown/unknown/unknown/unknown	cchfor + h2o -> cchlam + formate	cchfor + h2o -> cchlam + formate	cchfor + h2o -> cchlam + formate
CCHNM1	Carprolactam degradation	cyclohexanone monooxygenase	1.14.13.22	H16_B1746	unknown	cchexo + o2 + nadph + 2 h <-> 6hnld + nadp + h2o	cchexo + o2 + nadph + h <-> 6hnld + nadp + h2o	cchexo + o2 + nadph + h <-> 6hnld + nadp + h2o
GLTN3	Carprolactam degradation	gluconolactonase	3.1.1.17	H16_A3012/H16_B0345/H16_B1441	gnl1/gnl2/gnl3	6hnld + h2o -> 6hhn + h	6hnld + h2o -> 6hhn + h	6hnld + h2o -> 6hhn + h
ADIPL	Carprolactam degradation	adipate-CoA ligase	6.2.1.-	H16_A0866/H16_A0871/H16_A1230/H16_A1519/H16_A1700/H16_A1718/H16_A2252/H16_A2794/H16_A2807/H16_A2978/H16_B0174/H16_B0677/H16_B0910/H16_B1148/H16_B1264/H16_B1335/H16_B1662/H16_B1709/H16_B2522	unknown/unknown/unknown/unknown/unknown/unknown/unknown/unknown/unknown/unknown/unknown/unknown/unknown/unknown/unknown/unknown/unknown/unknown/unknown/	adip + coa + atp + h -> adipcoa + amp + ppi	adip + coa + atp -> adipcoa + amp + ppi + h	adip + coa + atp -> adipcoa + amp + ppi + h
ACOADG	Carprolactam degradation	acyl-CoA dehydrogenase	1.3.99.-	H16_B2555	unknown	adipcoa + fad -> carpcoa + fadh2	adipcoa + fad + h -> carpcoa + fadh2	adipcoa + fad + h -> carpcoa + fadh2
CCHNM2	Carprolactam degradation	cyclohexanone monooxygenase	1.14.13.22	H16_B1746	unknown	nadph + hccho + 2 h + o2 -> nadp + oohoch + h2o	nadph + hccho + h + o2 -> nadp + oohoch + h2o	nadph + hccho + h + o2 -> nadp + oohoch + h2o
MDH1	Citric Acid Cycle	malate dehydrogenase	1.1.1.37	H16_A2634/H16_B0334	mdh1/mdh2	mal + nad <-> h + nadh + oaa	mal + nad <-> h + nadh + oaa	mal + nad <-> h + nadh + oaa
ICITD	Citric Acid Cycle	isocitrate dehydrogenase (NAD)	1.1.1.41	H16_B1016	icd3	icit + nad <-> akg + co2 + nadh	icit + nad <-> akg + co2 + nadh	icit + nad <-> akg + co2 + nadh
ICITDp	Citric Acid Cycle	isocitrate dehydrogenase (NADP)	1.1.1.42	H16_A3056/H16_B1931	icd1/icd2	icit + nadp <-> akg + co2 + h + nadph	icit + nadp <-> akg + co2 + nadph	icit + nadp <-> akg + co2 + nadph
MDH2	Citric Acid Cycle	Malate dehydrogenase (ubiquinone 8 as acceptor)	1.1.99.16			mal + uq -> oaa + uqh2	mal + uq -> oaa + uqh2	mal + uq -> oaa + uqh2
CITL	Citric Acid Cycle	Citrate lyase	4.1.3.6	H16_A2635/H16_B0353/H16_B0680/H16_B2113	citE1/citE2/citE3/citE4	cit -> ac + oaa	cit -> ac + oaa	cit -> ac + oaa
FUMR	Citric Acid Cycle	fumarase	4.2.1.2	H16_A2528/H16_B0103	fumA/fumC	fum + h2o <-> mal	fum + h2o <-> mal	fum + h2o <-> mal
ACONT1	Citric Acid Cycle	aconitase (citrate hydro-lyase)	4.2.1.3	H16_A1907/H16_A2638/H16_B0568	acnM/acnA/acnB	cit <-> acon-C + h2o	cit <-> acon-C + h2o	cit <-> acon-C + h2o
ACONT2	Citric Acid Cycle	aconitase (isocitrate hydro-lyase)	4.2.1.3	H16_A1907/H16_A2638/H16_B0568	acnM/acnA/acnB	acon-C + h2o <-> icit	acon-C + h2o <-> icit	acon-C + h2o <-> icit
SUCOAS	Citric Acid Cycle	succinyl-CoA synthetase (ADP-forming)	6.2.1.5	H16_A0547&H16_A0548	sucC&sucD	atp + coa + succ + h <-> adp + pi + succoa	atp + coa + succ -> succoa + adp + pi	atp + coa + succ <-> adp + pi + succoa
AKGDH	Citric Acid Cycle	2-Oxogluterate dehydrogenase		(H16_A2325)(H16_A1377/H16_A2323/H16_A3724/H16_B1098)(H16_A2324)	(odhA)(pdhL/odhL/lpdaA/unknown)(odhB)	akg + coa + nad -> co2 + nadh + succoa	akg + coa + nad -> co2 + nadh + succoa	akg + coa + nad -> co2 + nadh + succoa
CITS	Citric Acid Cycle	citrate synthase	2.3.3.1	H16_A1229/H16_A2627/H16_B0357/H16_B0414/H16_B2211	unknown/cisY/unknown/unknown/unknown	accoa + h2o + oaa -> cit + coa + h	accoa + h2o + oaa -> cit + coa + h	accoa + h2o + oaa -> cit + coa + h
SUCCD3	Citric Acid Cycle	succinate dehydrogenase (irreversible)	1.3.99.1	H16_A2629&H16_A2630&H16_A2631&H16_A2632&H16_B0204	sdhB&sdhA&sdhD&sdhC&unknown	succ + fad -> fum + fadh2	succ + fad + h -> fum + fadh2	succ + fad + h -> fum + fadh2
NITL2	Cyanoamino acid metabolism	nitrilase	3.5.5.1	H16_A1125	nit	aamppn + 2 h2o -> ala + nh4	aamppn + 2 h2o + h -> ala + nh4	aamppn + 2 h2o + h -> ala + nh4
ACDH4	Cyanoamino acid metabolism	acyl dehydratase	4.2.1.-	H16_A1069/H16_A1070/H16_A1289/H16_A2151/H16_A3307/H16_B0359/H16_B0706	unknown/unknown/unknown/unknown/unknown/unknown/unknown/	hcyst + 2 cn + h2 -> aagcya + hcys + tcynt + h	hcyst + 2 cn + h2 -> aagcya + hcys + tcynt + h	hcyst + 2 cn + h2 -> aagcya + hcys + tcynt + h
NITL3	Cyanoamino acid metabolism	nitrilase	3.5.5.1	H16_A1125	nit	gagcya + 2 h2o -> glu + nh4	gagcya + 2 h2o + h -> glu + nh4	gagcya + 2 h2o + h -> glu + nh4
GGMT1	Cyanoamino acid metabolism	gamma-glutamyltranspeptidase	2.3.2.2	H16_A0784/H16_A1098/H16_A2780/H16_B0984	ggt2a/unknown/ggt2b/ggt2c	cyaala + glu + h -> ggbap + h2o + co2	cyaala + glu + h -> ggbap + h2o + co2	cyaala + glu + 2 h -> ggbap + h2o + co2
GGMT2	Cyanoamino acid metabolism	gamma-glutamyltranspeptidase	2.3.2.2	H16_A0784/H16_A1098/H16_A2780/H16_B0984	ggt2a/unknown/ggt2b/ggt2c	cyaala + glu -> ggbcya + h2o	cyaala + glu -> ggbcya + h2o	cyaala + glu + h -> ggbcya + h2o
SEROAT	Cysteine Metabolism	serine O-acetyltransferase	2.3.1.30	H16_A1216	cysE	accoa + ser <-> aser + coa	accoa + ser <-> aser + coa	accoa + ser <-> aser + coa
CYSSULD	Cysteine Metabolism	L-cysteine sulfinic acid desulfurase	4.1.1.12	H16_A3009	asdA	3slala + h -> ala + so2	3slala + h -> ala + so2	3slala + h -> ala + so2
CYSST1	Cysteine Metabolism	cysteine synthase	2.5.1.47	H16_A0807/H16_A1903/H16_B2378	cysK1/cysK2/cysK4	aser + h2s -> ac + cys + h	aser + h2s -> ac + cys	aser + h2s -> ac + cys
CYSTBL2	Cysteine metabolism	cystathionine beta-lyase	4.4.1.8	H16_A1447	metC	cyst + h2o -> pyr + nh4 + tcys	cyst + h2o -> pyr + nh4 + tcys	cyst + h2o -> pyr + nh4 + tcys
CYTTS6	Cysteine metabolism	cystathionine gamma-synthase	2.5.1.48	H16_A2606	metB	aser + tsul -> sslcys + ac + h	aser + tsul -> sslcys + ac + h	aser + tsul -> sslcys + ac + h
CYSST2	Cysteine metabolism	cysteine synthase	2.5.1.47	H16_A0807/H16_A1903/H16_B2378	cysK1/cysK2/cysK4	aser + tsul -> sslcys + ac + h	aser + tsul -> sslcys + ac + h	aser + tsul -> sslcys + ac + h
SERDHT2	Cysteine metabolism	L-serine dehydratase	4.3.1.17	H16_A3622	sdaA	ser -> 2aa + h2o	ser -> 2aa + h2o	ser -> 2aa + h2o
OAHSL2	Cysteine metabolism	O-acetylhomoserine (thiol)-lyase	2.5.1.49	H16_A1313/H16_B2229	metY1/metY2	aser + tsul + rthio -> cys + so3 + othio + ac + h	aser + tsul + rthio -> cys + so3 + othio + ac + h	aser + tsul + rthio -> cys + so3 + othio + ac + h
CYSST3	Cysteine metabolism	cysteine synthase	2.5.1.47	H16_A0807/H16_A1903/H16_B2378	cysK1/cysK2/cysK4	aser + tsul + rthio -> cys + so3 + othio + ac + h	aser + tsul + rthio -> cys + so3 + othio + ac + h	aser + tsul + rthio -> cys + so3 + othio + ac + h
ASPAM1	Cysteine metabolism	aspartate aminotransferase	2.6.1.1	H16_A2857	unknown	mpyr + glu -> cys + akg	mpyr + glu -> cys + akg	mpyr + glu -> cys + akg
ASPAM2	Cysteine metabolism	aspartate aminotransferase	2.6.1.1	H16_A2857	unknown	3slala + akg + h -> 3sfpyr + glu	3slala + akg + h -> 3sfpyr + glu	3slala + akg -> 3sfpyr + glu
ASPAM3	Cysteine metabolism	aspartate aminotransferase	2.6.1.1	H16_A2857	unknown	cysteate + akg -> 3spyr + glu	cysteate + akg -> 3spyr + glu	cysteate + akg -> 3spyr + glu
L-LACD5	Cysteine metabolism	L-lactate dehydrogenase	1.1.1.27	H16_A0666	ldh	mpyr + nadh + h -> 3mlac + nad	mpyr + nadh + h -> 3mlac + nad	mpyr + nadh + h -> 3mlac + nad

SADT2	Cysteine Metabolism	Sulfate adenyltransferase				atp + selnt <-> ppi + aselnt	atp + selnt <-> ppi + aselnt + h	atp + selnt <-> ppi + aselnt + h
ALAALAL	D-alanine metabolism	D-alanine-D-alanine ligase	6.3.2.4	H16_A3271	ddlB	2 dala + atp <-> adp + alaala + pi	2 dala + atp <-> adp + alaala + h + pi	2 dala + atp <-> adp + alaala + 2 h + pi
DATA4	D-alanine metabolism	D-alanine transaminase	2.6.1.21	H16_A2521	dat	dala + akg <-> pyr + dglu	dala + akg <-> pyr + dglu	dala + akg <-> pyr + dglu
GLUN2	D-glutamine and D-glutamate metabolism	glutaminase	3.5.1.38	H16_A1910/H16_A2280	ansA/unknown	dgln + h2o -> dglu + nh4	dgln + h2o -> dglu + nh4	dgln + h2o -> dglu + nh4
BZACCOAT1	Ethylbenzene degradation	benzoyl acetyl-CoA thiolase	2.3.1.16	H16_A0462/H16_A1290/H16_B0200	unknown/unknown/pcaF	bzaccoa + coa -> bzocoa + accoa	bzaccoa + coa -> bzocoa + accoa	bzaccoa + coa -> bzocoa + accoa
BZACCOAT2	Ethylbenzene degradation	benzoyl acetyl-CoA thiolase	2.3.1.-	H16_A0039/H16_A0240/H16_A0269/H16_A0699/H16_A1315/H16_A1564/H16_A1683/H16_A1802/H16_A2759/H16_A3071/H16_A3093/H16_A3221/H16_A3529/H16_A3586/H16_B0018/H16_B0021/H16_B0032/H16_B0219/H16_B1278/H16_B1292/H16_B1407/H16_B1663/H16_B1899/H16_B2397/	unknown/unknown/unknown/unknown/unknown/unknown/unknown/unknown/unknown/unknown/unknown/unknown/unknown/pat/unknown/unknown/wbpD/unknown/unknown/phnT/unknown/unknown/unknown/unknown/	bzaccoa + coa -> bzocoa + accoa	bzaccoa + coa -> bzocoa + accoa	bzaccoa + coa -> bzocoa + accoa
FABC120	Fatty acid biosynthesis	Fatty acid biosynthesis (dodecanoic acid; c12:0)	2.3.1.41 AND 2.3.1.179 AND 2.3.1.180 AND 1.1.1.100			acACP + 5 malACP + 10 nadph + 25 h -> 10 nadp + c120ACP + 5 co2 + 5 ACP + 5 h2o	acACP + 5 malACP + 10 nadph + 15 h -> 10 nadp + c120ACP + 5 co2 + 5 ACP + 5 h2o	acACP + 5 malACP + 10 nadph + 15 h -> 10 nadp + c120ACP + 5 co2 + 5 ACP + 5 h2o
FABC140	Fatty acid biosynthesis	Fatty acid biosynthesis (tetradecanoic acid; c14:0)	2.3.1.41 AND 2.3.1.179 AND 2.3.1.180 AND 1.1.1.100			acACP + 6 malACP + 12 nadph + 30 h -> 12 nadp + c140ACP + 6 co2 + 6 ACP + 6 h2o	acACP + 6 malACP + 12 nadph + 18 h -> 12 nadp + c140ACP + 6 co2 + 6 ACP + 6 h2o	acACP + 6 malACP + 12 nadph + 18 h -> 12 nadp + c140ACP + 6 co2 + 6 ACP + 6 h2o
FABC141	Fatty acid biosynthesis	Fatty acid biosynthesis (tetradecanoic acid; c14:1)	2.3.1.41 AND 2.3.1.179 AND 2.3.1.180 AND 1.1.1.100			acACP + 6 malACP + 11 nadph + 28 h -> 11 nadp + c141ACP + 6 co2 + 6 ACP + 6 h2o	acACP + 6 malACP + 11 nadph + 17 h -> 11 nadp + c141ACP + 6 co2 + 6 ACP + 6 h2o	acACP + 6 malACP + 11 nadph + 17 h -> 11 nadp + c141ACP + 6 co2 + 6 ACP + 6 h2o
FABC150	Fatty acid biosynthesis	Fatty acid biosynthesis (pentadecanoic acid; c15:0)	2.3.1.41 AND 2.3.1.179 AND 2.3.1.180 AND 1.1.1.100			ppacp + 6 malACP + 12 nadph + 30 h -> 12 nadp + c150ACP + 6 co2 + 6 ACP + 6 h2o	ppacp + 6 malACP + 12 nadph + 18 h -> 12 nadp + 6 co2 + 6 ACP + 6 h2o + c150ACP	ppacp + 6 malACP + 12 nadph + 18 h -> 12 nadp + c150ACP + 6 co2 + 6 ACP + 6 h2o
FABC151	Fatty acid biosynthesis	Fatty acid biosynthesis (pentadecanoic acid; c15:1)	2.3.1.41 AND 2.3.1.179 AND 2.3.1.180 AND 1.1.1.100			ppacp + 6 malACP + 11 nadph + 28 h -> 11 nadp + c151ACP + 6 co2 + 6 ACP + 6 h2o	ppacp + 6 malACP + 11 nadph + 17 h -> 11 nadp + 6 co2 + 6 ACP + 6 h2o + c151ACP	ppacp + 6 malACP + 11 nadph + 17 h -> 11 nadp + c151ACP + 6 co2 + 6 ACP + 6 h2o
FABC160	Fatty acid biosynthesis	Fatty acid biosynthesis (hexadecanoic acid; c16:0)	2.3.1.41 AND 2.3.1.179 AND 2.3.1.180 AND 1.1.1.100			acACP + 7 malACP + 14 nadph + 36 h -> 14 nadp + c160ACP + 7 co2 + 7 ACP + 7 h2o	acACP + 7 malACP + 14 nadph + 22 h -> 14 nadp + c160ACP + 7 co2 + 7 ACP + 7 h2o	acACP + 7 malACP + 14 nadph + 22 h -> 14 nadp + c160ACP + 7 co2 + 7 ACP + 7 h2o
FABC161	Fatty acid biosynthesis	Fatty acid biosynthesis (hexadecanoic acid; c16:1)	2.3.1.41 AND 2.3.1.179 AND 2.3.1.180 AND 1.1.1.100			acACP + 7 malACP + 13 nadph + 34 h -> 13 nadp + c161ACP + 7 co2 + 7 ACP + 7 h2o	acACP + 7 malACP + 13 nadph + 21 h -> 13 nadp + c161ACP + 7 co2 + 7 ACP + 7 h2o	acACP + 7 malACP + 13 nadph + 21 h -> 13 nadp + c161ACP + 7 co2 + 7 ACP + 7 h2o
FABC170	Fatty acid biosynthesis	Fatty acid biosynthesis (heptadecanoic acid; c17:0)	2.3.1.41 AND 2.3.1.179 AND 2.3.1.180 AND 1.1.1.100			ppacp + 7 malACP + 14 nadph + 35 h -> 14 nadp + c170ACP + 7 co2 + 7 ACP + 7 h2o	ppacp + 7 malACP + 14 nadph + 21 h -> 14 nadp + 7 co2 + 7 ACP + 7 h2o + c170ACP	ppacp + 7 malACP + 14 nadph + 21 h -> 14 nadp + c170ACP + 7 co2 + 7 ACP + 7 h2o
FABC171	Fatty acid biosynthesis	Fatty acid biosynthesis (heptadecanoic acid; c17:1)	2.3.1.41 AND 2.3.1.179 AND 2.3.1.180 AND 1.1.1.100			ppacp + 7 malACP + 13 nadph + 33 h -> 13 nadp + c171ACP + 7 co2 + 7 ACP + 7 h2o	ppacp + 7 malACP + 13 nadph + 20 h -> 13 nadp + 7 co2 + 7 ACP + 7 h2o + c171ACP	ppacp + 7 malACP + 13 nadph + 20 h -> 13 nadp + c171ACP + 7 co2 + 7 ACP + 7 h2o
FABC180	Fatty acid biosynthesis	Fatty acid biosynthesis (octadecanoic acid; c18:0)	2.3.1.41 AND 2.3.1.179 AND 2.3.1.180 AND 1.1.1.100			acACP + 8 malACP + 16 nadph + 41 h -> 16 nadp + c180ACP + 8 co2 + 8 ACP + 8 h2o	acACP + 8 malACP + 16 nadph + 25 h -> 16 nadp + c180ACP + 8 co2 + 8 ACP + 8 h2o	acACP + 8 malACP + 16 nadph + 25 h -> 16 nadp + c180ACP + 8 co2 + 8 ACP + 8 h2o
FABC181	Fatty acid biosynthesis	Fatty acid biosynthesis (octadecanoic acid; c18:1)	2.3.1.41 AND 2.3.1.179 AND 2.3.1.180 AND 1.1.1.100			acACP + 8 malACP + 15 nadph + 39 h -> 15 nadp + c181ACP + 8 co2 + 8 ACP + 8 h2o	acACP + 8 malACP + 15 nadph + 24 h -> 15 nadp + c181ACP + 8 co2 + 8 ACP + 8 h2o	acACP + 8 malACP + 15 nadph + 24 h -> 15 nadp + c181ACP + 8 co2 + 8 ACP + 8 h2o
FABC190	Fatty acid biosynthesis	Fatty acid biosynthesis (nonadecanoic acid; c19:0)	2.3.1.41 AND 2.3.1.179 AND 2.3.1.180 AND 1.1.1.100			ppacp + 8 malACP + 16 nadph + 40 h -> 16 nadp + c190ACP + 8 co2 + 8 ACP + 8 h2o	ppacp + 8 malACP + 16 nadph + 24 h -> 16 nadp + 8 co2 + 8 ACP + 8 h2o + c190ACP	ppacp + 8 malACP + 16 nadph + 24 h -> 16 nadp + c190ACP + 8 co2 + 8 ACP + 8 h2o
ACCOAT	Fatty acid biosynthesis	Acetyl-CoA ACP transacylase	2.3.1.179/2.3.1.180/2.3.1.86/2.3.1.41			ACP + accoa <-> acACP + coa	ACP + accoa <-> acACP + coa	ACP + accoa <-> acACP + coa
PPCOAT	Fatty acid biosynthesis	Propionyl-CoA ACP transacylase	2.3.1.179/2.3.1.180/2.3.1.86/2.3.1.41			ACP + ppcoa <-> ppacp + coa	ACP + ppcoa <-> coa + ppacp	ACP + ppcoa <-> ppacp + coa
MALCOAT	Fatty acid biosynthesis	Malonyl-CoA-ACP transacylase	2.3.1.39/2.3.1.86	(H16_A2568)/(H16_A1971)	(fabD)/(unknown)	ACP + malcoa <-> coa + malACP	ACP + malcoa <-> coa + malACP	ACP + malcoa <-> coa + malACP
BKACPS1	Fatty acid biosynthesis	beta-ketoacyl-ACP synthase	2.3.1.41/2.3.1.179/2.3.1.86/2.3.1.41			acACP + h + malACP -> ACP + actACP + co2	acACP + h + malACP -> ACP + actACP + co2	acACP + h + malACP -> ACP + actACP + co2
ACCOACB	Fatty acid biosynthesis	acetyl-CoA carboxylase	6.4.1.2	H16_A1223&H16_A2611	accA1&accD	accoa + atp + hco3 -> adp + h + malcoa + pi	accoa + atp + hco3 -> adp + h + malcoa + pi	accoa + atp + hco3 -> adp + h + malcoa + pi

BITCB	Fatty acid biosynthesis	biotin carboxylase	6.3.4.14	H16_A0184/H16_A3172/H16_A3290/H16_B1757	accC1/accC2/accC3/unknown	accoa + atp + hco3 -> adp + h + malcoa + pi	accoa + atp + hco3 -> adp + h + malcoa + pi	accoa + atp + hco3 -> adp + h + malcoa + pi
BKACPS2	Fatty acid biosynthesis	beta-ketoacyl-ACP synthase	2.3.1.41/2.3.1.179/2.3.1.86/2.3.1.41			accoa + h + malACP -> actACP + co2 + coa	accoa + h + malACP -> actACP + co2 + coa	accoa + h + malACP -> actACP + co2 + coa
FAMC120	Fatty acid metabolism	Fatty acid metabolism (dodecanoic acid; c12:0)	6.2.1.3 AND 1.3.99.3AND 4.2.1.17AND 1.1.1.35AND			c120 + 6 coa + 5 fad + 5 nad + atp + 5 h2o -> 6 accoa + 5 fadh2 + 5 nadh + amp + ppi + 4 h	c120 + 6 coa + 5 fad + 5 nad + atp -> 6 accoa + 5 fadh2 + 5 nadh + amp + ppi + h	c120 + 6 coa + 5 fad + 5 nad + atp + 5 h2o -> 6 accoa + 5 fadh2 + 5 nadh + amp + ppi + h
FAMC140	Fatty acid metabolism	Fatty acid metabolism (tetradecanoic acid; c14:0)	6.2.1.3 AND 1.3.99.3AND 4.2.1.17AND 1.1.1.35AND			c140 + 7 coa + 6 fad + 6 nad + atp + 6 h2o -> 7 accoa + 6 fadh2 + 6 nadh + amp + ppi + 5 h	c140 + 7 coa + 6 fad + 6 nad + atp -> 7 accoa + 6 fadh2 + 6 nadh + amp + ppi + h	c140 + 7 coa + 6 fad + 6 nad + atp + 6 h2o -> 7 accoa + 6 fadh2 + 6 nadh + amp + ppi + h
FAMC141	Fatty acid metabolism	Fatty acid metabolism (tetradecanoic acid; c14:1)	6.2.1.3 AND 1.3.99.3AND 4.2.1.17AND 1.1.1.35AND			ttdcea + 7 coa + 6 fad + 6 nad + atp + 6 h2o -> 7 accoa + 6 fadh2 + 6 nadh + amp + ppi + 5 h	c141 + 7 coa + 6 fad + 6 nad + atp -> 7 accoa + 6 fadh2 + 6 nadh + amp + ppi + h	ttdcea + 7 coa + 6 fad + 6 nad + atp + 6 h2o -> 7 accoa + 6 fadh2 + 6 nadh + amp + ppi + h
FAMC150	Fatty acid metabolism	Fatty acid metabolism (pentadecanoic acid; c15:0)	6.2.1.3 AND 1.3.99.3AND 4.2.1.17AND 1.1.1.35AND			c150 + 7 coa + 6 fad + 6 nad + atp + 6 h2o -> 6 accoa + ppcoa + 6 fadh2 + 6 nadh + amp + ppi + 5 h	c150 + 7 coa + 6 fad + 6 nad + atp -> 6 accoa + ppcoa + 6 fadh2 + 6 nadh + amp + ppi + h	c150 + 7 coa + 6 fad + 6 nad + atp + 6 h2o -> 6 accoa + ppcoa + 6 fadh2 + 6 nadh + amp + ppi + h
FAMC151	Fatty acid metabolism	Fatty acid metabolism (pentadecanoic acid; c15:1)	6.2.1.3 AND 1.3.99.3AND 4.2.1.17AND 1.1.1.35AND			c151 + 7 coa + 6 fad + 6 nad + atp + 6 h2o -> 6 accoa + ppcoa + 6 fadh2 + 6 nadh + amp + ppi + 5 h	c151 + 7 coa + 6 fad + 6 nad + atp -> 6 accoa + ppcoa + 6 fadh2 + 6 nadh + amp + ppi + h	c151 + 7 coa + 6 fad + 6 nad + atp + 6 h2o -> 6 accoa + ppcoa + 6 fadh2 + 6 nadh + amp + ppi + h
FAMC160	Fatty acid metabolism	Fatty acid metabolism (hexadecanoic acid; c16:0)	6.2.1.3 AND 1.3.99.3AND 4.2.1.17AND 1.1.1.35AND			c160 + 8 coa + 7 fad + 7 nad + atp + 7 h2o -> 8 accoa + 7 fadh2 + 7 nadh + amp + ppi + 6 h	c160 + 8 coa + 7 fad + 7 nad + atp -> 8 accoa + 7 fadh2 + 7 nadh + amp + ppi + h	c160 + 8 coa + 7 fad + 7 nad + atp + 7 h2o -> 8 accoa + 7 fadh2 + 7 nadh + amp + ppi + h
FAMC161	Fatty acid metabolism	Fatty acid metabolism (hexadecanoic acid; c16:1)	6.2.1.3 AND 1.3.99.3AND 4.2.1.17AND 1.1.1.35AND			c161 + 8 coa + 7 fad + 7 nad + atp + 7 h2o -> 8 accoa + 7 fadh2 + 7 nadh + amp + ppi + 6 h	c161 + 8 coa + 7 fad + 7 nad + atp -> 8 accoa + 7 fadh2 + 7 nadh + amp + ppi + h	c161 + 8 coa + 7 fad + 7 nad + atp + 7 h2o -> 8 accoa + 7 fadh2 + 7 nadh + amp + ppi + h
FAMC170	Fatty acid metabolism	Fatty acid metabolism (heptadecanoic acid; c17:0)	6.2.1.3 AND 1.3.99.3AND 4.2.1.17AND 1.1.1.35AND			c170 + 8 coa + 7 fad + 7 nad + atp + 7 h2o -> 7 accoa + ppcoa + 7 fadh2 + 7 nadh + amp + ppi + 6 h	c170 + 8 coa + 7 fad + 7 nad + atp -> 7 accoa + ppcoa + 7 fadh2 + 7 nadh + amp + ppi + h	c170 + 8 coa + 7 fad + 7 nad + atp + 7 h2o -> 7 accoa + ppcoa + 7 fadh2 + 7 nadh + amp + ppi + h
FAMC171	Fatty acid metabolism	Fatty acid metabolism (heptadecanoic acid; c17:1)	6.2.1.3 AND 1.3.99.3AND 4.2.1.17AND 1.1.1.35AND			c171 + 8 coa + 7 fad + 7 nad + atp + 7 h2o -> 7 accoa + ppcoa + 7 fadh2 + 7 nadh + amp + ppi + 6 h	c171 + 8 coa + 7 fad + 7 nad + atp -> 7 accoa + ppcoa + 7 fadh2 + 7 nadh + amp + ppi + h	c171 + 8 coa + 7 fad + 7 nad + atp + 7 h2o -> 7 accoa + ppcoa + 7 fadh2 + 7 nadh + amp + ppi + h
FAMC180	Fatty acid metabolism	Fatty acid metabolism (octadecanoic acid; c18:0)	6.2.1.3 AND 1.3.99.3AND 4.2.1.17AND 1.1.1.35AND			c180 + 9 coa + 8 fad + 8 nad + atp + 8 h2o -> 9 accoa + 8 fadh2 + 8 nadh + amp + ppi + 7 h	c180 + 9 coa + 8 fad + 8 nad + atp -> 9 accoa + 8 fadh2 + 8 nadh + amp + ppi + h	c180 + 9 coa + 8 fad + 8 nad + atp + 8 h2o -> 9 accoa + 8 fadh2 + 8 nadh + amp + ppi + h
FAMC181	Fatty acid metabolism	Fatty acid metabolism (octadecanoic acid; c18:1)	6.2.1.3 AND 1.3.99.3AND 4.2.1.17AND 1.1.1.35AND			c181 + 9 coa + 8 fad + 8 nad + atp + 8 h2o -> 9 accoa + 8 fadh2 + 8 nadh + amp + ppi + 7 h	c181 + 9 coa + 8 fad + 8 nad + atp -> 9 accoa + 8 fadh2 + 8 nadh + amp + ppi + h	c181 + 9 coa + 8 fad + 8 nad + atp + 8 h2o -> 9 accoa + 8 fadh2 + 8 nadh + amp + ppi + h
FAMC190	Fatty acid metabolism	Fatty acid metabolism (nonadecanoic acid; c19:0)	6.2.1.3 AND 1.3.99.3AND 4.2.1.17AND 1.1.1.35AND			c190 + 9 coa + 8 fad + 8 nad + atp + 8 h2o -> 8 accoa + ppcoa + 8 fadh2 + 8 nadh + amp + ppi + 7 h	c190 + 9 coa + 8 fad + 8 nad + atp -> 8 accoa + ppcoa + 8 fadh2 + 8 nadh + amp + ppi + h	c190 + 9 coa + 8 fad + 8 nad + atp + 8 h2o -> 8 accoa + ppcoa + 8 fadh2 + 8 nadh + amp + ppi + h
HACOAD1	Fatty acid metabolism	3-hydroxyacyl-CoA dehydrogenase (acetoacetyl-CoA)	1.1.1.35	H16_A0282/H16_A0602/H16_A1102/H16_A1888/H16_B0388/H16_B0724/H16_B1652	paaH1/unknown/paaH2/unknown/unknown/unknown/unknown	3hbcoa + nad -> aacoa + h + nadh	3hbcoa + nad -> aacoa + h + nadh	3hbcoa + nad -> aacoa + h + nadh
ACOADH1	Fatty acid metabolism	acyl-CoA dehydrogenase (butanoyl-CoA)	1.3.99.2	H16_A0172/H16_B0485/H16_B0752/H16_B0850/H16_B1371	unknown/unknown/unknown/unknown/unknown/unknown	c040coa + fad <-> ccoa + fadh2	c040coa + fad + h <-> ccoa + fadh2	c040coa + fad + h <-> ccoa + fadh2
ACCOAAT1	Fatty acid metabolism	acetyl-CoA C-acetyltransferase	2.3.1.9	H16_A0170/H16_A0867/H16_A0868/H16_A0872/H16_A1297/H16_A1438/H16_A1445/H16_A1528/H16_A1713/H16_A1720/H16_A1887/H16_A2148/H16_B0380/H16_B0381/H16_B0406/H16_B0662/H16_B0668/H16_B0759/H16_B1369/H16_B1771	unknown/unknown/unknown/unknown/unknown/phaA/bktB/unknown/unknown/unknown/unknown/unknown/unknown/unknown/unknown/unknown/unknown/unknown/unknown/unknown	2 accoa <-> aacoa + coa	2 accoa <-> aacoa + coa	2 accoa <-> aacoa + coa

ENCOAH1	Fatty acid metabolism	enoyl-CoA hydratase	4.2.1.17	H16_A0100/H16_A0142/H16_A0179/H16_A0461/H16_A0464/H16_A0810/H16_A0865/H16_A0873/H16_A1101/H16_A1410/H16_A1699/H16_A1716/H16_A1719/H16_A1832/H16_A1885/H16_A1889/H16_A2138/H16_A2258/H16_A2979/H16_A3201/H16_A3311/H16_A3593/H16_A3594/H16_B0365/H16_B0382/H16_B0389/H16_B0402/H16_B0419/H16_B0420/H16_B0657/H16_B0659/H16_B0698/H16_B0724/H16_B0756/H16_B0848/H16_B0915/H16_B1188/H16_B1346/H16_B1439/H16_B1738/H16_B1741/H16_B1742/H16_B1773/H16_B1905/H16_B1914/H16_B2156/H16_B2478/	unknown/	3hbcoa <-> ccoa + h2o	3hbcoa <-> ccoa + h2o	3hbcoa <-> ccoa + h2o
34DHDHF	Fluorene degradation	3,4-dihydroxy-3,4-dihydrofluorene dehydrogenase	1.3.1.-	H16_B0731/H16_B0734	unknown/unknown	c34dhdhf -> 34dhflu + h2	c34dhdhf -> 34dhflu + h2	c34dhdhf -> 34dhflu + h2
34DHFD	Fluorene degradation	3,4-dihydroxyfluorene 4,4a-dioxygenase	1.13.11.-	H16_A0633/H16_B0223/H16_B0757/H16_B1109/H16_B1420/H16_B1836	pcaH2/unknown/unknown/unknown/unknown/unknown	34dhflu + o2 -> hodhiybe + h	34dhflu + o2 -> hodhiybe + h	34dhflu + o2 -> hodhiybe + h
12DHFD	Fluorene degradation	1,2-dihydroxyfluorene 1,1a-dioxygenase	1.13.11.-	H16_A0633/H16_B0223/H16_B0757/H16_B1109/H16_B1420/H16_B1836	pcaH2/unknown/unknown/unknown/unknown/unknown	12dhflu + o2 -> hodhiyb2e + h	12dhflu + o2 -> hodhiyb2e + h	12dhflu + o2 -> hodhiyb2e + h
2HOHPHDB	Fluorene degradation	2-Hydroxy-6-oxo-6-(2-hydroxyphenyl)-hexa-2,4-dienoate benzoylhydrolase	3.7.1.8	H16_B0600	bphD	2hohphd + h2o -> salcyl + op4en + h	2hohphd + h2o -> salcyl + op4en + h	2hohphd + h2o -> salcyl + op4en + h
2HOHPHODB	Fluorene degradation	2-Hydroxy-6-oxo-6-(2-hydroxyphenoxy)-hexa-2,4-dienoate benzoylhydrolase	3.7.1.8	H16_B0600	bphD	2hohphod + h2o -> 2hmuc + catech + h	2hohphod + h2o -> 2hmuc + catech + h	2hohphod + h2o -> 2hmuc + catech + h
BZOTD3	Fluorobenzoate degradation	benzoate 1,2-dioxygenase	1.14.12.10	H16_A1961&H16_A1962&H16_A1963	benC&benB&benA	2flrbz + nadh + h + o2 -> 2fchdc + nad	2flrbz + nadh + h + o2 -> 2fchdc + nad	2flrbz + nadh + h + o2 -> 2fchdc + nad
BZOTD4	Fluorobenzoate degradation	benzoate 1,2-dioxygenase	1.14.12.10	H16_A1961&H16_A1962&H16_A1963	benC&benB&benA	2flrbz + nadh + h + o2 -> 6fchdc + nad	2flrbz + nadh + h + o2 -> 6fchdc + nad	2flrbz + nadh + h + o2 -> 6fchdc + nad
BZOTD5	Fluorobenzoate degradation	benzoate 1,2-dioxygenase	1.14.12.10	H16_A1961&H16_A1962&H16_A1963	benC&benB&benA	3flrbz + nadh + o2 + h -> 3fchdc + nad	3flrbz + nadh + o2 + h -> 3fchdc + nad	3flrbz + nadh + o2 + h -> 3fchdc + nad
BZOTD6	Fluorobenzoate degradation	benzoate 1,2-dioxygenase	1.14.12.10	H16_A1961&H16_A1962&H16_A1963	benC&benB&benA	3flrbz + nadh + o2 + h -> 5fchdc + nad	3flrbz + nadh + o2 + h -> 5fchdc + nad	3flrbz + nadh + o2 + h -> 5fchdc + nad
BZOTD7	Fluorobenzoate degradation	benzoate 1,2-dioxygenase	1.14.12.10	H16_A1961&H16_A1962&H16_A1963	benC&benB&benA	4flrbz + nadh + h + o2 -> 4fchdc + nad	4flrbz + nadh + h + o2 -> 4fchdc + nad	4flrbz + nadh + h + o2 -> 4fchdc + nad
16DCDC2	Fluorobenzoate degradation	1,6-dihydroxycyclohexa-2,4-diene-1-carboxylate dehydrogenase	1.3.1.25	H16_A1960	benD	3fchdc + nad -> 3flrcatech + nadh + co2	3fchdc + nad -> 3flrcatech + nadh + co2	3fchdc + nad -> 3flrcatech + nadh + co2
16DCDC3	Fluorobenzoate degradation	1,6-dihydroxycyclohexa-2,4-diene-1-carboxylate dehydrogenase	1.3.1.25	H16_A1960	benD	5fchdc + nad -> 4flrcatech + nadh + co2	5fchdc + nad -> 4flrcatech + nadh + co2	5fchdc + nad -> 4flrcatech + nadh + co2
16DCDC4	Fluorobenzoate degradation	1,6-dihydroxycyclohexa-2,4-diene-1-carboxylate dehydrogenase	1.3.1.25	H16_A1960	benD	4fchdc + nad -> 4flrcatech + nadh + co2	4fchdc + nad -> 4flrcatech + nadh + co2	4fchdc + nad -> 4flrcatech + nadh + co2
CATCHDG6	Fluorobenzoate degradation	catechol 1,2-dioxygenase	1.13.11.1	H16_A1964/H16_B0968	catA/pcpA	3flrcatech + o2 -> 2flcmuc + 2 h	3flrcatech + o2 -> 2flcmuc + 2 h	3flrcatech + o2 -> 2flcmuc + 2 h
CATCHDG7	Fluorobenzoate degradation	catechol 1,2-dioxygenase	1.13.11.1	H16_A1964/H16_B0968	catA/pcpA	4flrcatech + o2 -> 3flcmuc + 2 h	4flrcatech + o2 -> 3flcmuc + 2 h	4flrcatech + o2 -> 3flcmuc + 2 h
FLBZDD	Fluorobenzoate degradation	fluorobenzene dihydrodiol dehydrogenase	1.3.1.-	H16_B0731/H16_B0734	unknown/unknown	4fchcd + nad -> 4flrcatech + nadh + h	4fchcd + nad -> 4flrcatech + nadh + h	4fchcd + nad -> 4flrcatech + nadh + h
MCCIS5	Fluorobenzoate degradation	muconate cycloisomerase	5.5.1.1	H16_A1966/H16_B0536	catB3/catB4	3flcmuc + h -> 4fmuclac	3flcmuc + h -> 4fmuclac	3flcmuc + h -> 4fmuclac
CMBLD6	Fluorobenzoate degradation	carboxymethylenebutenolidase	3.1.1.45	H16_A2215/H16_A2739/H16_A3488	unknown/unknown/unknown	4fmuclac + h2o -> 2mac + hf + 2 h	4fmuclac + h2o -> 2mac + hf + 2 h	4fmuclac + h2o -> 2mac + hf + 2 h
CMBLD7	Fluorobenzoate degradation	carboxymethylenebutenolidase	3.1.1.45	H16_A2215/H16_A2739/H16_A3488	unknown/unknown/unknown	5fmuclac + h2o -> 2mac + hf + 2 h	5fmuclac + h2o -> 2mac + hf + 2 h	5fmuclac + h2o -> 2mac + hf + 2 h
HACDH	Fluorobenzoate degradation	haloacetate dehalogenase	3.8.1.3			hf + glycolate + h <-> flac + h2o	hf + glycolate + h <-> flac + h2o	hf + glycolate + h <-> flac + h2o
FLALDO	Fluorobenzoate degradation	fluoroacetaldehyde:NAD+ oxidoreductase	1.2.1.69			flac + nadh + 2 h <-> flald + nad + h2o	flac + nadh + 2 h <-> flald + nad + h2o	flac + nadh + 2 h <-> flald + nad + h2o
FLALDTHR	Fluorobenzoate degradation	fluoroacetaldehyde:L-threonine aldehydetransferase	2.2.1.8			flald + thr <-> acal + 4flthr	flald + thr <-> acal + 4flthr	flald + thr <-> acal + 4flthr
DHFR1	Folate Biosynthesis	dihydrofolate reductase	1.5.1.3	H16_A2704	folA2	dhf + 2 h + nadph <-> nadp + thf	dhf + h + nadph <-> nadp + thf	dhf + h + nadph <-> nadp + thf
DHPS2	Folate Biosynthesis	dihydropteroate synthase	2.5.1.15	H16_A2446	folP	paba + ahhmd -> dhpt + ppi	paba + ahhmd -> dhpt + ppi + h	paba + ahhmd -> dhpt + ppi + h
GTPCHI	Folate Biosynthesis	GTP cyclohydrolase I	3.5.4.16	H16_B1967	folE	gtp + h2o -> ahdt + formate + h	gtp + h2o -> ahdt + formate + h	gtp + h2o -> ahdt + formate + h
FPGLUS1	Folate Biosynthesis	folylpolyglutamate synthase	6.3.2.17	H16_A2610	folC	atp + dhpt + glu -> adp + dhf + pi	atp + dhpt + glu -> adp + dhf + h + pi	atp + dhpt + glu -> adp + dhf + h + pi
ABZS	Folate Biosynthesis	4-aminobenzoate synthase	4.1.3.38	H16_A3087	unknown	adchor -> paba + h + pyr	adchor -> paba + h + pyr	adchor -> paba + h + pyr
ADCMS	Folate Biosynthesis	4-amino-4-deoxychorismate synthase	2.6.1.85	H16_A3087	unknown	chor + gln -> adchor + glu	chor + gln -> adchor + glu	chor + gln -> adchor + glu
DHNPTA	Folate Biosynthesis	dihydroneopterin aldolase	4.1.2.25	H16_A0259	folB	dhnpt -> ahhmp + glal	dhnpt -> ahhmp + glal	dhnpt -> ahhmp + glal
DHNMPDP	Folate Biosynthesis	Dihydroneopterin monophosphate dephosphorylase	3.6.1.-	H16_A0260/H16_A0711/H16_A0728/H16_A0917/H16_A0983/H16_A1118/H16_A1282/H16_A1904/H16_A2322/H16_A2683/H16_A2711/H16_A2943/H16_A3250/H16_A3452/H16_A3617/H16_A3646/H16_B0187/H16_B0193/H16_B0538/H16_B1571/H16_B1613/H16_B2032/H16_B2325/H16_B2575/	unknown/cpaF1/unknown/unknown/cpaF3/unknown/unknown/prpR/unknown/unknown/unknown/mrp/obg/unknown/uvrD3/parA2/cpaF2/unknown/poxR/uvrA2/unknown/unknown/norR2/unknown/	dhpmp + h2o -> dhnpt + pi	dhpmp + h2o -> dhnpt + pi	dhpmp + h2o -> dhnpt + pi

DHNTPP	Folate Biosynthesis	Dihydroneopterin triphosphate pyrophosphatase	3.6.1.-	H16_A0260/H16_A0711/H16_A0728/H16_A0917/H16_A0983/H16_A1118/H16_A1282/H16_A1904/H16_A2322/H16_A2683/H16_A2711/H16_A2943/H16_A3250/H16_A3452/H16_A3617/H16_A3646/H16_B0187/H16_B0193/H16_B0538/H16_B1571/H16_B1613/H16_B2032/H16_B2325/H16_B2575/	unknown/cpaF1/unknown/unknown/cpaF3/unknown/unknown/prpR/unknown/unknown/unknown/mrp/obg/unknown/uvrD3/parA2/cpaF2/unknown/poxR/uvrA2/unknown/unknown/norR2/unknown/	ahdt + h2o -> dhpmp + ppi	ahdt + h2o -> dhpmp + ppi + 2 h	ahdt + h2o -> dhpmp + ppi + 2 h
HMDPPK	Folate Biosynthesis	6-hydroxymethyl-dihydropterin pyrophosphokinase	2.7.6.3	H16_A3082	folK	ahhmp + atp -> ahhmd + amp	ahhmp + atp -> ahhmd + amp + h	ahhmp + atp -> ahhmd + amp + h
6PYRTP	Folate Biosynthesis	6-pyruvoyl tetrahydrobiopterin synthase	4.2.3.12	H16_A1924	ptpS	ahdt -> pythp + pppi	ahdt -> pythp + pppi	ahdt -> pythp + pppi + h
AKLPP	Folate Biosynthesis	alkaline phosphatase	3.1.3.1	H16_A2182/H16_A2183/H16_B0842	unknown/unknown/phoD	ahdt + 3 h2o -> dhnpt + 3 pi	ahdt + 3 h2o -> dhnpt + 3 pi + 3 h	ahdt + 3 h2o -> dhnpt + 3 pi + 2 h
DHPS1	Folate Biosynthesis	dihydropteroate synthase	2.5.1.15	H16_A2446	folP	ahhmp + paba -> dhpt + h2o	ahhmp + paba -> dhpt + h2o	ahhmp + paba -> dhpt + h2o
DHFR2	Folate Biosynthesis	dihydrofolate reductase	1.5.1.3	H16_A2704	folA2	dhf + nad <-> fl + nadh + h	dhf + nad <-> fl + nadh + h	dhf + nad <-> fl + nadh + h
DHFR3	Folate Biosynthesis	dihydrofolate reductase	1.5.1.3	H16_A2704	folA2	fl + nadh + h + h2 -> thf + nad	fl + nadh + h + h2 -> thf + nad	fl + nadh + h + h2 -> thf + nad
FPGLUS2	Folate Biosynthesis	folylpolyglutamate synthase	6.3.2.17	H16_A2610	folC	atp + thf + glu <-> adp + pi + thfglu	atp + thf + glu <-> adp + pi + thfglu + h	atp + thf + glu <-> adp + pi + thfglu + h
DHFR2p	Folate Biosynthesis	dihydrofolate reductase	1.5.1.3	H16_A2704	folA2	dhf + nadp <-> fl + nadph + 2 h	dhf + nadp <-> fl + nadph + h	dhf + nadp <-> fl + nadph + h
DHFR3p	Folate Biosynthesis	dihydrofolate reductase	1.5.1.3	H16_A2704	folA2	fl + nadph + 2 h + h2 -> thf + nadp	fl + nadph + h + h2 -> thf + nadp	fl + nadph + h + h2 -> thf + nadp
METTHFD	Folate Metabolism	methylenetetrahydrofolate dehydrogenase (NADP)	1.5.1.5	H16_A1370	folD	metthf + nadp <-> h + methf + nadph	metthf + nadp <-> methf + nadph	metthf + nadp <-> methf + nadph
FTHFDF	Folate Metabolism	formyltetrahydrofolate deformylase	3.5.1.10	H16_A2505/H16_B1956	unknown/purU	fthf + h2o -> formate + h + thf	fthf + h2o -> formate + h + thf	fthf + h2o -> formate + h + thf
GLYAMT	Folate Metabolism	aminomethyltransferase	2.1.2.10	H16_A1567/H16_A3619	gcvT2/gcvT1	gly + nad + thf <-> co2 + metthf + nadh + nh4	gly + nad + thf <-> co2 + metthf + nadh + nh4	gly + nad + thf <-> co2 + metthf + nadh + nh4
METTHFR	Folate Metabolism	5,10-methylenetetrahydrofolate reductase (NADH)	1.5.1.20	H16_A0246	unknown	h + metthf + nadh -> mthf + nad	h + metthf + nadh -> mthf + nad	h + metthf + nadh -> mthf + nad
GSDH	Fructose and Mannose metabolism	glucose/sorbosone dehydrogenase	1.1.1.-	H16_A0679/H16_A0893/H16_A1256/H16_A1828/H16_A2460/H16_A2586/H16_B0034/H16_B0572/H16_B0663/H16_B0831/H16_B1417/H16_B2561	unknown/unknown/unknown/unknown/abmB/unknown/wecC/unknown/unknown/unknown/unknown/unknown/	sbt6p + nadp <-> sb1p + nadph + 2 h	sbt6p + nadp <-> sb1p + nadph + h	sbt6p + nadp <-> sb1p + nadph + h
HEXf	Fructose and Mannose metabolism	hexokinase (D-fructose:ATP)	2.7.1.4	H16_B1503	frcK	atp + fru -> adp + f6p	atp + fru -> adp + f6p + h	atp + fru -> adp + f6p + h
MAN6PI	Fructose and Mannose metabolism	mannose-6-phosphate isomerase	5.3.1.8	H16_B1152	unknown	man6p <-> f6p	man6p <-> f6p	man6p <-> f6p
PMANM	Fructose and Mannose metabolism	phosphomannomutase	5.4.2.8	H16_A1847/H16_A2445/H16_A2885	manB1/manB3/manB2	man1p <-> man6p	man1p <-> man6p	man1p <-> man6p
MAN1PGT	Fructose and Mannose metabolism	mannose-1-phosphate guanylyltransferase (GDP)	2.7.7.22	H16_A1854/H16_A2905	manC1/manC2	gdp + man1p -> gdpmann + pi	gdp + man1p -> gdpmann + pi	gdp + man1p + h -> gdpmann + pi
GDPMAND	Fructose and Mannose metabolism	GDP-D-mannose dehydratase	4.2.1.47	H16_A2900	unknown	gdpmann -> gdpddman + h2o	gdpmann -> gdpddman + h2o	gdpmann -> gdpddman + h2o
ALRTs	Fructose and Mannose metabolism	aldehyde reductase	1.1.1.21	H16_A3186	unknown	sot + nadp <-> glc + nadph + 2 h	sot + nadp <-> glc + nadph + h	sot + nadp <-> glc + nadph + h
FRUP	Fructose and Mannose metabolism	phosphatase	3.1.3.-	H16_A0168/H16_A0520/H16_B0594/H16_B1063	aceK/unknown/unknown/unknown	bf2p + h2o <-> fru + pi	bf2p + h2o <-> fru + pi	bf2p + h2o <-> fru + pi
F26BP	Fructose and Mannose metabolism	fructose-2,6-bisphosphatase	3.1.3.46	H16_B0760	unknown	bf26p + h2o -> f6p + pi	bf26p + h2o -> f6p + pi	bf26p + h2o -> f6p + pi
UDPG4E	Galactose metabolism	UDPglucose 4-epimerase	5.1.3.2	H16_B0226/H16_B0283	galE/unknown	udpg <-> udpgal	udpg <-> udpgal	udpg <-> udpgal
GALT1PD	Galactose metabolism	Galactitol-1-phosphate dehydrogenase	1.1.1.251			galt1p + nad <-> h + nadh + t6p	galt1p + nad <-> h + nadh + t6p	galt1p + nad <-> h + nadh + t6p
ALRTgp	Galactose metabolism	aldehyde reductase	1.1.1.21	H16_A3186	unknown	glac + nadp + 2 h2o <-> galt + nadph + 2 h + o2	glac + nadp + 2 h2o <-> galt + nadph + h + o2	glac + nadp + 2 h2o <-> galt + nadph + h + o2
ALRTg	Galactose metabolism	aldehyde reductase	1.1.1.21	H16_A3186	unknown	glac + nad + 2 h2o <-> galt + nadh + h + o2	glac + nad + 2 h2o <-> galt + nadh + h + o2	glac + nad + 2 h2o <-> galt + nadh + h + o2
CATCHDG2	gamma-Hexachlorocyclohexane degradation	catechol 1,2-dioxygenase	1.13.11.1	H16_A1964/H16_B0968	catA/pcpA	tchrocat + o2 -> tchccm + 2 h	tchrocat + o2 -> tchccm + h	tchrocat + o2 -> tchccm + h
CMBLD1	gamma-Hexachlorocyclohexane degradation	carboxymethylenebutenolidase	3.1.1.45	H16_A2215/H16_A2739/H16_A3488	unknown/unknown/unknown	dchrocmo + h2o -> dchroee + h	dchrocmo + h2o -> dchroee + h	dchrocmo + h2o -> dchroee + h
MACR1	gamma-Hexachlorocyclohexane degradation	maleylacetate reductase	1.3.1.32	H16_A1786/H16_B0970	unknown/pcpE	dchroee + 2 nadh + h -> coadip + hcl + 2 nad	dchroee + 2 nadh + h -> coadip + hcl + 2 nad	dchroee + 2 nadh + h -> coadip + hcl + 2 nad
CATOCS	gamma-Hexachlorocyclohexane degradation	unclear reaction				coadip + h2o -> chac + succ + h	coadip -> chac + succ + h	coadip + h2o -> chac + succ + h
HACDHG	gamma-Hexachlorocyclohexane degradation	haloacetate dehalogenase	3.8.1.3	H16_A0197	unknown	chac + h2o -> glycolate + hcl + h	chac + h2o -> glycolate + hcl + h	chac + h2o -> glycolate + hcl + h
BPDDO	gamma-Hexachlorocyclohexane degradation	biphenyl-2,3-diol 1,2-dioxygenase	1.13.11.39	H16_B0654	bphC	chcatol + o2 -> chhmsald + h	chcatol + o2 -> chhmsald + h	chcatol + o2 -> chhmsald + h
CATCHDG3	gamma-Hexachlorocyclohexane degradation	catechol 1,2-dioxygenase	1.13.11.1	H16_A1964/H16_B0968	catA/pcpA	chcatol + o2 -> chccm + 2 h	chcatol + o2 -> chccm + 2 h	chcatol + o2 -> chccm + 2 h
CMBLD2	gamma-Hexachlorocyclohexane degradation	carboxymethylenebutenolidase	3.1.1.45	H16_A2215/H16_A2739/H16_A3488	unknown/unknown/unknown	tcmbo + h2o -> 2mac + h	tcmbo + h2o -> 2mac + h	tcmbo + h2o -> 2mac + h
UNSMONO	gamma-Hexachlorocyclohexane degradation	unspecific monooxygenase	1.14.14.1	H16_B0939/H16_B1009	cyp/unknown	parat + h2o -> parax + h2s	parat + h2o -> parax + h2s + h	parat + h2o -> parax + h2s + h
4NPHP1	gamma-Hexachlorocyclohexane degradation	4-nitrophenyl phosphatase	3.1.3.2	H16_B1238	unknown	ntphp + h2o -> pnp + pi	ntphp + h2o -> pnp + pi	ntphp + h2o -> pnp + pi + h
4NPHP2	gamma-Hexachlorocyclohexane degradation	4-nitrophenyl phosphatase	3.1.3.1	H16_A2182/H16_A2183	unknown/unknown	ntphp + h2o -> pnp + pi	ntphp + h2o -> pnp + pi	ntphp + h2o -> pnp + pi + h
FBMO8	gamma-Hexachlorocyclohexane degradation	flavin-binding monooxygenase	1.14.13.-	H16_A1145/H16_B0495/H16_B1480/H16_B2135	unknown/unknown/unknown/unknown	pnp + o2 + nadph + h -> pbzq + no2 + nadp + h2o	pnp + o2 + nadph -> pbzq + no2 + nadp + h2o	pnp + o2 + nadph + h -> pbzq + no2 + nadp + h2o
FBMO9	gamma-Hexachlorocyclohexane degradation	flavin-binding monooxygenase	1.14.13.-	H16_A1145/H16_B0495/H16_B1480/H16_B2135	unknown/unknown/unknown/unknown	hqn + nadph + 2 h + o2 -> thbn + nadp + h2o	hqn + nadph + h + o2 -> thbn + nadp + h2o	hqn + nadph + h + o2 -> thbn + nadp + h2o

PHE2MO4	gamma-Hexachlorocyclohexane degradation	phenol 2-monooxygenase	1.14.13.7	H16_B0539&H16_B0540&H16_B0541&H16_B0542&H16_B0543&H16_B0544	poxA&poxB&poxC&poxD&poxE&poxF	resoc + o2 + nadph + 2 h -> thbn + nadp + h2o	resoc + o2 + nadph + h -> thbn + nadp + h2o	resoc + o2 + nadph + h -> thbn + nadp + h2o
4NOXDR	gamma-Hexachlorocyclohexane degradation	Oxidoreductase	1.14.-.-	H16_B0730/H16_B0738/H16_B2129	unknown/unknown/unknown	nicatol + o2 + 3 h2 -> thbn + no2 + h2o + h	nicatol + o2 + 3 h2 -> thbn + no2 + h2o + h	nicatol + o2 + 3 h2 -> thbn + no2 + h2o
GERCT	Geraniol degrdation	geranic acid CoA-transferase	2.8.3.-	H16_B0355/H16_B0367/H16_B0488/H16_B0655/H16_B0656/H16_B0847/H16_B0914	unknown/unknown/unknown/unknown/unknown/unknown/unknown/	gerana + coa + h -> tgercoa + h2o	gerana + coa + h -> tgercoa + h2o	gerana + coa + h -> tgercoa + h2o
CITNCL	Geraniol degrdation	citronellyl-CoA ligase	6.2.1.-	H16_A0866/H16_A0871/H16_A1230/H16_A1519/H16_A1700/H16_A1718/H16_A2252/H16_A2794/H16_A2807/H16_A2978/H16_B0174/H16_B0677/H16_B0910/H16_B1148/H16_B1264/H16_B1335/H16_B1662/H16_B1709/H16_B2522	unknown/unknown/unknown/unknown/unknown/unknown/unknown/unknown/unknown/unknown/unknown/unknown/unknown/unknown/unknown/unknown/unknown/unknown/unknown/	citnl + coa + atp + h -> citnlcoa + amp + ppi	citnl + coa + atp -> citnlcoa + amp + ppi + h	citnl + coa + atp -> citnlcoa + amp + ppi + h
ACCOAA2	Geraniol degrdation	acetyl-CoA acyltransferase	2.3.1.16	H16_A0462/H16_A1290/H16_B0200	unknown/unknown/pcaF	7m3o6ocoa + coa -> 5mh4ecoa + accoa	7m3o6ocoa + coa -> 5mh4ecoa + accoa	7m3o6ocoa + coa -> 5mh4ecoa + accoa
ENCOAR	Geraniol degrdation	enoyl-CoA reductase	1.3.99.-	H16_A2143/H16_A2149/H16_A2808/H16_B0675/H16_B0699	unknown/unknown/unknown/unknown/unknown/	5mh4ecoa + fad -> 2e5mhdcoa + fadh2	5mh4ecoa + fad + h -> 2e5mhdcoa + fadh2	5mh4ecoa + fad + h -> 2e5mhdcoa + fadh2
ACCOAA3	Geraniol degrdation	acetyl-CoA acyltransferase	2.3.1.16	H16_A0462/H16_A1290/H16_B0200	unknown/unknown/pcaF	5m3o4hcoa + coa -> 3mccoa + accoa	5m3o4hcoa + coa -> 3mccoa + accoa	5m3o4hcoa + coa -> 3mccoa + accoa
SUCSD2	Glutamate metabolism	succinate-semialdehyde dehydrogenase (NADP)	1.2.1.16	H16_B0982/H16_B1179/H16_B1537/H16_B2057	gabD1/gabD2/gabD3/gabD4	h2o + nadp + sucsal -> 3 h + nadph + succ	h2o + nadp + sucsal -> 2 h + nadph + succ	h2o + nadp + sucsal -> 2 h + nadph + succ
GABAT1	Glutamate metabolism	4-aminobutyrate transaminase	2.6.1.19	H16_B0981	gabT	gaba + akg -> glu + sucsal	gaba + akg -> glu + sucsal	gaba + akg -> glu + sucsal
CABPS	Glutamate metabolism	carbamoyl-phosphate synthase (glutamine-hydrolysing)	6.3.5.5	H16_A2106/H16_A2452/H16_A2454	carB1/carB2/carA	2 atp + gln + h2o + hco3 -> 2 adp + cap + glu + h + pi	2 atp + gln + h2o + hco3 -> 2 adp + cap + glu + 2 h + pi	2 atp + gln + h2o + hco3 -> 2 adp + cap + glu + 2 h + pi
GLUR	Glutamate metabolism	glutamate racemase	5.1.1.3	H16_A2529	murI	dglu <-> glu	dglu <-> glu	dglu <-> glu
GGLUCYSS	Glutamate metabolism	gamma-glutamylcysteine synthetase	6.3.2.2	H16_A0322	gshA	atp + cys + glu -> adp + gcys + pi	atp + cys + glu -> adp + gcys + h + pi	atp + cys + glu -> adp + gcys + h + pi
GTHRDS	Glutamate metabolism	glutathione synthetase	6.3.2.3	H16_A0323	gshB	atp + gcys + gly -> adp + rgt + pi	atp + gcys + gly -> adp + rgt + h + pi	atp + gcys + gly -> adp + rgt + h + pi
GLUS	Glutamate metabolism	glutamate synthase	1.4.1.13	H16_A3430/H16_A3431/H16_B2192/H16_B2193/H16_B2194	gltD/gltB1/glxB/glxC/gltB2	akg + gln + 2 h + nadph -> 2 glu + nadp	akg + gln + h + nadph -> 2 glu + nadp	akg + gln + h + nadph -> 2 glu + nadp
GLUDH2	Glutamate Metabolism	glutamate dehydrogenase	1.4.1.4	H16_B1945	gdhA2	glu + h2o + nadp <-> akg + 2 h + nadph + nh4	glu + h2o + nadp <-> akg + h + nadph + nh4	glu + h2o + nadp <-> akg + h + nadph + nh4
GLUDH1	Glutamate Metabolism	glutamate dehydrogenase	1.4.1.3	H16_A0471	gdhA1	glu + h2o + nadp <-> akg + 2 h + nadph + nh4	glu + h2o + nadp <-> akg + h + nadph + nh4	glu + h2o + nadp <-> akg + h + nadph + nh4
GLUDH3	Glutamate Metabolism	glutamate dehydrogenase	1.4.1.2	H16_A1356	gudB	glu + h2o + nad <-> akg + h + nadh + nh4	glu + h2o + nad <-> akg + h + nadh + nh4	glu + h2o + nad <-> akg + h + nadh + nh4
GLUN1	Glutamate Metabolism	glutaminase	3.5.1.38	H16_A1910/H16_A2280	ansA/unknown	gln + h2o -> glu + nh4	gln + h2o -> glu + nh4	gln + h2o -> glu + nh4
GLUDCB	Glutamate Metabolism	glutamate Decarboxylase	4.1.1.19	H16_A2930	ldcC	glu + h -> gaba + co2	glu + h -> gaba + co2	glu + h -> gaba + co2
GLNST1	Glutamate metabolism	glutamine synthetase	6.3.1.2	H16_A2335/H16_B0618/H16_B2191	glna/glnA2/glnA3	atp + glu + nh4 -> adp + gln + pi	atp + glu + nh4 -> adp + gln + h + pi	atp + glu + nh4 -> adp + gln + h + pi
NADSG	Glutamate Metabolism	NAD+ synthase (glutamine-hydrolysing)	6.3.5.1	H16_A0749	nadE	atp + dnad + gln + h2o + h -> amp + ppi + nad + glu	atp + dnad + gln + h2o -> amp + ppi + nad + glu + h	atp + dnad + gln + h2o -> amp + ppi + nad + glu + h
GABAT2	Glutamate Metabolism	4-aminobutyrate transaminase	2.6.1.19	H16_B0981	gabT	gaba + akg <-> glu + sucsal	gaba + akg <-> glu + sucsal	gaba + akg <-> glu + sucsal
GTADT1	Glutamate metabolism	aspartyl-tRNA(Asn)/glutamyl-tRNA (Gln) amidotransferase	6.3.5.7	H16_A0108&H16_A0111&H16_A0112&H16_A1509&H16_A1882	gatB&gatA1&gatC&gatA3&unknown	glutrna + gln + atp + h2o -> glntrna + glu + pi + adp	glutrna + gln + atp + h2o -> glntrna + glu + pi + adp + h	glutrna + gln + atp + h2o -> glntrna + glu + pi + adp + h
MAAMPT	Glutathione Metabolism	membrane alanyl aminopeptidase	3.4.11.2	H16_A1000	pepN	h2o + progly -> gly + pro	h2o + progly -> gly + pro	h2o + progly -> gly + pro
GTSPMDS	Glutathione Metabolism	glutathionylspermidine synthetase	6.3.1.8	H16_A0042	unknown	atp + rgt + sprmd -> adp + gtspmd + pi	atp + rgt + sprmd -> adp + gtspmd + h + pi	atp + rgt + sprmd -> adp + gtspmd + h + pi
GTHHR	Glutathione Metabolism	glutathione hydralase	2.3.2.2	H16_A0784/H16_A1098/H16_A2780/H16_B0984	ggt2a/unknown/ggt2b/ggt2c	rgt + h2o -> cysgly + glu	rgt + h2o -> cysgly + glu	rgt + h2o -> cysgly + glu + h
ALAAPT	Glutathione Metabolism	alanyl aminopeptidase	3.4.11.2	H16_A1000	pepN	cysgly + h2o -> cys + gly	cysgly + h2o -> cys + gly	cysgly + h2o + h -> cys + gly
GTTPX	Glutathione metabolism	glutathione peroxidase	1.11.1.9	H16_A0065	unknown	h2o2 + 2 rgt <-> ogt + 2 h2o	h2o2 + 2 rgt <-> ogt + 2 h2o	h2o2 + 2 rgt <-> ogt + 2 h2o + 2 h
ALCDgl	Glycerolipid Metabolism	alcohol dehydrogenase (glycerol)	1.1.1.21	H16_A3186/H16_B2162	unknown/unknown	t3 + h + nadh <-> gl + nad	t3 + h + nadh <-> gl + nad	t3 + h + nadh <-> gl + nad
GLYCK	Glycerolipid Metabolism	glycerol kinase	2.7.1.30	H16_A2507/H16_B1199	unknown/unknown	atp + gl -> adp + glyc3p	atp + gl -> adp + glyc3p + h	atp + gl -> adp + glyc3p + h
ALHD2	Glycerolipid Metabolism	aldehyde dehydrogenase (NAD+)	1.2.1.3	H16_A0232/H16_A0745/H16_A1114/H16_A1495/H16_B0212/H16_B0421/H16_B0737/H16_B0833/H16_B1534/H16_B1735/H16_B1751/H16_B1835/H16_B1960/H16_B2444	unknown/unknown/unknown/unknown/unknown/unknown/unknown/unknown/unknown/unknown/unknown/unknown/exaC/unknown/	t3 + nad + h2o <-> glyc-R + nadh + 2 h	t3 + nad + h2o <-> glyc-R + nadh + 2 h	t3 + nad + h2o <-> glyc-R + nadh + 2 h
TAGL1	Glycerolipid Metabolism	triacylglycerol lipase	3.1.1.3	H16_A1322	unknown	dgr + h2o -> agl + 0.007 c120 + 0.419 c140 + 0.007 c150 + 0.254 c160 + 0.175 c161 + 0.006 c170 + 0.007 c171 + 0.013 c180 + 0.112 c181	dgr + h2o -> agl + 0.007 c120 + 0.419 c140 + 0.007 c150 + 0.254 c160 + 0.175 c161 + 0.006 c170 + 0.007 c171 + 0.013 c180 + 0.112 c181	dgr + h2o -> agl + 0.007 c120 + 0.419 c140 + 0.007 c150 + 0.254 c160 + 0.175 c161 + 0.006 c170 + 0.007 c171 + 0.013 c180 + 0.112 c181
TAGL1_Nlimit	Glycerolipid Metabolism	triacylglycerol lipase	3.1.1.3	H16_A1322	unknown	dgr + h2o -> agl + 0.002 c120 + 0.414 c140 + 0.001 c141 + 0.028 c150 + 0.01 c151 + 0.255 c160 + 0.06 c161 + 0.105 c170 + 0.008 c180 + 0.105 c181 + 0.012 c190	dgr + h2o -> agl + 0.002 c120 + 0.414 c140 + 0.001 c141 + 0.028 c150 + 0.01 c151 + 0.255 c160 + 0.06 c161 + 0.105 c170 + 0.008 c180 + 0.105 c181 + 0.012 c190	dgr + h2o -> agl + 0.002 c120 + 0.414 c140 + 0.001 c141 + 0.028 c150 + 0.01 c151 + 0.255 c160 + 0.06 c161 + 0.105 c170 + 0.008 c180 + 0.105 c181 + 0.012 c190
TAGL2	Glycerolipid Metabolism	triacylglycerol lipase	3.1.1.3	H16_A1322	unknown	tgl + h2o -> 0.007 c120 + 0.419 c140 + 0.007 c150 + 0.254 c160 + 0.175 c161 + 0.006 c170 + 0.007 c171 + 0.013 c180 + 0.112 c181	tgl + h2o -> 0.007 c120 + 0.419 c140 + 0.007 c150 + 0.254 c160 + 0.175 c161 + 0.006 c170 + 0.007 c171 + 0.013 c180 + 0.112 c181	tgl + h2o -> 0.007 c120 + 0.419 c140 + 0.007 c150 + 0.254 c160 + 0.175 c161 + 0.006 c170 + 0.007 c171 + 0.013 c180 + 0.112 c181
TAGL2_Nlimit	Glycerolipid Metabolism	triacylglycerol lipase	3.1.1.3	H16_A1322	unknown	tgl + h2o -> 0.002 c120 + 0.414 c140 + 0.001 c141 + 0.028 c150 + 0.01 c151 + 0.255 c160 + 0.06 c161 + 0.105 c170 + 0.008 c180 + 0.105 c181 + 0.012 c190	tgl + h2o -> 0.002 c120 + 0.414 c140 + 0.001 c141 + 0.028 c150 + 0.01 c151 + 0.255 c160 + 0.06 c161 + 0.105 c170 + 0.008 c180 + 0.105 c181 + 0.012 c190	tgl + h2o -> 0.002 c120 + 0.414 c140 + 0.001 c141 + 0.028 c150 + 0.01 c151 + 0.255 c160 + 0.06 c161 + 0.105 c170 + 0.008 c180 + 0.105 c181 + 0.012 c190
ALCDglp	Glycerolipid Metabolism	alcohol dehydrogenase (glycerol)	1.1.1.21	H16_A3186/H16_B2162	unknown/unknown	t3 + 2 h + nadph <-> gl + nadp	t3 + h + nadph <-> gl + nadp	t3 + h + nadph <-> gl + nadp
GL3PDp	Glycerophospholipid Metabolism	glycerol-3-phosphate dehydrogenase (NADP)	1.1.1.94	H16_A0336	gpsA	glyc3p + nadp <-> dhap + 2 h + nadph	glyc3p + nadp <-> dhap + h + nadph	glyc3p + nadp <-> dhap + h + nadph
ETHAML	Glycerophospholipid Metabolism	Ethanolamine ammonia-lyase	4.3.1.7	H16_B0096	eutB	etha -> acal + nh4	etha -> acal + nh4	etha -> acal + nh4
G3POAT	Glycerophospholipid Metabolism	glycerol-3-phosphate O-acyltransferase	2.3.1.15			glyc3p + 0.007 c120ACP + 0.419 c140ACP + 0.007 c150ACP + 0.254 c160ACP + 0.175 c161ACP + 0.006 c170ACP + 0.007 c171ACP + 0.013 c180ACP + 0.112 c181ACP -> agl3p + ACP	glyc3p + 0.007 c120ACP + 0.419 c140ACP + 0.007 c150ACP + 0.254 c160ACP + 0.175 c161ACP + 0.013 c180ACP + 0.112 c181ACP + 0.006 c170ACP + 0.007 c171ACP -> agl3p + ACP	glyc3p + 0.007 c120ACP + 0.419 c140ACP + 0.007 c150ACP + 0.254 c160ACP + 0.175 c161ACP + 0.006 c170ACP + 0.007 c171ACP + 0.013 c180ACP + 0.112 c181ACP -> agl3p + ACP
G3POAT_Nlimit	Glycerophospholipid Metabolism	glycerol-3-phosphate O-acyltransferase	2.3.1.15			glyc3p + 0.002 c120ACP + 0.414 c140ACP + 0.001 c141ACP + 0.028 c150ACP + 0.01 c151ACP + 0.255 c160ACP + 0.06 c161ACP + 0.105 c170ACP + 0.008 c180ACP + 0.105 c181ACP + 0.012 c190ACP -> agl3p + ACP	glyc3p + 0.002 c120ACP + 0.414 c140ACP + 0.001 c141ACP + 0.028 c150ACP + 0.01 c151ACP + 0.255 c160ACP + 0.06 c161ACP + 0.105 c170ACP + 0.008 c180ACP + 0.105 c181ACP + 0.012 c190ACP -> agl3p + ACP	glyc3p + 0.002 c120ACP + 0.414 c140ACP + 0.001 c141ACP + 0.028 c150ACP + 0.01 c151ACP + 0.255 c160ACP + 0.06 c161ACP + 0.105 c170ACP + 0.008 c180ACP + 0.105 c181ACP + 0.012 c190ACP -> agl3p + ACP

AG3POAT	Glycerophospholipid Metabolism	1-acylglycerol-3-phosphate O-acyltransferase	2.3.1.51	H16_A0519/ H16_A2911	plsC1/plsC2	agl3p + 0.007 c120ACP + 0.419 c140ACP + 0.007 c150ACP + 0.254 c160ACP + 0.175 c161ACP + 0.006 c170ACP + 0.007 c171ACP + 0.013 c180ACP + 0.112 c181ACP -> pa + ACP	agl3p + 0.007 c120ACP + 0.419 c140ACP + 0.007 c150ACP + 0.254 c160ACP + 0.175 c161ACP + 0.013 c180ACP + 0.112 c181ACP + 0.006 c170ACP + 0.007 c171ACP -> pa + ACP	agl3p + 0.007 c120ACP + 0.419 c140ACP + 0.007 c150ACP + 0.254 c160ACP + 0.175 c161ACP + 0.006 c170ACP + 0.007 c171ACP + 0.013 c180ACP + 0.112 c181ACP -> pa + ACP
AG3POAT_Nlimit	Glycerophospholipid Metabolism	1-acylglycerol-3-phosphate O-acyltransferase	2.3.1.51	H16_A0519/ H16_A2911	plsC1/plsC2	agl3p + 0.002 c120ACP + 0.414 c140ACP + 0.001 c141ACP + 0.028 c150ACP + 0.01 c151ACP + 0.255 c160ACP + 0.06 c161ACP + 0.105 c170ACP + 0.008 c180ACP + 0.105 c181ACP + 0.012 c190ACP -> pa + ACP	agl3p + 0.002 c120ACP + 0.414 c140ACP + 0.001 c141ACP + 0.028 c150ACP + 0.01 c151ACP + 0.255 c160ACP + 0.06 c161ACP + 0.105 c170ACP + 0.008 c180ACP + 0.105 c181ACP + 0.012 c190ACP -> pa + ACP	agl3p + 0.002 c120ACP + 0.414 c140ACP + 0.001 c141ACP + 0.028 c150ACP + 0.01 c151ACP + 0.255 c160ACP + 0.06 c161ACP + 0.105 c170ACP + 0.008 c180ACP + 0.105 c181ACP + 0.012 c190ACP -> pa + ACP
DGRK	Glycerophospholipid Metabolism	diacylglycerol kinase	2.7.1.107	H16_A1027	dgkA	dgr + atp -> adp + pa	dgr + atp -> adp + pa	dgr + atp -> adp + pa
PPTCT	Glycerophospholipid Metabolism	phosphatidate cytidylyltransferase	2.7.7.41	H16_A2088	unknown	pa + ctp -> cdpdg + ppi	pa + ctp -> cdpdg + ppi	pa + ctp -> cdpdg + ppi
PPGS	Glycerophospholipid Metabolism	Phosphatidylglycerol synthase	2.7.8.5	H16_A2546	unknown	cdpdg + glyc3p -> cmp + pgp	cdpdg + glyc3p -> cmp + pgp	cdpdg + glyc3p -> cmp + pgp
PPSERS	Glycerophospholipid Metabolism	Phosphatidylserine syntase	2.7.8.8	H16_A1039	pssA	cdpdg + ser -> cmp + ps	cdpdg + ser -> cmp + ps	cdpdg + ser -> cmp + ps
PLIPASA1C	Glycerophospholipid Metabolism	Phospholipase A1 (phosphatidylcholine)	3.1.1.32	H16_A1139	unknown	pc -> 2ag3pc + 0.007 c120 + 0.419 c140 + 0.007 c150 + 0.254 c160 + 0.175 c161 + 0.006 c170 + 0.007 c171 + 0.013 c180 + 0.112 c181	pc -> 2ag3pc + 0.007 c120 + 0.419 c140 + 0.007 c150 + 0.254 c160 + 0.175 c161 + 0.006 c170 + 0.007 c171 + 0.013 c180 + 0.112 c181	pc -> 2ag3pc + 0.007 c120 + 0.419 c140 + 0.007 c150 + 0.254 c160 + 0.175 c161 + 0.006 c170 + 0.007 c171 + 0.013 c180 + 0.112 c181
PLIPASA1C_Nlimit	Glycerophospholipid Metabolism	Phospholipase A1 (phosphatidylcholine)	3.1.1.32	H16_A1139	unknown	pc -> 2ag3pc + 0.002 c120 + 0.414 c140 + 0.001 c141 + 0.028 c150 + 0.01 c151 + 0.255 c160 + 0.06 c161 + 0.105 c170 + 0.008 c180 + 0.105 c181 + 0.012 c190	pc -> 2ag3pc + 0.002 c120 + 0.414 c140 + 0.001 c141 + 0.028 c150 + 0.01 c151 + 0.255 c160 + 0.06 c161 + 0.105 c170 + 0.008 c180 + 0.105 c181 + 0.012 c190	pc -> 2ag3pc + 0.002 c120 + 0.414 c140 + 0.001 c141 + 0.028 c150 + 0.01 c151 + 0.255 c160 + 0.06 c161 + 0.105 c170 + 0.008 c180 + 0.105 c181 + 0.012 c190
PLIPASA1S	Glycerophospholipid Metabolism	Phospholipase A1 (Phosphatidylserine)	3.1.1.32	H16_A1139	unknown	ps -> 2ag3ps + 0.007 c120 + 0.419 c140 + 0.007 c150 + 0.254 c160 + 0.175 c161 + 0.006 c170 + 0.007 c171 + 0.013 c180 + 0.112 c181	ps -> 2ag3ps + 0.007 c120 + 0.419 c140 + 0.007 c150 + 0.254 c160 + 0.175 c161 + 0.006 c170 + 0.007 c171 + 0.013 c180 + 0.112 c181	ps -> 2ag3ps + 0.007 c120 + 0.419 c140 + 0.007 c150 + 0.254 c160 + 0.175 c161 + 0.006 c170 + 0.007 c171 + 0.013 c180 + 0.112 c181
PLIPASA1S_Nlimit	Glycerophospholipid Metabolism	Phospholipase A1 (Phosphatidylserine)	3.1.1.32	H16_A1139	unknown	ps -> 2ag3ps + 0.002 c120 + 0.414 c140 + 0.001 c141 + 0.028 c150 + 0.01 c151 + 0.255 c160 + 0.06 c161 + 0.105 c170 + 0.008 c180 + 0.105 c181 + 0.012 c190	ps -> 2ag3ps + 0.002 c120 + 0.414 c140 + 0.001 c141 + 0.028 c150 + 0.01 c151 + 0.255 c160 + 0.06 c161 + 0.105 c170 + 0.008 c180 + 0.105 c181 + 0.012 c190	ps -> 2ag3ps + 0.002 c120 + 0.414 c140 + 0.001 c141 + 0.028 c150 + 0.01 c151 + 0.255 c160 + 0.06 c161 + 0.105 c170 + 0.008 c180 + 0.105 c181 + 0.012 c190
PLIPASA1E	Glycerophospholipid Metabolism	Phospholipase A1 (phosphatidylethanolamine)	3.1.1.32	H16_A1139	unknown	pe -> 2ag3pe + 0.007 c120 + 0.419 c140 + 0.007 c150 + 0.254 c160 + 0.175 c161 + 0.006 c170 + 0.007 c171 + 0.013 c180 + 0.112 c181	pe -> 2ag3pe + 0.007 c120 + 0.419 c140 + 0.007 c150 + 0.254 c160 + 0.175 c161 + 0.013 c180 + 0.112 c181 + 0.006 c170 + 0.007 c171	pe -> 2ag3pe + 0.007 c120 + 0.419 c140 + 0.007 c150 + 0.254 c160 + 0.175 c161 + 0.006 c170 + 0.007 c171 + 0.013 c180 + 0.112 c181
PLIPASA1E_Nlimit	Glycerophospholipid Metabolism	Phospholipase A1 (phosphatidylethanolamine)	3.1.1.32	H16_A1139	unknown	pe -> 2ag3pe + 0.002 c120 + 0.414 c140 + 0.001 c141 + 0.028 c150 + 0.01 c151 + 0.255 c160 + 0.06 c161 + 0.105 c170 + 0.008 c180 + 0.105 c181 + 0.012 c190	pe -> 2ag3pe + 0.002 c120 + 0.414 c140 + 0.001 c141 + 0.028 c150 + 0.01 c151 + 0.255 c160 + 0.06 c161 + 0.105 c170 + 0.008 c180 + 0.105 c181 + 0.012 c190	pe -> 2ag3pe + 0.002 c120 + 0.414 c140 + 0.001 c141 + 0.028 c150 + 0.01 c151 + 0.255 c160 + 0.06 c161 + 0.105 c170 + 0.008 c180 + 0.105 c181 + 0.012 c190
PGRPP	Glycerophospholipid Metabolism	phosphatidylglycerol phosphate phosphatase	3.1.3.27	H16_A3155	pgpA	pgp + h2o -> pg + pi	pgp + h2o -> pg + pi	pgp + h2o -> pg + pi
GPPDPD1	Glycerophospholipid Metabolism	Glycerophosphodiester phosphodiesterase (Glycerophosphocholine)	3.1.4.46	H16_A0499/H16_A2326	gdpD/ugpQ	g3pc + h2o -> choline + glyc3p	g3pc + h2o -> choline + glyc3p + h	g3pc + h2o -> choline + glyc3p + h
GPPDPD2	Glycerophospholipid Metabolism	Glycerophosphodiester phosphodiesterase (Glycerophosphoethanolamine)	3.1.4.46	H16_A0499/H16_A2326	gdpD/ugpQ	g3pe + h2o -> etha + glyc3p	g3pe + h2o -> etha + glyc3p + h	g3pe + h2o -> etha + glyc3p + h
GPPDPD3	Glycerophospholipid Metabolism	Glycerophosphodiester phosphodiesterase (Glycerophosphoserine)	3.1.4.46	H16_A0499/H16_A2326	gdpD/ugpQ	g3ps + h2o -> glyc3p + ser	g3ps + h2o -> glyc3p + ser + h	g3ps + h2o -> glyc3p + ser + h
GPPDPD4	Glycerophospholipid Metabolism	Glycerophosphodiester phosphodiesterase (Glycerophosphoglycerol)	3.1.4.46	H16_A0499/H16_A2326	gdpD/ugpQ	g3pg + h2o -> gl + glyc3p	g3pg + h2o -> gl + glyc3p + h	g3pg + h2o -> gl + glyc3p + h
GPPDPD5	Glycerophospholipid Metabolism	Glycerophosphodiester phosphodiesterase (Glycerophosphoinositol)	3.1.4.46	H16_A0499/H16_A2326	gdpD/ugpQ	g3pi + h2o -> glyc3p + mi	g3pi + h2o -> glyc3p + mi + h	g3pi + h2o -> glyc3p + mi + h
CDPDGP	Glycerophospholipid Metabolism	CDP-diacylglycerol pyrophosphatase	3.6.1.26	H16_B2144	unknown	cdpdg + h2o -> pa + cmp	cdpdg + h2o -> pa + cmp	cdpdg + h2o -> pa + cmp
PSERD	Glycerophospholipid Metabolism	Phosphatidylserine decarboxylase	4.1.1.65	H16_A1038	psd	ps -> pe + co2	ps -> pe + co2	ps -> pe + co2
CLPNS1	Glycerophospholipid Metabolism	cardiolipin synthase	2.7.8.-	H16_A0458/H16_B1255	unknown/unknown	2 pg <-> clpn + gl	2 pg <-> clpn + gl	2 pg <-> clpn + gl
PPLDc	Glycerophospholipid Metabolism	phospholipase D	3.1.4.4	H16_B0932/H16_B1107	unknown/unknown	pc + h2o -> pa + choline	pc + h2o -> pa + choline	pc + h2o -> pa + choline
PPLDe	Glycerophospholipid Metabolism	phospholipase D	3.1.4.4	H16_B0932/H16_B1107	unknown/unknown	pe + h2o -> pa + etha	pe + h2o -> pa + etha	pe + h2o -> pa + etha
PPLCc	Glycerophospholipid Metabolism	phospholipase C	3.1.4.3	H16_A2724/H16_B0534/ H16_B1067/H16_B1166	plcN1/plcN2/plcN3/ plcN4	pc + h2o -> dgr + cholp	pc + h2o -> dgr + cholp	pc + h2o -> dgr + cholp
PPLCe	Glycerophospholipid Metabolism	phospholipase C	3.1.4.3	H16_A2724/H16_B0534/ H16_B1067/H16_B1166	plcN1/plcN2/plcN3/ plcN4	pe + h2o -> dgr + ethap	pe + h2o -> dgr + ethap	pe + h2o -> dgr + ethap
PPLCg	Glycerophospholipid Metabolism	phospholipase C	3.1.4.3	H16_A2724/H16_B0534/ H16_B1067/H16_B1166	plcN1/plcN2/plcN3/ plcN4	pg + h2o -> dgr + glyc3p	pg + h2o -> dgr + glyc3p	pg + h2o -> dgr + glyc3p
CLPNS2	Glycerophospholipid Metabolism	cardiolipin synthase	2.7.8.-	H16_A0458/H16_B1255	unknown/unknown	pg + cdpdg -> clpn + cmp	pg + cdpdg -> clpn + cmp	pg + cdpdg -> clpn + cmp
ETNP	Glycerophospholipid metabolism	ethanolaminephosphotransferase	2.7.8.1			pe + cmp <-> cdpetn + dgr	pe + cmp <-> cdpetn + dgr	pe + cmp <-> cdpetn + dgr
GL3PD	Glycerophospholipid Metabolism	glycerol-3-phosphate dehydrogenase (NADP)	1.1.1.94	H16_A0336	gpsA	glyc3p + nad <-> dhap + h + nadh	glyc3p + nad <-> dhap + h + nadh	glyc3p + nad <-> dhap + h + nadh
HPYRR	Glycine, Serine and threonine Metabolism	Hydroxypyruvate reductase (NADH)	1.1.1.29	H16_B0611	hprA	h + hpyr + nadh -> glyc-R + nad	h + hpyr + nadh -> glyc-R + nad	h + hpyr + nadh -> glyc-R + nad
HPYRRp	Glycine, Serine and threonine Metabolism	Hydroxypyruvate reductase (NADPH)	1.1.1.81	H16_A2132/H16_A3601	ttuD2/ttuD1	2 h + hpyr + nadph -> glyc-R + nadp	h + hpyr + nadph -> glyc-R + nadp	h + hpyr + nadph -> glyc-R + nadp
THRDH	Glycine, Serine and threonine Metabolism	L-threonine dehydrogenase	1.1.1.103	H16_A1934	tdh	nad + thr -> 2aobut + h + nadh	nad + thr -> 2aobut + h + nadh	nad + thr -> 2aobut + 2 h + nadh
PGLCED	Glycine, Serine and threonine Metabolism	D-3-phosphoglycerate dehydrogenase	1.1.1.95	H16_A0185/H16_A3712/ H16_B0347/H16_B0466/ H16_B0824/H16_B0841/ H16_B1819	unknown/serA1/serA2/serA3/serA4/serA5/serA6	3pg + nad -> 3php + h + nadh	3pg + nad -> 3php + h + nadh	3pg + nad -> 3php + h + nadh
GLYHMT	Glycine, Serine and threonine Metabolism	glycine hydroxymethyltransferase	2.1.2.1	H16_A2834	glyA	ser + thf <-> gly + h2o + metthf	ser + thf <-> gly + h2o + metthf	ser + thf <-> gly + h2o + metthf
GLYCAT	Glycine, Serine and threonine Metabolism	glycine C-acetyltransferase	2.3.1.29	H16_B0819	kbl	accoa + gly <-> 2aobut + coa	accoa + gly <-> 2aobut + coa	accoa + gly <-> 2aobut + coa + h
PSERT	Glycine, Serine and threonine Metabolism	phosphoserine transaminase	2.6.1.52	H16_A0791	serC	3php + glu -> akg + pser	3php + glu -> akg + pser	3php + glu -> akg + pser
GLYCEK2	Glycine, Serine and threonine Metabolism	glycerate kinase	2.7.1.31	H16_B0612	glxK	atp + glyc-R -> 2pg + adp	atp + glyc-R -> 2pg + adp + h	atp + glyc-R -> 2pg + adp + h
PSERP	Glycine, Serine and threonine Metabolism	phosphoserine phosphatase (L-serine)	3.1.3.3	H16_A1452/H16_A3080/ H16_B1164	serB1/serB2/serB3	h2o + pser -> pi + ser	h2o + pser -> pi + ser	h2o + pser -> pi + ser
SERD	Glycine, Serine and threonine Metabolism	L-serine deaminase	4.3.1.17	H16_A3622	sdaA	ser -> nh4 + pyr	ser -> nh4 + pyr	ser -> nh4 + pyr
MNAO1	Glycine, Serine and threonine Metabolism	monoamine oxidase	1.4.3.4	H16_A0831	maoB	aact + h2o + o2 -> h2o2 + mtg + nh4	aact + h2o + o2 -> h2o2 + mtg + nh4	aact + h2o + o2 + h -> h2o2 + mtg + nh4

ALDRm	Glycine, Serine and threonine Metabolism	aldose reductase (methylglyoxal)	1.1.1.-	H16_A0679/H16_A0893/H16_A1256/H16_A1828/H16_A2460/H16_A2586/H16_B0034/H16_B0572/H16_B0663/H16_B0831/H16_B1417/H16_B2561	unknown/unknown/unknown/unknown/abmB/unknown/wecC/unknown/unknown/unknown/unknown/unknown/	2 h + mtg + nadph -> acetol + nadp	h + mtg + nadph -> acetol + nadp	h + mtg + nadph -> acetol + nadp
HSERD	Glycine, Serine and Threonine Metabolism	homoserine dehydrogenase (NADPH)	1.1.1.3	H16_A2266	thrA	hser + nadp <-> aspsa + 2 h + nadph	hser + nadp <-> aspsa + h + nadph	hser + nadp <-> aspsa + h + nadph
ASPSAD	Glycine, Serine and Threonine Metabolism	aspartate-semialdehyde dehydrogenase	1.2.1.11	H16_A2618	asd	aspsa + nadp + pi <-> basp + 3 h + nadph	aspsa + nadp + pi <-> basp + h + nadph	aspsa + nadp + pi <-> basp + h + nadph
HSERK	Glycine, Serine and Threonine Metabolism	homoserine kinase	2.7.1.39	H16_A2744/H16_A3212/H16_A3213/H16_A3222	thrB/unknown/unknown/unknown	atp + hser -> adp + phser	atp + hser -> adp + phser + h	atp + hser -> adp + phser + h
ASPK	Glycine, Serine and Threonine Metabolism	aspartate kinase	2.7.2.4	H16_A1225	lysC	asp + atp <-> basp + adp	asp + atp <-> basp + adp	asp + atp <-> basp + adp
THRAD2	Glycine, Serine and Threonine Metabolism	L-allo-threonine aldolase	4.1.2.5	H16_A2762	ltaA	athr -> acal + gly	athr -> acal + gly	athr -> acal + gly
THRAD1	Glycine, Serine and Threonine Metabolism	threonine aldolase	4.1.2.5	H16_A2762	ltaA	thr -> acal + gly	thr -> acal + gly	thr -> acal + gly
THRS	Glycine, Serine and Threonine Metabolism	threonine synthase	4.2.3.1	H16_A2265/H16_B0301	thrC/unknown	h2o + phser -> pi + thr	h2o + phser -> pi + thr	h2o + phser -> pi + thr
AOBUTDCs	Glycine, Serine and Threonine Metabolism	L-2-amino-3-oxobutanoate decarboxylation (spontaneous)		unknown	unknown	2aobut + h -> aact + co2	2aobut + h -> aact + co2	2aobut + h -> aact + co2
BETALDDH1	Glycine, Serine and Threonine Metabolism	betaine-aldehyde dehydrogenase	1.2.1.8	H16_B2130	betB	bal + h2o + nad -> glyb + 2 h + nadh	bal + h2o + nad -> glyb + 2 h + nadh	bal + h2o + nad -> glyb + 2 h + nadh
BETALDDH2	Glycine, Serine and Threonine Metabolism	betaine-aldehyde dehydrogenase	1.2.1.8	H16_B2130	betB	bal + h2o + nadp -> glyb + 3 h + nadph	bal + h2o + nadp -> glyb + 2 h + nadph	bal + h2o + nadp -> glyb + 2 h + nadph
SERDHT1	Glycine, Serine and Threonine Metabolism	L-serine dehydratase	4.3.1.19	H16_A0427/H16_B0554	unknown/tdcB	ser -> nh4 + pyr	ser -> nh4 + pyr	ser -> nh4 + pyr
THRD_L	Glycine, Serine and Threonine Metabolism	threonine dehydratase	4.3.1.19	H16_A0427/H16_B0554	unknown/tdcB	thr -> obut + nh4	thr -> obut + nh4	thr -> obut + nh4
GLYD	Glycine, Serine and Threonine Metabolism	glycine dehydrogenase	1.4.4.2	H16_A3621	gcvP	gly + lipop -> sap + co2	gly + lipop -> sap + co2	gly + lipop -> sap + co2
AMTF1	Glycine, Serine and Threonine Metabolism	aminomethyltransferase	2.1.2.10	H16_A3619	gcvT1	sap + thf + h -> dlipop + metthf + nh4	sap + thf + h -> dlipop + metthf + nh4	sap + thf + h -> dlipop + metthf + nh4
DLPD	Glycine, Serine and Threonine Metabolism	dihydrolipoamide dehydrogenase	1.8.1.4	H16_A1377/H16_A2323/H16_A3724/H16_B1098	pdhL/odhL/lpdaA/unknown	dlipop + nad -> lipop + nadh + h	dlipop + nad -> lipop + nadh + h	dlipop + nad -> lipop + nadh + h
CHOLD1	Glycine, Serine and Threonine Metabolism	choline dehydrogenase	1.1.99.1	H16_A0233/H16_A1655/H16_A3663/H16_A3737/H16_B2131	betA2/betA3/betA4/unknown/betA1	choline + fad -> bal + fadh2	choline + fad + h -> bal + fadh2	choline + fad + h -> bal + fadh2
CHOLD2	Glycine, Serine and Threonine Metabolism	choline dehydrogenase	1.1.99.1	H16_A0233/H16_A1655/H16_A3663/H16_A3737/H16_B2131	betA2/betA3/betA4/unknown/betA1	bal + fad + h2o -> fadh2 + glyb + h	bal + fad + h2o -> fadh2 + glyb	bal + fad + h2o -> fadh2 + glyb
BETHM	Glycine, Serine and Threonine Metabolism	betaine-homocysteine S-methyltransferase	2.1.1.5	H16_A0150	bhmT	glyb + hcys -> dimgly + met	glyb + hcys -> dimgly + met	glyb + hcys -> dimgly + met
DABOT	Glycine, Serine and Threonine Metabolism	diaminobutyrate-2-oxoglutarate transaminase	2.6.1.76	H16_B1692	unknown	glu + aspsa -> akg + 24dab	glu + aspsa -> akg + 24dab	glu + aspsa -> akg + 24dab
PGLCM	Glycolysis/Gluconeogenesis	phosphoglucomutase	5.4.2.2			g1p <-> g6p	g1p <-> g6p	g1p <-> g6p
LACDH	Glycolysis/Gluconeogenesis	L-lactate dehydrogenase	1.1.1.27	H16_A0666	ldh	llac + nad <-> pyr + nadh + h	llac + nad <-> pyr + nadh + h	llac + nad <-> pyr + nadh + h
GA3PD	Glycolysis/Gluconeogenesis	glyceraldehyde-3-phosphate dehydrogenase	1.2.1.12	H16_A3146/H16_B1386	gapA/cbbG2	g3p + nad + pi <-> 13pdg + nadh + 2 h	g3p + nad + pi <-> 13pdg + nadh + h	g3p + nad + pi <-> 13pdg + nadh + h
GLK	Glycolysis/Gluconeogenesis	glucokinase	2.7.1.2	H16_B2564	glk	atp + glc -> adp + g6p	atp + glc -> adp + g6p + h	atp + glc -> adp + g6p + h
PYK	Glycolysis/Gluconeogenesis	pyruvate kinase	2.7.1.40	H16_A0567/H16_A3602/H16_B0961	pyk1/pyk2/pyk3	adp + pep -> atp + pyr	adp + pep -> atp + pyr	adp + pep -> atp + pyr
PGK	Glycolysis/Gluconeogenesis	phosphoglycerate kinase	2.7.2.3	H16_A0566/H16_B1385	pgk/cbbK2	3pg + atp <-> 13pdg + adp	3pg + atp <-> 13pdg + adp	3pg + atp <-> 13pdg + adp
FBP	Glycolysis/Gluconeogenesis	fructose-bisphosphatase	3.1.3.11	H16_A0999/H16_B1390	fbp/cbbF2	fdp + h2o -> f6p + pi	fdp + h2o -> f6p + pi	fdp + h2o -> f6p + pi
APPS1	Glycolysis/Gluconeogenesis	acylphosphatase	3.6.1.7	H16_A3325	acyP	13pdg + h2o -> 3pg + pi	13pdg + h2o -> 3pg + pi + h	13pdg + h2o -> 3pg + pi + h
FBA	Glycolysis/Gluconeogenesis	fructose-bisphosphate aldolase	4.1.2.13	H16_A0568/H16_B0278/H16_B1384	fba/fbaB/cbbA2	fdp <-> dhap + g3p	fdp <-> dhap + g3p	fdp <-> dhap + g3p
ENO	Glycolysis/Gluconeogenesis	enolase	4.2.1.11	H16_A1188	eno	2pg <-> h2o + pep	2pg + h <-> h2o + pep	2pg + h <-> h2o + pep
TPI	Glycolysis/Gluconeogenesis	triose-phosphate isomerase	5.3.1.1	H16_A1047	tpiA	dhap <-> g3p	dhap <-> g3p	dhap <-> g3p
PGI	Glycolysis/Gluconeogenesis	glucose-6-phosphate isomerase	5.3.1.9	H16_A1502/H16_B1502	pgi1/pgi2	g6p <-> f6p	g6p <-> f6p	g6p <-> f6p
PGM	Glycolysis/Gluconeogenesis	phosphoglycerate mutase	5.4.2.1	H16_A0332/H16_A0493	pgam1/pgam2	2pg <-> 3pg	2pg <-> 3pg	2pg <-> 3pg
PDH1	Glycolysis/Gluconeogenesis	pyruvate dehydrogenase E1 component	1.2.4.1	H16_A1374/H16_A1753/H16_B0145/H16_B1300/H16_B2233/H16_B2234	pdhA1/pdhA2/acoB/aceE/bkdA1/bkdA2	pyr + lipo + h -> adlipo + co2	pyr + lipo + h -> adlipo + co2	pyr + lipo + h -> adlipo + co2
PDH2	Glycolysis/Gluconeogenesis	pyruvate dehydrogenase E2 component (dihydrolipoamide acetyltransferase)	2.3.1.12	H16_A1375/H16_B0146	pdhB/acoC	coa + adlipo -> accoa + dlipo	coa + adlipo -> accoa + dlipo	coa + adlipo -> accoa + dlipo
PDH3	Glycolysis/Gluconeogenesis	dihydrolipoamide dehydrogenase	1.8.1.4	H16_A1377/H16_A2323/H16_A3724/H16_B1098	pdhL/odhL/lpdaA/unknown	dlipo + nad -> lipo + nadh + h	dlipo + nad -> lipo + nadh + h	dlipo + nad -> lipo + nadh + h
TARSAR	Glyoxylate and Dicarboxylate metabolism	tartronate semialdehyde reductase	1.1.1.60	H16_A3600	unknown	h3op + h + nadh <-> glyc-R + nad	h3op + h + nadh <-> glyc-R + nad	h3op + h + nadh <-> glyc-R + nad
LCTAD1	Glyoxylate and Dicarboxylate metabolism	lactaldehyde dehydrogenase	1.2.1.21 (1.2.1.22 in kegg)	H16_A1919	unknown	h2o + llald + nad -> 2 h + llac + nadh	h2o + llald + nad -> 2 h + llac + nadh	h2o + llald + nad -> 2 h + llac + nadh
PGLYCP	Glyoxylate and Dicarboxylate metabolism	Phosphoglycolate phosphatase	3.1.3.18	H16_A0174/H16_A3318/H16_B1387	unknown/gph/cbbZ2	2ppg + h2o -> glycolate + pi	2ppg + h2o -> glycolate + pi	2ppg + h2o -> glycolate + pi
HPYRI	Glyoxylate and Dicarboxylate metabolism	hydroxypyruvate isomerase	5.3.1.22	H16_A1558/H16_A3599	hyi1/hyi2	hpyr <-> h3op	hpyr <-> h3op	hpyr <-> h3op
GLYCLTO	Glyoxylate and Dicarboxylate metabolism	Glycolate oxidase	1.1.1.29	H16_B0611	hprA	glycolate + uq -> glx + uqh2	glycolate + uq -> glx + uqh2	glycolate + uq -> glx + uqh2
GLCALDD	Glyoxylate and Dicarboxylate metabolism	Glycolaldehyde dehydrogenase	1.2.1.21			glal + h2o + nad -> glycolate + 2 h + nadh	glal + h2o + nad -> glycolate + 2 h + nadh	glal + h2o + nad -> glycolate + 2 h + nadh
GLYCEK1	Glyoxylate and Dicarboxylate metabolism	glycerate kinase	2.7.1.31	H16_B0612	glxK	atp + glyc-R -> 3pg + adp	atp + glyc-R -> 3pg + adp + h	atp + glyc-R -> 3pg + adp + h

GLOXCL	Glyoxylate and Dicarboxylate metabolism	glyoxalate carboligase	4.1.1.47	H16_A3598	unknown	2 glx + h -> h3op + co2	2 glx + h -> h3op + co2	2 glx + h -> h3op + co2
GLYCDH	Glyoxylate and Dicarboxylate metabolism	Glycolate dehydrogenase	1.1.1.29	H16_B0611	hprA	glx + h + nadh -> glycolate + nad	glx + h + nadh -> glycolate + nad	glx + h + nadh -> glycolate + nad
FDH	Glyoxylate and Dicarboxylate metabolism	formate dehydrogenase	1.2.1.2	(H16_A0640&H16_A0641&H16_A0642&H16_A0644)/H16_A2934&H16_A2936&H16_A2937&H16_B1471)/H16_A3292/H16_B1383/(H16_B1452&H16_B1453&H16_B1454)/(H16_B1700&H16_B1701)	(fdsG&fdsB&fdsA&fdsD)/(fdhC&fdhB1&fdhA1&fdhA2)/unknown/cbbB/(fdoG&fdoH&fdoI)/(fdwA&fdwB)	formate + nad <-> co2 + nadh	formate + nad <-> co2 + nadh	formate + nad <-> co2 + nadh
ACFM1	Glyoxylate and Dicarboxylate metabolism	arylformamidase	3.5.1.9	H16_A3005/H16_B1997	unknown/unknown	forkn + h2o -> formate + kn + h	forkn + h2o -> formate + kn + h	forkn + h2o -> formate + kn + h
FMDF	Glyoxylate and Dicarboxylate metabolism	formylmethionine deformylase	3.5.1.31	H16_A3700	def	formt + h2o -> formate + met	formt + h2o -> formate + met	formt + h2o -> formate + met
FMMS	Glyoxylate and Dicarboxylate metabolism	formamidase	3.5.1.49	H16_B0072/H16_B0476	fmdA1/fmdA2	fa + h2o -> formate + nh4	fa + h2o -> formate + nh4	fa + h2o -> formate + nh4
FGDM	Glyoxylate and Dicarboxylate metabolism	N-formylglutamate deformylase	3.5.1.68	H16_A1109/H16_A1306/H16_A3013/H16_A3649	hutG2/hutG3/hutG1/hutG4	forglu + h2o -> formate + glu	forglu + h2o -> formate + glu	forglu + h2o -> formate + glu
S2HAO	Glyoxylate and Dicarboxylate metabolism	(S)-2-hydroxy-acid oxidase	1.1.3.15	H16_A3094/H16_A3096/H16_A3097	glcD1/glcE/glcF	glycolate + o2 -> glx + h2o2	glycolate + o2 -> glx + h2o2	glycolate + o2 -> glx + h2o2
HISTDH	Histidine Metabolism	histidinol dehydrogenase	1.1.1.23	H16_A1694/H16_A3416	unknown/hisD	h2o + hisol + 2 nad -> 3 h + his + 2 nadh	h2o + hisol + 2 nad -> 3 h + his + 2 nadh	h2o + hisol + 2 nad -> 3 h + his + 2 nadh
ATPPRT	Histidine Metabolism	ATP phosphoribosyltransferase	2.4.2.17	H16_A3417	hisG	atp + prpp -> ppi + prbatp	atp + prpp -> ppi + prbatp + h	atp + prpp -> ppi + prbatp + h
HISTPT	Histidine Metabolism	histidinol-phosphate transaminase	2.6.1.9	H16_A0793/H16_A3415	hisC1/hisC2	glu + imACP -> akg + hisolp	glu + imACP -> akg + hisolp	glu + imACP -> akg + hisolp
HISTP	Histidine Metabolism	histidinol-phosphatase	3.1.3.15			h2o + hisolp -> hisol + pi	h2o + hisolp -> hisol + pi	h2o + hisolp -> hisol + pi
PRAMPCH	Histidine Metabolism	phosphoribosyl-AMP cyclohydrolase	3.5.4.19	H16_A3409	hisI	h2o + prbamp + h -> prfp	h2o + prbamp -> prfp	h2o + prbamp -> prfp
PRATPPP	Histidine Metabolism	phosphoribosyl-ATP pyrophosphatase	3.6.1.31	H16_A3408	hisE	h2o + prbatp -> ppi + prbamp	h2o + prbatp -> ppi + prbamp + h	h2o + prbatp -> ppi + prbamp + 2 h
IMGPDH	Histidine Metabolism	imidazoleglycerol-phosphate dehydratase	4.2.1.19	H16_A3414	hisB	dimgp + h -> h2o + imACP	dimgp -> h2o + imACP	dimgp -> h2o + imACP
PRMIIZCI	Histidine Metabolism	1-(5-phosphoribosyl)-5-[(5-phosphoribosylamino)methylideneamino)imidazole-4-carboxamide isomerase	5.3.1.16	H16_A3411	hisA	prfp <-> prlp	prfp <-> prlp	prfp <-> prlp
IMG3PS	Histidine Metabolism	Imidazole-glycerol-3-phosphate synthase	4.1.3.-/2.4.2.-	H16_A3410/H16_A3412	hisF/hisH	gln + prlp -> aicar + dimgp + glu + 2 h	gln + prlp -> aicar + dimgp + glu + 2 h	gln + prlp -> aicar + dimgp + glu + h
HISAL	Histidine metabolism	histidine ammonia-lyase	4.3.1.3	H16_A3018	hutH	his -> urocan + nh4	his -> urocan + nh4	his -> urocan + nh4
UROCH	Histidine metabolism	urocanate hydratase	4.2.1.49	H16_A0695/H16_A3017	hutU2/hutU1	urocan + h2o -> 4i5p + h	urocan + h2o -> 4i5p + h	urocan + h2o -> 4i5p + h
FBMO1	Histidine metabolism	flavin-binding monooxygenase	1.14.13.-	H16_A1145/H16_B0495/H16_B1480/H16_B2135	unknown/unknown/unknown/unknown	2 4i5p + o2 -> 2 ht5p	2 4i5p + o2 + 2 h -> 2 ht5p	2 4i5p + o2 + 2 h -> 2 ht5p
IMZPP	Histidine metabolism	imidazolonepropionase	3.5.2.7	H16_A3015	hutI	4i5p + h2o -> nfglu	4i5p + h2o + h -> nfglu	4i5p + h2o + h -> nfglu
FORGD	Histidine metabolism	formimidoylglutamate deiminase	3.5.3.13	H16_A3014	hutF	nfglu + h2o -> forglu + nh4	nfglu + h2o -> forglu + nh4	nfglu + h2o -> forglu + nh4
HTTOG	Histidine metabolism	lumping reaction				ht5p + 2 h2o + h -> glu + nh4 + co2	ht5p + 2 h2o + h -> glu + nh4 + co2	ht5p + 2 h2o + h -> glu + nh4 + co2
ALHD7	Histidine metabolism	aldehyde dehydrogenase (NAD+)	1.2.1.3	H16_A0232/H16_A0745/H16_A1114/H16_A1495/H16_B0212/H16_B0421/H16_B0737/H16_B0833/H16_B1534/H16_B1735/H16_B1751/H16_B1835/H16_B1960/H16_B2444	unknown/unknown/unknown/unknown/unknown/unknown/unknown/unknown/unknown/unknown/unknown/unknown/exaC/unknown/	i4aa + nad + h2o -> i4ac + nadh + 2 h	i4aa + nad + h2o -> i4ac + nadh + 2 h	i4aa + nad + h2o -> i4ac + nadh + 2 h
MNAO2	Histidine metabolism	monoamine oxidase	1.4.3.4	H16_A0831	maoB	nmhis + h2o + o2 -> mlzac + nh4 + h2o2	nmhis + h2o + o2 -> mlzac + nh4 + h2o2	nmhis + h2o + o2 -> mlzac + nh4 + h2o2
MSDHA	Inositol metabolism	malonate-semialdehyde dehydrogenase (acetylating)	1.2.1.27	H16_A0273/H16_A3664/H16_B1191	mmsA1/mmsA2/mmsA3	3opp + coa + nad -> accoa + co2 + nadh	3opp + coa + nad -> accoa + co2 + nadh	3opp + coa + nad -> accoa + co2 + nadh
MI1P	Inositol Phosphate Metabolism	myo-inositol 1-phosphatase	3.1.3.25	H16_A1214	suhB	h2o + dmi1p -> mi + pi	h2o + dmi1p -> mi + pi	h2o + dmi1p -> mi + pi
UDPAGAT	Lipopolysaccharide Biosynthesis	UDP-N-acetylglucosamine acyltransferase	2.3.1.129	H16_A2043	lpxA	3hmrsACP + udpnag <-> ACP + udpg2aa	3hmrsACP + udpnag <-> ACP + udpg2aa	3hmrsACP + udpnag <-> ACP + udpg2aa
LIPADSS	Lipopolysaccharide Biosynthesis	Lipid A disaccaride synthase	2.4.1.182	H16_A2042	lpxB	lipidX + udpg23a -> lipidAds + udp	lipidX + udpg23a + h -> lipidAds + udp	lipidX + udpg23a -> lipidAds + udp
TADSK	Lipopolysaccharide Biosynthesis	Tetraacyldisaccharide 4'kinase	2.7.1.130	H16_A0606	lpxK	atp + lipidAds -> adp + lipidA	atp + lipidAds -> adp + lipidA + 2 h	atp + lipidAds -> adp + lipidA + 2 h
KDOCTT	Lipopolysaccharide Biosynthesis	3-deoxy-manno-octulosonate cytidylyltransferase	2.7.7.38	H16_A0604	kdsB	ctp + kdo -> ckdo + ppi	ctp + kdo -> ckdo + ppi + h	ctp + kdo -> ckdo + ppi + h
KDO8PP	Lipopolysaccharide Biosynthesis	3-deoxy-manno-octulosonate-8-phosphatase	3.1.3.45			h2o + kdo8p -> kdo + pi	h2o + kdo8p -> kdo + pi	h2o + kdo8p -> kdo + pi
KDO8PS	Lipopolysaccharide Biosynthesis	2-dehydro-3-deoxyphosphooctonate aldolase (3-deoxy-D-manno-octulosonic -acid 8-phosphate synthase)	2.5.1.55	H16_A1186	kdsA	a5p + h2o + pep -> kdo8p + pi	a5p + h2o + pep -> h + kdo8p + pi	a5p + h2o + pep -> h + kdo8p + pi
AGMHEP	Lipopolysaccharide Biosynthesis	ADP-D-glycero-D-manno-heptose epimerase	5.1.3.20	H16_A0804	hldD	adpdgdmhep -> adphep	adpdgdmhep -> adphep	adpdgdmhep -> adphep
EDOTXS1	Lipopolysaccharide Biosynthesis	Endotoxin Synthesis (lauroyl transferase)	2.3.1.-	H16_A0228/H16_A0229/H16_B0917	unknown/unknown/unknown	c120ACP + k2lipiv -> ACP + lk2lipiv	c120ACP + k2lipiv -> ACP + lk2lipiv	c120ACP + k2lipiv -> ACP + lk2lipiv
EDOTXS2	Lipopolysaccharide Biosynthesis	Endotoxin Synthesis (myristoyl transferase)	2.3.1.-			lk2lipiv + c140ACP -> ACP + lipa	lk2lipiv + c140ACP -> ACP + lipa	lk2lipiv + c140ACP -> ACP + lipa
GMHEPADT	Lipopolysaccharide Biosynthesis	D-glycero-D-manno-hepose 1-phosphate adenyltransferase	2.7.-.-	H16_A0803	hldA	atp + dgdmh1p -> adpdgdmhep + ppi	atp + dgdmh1p -> adpdgdmhep + ppi	atp + dgdmh1p -> adpdgdmhep + ppi
GMHEPK	Lipopolysaccharide Biosynthesis	D-glycero-D-manno-heptose 7-phosphate kinase	2.7.-.-	H16_A0803	hldA	atp + dgdmh7p -> adp + dgdmh17bp	atp + dgdmh7p -> adp + dgdmh17bp + h	atp + dgdmh7p -> adp + dgdmh17bp + h
GMHEPBP	Lipopolysaccharide Biosynthesis	D-glycero-D-manno-heptose 1,7-bisphosphate phosphatase	3.1.1.-			dgdmh17bp + h2o -> dgdmh1p + pi	dgdmh17bp + h2o -> dgdmh1p + pi	dgdmh17bp + h2o -> dgdmh1p + pi
DMOAT	Lipopolysaccharide Biosynthesis	3-deoxy-D-manno-octulosonic acid transferase	2.4.99.-	H16_A2883	kdtA	ckdo + lipidA -> cmp + kdolipid4	ckdo + lipidA -> cmp + kdolipid4 + h	ckdo + lipidA -> cmp + kdolipid4 + h

DMOAT2	Lipopolysaccharide Biosynthesis	3-deoxy-D-manno-octulosonic acid transferase	2.4.99.-	H16_A2883	kdtA	ckdo + kdolipid4 -> cmp + k2lipiv	ckdo + kdolipid4 -> cmp + k2lipiv + h	ckdo + kdolipid4 -> cmp + k2lipiv + h
S7PISM	Lipopolysaccharide Biosynthesis	sedoheptulose 7-phosphate isomerase	5.-.-.-			s7p -> dgdmh7p	s7p -> dgdmh7p	s7p -> dgdmh7p
U3HGAAT	Lipopolysaccharide Biosynthesis	UDP-3-O-(3-hydroxymyristoyl)glucosamine acyltransferase	2.3.1.-	H16_A2045	lpxD	3hmrsACP + u3hga -> ACP + h + udpg23a	3hmrsACP + u3hga -> ACP + h + udpg23a	3hmrsACP + u3hga -> ACP + udpg23a
U3AGDA	Lipopolysaccharide Biosynthesis	UDP-3-O-acetylglucosamine deacetylase	3.5.1.-	H16_A3266	lpxC	h2o + udpg2aa -> ac + u3hga	h2o + udpg2aa -> ac + u3hga	h2o + udpg2aa -> ac + u3hga + h
UDPSH	Lipopolysaccharide Biosynthesis	UDP-sugar hydrolase	3.6.1.-			h2o + udpg23a -> lipidX + ump	h2o + udpg23a -> lipidX + ump + 2 h	h2o + udpg23a -> lipidX + ump + 2 h
DHDCR	Lysine Biosynthesis	dihydrodipicolinate reductase (NADPH)	1.3.1.26	H16_A3141/H16_A3348	dapB/unknown	dhdp + 2 h + nadph -> nadp + tdhdp	dhdp + h + nadph -> nadp + tdhdp	dhdp + h + nadph -> nadp + tdhdp
THDPSUC	Lysine Biosynthesis	tetrahydrodipicolinate succinylase	2.3.1.117	H16_A2066	dapD	h2o + succoa + tdhdp -> coa + sl2a6o	h2o + succoa + tdhdp -> coa + sl2a6o	h2o + succoa + tdhdp -> coa + sl2a6o
SUCDPT	Lysine Biosynthesis	succinyldiaminopimelate transaminase	2.6.1.17	H16_A2065/H16_A3025	unknown/argD	akg + sl26da <-> glu + sl2a6o	akg + sl26da <-> glu + sl2a6o	akg + sl26da <-> glu + sl2a6o
SUCDPDS	Lysine Biosynthesis	succinyl-diaminopimelate desuccinylase	3.5.1.18	H16_A2069	dapE	h2o + sl26da -> 26dap-LL + succ	h2o + sl26da -> 26dap-LL + succ	h2o + sl26da -> 26dap-LL + succ
DAPMDC	Lysine Biosynthesis	diaminopimelate decarboxylase	4.1.1.20	H16_A3443	lysA2	26dap-M + h -> co2 + lys	26dap-M + h -> co2 + lys	26dap-M + h -> co2 + lys
DHDPCS	Lysine Biosynthesis	dihydrodipicolinate synthase	4.2.1.52	H16_A1204/H16_B0213/H16_B0891/H16_B1831	dapA1/dapA2/dapA3/dapA4	aspsa + pyr -> dhdp + h + 2 h2o	aspsa + pyr -> dhdp + h + 2 h2o	aspsa + pyr -> dhdp + h + 2 h2o
DAPME	Lysine Biosynthesis	diaminopimelate epimerase	5.1.1.7	H16_A0227	dapF	26dap-LL <-> 26dap-M	26dap-LL <-> 26dap-M	26dap-LL <-> 26dap-M
GLUCD1	Lysine degradation	glutaryl-CoA dehydrogenase	1.3.99.7	H16_A2818	gcdH	glutcoa + nad -> ccoa + nadh + co2	glutcoa + nad -> ccoa + nadh + co2	glutcoa + nad -> ccoa + nadh + co2
OGDH2	Lysine degradation	2-oxoglutarate dehydrogenase E2 component (dihydrolipoamide succinyltransferase)	2.3.1.61	H16_A2324	odhB	coa + sgdhl <-> glutcoa + dlipoe	coa + sgdhl <-> glutcoa + dlipoe	coa + sgdhl <-> glutcoa + dlipoe
OGDH1	Lysine degradation	2-oxoglutarate dehydrogenase E1 component	1.2.4.2	H16_A2325	odhA	2oad + lipoe + h -> sgdhl + co2	2oad + lipoe + h -> sgdhl + co2	2oad + lipoe + h -> sgdhl + co2
ENCOAH3	Membrane Lipid Metabolism	enoyl-CoA hydratase	4.2.1.17	H16_A0100/H16_A0142/H16_A0179/H16_A0461/H16_A0464/H16_A0810/H16_A0865/H16_A0873/H16_A1101/H16_A1410/H16_A1699/H16_A1716/H16_A1719/H16_A1832/H16_A1885/H16_A1889/H16_A2138/H16_A2258/H16_A2979/H16_A3201/H16_A3311/H16_A3593/H16_A3594/H16_B0365/H16_B0382/H16_B0389/H16_B0402/H16_B0419/H16_B0420/H16_B0657/H16_B0659/H16_B0698/H16_B0724/H16_B0756/H16_B0848/H16_B0915/H16_B1188/H16_B1346/H16_B1439/H16_B1738/H16_B1741/H16_B1742/H16_B1773/H16_B1905/H16_B1914/H16_B2156/H16_B2478/	unknown/	3mccoa + h2o <-> 3hivcoa	3mccoa + h2o <-> 3hivcoa	3mccoa + h2o <-> 3hivcoa
ENCOAH4	Membrane Lipid Metabolism	enoyl-CoA hydratase	4.2.1.17	H16_A0100/H16_A0142/H16_A0179/H16_A0461/H16_A0464/H16_A0810/H16_A0865/H16_A0873/H16_A1101/H16_A1410/H16_A1699/H16_A1716/H16_A1719/H16_A1832/H16_A1885/H16_A1889/H16_A2138/H16_A2258/H16_A2979/H16_A3201/H16_A3311/H16_A3593/H16_A3594/H16_B0365/H16_B0382/H16_B0389/H16_B0402/H16_B0419/H16_B0420/H16_B0657/H16_B0659/H16_B0698/H16_B0724/H16_B0756/H16_B0848/H16_B0915/H16_B1188/H16_B1346/H16_B1439/H16_B1738/H16_B1741/H16_B1742/H16_B1773/H16_B1905/H16_B1914/H16_B2156/H16_B2478/	unknown/	2mp2ecoa + h2o -> 3hibcoa	2mp2ecoa + h2o -> 3hibcoa	2mp2ecoa + h2o -> 3hibcoa
ENCOAH5	Membrane Lipid Metabolism	enoyl-CoA hydratase	4.2.1.17	H16_A0100/H16_A0142/H16_A0179/H16_A0461/H16_A0464/H16_A0810/H16_A0865/H16_A0873/H16_A1101/H16_A1410/H16_A1699/H16_A1716/H16_A1719/H16_A1832/H16_A1885/H16_A1889/H16_A2138/H16_A2258/H16_A2979/H16_A3201/H16_A3311/H16_A3593/H16_A3594/H16_B0365/H16_B0382/H16_B0389/H16_B0402/H16_B0419/H16_B0420/H16_B0657/H16_B0659/H16_B0698/H16_B0724/H16_B0756/H16_B0848/H16_B0915/H16_B1188/H16_B1346/H16_B1439/H16_B1738/H16_B1741/H16_B1742/H16_B1773/H16_B1905/H16_B1914/H16_B2156/H16_B2478/	unknown/	2m2ecoa + h2o -> s3h2mbcoa	2m2ecoa + h2o -> s3h2mbcoa	2m2ecoa + h2o -> s3h2mbcoa
HACOAD2	Membrane Lipid Metabolism	3-hydroxyacyl-CoA dehydrogenase (3-oxohexanoyl-CoA)	1.1.1.35	H16_A0282/H16_A0602/H16_A1102/H16_A1888/H16_B0388/H16_B0724/H16_B1652	paaH1/unknown/paaH2/unknown/unknown/unknown/unknown	s3h2mbcoa + nad <-> 2maaccoa + nadh + h	s3h2mbcoa + nad <-> 2maaccoa + nadh + h	s3h2mbcoa + nad <-> 2maaccoa + nadh + h
HACOAD3	Membrane Lipid Metabolism	3-hydroxyacyl-CoA dehydrogenase (3-oxooctanoyl-CoA)	1.1.1.35	H16_A0282/H16_A0602/H16_A1102/H16_A1888/H16_B0388/H16_B0724/H16_B1652	paaH1/unknown/paaH2/unknown/unknown/unknown/unknown	hibut + nad <-> mmsa + nadh + h	hibut + nad <-> mmsa + nadh + h	hibut + nad <-> mmsa + nadh + h

ENCOAH6	Membrane Lipid Metabolism	enoyl-CoA hydratase	4.2.1.17	H16_A0100/H16_A0142/H16_A0179/H16_A0461/H16_A0464/H16_A0810/H16_A0865/H16_A0873/H16_A1101/H16_A1410/H16_A1699/H16_A1716/H16_A1719/H16_A1832/H16_A1885/H16_A1889/H16_A2138/H16_A2258/H16_A2979/H16_A3201/H16_A3311/H16_A3593/H16_A3594/H16_B0365/H16_B0382/H16_B0389/H16_B0402/H16_B0419/H16_B0420/H16_B0657/H16_B0659/H16_B0698/H16_B0724/H16_B0756/H16_B0848/H16_B0915/H16_B1188/H16_B1346/H16_B1439/H16_B1738/H16_B1741/H16_B1742/H16_B1773/H16_B1905/H16_B1914/H16_B2156/H16_B2478/	unknown/	carpcoa + h2o -> hadpcoa	carpcoa + h2o -> hadpcoa	carpcoa + h2o -> hadpcoa
HACOAD4	Membrane Lipid Metabolism	3-hydroxyacyl-CoA dehydrogenase (3-oxodecanoyl-CoA)	1.1.1.35	H16_A0282/H16_A0602/H16_A1102/H16_A1888/H16_B0388/H16_B0724/H16_B1652	paaH1/unknown/paaH2/unknown/unknown/unknown/unknown	hadpcoa + nad -> ooadpcoa + nadh + h	hadpcoa + nad -> ooadpcoa + nadh + h	hadpcoa + nad -> ooadpcoa + nadh + h
ENCOAH7	Membrane Lipid Metabolism	enoyl-CoA hydratase	4.2.1.17	H16_A0100/H16_A0142/H16_A0179/H16_A0461/H16_A0464/H16_A0810/H16_A0865/H16_A0873/H16_A1101/H16_A1410/H16_A1699/H16_A1716/H16_A1719/H16_A1832/H16_A1885/H16_A1889/H16_A2138/H16_A2258/H16_A2979/H16_A3201/H16_A3311/H16_A3593/H16_A3594/H16_B0365/H16_B0382/H16_B0389/H16_B0402/H16_B0419/H16_B0420/H16_B0657/H16_B0659/H16_B0698/H16_B0724/H16_B0756/H16_B0848/H16_B0915/H16_B1188/H16_B1346/H16_B1439/H16_B1738/H16_B1741/H16_B1742/H16_B1773/H16_B1905/H16_B1914/H16_B2156/H16_B2478/	unknown/	2e5mhdcoa + h2o -> 3h5m4ecoa	2e5mhdcoa + h2o -> 3h5m4ecoa	2e5mhdcoa + h2o -> 3h5m4ecoa
HACOAD5	Membrane Lipid Metabolism	3-hydroxyacyl-CoA dehydrogenase (3-oxododecanoyl-CoA)	1.1.1.35	H16_A0282/H16_A0602/H16_A1102/H16_A1888/H16_B0388/H16_B0724/H16_B1652	paaH1/unknown/paaH2/unknown/unknown/unknown/unknown	3h5m4ecoa + nad -> 5m3o4hcoa + nadh + h	3h5m4ecoa + nad -> 5m3o4hcoa + nadh + h	3h5m4ecoa + nad -> 5m3o4hcoa + nadh + h
3HMYSAS	Membrane Lipid Metabolism	3-hydroxy-myristoyl-ACP synthesis				c120ACP + 2 h + malACP + nadph + h -> 3hmrsACP + ACP + co2 + nadp	c120ACP + 2 h + malACP + nadph -> 3hmrsACP + ACP + co2 + nadp	c120ACP + 2 h + malACP + nadph -> 3hmrsACP + ACP + co2 + nadp
FALDHD	Methane Metabolism	formaldehyde dehydrogenase	1.1.1.284	H16_B1195	adhC	hmgth + nad <-> fmgt + h + nadh	hmgth + nad <-> fmgt + h + nadh	hmgth + nad <-> fmgt + h + nadh
2KMBT	Methionine Metabolism	2-keto-4-methylthiobutyrate transamination	2.6.1.57	H16_A1151/H16_B1081	tyrB1/tyrB2	2kmb + glu -> akg + met	2kmb + glu -> akg + met	2kmb + glu -> akg + met
METS	Methionine Metabolism	methionine synthase	2.1.1.13	H16_A0151	metH	mthf + hcys -> met + thf	mthf + hcys -> met + thf	mthf + hcys -> met + thf
METADT	Methionine Metabolism	methionine adenosyltransferase	2.5.1.6	H16_A0230/H16_A1975	metK1/metK2	atp + h2o + met + h -> sam + pi + ppi	atp + h2o + met -> sam + pi + ppi + h	atp + h2o + met -> sam + pi + ppi + 2 h
ADHCYSNS	Methionine Metabolism	S-adenosylhomocysteine nucleosidase	3.2.2.9	H16_A3337	pfs	sah + h2o -> ad + srlh	sah + h2o -> ad + srlh	sah + h2o + h -> ad + srlh
CYTTS1	Methionine Metabolism	cystathionine gamma-synthase	2.5.1.48	H16_A2606	metB	cys + oslhser -> llct + h + succ	cys + oslhser -> llct + h + succ	cys + oslhser -> llct + h + succ
CYSTBL1	Methionine Metabolism	cystathionine b-lyase	4.4.1.8	H16_A1447	metC	llct + h2o -> hcys + nh4 + pyr	llct + h2o -> hcys + nh4 + pyr	llct + h2o -> hcys + nh4 + pyr
LAAO2	Methionine metabolism	L-amino-acid oxidase	1.4.3.2	H16_A0845/H16_A0856	lao1/lao2	met + h2o + o2 -> 2kmb + nh4 + h2o2	met + h2o + o2 -> 2kmb + nh4 + h2o2	met + h2o + o2 -> 2kmb + nh4 + h2o2
ADHC1	Methionine metabolism	adenosylhomocysteinase	3.3.1.1	H16_A0244	ahcY	sah + h2o <-> adn + hcys	sah + h2o <-> adn + hcys	sah + h2o <-> adn + hcys + h
CYTTS2	Methionine metabolism	cystathionine gamma-synthase	2.5.1.48	H16_A2606	metB	oslhser + h2o <-> obut + succ + nh4 + h	oslhser + h2o <-> obut + succ + nh4 + h	oslhser + h2o <-> obut + succ + nh4 + h
CYTTS3	Methionine metabolism	cystathionine gamma-synthase	2.5.1.48	H16_A2606	metB	oahser + cys <-> llct + ac + h	oahser + cys <-> llct + ac + h	oahser + cys <-> llct + ac + h
CYTTS4	Methionine metabolism	cystathionine gamma-synthase	2.5.1.48	H16_A2606	metB	oahser + h2s -> hcys + ac + h	oahser + h2s -> hcys + ac	oahser + h2s -> hcys + ac
OAHSL1	Methionine metabolism	O-acetylhomoserine (thiol)-lyase	2.5.1.49	H16_A1313/H16_B2229	metY1/metY2	oahser + tsul + rthio -> hcys + so3 + othio + ac + h	oahser + tsul + rthio -> hcys + so3 + othio + ac + h	oahser + tsul + rthio -> hcys + so3 + othio + ac + h
CYTTS5	Methionine metabolism	cystathionine gamma-synthase	2.5.1.48	H16_A2606	metB	oslhser + h2s <-> hcys + succ + h	oslhser + h2s <-> hcys + succ	oslhser + h2s <-> hcys + succ
HSERA	Methionine metabolism	homoserine O-acetyltransferase	2.3.1.31	H16_A0211	metX	accoa + hser <-> coa + oahser	accoa + hser <-> coa + oahser	accoa + hser <-> coa + oahser
MTTGH	Methionine metabolism	5-methyltetrahydropteroyltriglutamate--homocysteine methyltransferase	2.1.1.14	H16_B1581	metE	5mtglu + hcys -> tglu + met	5mtglu + hcys -> tglu + met	5mtglu + hcys -> tglu + met
NTPPD6	Naphthalene and Anthracene degradation	2-nitropropane dioxygenase	1.13.11.-	H16_A0633/H16_B0223/H16_B0757/H16_B1109/H16_B1420/H16_B1836	pcaH2/unknown/unknown/unknown/unknown/unknown	phentrc + nadh + h + o2 -> c34dhphe + nad	phentrc + nadh + h + o2 -> c34dhphe + nad	phentrc + nadh + h + o2 -> c34dhphe + nad
FBMO12	Naphthalene and Anthracene degradation	chlorophenol 4-monooxygenase	1.14.13.-	H16_A1145/H16_B0495/H16_B1480/H16_B2135	unknown/unknown/unknown/unknown	phentrc + o2 + nadh + h -> pheth12o + h2o + nad	phentrc + o2 + nadh + h -> pheth12o + h2o + nad	phentrc + o2 + nadh + h -> pheth12o + h2o + nad
FBMO13	Naphthalene and Anthracene degradation	chlorophenol 4-monooxygenase	1.14.13.-	H16_A1145/H16_B0495/H16_B1480/H16_B2135	unknown/unknown/unknown/unknown	phentrc + o2 + nadh + h -> phatol + h2o + nad	phentrc + o2 + nadh + h -> phatol + h2o + nad	phentrc + o2 + nadh + h -> phatol + h2o + nad
FBMO14	Naphthalene and Anthracene degradation	chlorophenol 4-monooxygenase	1.14.13.-	H16_A1145/H16_B0495/H16_B1480/H16_B2135	unknown/unknown/unknown/unknown	phentrc + o2 + nadh + h -> pheth910o + h2o + nad	phentrc + o2 + nadh + h -> pheth910o + h2o + nad	phentrc + o2 + nadh + h -> pheth910o + h2o + nad
SALCH1	Naphthalene and Anthracene degradation	salicylate hydroxylase	1.14.13.1	H16_A0578/H16_A0922/H16_A1785/H16_B0750/H16_B0876	unknown/unknown/unknown/unknown/unknown	hnaptho + nadh + o2 + 2 h -> napth12d + co2 + nad + h2o	hnaptho + nadh + o2 + 2 h -> napth12d + co2 + nad + h2o	hnaptho + nadh + o2 + 2 h -> napth12d + co2 + nad + h2o
NTPPD7	Naphthalene and Anthracene degradation	2-nitropropane dioxygenase	1.13.11.-	H16_A0633/H16_B0223/H16_B0757/H16_B1109/H16_B1420/H16_B1836	pcaH2/unknown/unknown/unknown/unknown/unknown	12anthcd + o2 -> carvnapc + 2 h	12anthcd + o2 -> carvnapc + 2 h	12anthcd + o2 -> carvnapc + 2 h
NTPPD8	Naphthalene and Anthracene degradation	2-nitropropane dioxygenase	1.13.11.-	H16_A0633/H16_B0223/H16_B0757/H16_B1109/H16_B1420/H16_B1836	pcaH2/unknown/unknown/unknown/unknown/unknown	12anthcd + o2 -> hnapoxen + h	12anthcd + o2 -> hnapoxen + h	12anthcd + o2 -> hnapoxen + h
NTPPD9	Naphthalene and Anthracene degradation	2-nitropropane dioxygenase	1.13.11.-	H16_A0633/H16_B0223/H16_B0757/H16_B1109/H16_B1420/H16_B1836	pcaH2/unknown/unknown/unknown/unknown/unknown	3h2napth + o2 -> cmcdopp + h	3h2napth + o2 -> cmcdopp + h	3h2napth + o2 -> cmcdopp + h
NTPPD10	Naphthalene and Anthracene degradation	2-nitropropane dioxygenase	1.13.11.-	H16_A0633/H16_B0223/H16_B0757/H16_B1109/H16_B1420/H16_B1836	pcaH2/unknown/unknown/unknown/unknown/unknown	dhnapthsul + o2 -> hsulpob + h	dhnapthsul + o2 -> hsulpob + h	dhnapthsul + o2 -> hsulpob + h

FBMO15	Naphthalene and Anthracene degradation	chlorophenol 4-monooxygenase	1.14.13.-	H16_A1145/H16_B0495/H16_B1480/H16_B2135	unknown/unknown/unknown/unknown	salcyl + nadh + o2 + h -> gensa + nad + h2o	salcyl + nadh + o2 + h -> gensa + nad + h2o	salcyl + nadh + o2 + h -> gensa + nad + h2o
FBMO15p	Naphthalene and Anthracene degradation	chlorophenol 4-monooxygenase	1.14.13.-	H16_A1145/H16_B0495/H16_B1480/H16_B2135	unknown/unknown/unknown/unknown	salcyl + nadph + o2 + 2 h -> gensa + nadp + h2o	salcyl + nadph + o2 + h -> gensa + nadp + h2o	salcyl + nadph + o2 + h -> gensa + nadp + h2o
SALCH2	Naphthalene and Anthracene degradation	salicylate hydroxylase	1.14.13.1	H16_A0578/H16_A0922/H16_A1785/H16_B0750/H16_B0876	unknown/unknown/unknown/unknown/unknown	salcyl + o2 + nadh + 2 h -> catech + co2 + nad + h2o	salcyl + o2 + nadh + 2 h -> catech + co2 + nad + h2o	salcyl + o2 + nadh + 2 h -> catech + co2 + nad + h2o
ANOXDR	Naphthalene and Anthracene degradation	Oxidoreductase	1.14.-.-	H16_B0730/H16_B0738/H16_B2129	unknown/unknown/unknown	aniline + o2 + h + h2 -> catech + nh4	aniline + o2 + h + h2 -> catech + nh4	aniline + o2 + h + h2 -> catech + nh4
NITOXDR	Naphthalene and Anthracene degradation	Oxidoreductase	1.14.-.-	H16_B0730/H16_B0738/H16_B2129	unknown/unknown/unknown	nitbz + o2 + h2 -> catech + no2 + h	nitbz + o2 + h2 -> catech + no2 + h	nitbz + o2 + h2 -> catech + no2 + h
PHE2MO5	Naphthalene and Anthracene degradation	phenol 2-monooxygenase	1.14.13.7	H16_B0539&H16_B0540&H16_B0541&H16_B0542&H16_B0543&H16_B0544	poxA&poxB&poxC&poxD&poxE&poxF	phenol + o2 + nadph + 2 h -> catech + nadp + h2o	phenol + o2 + nadph + h -> catech + nadp + h2o	phenol + o2 + nadph + h -> catech + nadp + h2o
PHOXDR	Naphthalene and Anthracene degradation	Oxidoreductase	1.14.-.-	H16_B0730/H16_B0738/H16_B2129	unknown/unknown/unknown	pheborn + o2 + 2.5 h2 -> phenol + 3 h2o	pheborn + o2 + 2.5 h2 -> phenol + 3 h2o	pheborn + o2 + 2.5 h2 -> phenol + 3 h2o
ANTOXDR	Naphthalene and Anthracene degradation	Oxidoreductase	1.14.-.-	H16_B0730/H16_B0738/H16_B2129	unknown/unknown/unknown	anthrc + o2 + h2 -> anthr910d	anthrc + o2 + h2 -> anthr910d	anthrc + o2 + h2 -> anthr910d
NACMNP	Nicotinate and Nicotinamide metabolism	nicotinic acid mononucleotide pyrophosphorylase	2.4.2.11	H16_A2589	pncB	atp + h2o + nac + prpp + h -> adp + nacn + pi + ppi	nac + prpp -> nacn + ppi	atp + h2o + nac + prpp -> adp + nacn + pi + ppi + h
NACNDP	Nicotinate and Nicotinamide metabolism	nicotinate-nucleotide diphosphorylase (carboxylating)	2.4.2.19	H16_A3037/H16_B0560	nadC/unknown	2 h + prpp + qa <-> co2 + nacn + ppi	h + prpp + qa <-> co2 + nacn + ppi	h + prpp + qa <-> co2 + nacn + ppi
NADK	Nicotinate and Nicotinamide metabolism	NAD kinase	2.7.1.23	H16_A1132/H16_B0143	unknown/acoX	atp + nad -> adp + nadp	atp + nad -> adp + nadp + h	atp + nad -> adp + nadp + h
NAMNAT	Nicotinate and Nicotinamide metabolism	nicotinamide-nucleotide adenylyltransferase	2.7.7.18	H16_A0913	nadD	atp + namn -> nad + ppi	atp + namn -> nad + ppi	atp + namn -> nad + ppi
NACM	Nicotinate and Nicotinamide metabolism	nicotinamidase	3.5.1.19	H16_A1527	pncA	h2o + nam -> nac + nh4	h2o + nam -> nac + nh4	h2o + nam -> nac + nh4
NADDP1	Nicotinate and Nicotinamide metabolism	NAD diphosphatase	3.6.1.22	H16_A2761	unknown	h2o + nad -> amp + namn	h2o + nad -> amp + namn + 2 h	h2o + nad -> amp + namn + 2 h
ASPOX2	Nicotinate and Nicotinamide metabolism	L-aspartate oxidase	1.4.3.16	H16_A3036	nadB	asp + o2 <-> h + h2o2 + iasp	asp + o2 <-> h + h2o2 + iasp	asp + o2 <-> h + h2o2 + iasp
QULS	Nicotinate and Nicotinamide metabolism	quinolinate synthase		H16_A3038	nadA (2.5.1.72)	dhap + iasp <-> 2 h2o + pi + qa	dhap + iasp <-> 2 h2o + pi + qa	dhap + iasp <-> 2 h2o + pi + qa
NTNAT	Nicotinate and Nicotinamide metabolism	nicotinate-nucleotide adenylyltransferase	2.7.7.18	H16_A0913	nadD	atp + nacn <-> dnad + ppi + h	atp + nacn <-> dnad + ppi + h	atp + nacn <-> dnad + ppi + h
NTD13	Nicotinate and Nicotinamide metabolism	5'-nucleotidase	3.1.3.5	H16_A2376	surE	namn + h2o -> namd + pi	namn + h2o -> namd + pi	namn + h2o -> namd + pi
NTD14	Nicotinate and Nicotinamide metabolism	5'-nucleotidase	3.1.3.5	H16_A2376	surE	nacn + h2o -> nacd + pi	nacn + h2o -> nacd + pi	nacn + h2o -> nacd + pi
NADDP2	Nicotinate and Nicotinamide metabolism	NAD diphosphatase	3.6.1.22	H16_A2761	unknown	dnad + h2o + h -> amp + nacn	dnad + h2o -> amp + nacn + h	dnad + h2o -> amp + nacn + h
NODOX1	Nitrogen Metabolism	nitric oxide dioxygenase	1.14.12.17	H16_A3533	hmp2	nadh + 2 no + 2 o2 -> h + nad + 2 no3	nadh + 2 no + 2 o2 -> h + nad + 2 no3	nadh + 2 no + 2 o2 -> h + nad + 2 no3
NODOX2	Nitrogen Metabolism	nitric oxide dioxygenase	1.14.12.17	H16_A3533	hmp2	nadph + 2 no + 2 o2 -> nadp + 2 no3	nadph + 2 no + 2 o2 -> nadp + 2 no3 + h	nadph + 2 no + 2 o2 -> nadp + 2 no3 + h
NO3RUQ1	Nitrogen Metabolism	Nitrate reductase (Ubiquinol-8)	1.7.99.4	H16_B0776/H16_B2265/H16_B2266/H16_B2267/H16_B2268	unknown/narG2/narH2/narJ2/narI2	2 h + no3 + uqh2 -> 2 h_e + h2o + no2 + uq	no3 + uqh2 + 2 h -> h2o + no2 + uq + 2 h_e	2 h + no3 + uqh2 -> 2 h_e + h2o + no2 + uq
CYNTL	Nitrogen Metabolism	cyanate lyase	4.2.1.104	H16_B0046	cynS	cynt + hco3 -> co2 + cabm	cynt + h + hco3 -> co2 + cabm	cynt + h + hco3 -> co2 + cabm
NADFRBO	Nitrogen Metabolism	NADH:flavorubredoxin oxidoreductase	1.7.99.7	H16_B2323	norB2	h + nadh + 2 no -> h2o + n2o + nad	h + nadh + 2 no -> h2o + n2o + nad	h + nadh + 2 no -> h2o + n2o + nad
CNITRT	Nitrogen metabolism	cytochrome cd1 nitrite reductase (NirS)	1.7.2.1	H16_B2277	nirS	no2 + fadh2 + h -> no + h2o + fad	no2 + fadh2 + h -> no + h2o + fad	no2 + fadh2 + h -> no + h2o + fad
NITRT	Nitrogen metabolism	nitrite reductase	1.7.1.4	H16_B0777&H16_B0778&H16_B0779&H16_B0948	unknown&nasE&nasD&unknown	no2 + 3 nadh + 5 h -> 3 nad + nh4 + 2 h2o	no2 + 3 nadh + 5 h -> 3 nad + nh4 + 2 h2o	no2 + 3 nadh + 5 h -> 3 nad + nh4 + 2 h2o
NITORT	Nitrogen metabolism	nitrous-oxide reductase	1.7.99.6	PHG252	nosZ	n2o + fadh2 -> n2 + fad + h2o	n2o + fadh2 -> n2 + fad + h2o + h	n2o + fadh2 -> n2 + fad + h2o + h
ADNK3	Nucleotide Salvage Pathway	adentylate kinase (GTP)	2.7.4.3	H16_A0603	adk	amp + gtp <-> adp + gdp	amp + gtp + h <-> adp + gdp	amp + gtp <-> adp + gdp
ADNK4	Nucleotide Salvage Pathway	adentylate kinase (ITP)	2.7.4.3	H16_A0603	adk	amp + itp <-> adp + idp	amp + itp + h <-> adp + idp	amp + itp <-> adp + idp
TDPDRHR	Nucleotide sugars metabolism	dTDP-4-dehydrorhamnose reductase	1.1.1.133	H16_A1850/H16_A2908	rfbD/unknown	dtdp4d6dm + 2 h + nadph -> dtdprmn + nadp	dtdp4d6dm + h + nadph -> dtdprmn + nadp	dtdp4d6dm + h + nadph -> dtdprmn + nadp
UDPG6D	Nucleotide sugars metabolism	UDPglucose 6-dehydrogenase	1.1.1.22	H16_A0802	ugd	h2o + 2 nad + udpg -> 3 h + 2 nadh + udpglcur	h2o + 2 nad + udpg -> 3 h + 2 nadh + udpglcur	h2o + 2 nad + udpg -> 3 h + 2 nadh + udpglcur
G1PTT1	Nucleotide sugars metabolism	glucose-1-phosphate thymidylyltransferase	2.7.7.24	H16_A1864	rfbA	dttp + g1p -> dtdpglu + ppi	dttp + g1p -> dtdpglu + ppi	dttp + g1p -> dtdpglu + ppi
UG1PUT	Nucleotide sugars metabolism	UTP-glucose-1-phosphate uridylyltransferase (irreversible)	2.7.7.9	H16_A2752	galU	g1p + utp -> ppi + udpg	g1p + utp -> ppi + udpg	g1p + utp -> ppi + udpg
TDPGLUD	Nucleotide sugars metabolism	dTDPglucose 4,6-dehydratase	4.2.1.46	H16_A1851/H16_A2909/H16_B1642	rfbB2/unknown/unknown	dtdpglu -> dtdp4d6dg + h2o	dtdpglu -> dtdp4d6dg + h2o	dtdpglu -> dtdp4d6dg + h2o
TDPDRHE	Nucleotide sugars metabolism	dTDP-4-dehydrorhamnose 3,5-epimerase	5.1.3.13	H16_A1848/H16_A2906	rfbC/unknown	dtdp4d6dg -> dtdp4d6dm	dtdp4d6dg -> dtdp4d6dm	dtdp4d6dg -> dtdp4d6dm
G1PTT2	Nucleotide sugars metabolism	glucose-1-phosphate thymidylyltransferase	2.7.7.33	H16_A2893	rfbF	dttp + g1p -> dtdpglu + ppi	dttp + g1p -> dtdpglu + ppi	dttp + g1p -> dtdpglu + ppi
UDPG4E1	Nucleotide sugars metabolism	UDP-glucose 4-epimerase	5.1.3.2	H16_B0226/H16_B0283	galE/unknown	dtdpglu <-> dtdpgal	dtdpglu <-> dtdpgal	dtdpglu <-> dtdpgal
UDPG4E2	Nucleotide sugars metabolism	UDP-glucose 4-epimerase	5.1.3.2	H16_B0226/H16_B0283	galE/unknown	udpg <-> udpgal	udpg <-> udpgal	udpg <-> udpgal
AMTF2	One carbon pool by Folate	aminomethyltransferase	2.1.2.10	H16_A1567/H16_A3619	gcvT2/gcvT1	methf + h2o -> 5fthf + h	methf + h2o -> 5fthf + h	methf + h2o -> 5fthf + h
FORTF	One carbon pool by Folate	5-formyltetrahydrofolate cyclo-ligase	6.3.3.2	H16_A0249	unknown	5fthf + atp + h -> adp + pi + methf	5fthf + atp -> adp + pi + methf	5fthf + atp -> adp + pi + methf
LACDHq	Oxidative Phosphorylation	L-Lactate dehydrogenase (ubiquinone)	1.1.2.3	H16_B0460/H16_B1817	lldA/lldD	llac + uq -> pyr + uqh2	llac + uq -> pyr + uqh2	llac + uq -> pyr + uqh2
DLDHDq	Oxidative Phosphorylation	D-lactate dehydrogenase	1.1.2.4	H16_A3091	dld	lac + uq -> pyr + uqh2	lac + uq -> pyr + uqh2	lac + uq -> pyr + uqh2
GL3PDq	Oxidative Phosphorylation	glycerol-3-phosphate dehydrogenase (ubiquinone-8)	1.1.99.5	H16_A2508/H16_B1198	unknown/unknown	glyc3p + uq -> dhap + uqh2	glyc3p + uq -> dhap + uqh2	glyc3p + uq -> dhap + uqh2

HYDGq	Oxidative Phosphorylation	hydrogenase (ubiquinone-8: 2 protons)	1.18.99.1			2 h + h2 + uq -> 2 h_e + uqh2	2 h + uq + h2 -> uqh2 + 2 h_e	2 h + h2 + uq -> 2 h_e + uqh2
FDHGq	Oxidative Phosphorylation	formate dehydrogenase (quinone-8)	1.2.2.1			formate + h + uq -> co2 + uqh2	formate + 3 h + uq -> co2 + uqh2 + 2 h_e	formate + h + uq -> co2 + uqh2
POX	Oxidative Phosphorylation	pyruvate oxidase	1.2.2.2	H16_A3123	unknown	h2o + pyr + uq -> ac + co2 + uqh2	h2o + pyr + uq -> ac + co2 + uqh2	h2o + pyr + uq -> ac + co2 + uqh2
SUCCD1	Oxidative Phosphorylation	succinate dehydrogenase	1.3.99.1	H16_A2629&H16_A2630&H16_A2631&H16_A2632&H16_B0204	sdhB&sdhA&sdhD&sdhC&unknown	uq + succ -> fum + uqh2	uq + succ -> fum + uqh2	uq + succ -> fum + uqh2
NADPTH	Oxidative Phosphorylation	NAD(P) transhydrogenase	1.6.1.2	H16_A0850&H16_A0851&H16_A0852&H16_A1264&H16_A1265&H16_A1266&H16_A3128&H16_A3130&H16_A3131&H16_B1714&H16_B1715	pntAa1&pntAb1&pntB1&pntAa2&pntAb2&pntB2&pntAa3&pntB3&pntB4&pntA4	2 h_e + nadh + nadp -> 3 h + nad + nadph	nadh + nadp + 2 h_e -> 2 h + nad + nadph	2 h_e + nadh + nadp -> 2 h + nad + nadph
NADHHq1	Oxidative Phosphorylation	NADH dehydrogenase (ubiquinone-8 & 3 protons)	1.6.5.3	H16_A0251/H16_A1050/H16_A1051/H16_A1052/H16_A1053/H16_A1054/H16_A1055/H16_A1056/H16_A1057/H16_A1058/H16_A1059/H16_A1060/H16_A1061/H16_A1062/H16_A1063/	unknown/nuoA/nuoB/nuoC/nuoD/nuoE/nuoF/nuoG/nuoH/nuoI/nuoJ/nuoK/nuoL/nuoM/nuoN/	4 h + nadh + uq -> 3 h_e + nad + uqh2	4 h + nadh + uq -> nad + uqh2 + 3 h_e	4 h + nadh + uq -> 3 h_e + nad + uqh2
NADHHq2	Oxidative Phosphorylation	NADH dehydrogenase (ubiquinone-8)	1.6.5.3	H16_A0251/H16_A1050/H16_A1051/H16_A1052/H16_A1053/H16_A1054/H16_A1055/H16_A1056/H16_A1057/H16_A1058/H16_A1059/H16_A1060/H16_A1061/H16_A1062/H16_A1063/	unknown/nuoA/nuoB/nuoC/nuoD/nuoE/nuoF/nuoG/nuoH/nuoI/nuoJ/nuoK/nuoL/nuoM/nuoN/	h + nadh + uq -> nad + uqh2	h + nadh + uq -> nad + uqh2	h + nadh + uq -> nad + uqh2
NO3RUQ2	Oxidative Phosphorylation	Nitrate reductase (Ubiquinol-8)	1.7.99.4	H16_B0776/H16_B2265/H16_B2266/H16_B2267/H16_B2268	unknown/narG2/narH2/narJ2/narI2	2 h + no3 + uqh2 -> 2 h_e + h2o + no2 + uq	2 h + no3 + uqh2 -> h2o + no2 + uq + 2 h_e	2 h + no3 + uqh2 -> 2 h_e + h2o + no2 + uq
THIORp	Oxidative Phosphorylation	thioredoxin reductase (NADPH)	1.8.1.9	H16_A0753/H16_A1199/H16_A1779/H16_A2592/H16_B1092/H16_B1422	unknown/trxB1/unknown/trxB2/unknown/unknown	2 h + nadph + othio -> nadp + rthio	h + nadph + othio -> nadp + rthio	h + nadph + othio -> nadp + rthio
ATPSYN	Oxidative Phosphorylation	ATP synthase (four protons for one ATP)	3.6.3.14	H16_A3636&H16_A3637&H16_A3638&H16_A3639&H16_A3640&H16_A3641&H16_A3642&H16_A3643&H16_B2371	atpC&atpD&atpG&atpA&atpH&atpF&atpE&atpB&fliI	adp + 4 h_e + pi <-> atp + 4 h + h2o	adp + pi + 4 h_e <-> atp + 3 h + h2o	adp + 4 h_e + pi <-> atp + 3 h + h2o
BTCRNCT	Oxidative Phosphorylation	gamma-butyrobetainyl-CoA: carnitine CoA transferase		H16_A2078/H16_B2438	unknown/unknown	bbtcoa + crn <-> crncoa + gbbtn	bbtcoa + crn <-> crncoa + gbbtn	bbtcoa + crn <-> crncoa + gbbtn
CTBTCRNCT	Oxidative Phosphorylation	crotonobetainyl-CoA: carnitine CoA transferase		H16_A2078/H16_B2438	unknown/unknown	crn + ctbtcoa <-> crncoa + ctbt	crn + ctbtcoa <-> crncoa + ctbt	crn + ctbtcoa <-> crncoa + ctbt
CRNCDH	Oxidative Phosphorylation	Carnityl-CoA dehydratse		H16_A2078/H16_B2438	unknown/unknown	crncoa <-> ctbtcoa + h2o	crncoa <-> ctbtcoa + h2o	crncoa <-> ctbtcoa + h2o
CYTCOBD	Oxidative Phosphorylation	cytochrome oxidase bd (ubiquinol-8: 2 protons)				2 h + 0.5 o2 + uqh2 -> 2 h_e + h2o + uq	2 h + 0.5 o2 + uqh2 -> h2o + uq + 2 h_e	2 h + 0.5 o2 + uqh2 -> 2 h_e + h2o + uq
CYTCOBO3	Oxidative Phosphorylation	cytochrome oxidase bo3 (ubiquinol-8: 4 protons)				4 h + 0.5 o2 + uqh2 -> 4 h_e + h2o + uq	4 h + 0.5 o2 + uqh2 -> h2o + uq + 4 h_e	4 h + 0.5 o2 + uqh2 -> 4 h_e + h2o + uq
NADTRHG	Oxidative Phosphorylation	NAD transhydrogenase				h + nad + nadph -> nadh + nadp	nad + nadph -> nadh + nadp	nad + nadph -> nadh + nadp
NITRR	Oxidative Phosphorylation	nitrite Reductase (NADH)				5 h + 3 nadh + no2 -> 2 h2o + 3 nad + nh4	5 h + 3 nadh + no2 -> 2 h2o + 3 nad + nh4	5 h + 3 nadh + no2 -> 2 h2o + 3 nad + nh4
SUCCD2	Oxidative phosphorylation	succinate dehydrogenase	EC-Undetermined			fadh2 + uq -> fad + uqh2	fadh2 + uq -> fad + uqh2 + h	fadh2 + uq -> fad + uqh2 + h
DP2R	Pantothenate and CoA biosynthesis	2-dehydropantoate 2-reductase	1.1.1.169	H16_A1715/H16_B1719/H16_B1769	apbA1/apbA2/apbA3	dhpant + 2 h + nadph -> nadp + pant	dhpant + h + nadph -> nadp + pant	dhpant + h + nadph -> nadp + pant
MOBHMT	Pantothenate and CoA biosynthesis	3-methyl-2-oxobutanoate hydroxymethyltransferase	2.1.2.11	H16_A3084	panB	3mob + h2o + metthf -> dhpant + thf	3mob + h2o + metthf -> dhpant + thf	3mob + h2o + metthf -> dhpant + thf
DPCOAK	Pantothenate and CoA biosynthesis	dephospho-CoA kinase	2.7.1.24	H16_A3258	coaE	atp + dpcoa -> adp + coa	atp + dpcoa -> adp + coa + h	atp + dpcoa -> adp + coa + h
PNTOK1	Pantothenate and CoA biosynthesis	pantothenate kinase	2.7.1.33	H16_A0136	unknown	atp + pnto -> 4ppnto + adp	atp + pnto -> 4ppnto + adp + h	atp + pnto -> 4ppnto + adp + h
PTHPAT	Pantothenate and CoA biosynthesis	pantetheine-phosphate adenylyltransferase	2.7.7.3	H16_A0367	unknown	atp + 4ppnte -> dpcoa + ppi	atp + 4ppnte -> dpcoa + ppi	atp + 4ppnte -> dpcoa + ppi
ACPS	Pantothenate and CoA biosynthesis	acyl-carrier protein synthase	2.7.8.7	H16_A2551	acpS	apoACP + coa -> ACP + pap	apoACP + coa -> ACP + pap + h	apoACP + coa -> ACP + pap + h
PPTCDC	Pantothenate and CoA biosynthesis	phosphopantothenoylcysteine decarboxylase	4.1.1.36	H16_A3048	dfp	4ppcys + h -> co2 + 4ppnte	4ppcys + h -> co2 + 4ppnte	4ppcys + h -> co2 + 4ppnte
PANTOS	Pantothenate and CoA biosynthesis	pantothenate synthase	6.3.2.1	H16_A2959	unknown	bala + atp + pant -> amp + pnto + ppi	bala + atp + pant -> amp + pnto + ppi + 2 h	bala + atp + pant -> amp + pnto + ppi + 2 h
PPNTCL	Pantothenate and CoA biosynthesis	phosphopantothenate-cysteine ligase	6.3.2.5	H16_A3048	dfp	4ppnto + ctp + cys -> 4ppcys + cmp + ppi	4ppnto + ctp + cys -> 4ppcys + cmp + ppi + 2 h	4ppnto + ctp + cys -> 4ppcys + cmp + ppi + 2 h
PNTOK2	Pantothenate and Coa Biosynthesis	pantothenate kinase	2.7.1.33	H16_A0136	unknown	atp + pantcys -> adp + 4ppcys	atp + pantcys -> adp + 4ppcys + h	atp + pantcys -> adp + 4ppcys + h
PNTOK3	Pantothenate and Coa Biosynthesis	pantothenate kinase	2.7.1.33	H16_A0136	unknown	atp + ptt -> adp + 4ppnte	atp + ptt -> adp + 4ppnte + h	atp + ptt -> adp + 4ppnte + h
ALTRNH	Pentose and glucuronate interconversion	altronate hydrolase	4.2.1.7	H16_A2758/H16_B0321	unknown/unknown	dalt -> kdg + h2o	dalt -> kdg + h2o	dalt -> kdg + h2o
ALRTx	Pentose and glucuronate interconversion	aldehyde reductase	1.1.1.21	H16_A3186	unknown	xylt + nadp <-> xyl + nadph + 2 h	xylt + nadp <-> xyl + nadph + h	xylt + nadp <-> xyl + nadph + h
ALRTap	Pentose and glucuronate interconversion	aldehyde reductase	1.1.1.21	H16_A3186	unknown	arbt + nadp <-> larabinose + nadph + 2 h	arbt + nadp <-> larabinose + nadph + h	arbt + nadp <-> larabinose + nadph + h
ALRTa	Pentose and glucuronate interconversion	aldehyde reductase	1.1.1.21	H16_A3186	unknown	arbt + nad <-> larabinose + nadh + h	arbt + nad <-> larabinose + nadh + h	arbt + nad <-> larabinose + nadh + h
GLCNK	Pentose Phosphate Pathway	gluconokinase	2.7.1.12	H16_A1179	gntK	atp + gluc -> d6pgc + adp	atp + gluc -> d6pgc + adp + h	atp + gluc -> d6pgc + adp + h
DDGLCNK	Pentose Phosphate Pathway	2-dehydro-3-deoxygluconokinase	2.7.1.45	H16_B1212	kdgK	kdg + atp -> kdpg + adp	kdg + atp -> kdpg + adp + h	kdg + atp -> kdpg + adp + h
AB5PI	Pentose Phosphate Pathway	arabinose-5-phosphate isomerase	5.3.1.13	H16_A0391	gutQ	rl5p <-> a5p	rl5p <-> a5p	rl5p <-> a5p
R15BPK	Pentose Phosphate Pathway	ribose-1,5-bisphosphokinase	2.7.4.23	H16_B1291	phnN	atp + r15bp -> adp + prpp	atp + r15bp + h -> adp + prpp	atp + r15bp -> adp + prpp
PRPPS	Pentose Phosphate Pathway	phosphoribosylpyrophosphate synthetase	2.7.6.1	H16_A0372	prsA	atp + r5p <-> amp + prpp	atp + r5p <-> amp + prpp + h	atp + r5p <-> amp + prpp + h
PPG2DG	Pentose Phosphate Pathway	phosphogluconate 2-dehydrogenase	1.1.1.43	H16_B1813	kguD	d6pgc + nad -> 2dhglcn6p + nadh + h	d6pgc + nad -> 2dhglcn6p + nadh + h	d6pgc + nad -> 2dhglcn6p + nadh + h
G6PDH	Pentose Phosphate Pathway	glucose 6-phosphate dehydrogenase	1.1.1.49	H16_A0316/H16_B1501/H16_B2566	zwf1/zwf2/zwf3	g6p + nadp <-> 6pgl + 2 h + nadph	g6p + nadp <-> 6pgl + h + nadph	g6p + nadp <-> 6pgl + h + nadph

TRKT1	Pentose Phosphate Pathway	transketolase	2.2.1.1	H16_A3147/H16_B1388	tktA/cbbT2	r5p + xu5p <-> g3p + s7p	r5p + xu5p <-> g3p + s7p	r5p + xu5p <-> g3p + s7p
TRKT2	Pentose Phosphate Pathway	transketolase	2.2.1.1	H16_A3147/H16_B1388	tktA/cbbT2	e4p + xu5p <-> f6p + g3p	e4p + xu5p <-> f6p + g3p	e4p + xu5p <-> f6p + g3p
TRADL	Pentose Phosphate Pathway	transaldolase	2.2.1.2	H16_A2346	tal	g3p + s7p <-> e4p + f6p	g3p + s7p <-> e4p + f6p	g3p + s7p <-> e4p + f6p
PGL	Pentose Phosphate Pathway	6-phosphogluconolactonase	3.1.1.31	H16_B2565	pgl	6pgl + h2o -> d6pgc + h	6pgl + h2o -> d6pgc + h	6pgl + h2o -> d6pgc + h
EDA	Pentose Phosphate Pathway	2-dehydro-3-deoxy-phosphogluconate aldolase	4.1.2.14	H16_B1213	eda	kdpg -> g3p + pyr	kdpg -> g3p + pyr	kdpg -> g3p + pyr
EDD	Pentose Phosphate Pathway	6-phosphogluconate dehydratase	4.2.1.12	H16_A1178/H16_B2567	edd1/edd2	d6pgc -> kdpg + h2o	d6pgc -> kdpg + h2o	d6pgc -> kdpg + h2o
RPE	Pentose Phosphate Pathway	ribulose 5-phosphate 3-epimerase	5.1.3.1	H16_A3317/H16_B1391	rpe/cbbE2	rl5p <-> xu5p	rl5p <-> xu5p	rl5p <-> xu5p
RPI	Pentose Phosphate Pathway	ribose-5-phosphate isomerase	5.3.1.6	H16_A2345	rpiA	r5p <-> rl5p	r5p <-> rl5p	r5p <-> rl5p
GLTN2	Pentose Phosphate Pathway	gluconolactonase	3.1.1.17	H16_A3012/H16_B0345/H16_B1441	gnl1/gnl2/gnl3	g15l + h2o -> gluc + h	g15l + h2o -> gluc + h	g15l + h2o -> gluc + h
PPG2DGp	Pentose Phosphate Pathway	phosphogluconate 2-dehydrogenase	1.1.1.43	H16_B1813	kguD	d6pgc + nadp -> 2dhglcn6p + nadph + 2 h	d6pgc + nadp -> 2dhglcn6p + nadph + h	d6pgc + nadp -> 2dhglcn6p + nadph + h
PAMPPT	Peptidoglycan Biosynthesis	phospho-N-acetylmuramoyl-pentapeptide-transferase (meso-2,6-diaminopimelate)	2.7.8.13	H16_A3276	mraY	udcpp + ugmda -> uagmda + ump	udcpp + ugmda -> uagmda + ump	udcpp + ugmda -> uagmda + ump
UDCPDP	Peptidoglycan Biosynthesis	undecaprenyl-diphosphatase	3.6.1.27	H16_A2871	bacA	h2o + udcpdp -> pi + udcpp	h2o + udcpdp -> 2 h + pi + udcpp	h2o + udcpdp -> h + pi + udcpp
UAMAGDS	Peptidoglycan Biosynthesis	UDP-N-acetylmuramoyl-L-alanyl-D-glutamyl-meso-2,6-diaminopimelate synthetase	6.3.2.13	H16_A3278	murE	26dap-M + atp + uamag -> adp + pi + ugmd	26dap-M + atp + uamag -> adp + h + pi + ugmd	26dap-M + atp + uamag -> adp + 2 h + pi + ugmd
UAMAGDAS	Peptidoglycan Biosynthesis	UDP-N-acetylmuramoyl-L-alanyl-D-glutamyl-meso-2,6-diaminopimeloyl-D-alanyl-D-alanine synthetase	6.3.2.10	H16_A3277	murF	alaala + atp + ugmd -> adp + pi + ugmda	alaala + atp + ugmd -> adp + h + pi + ugmda	alaala + atp + ugmd -> adp + pi + ugmda
UACMAS	Peptidoglycan Biosynthesis	UDP-N-acetylmuramoyl-L-alanine synthetase	6.3.2.8	H16_A3167/H16_A3272	murC1/murC2	ala + atp + udpnam -> adp + pi + uama	ala + atp + udpnam -> adp + h + pi + uama	ala + atp + udpnam -> adp + h + pi + uama
UACMAGS	Peptidoglycan Biosynthesis	UDP-N-acetylmuramoyl-L-alanyl-D-glutamate synthetase	6.3.2.9	H16_A3275	murD	atp + dglu + uama -> adp + pi + uamag	atp + dglu + uama -> adp + h + pi + uamag	atp + dglu + uama -> adp + h + pi + uamag
UAGMPUT	Peptidoglycan Biosynthesis	UDP-N-acetylglucosamine-N-acetylmuramyl-(pentapeptide)pyrophosphoryl-undecaprenol N-acetylglucosamine transferase	2.4.1.227	H16_A3273	murG	udpnag + uagmda -> uaagmda + udp	udpnag + uagmda -> uaagmda + udp	udpnag + uagmda -> uaagmda + udp + h
GLNST2	Peptidoglycan Biosynthesis	glutamine synthetase	6.3.1.2	H16_A2335/H16_B0618/H16_B2191	glna/glnA2/glnA3	uaagmda + atp + nh4 -> uaagmmda + adp + pi	uaagmda + atp + nh4 -> uaagmmda + adp + pi + h	uaagmda + atp + nh4 -> uaagmmda + adp + pi + h
NACMAA	Peptidoglycan Biosynthesis	N-acetylmuramoyl-L-alanine amidase	3.5.1.28	H16_A0597/H16_A3236	amiC/unknown	acala + h2o -> acmur + ala	acala + h2o -> acmur + ala	acala + h2o -> acmur + ala
UNAMPLA	Peptidoglycan Biosynthesis	UDP-N-acetylmuramoylpentapeptide-lysine N6-alanyltransferase	2.3.2.10			5 gly + uaagmmda -> uaagmm5da + 5 h2o	5 gly + uaagmmda -> uaagmm5da + 5 h2o	5 gly + uaagmmda -> uaagmm5da + 5 h2o
PGPS	Peptidoglycan Biosynthesis	peptidoglycan precursor synthesis				uaagmm5da -> udcpdp + ppeptido	uaagmm5da -> udcpdp + ppeptido	uaagmm5da -> udcpdp + ppeptido
DALAT	Peptidoglycan Biosynthesis	D-alanine transaminase	2.6.1.21	H16_A2521	dat	ppeptido + dala -> PEPTIDO + dala_e	ppeptido + dala -> PEPTIDO + dala_e	ppeptido + dala -> PEPTIDO + dala_e
ALHD3	Phenylalanine metabolism	aldehyde dehydrogenase (phenylacetaldehyde, NAD)	1.2.1.39	H16_B1358/H16_B1939	paaK2/feaB	h2o + nad + pacald -> 2 h + nadh + pac	h2o + nad + pacald -> 2 h + nadh + pac	h2o + nad + pacald -> 2 h + nadh + pac
OXP4EH	Phenylalanine metabolism	2-oxopent-4-enoate hydratase	4.2.1.80	H16_A0143/H16_B0548/H16_B0597/H16_B0884	mhpD2/mhpD1/bphH/mhpD3	h2o + op4en -> hopt	h2o + op4en -> hopt	h2o + op4en -> hopt
PHEACL	Phenylalanine metabolism	phenylacetate-CoA ligase	6.2.1.30	H16_A0291/H16_A3313	unknown	atp + coa + h + pac -> amp + phaccoa + ppi	atp + coa + pac -> amp + phaccoa + ppi + h	atp + coa + pac -> amp + phaccoa + ppi + h
3HCINNMH	Phenylalanine metabolism	3-hydroxycinnamate hydroxylase	1.14.13.-	H16_B1546	unknown	3hcinnm + h + nadh + o2 -> dhcinnm + h2o + nad	3hcinnm + h + nadh + o2 -> dhcinnm + h2o + nad	3hcinnm + h + nadh + o2 -> dhcinnm + h2o + nad
3HPPPNH	Phenylalanine metabolism	3-(3-hydroxy-phenyl)propionate hydroxylase	1.14.13.-	H16_B1546	unknown	3hpppn + h + nadh + o2 -> dhpppn + h2o + nad	3hpppn + h + nadh + o2 -> dhpppn + h2o + nad	3hpppn + h + nadh + o2 -> dhpppn + h2o + nad
CINNMDO	Phenylalanine metabolism	Cinnamate dioxygenase	1.14.12.19	H16_A1632/H16_B0800	nagH/unknown	cinnm + h + nadh + o2 -> cenchddd + nad	cinnm + h + nadh + o2 -> cenchddd + nad	cinnm + h + nadh + o2 -> cenchddd + nad
4H2OPNTA	Phenylalanine metabolism	4-hydroxy-2-oxopentanoate aldolase	4.1.3.39	H16_A1807/H16_B0552/H16_B0595	mhpE2/mhpE1/bpHI	hopt -> acal + pyr	hopt -> acal + pyr	hopt -> acal + pyr
PPPNDO	Phenylalanine metabolism	Phenylpropanoate Dioxygenase	1.14.12.19	H16_A1632&H16_B0800	nagH&unknown	h + nadh + o2 + pppn -> cechddd + nad	h + nadh + o2 + pppn -> cechddd + nad	h + nadh + o2 + pppn -> cechddd + nad
HPPH	Phenylalanine metabolism	hippurate hydrolase	3.5.1.32	H16_A0073/H16_A3299/H16_B0605/H16_B1473	unknown/hipO/unknown/unknown	benzot + gly -> hppr + h2o	benzot + gly -> hppr + h2o	benzot + gly -> hppr + h2o
CNTOB	Phenylalanine metabolism	unclear reaction				cinnm + 2 h2o + nad -> benzot + ac + nadh + 2 h	cinnm + 2 h2o + nad -> benzot + ac + nadh + 2 h	cinnm + 2 h2o + nad -> benzot + ac + nadh + 2 h
AMDS1	Phenylalanine metabolism	amidase	3.5.1.4	H16_A1469/H16_B1874/H16_B2459	unknown/unknown/aimE	pheact + h2o -> pac + nh4	pheact + h2o -> pac + nh4	pheact + h2o -> pac + nh4
4HPHED2	Phenylalanine metabolism	4-hydroxyphenylpyruvate dioxygenase	1.13.11.27	H16_B1083	hpd	phpyr + o2 -> 2hpa + co2	phpyr + o2 -> 2hpa + co2	phpyr + o2 -> 2hpa + co2
MNAO9	Phenylalanine metabolism	monoamine oxidase	1.4.3.4	H16_A0831	maoB	peamn + o2 + h2o -> pacald + nh4 + h2o2	peamn + o2 + h2o -> pacald + nh4 + h2o2	peamn + o2 + h2o -> pacald + nh4 + h2o2
DATA3	Phenylalanine metabolism	D-alanine transaminase	2.6.1.21	H16_A2521	dat	phpyr + dglu <-> dphe + akg	phpyr + dglu <-> dphe + akg	phpyr + dglu <-> dphe + akg
DAAD2	Phenylalanine metabolism	D-Amino acid dehydrogenase	1.4.99.1	H16_A0770/H16_A0817/H16_A1505/H16_B0508/H16_B1893	dadA2/dadA1/dadA5/dadA6/dadA7	dphe + h2o + fad -> phpyr + nh4 + fadh2	dphe + h2o + fad + h -> phpyr + nh4 + fadh2	dphe + h2o + fad + h -> phpyr + nh4 + fadh2
LAAO5	Phenylalanine metabolism	L-amino-acid oxidase	1.4.3.2	H16_A0845/H16_A0856	lao1/lao2	phe + h2o + o2 -> phpyr + nh4 + h2o2	phe + h2o + o2 -> phpyr + nh4 + h2o2	phe + h2o + o2 -> phpyr + nh4 + h2o2
ASPAM6	Phenylalanine metabolism	aspartate aminotransferase	2.6.1.1	H16_A2857	unknown	phe + akg <-> phpyr + glu	phe + akg <-> phpyr + glu	phe + akg <-> phpyr + glu

ACTF3	Phenylalanine metabolism	acetyltransferase	2.3.1.-	H16_A0039/H16_A0240/H16_A0269/H16_A0699/H16_A1315/H16_A1564/H16_A1683/H16_A1802/H16_A2759/H16_A3071/H16_A3093/H16_A3221/H16_A3529/H16_A3586/H16_B0018/H16_B0021/H16_B0032/H16_B0219/H16_B1278/H16_B1292/H16_B1407/H16_B1663/H16_B1899/H16_B2397/	unknown/unknown/unknown/unknown/unknown/unknown/unknown/unknown/unknown/unknown/unknown/unknown/unknown/pat/unknown/unknown/wbpD/unknown/unknown/phnT/unknown/unknown/unknown/unknown/	phaccoa + gly -> pheacgly + coa + h	phaccoa + gly -> pheacgly + coa + h	phaccoa + gly -> pheacgly + coa + h
SHKDH	Phenylalanine, Tyrosine and Tryptophan biosynthesis	shikimate dehydrogenase	1.1.1.25	H16_A3161	aroE	dhsk + 2 h + nadph <-> nadp + sme	dhsk + h + nadph <-> nadp + sme	dhsk + h + nadph <-> nadp + sme
PPNDHG	Phenylalanine, Tyrosine and Tryptophan biosynthesis	prephenate dehydrogenase	1.3.1.12	H16_A0794	tyrA	nad + phen -> 4hpp + co2 + nadh	nad + phen -> 4hpp + co2 + nadh	nad + phen -> 4hpp + co2 + nadh
ANTPRT	Phenylalanine, Tyrosine and Tryptophan biosynthesis	anthranilate phosphoribosyltransferase	2.4.2.18	H16_A0356/H16_A3321	trpD2/trpD1	an + prpp -> ppi + npran	an + prpp -> ppi + npran + h	an + prpp -> ppi + npran + h
PSHKCVT	Phenylalanine, Tyrosine and Tryptophan biosynthesis	3-phosphoshikimate 1-carboxyvinyltransferase	2.5.1.19	H16_A0795	aroA	pep + skm5p <-> 3psme + pi	pep + skm5p <-> 3psme + h + pi	pep + skm5p <-> 3psme + h + pi
TYRTA1	Phenylalanine, Tyrosine and Tryptophan biosynthesis	tyrosine transaminase	2.6.1.9	H16_A0793/H16_A3415	hisC1/hisC2	akg + tyr <-> 4hpp + glu	akg + tyr <-> 4hpp + glu	akg + tyr <-> 4hpp + glu
TYRTA2	Phenylalanine, Tyrosine and Tryptophan biosynthesis	tyrosine transaminase	2.6.1.57	H16_A1151/H16_B1081	tyrB1/tyrB2	akg + tyr <-> 4hpp + glu	akg + tyr <-> 4hpp + glu	akg + tyr <-> 4hpp + glu
PHETA1	Phenylalanine, Tyrosine and Tryptophan biosynthesis	phenylalanine transaminase	2.6.1.58			akg + phe <-> glu + phpyr	akg + phe <-> glu + phpyr	akg + phe <-> glu + phpyr
PHETA2	Phenylalanine, Tyrosine and Tryptophan biosynthesis	phenylalanine transaminase	2.6.1.57	H16_A1151/H16_B1081	tyrB1/tyrB2	akg + phe <-> glu + phpyr	akg + phe <-> glu + phpyr	akg + phe <-> glu + phpyr
SHKK	Phenylalanine, Tyrosine and Tryptophan biosynthesis	shikimate kinase	2.7.1.71	H16_A3435	aroL	atp + sme -> adp + skm5p	atp + sme -> adp + skm5p + h	atp + sme -> adp + skm5p + h
IG3PS	Phenylalanine, Tyrosine and Tryptophan biosynthesis	indole-3-glycerol-phosphate synthase	4.1.1.48	H16_A3322	trpC	2cpr5p + h -> 3ig3p + co2 + h2o	2cpr5p + h -> 3ig3p + co2 + h2o	2cpr5p + h -> 3ig3p + co2 + h2o
DOXPHS	Phenylalanine, Tyrosine and Tryptophan biosynthesis	3-deoxy-7-phosphoheptulonate synthase	2.5.1.54	H16_A1122/H16_B1076	aroG1/aroG2	e4p + h2o + pep -> 3ddah7p + pi	e4p + h2o + pep -> 3ddah7p + h + pi	e4p + h2o + pep -> 3ddah7p + h + pi
ANTHS	Phenylalanine, Tyrosine and Tryptophan biosynthesis	anthranilate synthase	4.1.3.27	H16_A3319 & H16_A3320	trpE&trpG	chor + gln -> an + glu + h + pyr	chor + gln -> an + glu + h + pyr	chor + gln -> an + glu + h + pyr
DHQND	Phenylalanine, Tyrosine and Tryptophan biosynthesis	3-dehydroquinate dehydratase	4.2.1.10	H16_A3170/H16_B0465	aroQ1/aroQ2	dqt -> dhsk + h2o	dqt -> dhsk + h2o	dqt -> dhsk + h2o
TRPS1	Phenylalanine, Tyrosine and Tryptophan biosynthesis	tryptophan synthase	4.2.1.20	H16_A2612&H16_A2614	trpA&trpB	3ig3p + ser -> g3p + h2o + trp	3ig3p + ser -> g3p + h2o + trp	3ig3p + ser -> g3p + h2o + trp
TRPS2	Phenylalanine, Tyrosine and Tryptophan biosynthesis	tryptophan synthase	4.2.1.20	H16_A2612&H16_A2614	trpA&trpB	indole + ser -> h2o + trp	indole + ser -> h2o + trp	indole + ser -> h2o + trp
TRPS3	Phenylalanine, Tyrosine and Tryptophan biosynthesis	tryptophan synthase	4.2.1.20	H16_A2612&H16_A2614	trpA&trpB	3ig3p -> g3p + indole	3ig3p -> g3p + indole	3ig3p -> g3p + indole
PPNDHT	Phenylalanine, Tyrosine and Tryptophan biosynthesis	prephenate dehydratase	4.2.1.51	H16_A0792	pheA	h + phen -> co2 + h2o + phpyr	h + phen -> co2 + h2o + phpyr	h + phen -> co2 + h2o + phpyr
CHORS	Phenylalanine, Tyrosine and Tryptophan biosynthesis	chorismate synthase	4.2.3.5	H16_A1317	aroC	3psme -> chor + pi	3psme -> chor + pi	3psme -> chor + pi
PRANTI	Phenylalanine, Tyrosine and Tryptophan biosynthesis	phosphoribosylanthranilate isomerase	5.3.1.24	H16_A2615	trpF	npran -> 2cpr5p	npran -> 2cpr5p	npran -> 2cpr5p
CHORM	Phenylalanine, Tyrosine and Tryptophan biosynthesis	chorismate mutase	5.4.99.5	H16_A0792	pheA	chor -> phen	chor -> phen	chor -> phen
DHQTS	Phenylalanine, Tyrosine and Tryptophan biosynthesis	3-dehydroquinate synthase	4.2.3.4	H16_A3434	aroB	3ddah7p -> dqt + pi	3ddah7p -> dqt + pi	3ddah7p -> dqt + pi
QDPQ1	Phenylalanine, Tyrosine and Tryptophan biosynthesis	quinate dehydrogenase (pyrroloquinoline-quinone)	1.1.99.25	H16_B1047	quiA	sme + pqq <-> dhsk + pqqh2	sme + pqq <-> dhsk + pqqh2	sme + pqq <-> dhsk + pqqh2
QDPQ2	Phenylalanine, Tyrosine and Tryptophan biosynthesis	quinate dehydrogenase (pyrroloquinoline-quinone)	1.1.99.25	H16_B1047	quiA	dhsk + pqqh2 + h2o <-> qt + pqq	dhsk + pqqh2 + h2o <-> qt + pqq	dhsk + pqqh2 + h2o <-> qt + pqq
PHEA4H	Phenylalanine, Tyrosine and Tryptophan biosynthesis	phenylalanine-4-hydroxylase	1.14.16.1	H16_A3678	phhA	tethbp + phe + o2 -> dhbpt + tyr + h2o	tethbp + phe + o2 -> dhbpt + tyr + h2o	tethbp + phe + o2 -> dhbpt + tyr + h2o
TYRTM	Phenylalanine, Tyrosine and Tryptophan biosynthesis	aromatic-amino-acid transaminase	2.6.1.57	H16_A1151/H16_B1081	tyrB1/tyrB2	phen + asp -> oaa + ag	phen + asp -> oaa + ag	phen + asp -> oaa + ag
CPPPGO	Porphyrin and Chlorophyll metabolism	coproporphyrinogen oxidase (O2 required)	1.3.3.3	H16_A0914	hemF	cpp + 2 h + o2 -> 2 co2 + 2 h2o + pphg	cpp + 2 h + o2 -> 2 co2 + 2 h2o + pphg	cpp + 2 h + o2 -> 2 co2 + 2 h2o + pphg
PPHGO	Porphyrin and Chlorophyll metabolism	protoporphyrinogen oxidase (aerobic)	1.3.3.4	H16_A2891/H16_B2453	unknown/unknown	3 o2 + 2 pphg -> 6 h2o + 2 ppix	3 o2 + 2 pphg -> 6 h2o + 2 ppix	3 o2 + 2 pphg -> 6 h2o + 2 ppix
PPHGOx	Porphyrin and Chlorophyll metabolism	protoporphyrinogen oxidase (anaerobic)	1.3.3.4	H16_A2891/H16_B2453	unknown/unknown	3 fum + pphg -> ppix + 3 succ	3 fum + pphg -> ppix + 3 succ	3 fum + pphg -> ppix + 3 succ
UPPMT	Porphyrin and Chlorophyll metabolism	uroporphyrinogen methyltransferase	2.1.1.107	H16_A2919/H16_A2994/H16_B2285	hemXD/cysG/nirE	2 sam + uppg3 -> 2 sah + dscl + h	2 sam + uppg3 -> 2 sah + dscl + h	2 sam + uppg3 -> 2 sah + dscl + h

CBIAT1	Porphyrin and Chlorophyll metabolism	Cobinamide adenyltransferase	2.5.1.17	H16_A2969/H16_A3090	btuR/unknown	atp + cbi <-> adcba + pppi	atp + cbi <-> adcba + pppi	atp + cbi <-> adcba + pppi + h
UPPDC1	Porphyrin and Chlorophyll metabolism	uroporphyrinogen decarboxylase (uroporphyrinogen III)	4.1.1.37	H16_A3633	hemE	4 h + uppg3 -> 4 co2 + cpp	4 h + uppg3 -> 4 co2 + cpp	4 h + uppg3 -> 4 co2 + cpp
PPBINGS	Porphyrin and Chlorophyll metabolism	porphobilinogen synthase	4.2.1.24	H16_A3453	hemB	2 5aop -> h + 2 h2o + pbg	2 5aop -> h + 2 h2o + pbg	2 5aop + h -> 2 h2o + pbg
HMBS	Porphyrin and Chlorophyll metabolism	hydroxymethylbilane synthase	2.5.1.16	H16_A2920	hemC	h2o + 4 pbg -> hmb + 4 nh4	h2o + 4 pbg -> hmb + 4 nh4	h2o + 4 pbg -> hmb + 4 nh4
FERCLT	Porphyrin and Chlorophyll metabolism	ferrochelatase	4.99.1.1	H16_A1134	hemH	fe2 + ppix -> pth + 2 h	fe2 + ppix -> pth + 2 h	fe2 + ppix -> pth + 2 h
GLU1SAT	Porphyrin and Chlorophyll metabolism	glutamate-1-semialdehyde aminotransferase	5.4.3.8	H16_A0734	hemL	glu1sa <-> 5aop	glu1sa <-> 5aop	glu1sa <-> 5aop + h
GLUTRS	Porphyrin and Chlorophyll metabolism	Glutamyl-tRNA synthetase	6.1.1.17	H16_A2403/H16_A2716	gltX1/gltX2	atp + glu + h + trnaglu -> amp + glutrna + ppi	atp + glu + trnaglu -> amp + glutrna + ppi + h	atp + glu + trnaglu -> amp + glutrna + ppi + h
ACOBPGT	Porphyrin and Chlorophyll metabolism	Adenosyl cobinamide phosphate guanyltransferase	2.7.7.62	H16_A2962	cobU	adcbap + gtp -> agdpcba + ppi	adcbap + gtp -> agdpcba + ppi	adcbap + gtp -> agdpcba + ppi
ADCOBK	Porphyrin and Chlorophyll metabolism	Adenosyl cobinamide kinase	2.7.1.156	H16_A2962	cobU	adcba + atp -> adcbap + adp	adcba + atp -> adcbap + adp + h	adcba + atp -> adcbap + adp + h
ADCOBPS	Porphyrin and Chlorophyll metabolism	Adenosylcobalamin 5'-phosphate synthase	2.7.8.26	H16_A2967	cobS1	agdpcba + rdmbzi + h -> adocbl + gmp	agdpcba + rdmbzi -> adocbl + gmp + h	agdpcba + rdmbzi -> adocbl + gmp + h
GLUTRR	Porphyrin and Chlorophyll metabolism	glutamyl-tRNA reductase	1.2.1.70	H16_A3339	hemA	glutrna + 2 h + nadph -> glu1sa + nadp + trnaglu	glutrna + h + nadph -> glu1sa + nadp + trnaglu	glutrna + h + nadph -> glu1sa + nadp + trnaglu
HEMEOS	Porphyrin and Chlorophyll metabolism	Heme O synthase	2.5.1.-	H16_A0352	ctaB	frdp + h2o + pth + 2 h -> hemeO + ppi	frdp + h2o + pth + h -> hemeO + ppi	frdp + h2o + pth + h -> hemeO + ppi
UPPDC2	Porphyrin and Chlorophyll metabolism	uroporphyrinogen decarboxylase	4.1.1.37	H16_A3633	hemE	uppg1 + 4 h -> cppi + 4 co2	uppg1 + 4 h -> cppi + 4 co2	uppg1 + 4 h -> cppi + 4 co2
SRHCC	Porphyrin and Chlorophyll metabolism	sirohydrochlorin cobaltochelatase	4.99.1.3	H16_A2993	cbiX	shcl + cobalt2 -> cobtpc + 2 h	shcl + cobalt2 -> cobtpc + 2 h	shcl + cobalt2 -> cobtpc + 2 h
PHEMEAS	Porphyrin and Chlorophyll metabolism	putative hemeA synthase		H16_A0351	ctaA	hemeO + h2o -> hemeA + 2 h2	hemeO + h2o -> hemeA + 2 h2	hemeO + h2o -> hemeA + 2 h2
CBIAT2	Porphyrin and Chlorophyll metabolism	Cobinamide adenyltransferase	2.5.1.17	H16_A2969/H16_A3090	btuR/unknown	cobacd + atp -> acda + pppi	cobacd + atp -> acda + pppi	cobacd + atp -> acda + pppi + h
ADNCOS	Porphyrin and Chlorophyll metabolism	adenosylcobyric acid synthase	6.3.5.10	H16_A2961	cbiP	acda + 4 gln + 4 atp + 4 h2o + h -> acha + 4 glu + 4 pi + 4 adp	acda + 4 gln + 4 atp + 4 h2o -> acha + 4 glu + 4 pi + 4 adp + 3 h	acda + 4 gln + 4 atp + 4 h2o -> acha + 4 glu + 4 pi + 4 adp + 3 h
COBBP1	Porphyrin and Chlorophyll metabolism	cobalamin biosynthetic protein CobC	6.3.1.10	H16_A2963/H16_A2964	cbiB/cobD	atp + acha + amppo -> adp + pi + adcba + h	atp + acha + amppo -> adp + pi + adcba + 2 h	atp + acha + amppo -> adp + pi + adcba + 2 h
COBBP2	Porphyrin and Chlorophyll metabolism	cobalamin biosynthetic protein CobC	6.3.1.10	H16_A2963/H16_A2964	cbiB/cobD	acha + d1ap2oop + atp -> adcbap + adp + pi + h	acha + d1ap2oop + atp -> adcbap + adp + pi + 2 h	acha + d1ap2oop + atp -> adcbap + adp + pi + 2 h
PPNAK	Propanoate metabolism	Propionate kinase	2.7.2.1	H16_A0670/H16_B1630	ackA2/ackA	adp + ppap <-> atp + ppa	adp + ppap <-> atp + ppa	adp + ppap <-> atp + ppa
MICITL	Propanoate metabolism	methylisocitrate lyase	4.1.3.30	H16_A1905	prpB	micit <-> pyr + succ	micit <-> pyr + succ	micit <-> pyr + succ
MCITDH	Propanoate metabolism	2-methylcitrate dehydratase	4.2.1.79	H16_A1909/H16_B0681/H16_B1436/H16_B1444	prpD1/prpD2/prpD3/prpD4	2mcit -> maco + h2o	2mcit -> maco + h2o	2mcit -> maco + h2o
OBUTFL	Propanoate metabolism	2-Oxobutanoate formate lyase	2.3.1.54			obut + coa -> formate + ppcoa	obut + coa -> formate + ppcoa	obut + coa -> formate + ppcoa
PACTF	Propanoate metabolism	Phosphate acetyltransferase	2.3.1.8	H16_B1631/H16_B1871	pta1/pta2	pi + ppcoa -> coa + ppap + h	pi + ppcoa -> coa + ppap	pi + ppcoa -> coa + ppap
MCDCX	Propanoate metabolism	malonyl-CoA decarboxylase	4.1.1.9	H16_A2981	mcd	malcoa + h -> accoa + co2	malcoa + h -> accoa + co2	malcoa + h -> accoa + co2
HIBCH	Propanoate metabolism	3-hydroxyisobutyryl-CoA hydrolase	6.2.1.-	H16_A0866/H16_A0871/H16_A1230/H16_A1519/H16_A1700/H16_A1718/H16_A2252/H16_A2794/H16_A2807/H16_A2978/H16_B0174/H16_B0677/H16_B0910/H16_B1148/H16_B1264/H16_B1335/H16_B1662/H16_B1709/H16_B2522	unknown/unknown/unknown/unknown/unknown/unknown/unknown/unknown/unknown/unknown/unknown/unknown/unknown/unknown/unknown/unknown/unknown/unknown/unknown/	3hpcoa + pi + adp <-> 3hpp + coa + atp + h	3hpcoa + pi + adp <-> 3hpp + coa + atp	3hpcoa + pi + adp <-> 3hpp + coa + atp
ENCOAH2	Propanoate metabolism	enoyl-CoA hydratase	4.2.1.17	H16_A0100/H16_A0142/H16_A0179/H16_A0461/H16_A0464/H16_A0810/H16_A0865/H16_A0873/H16_A1101/H16_A1410/H16_A1699/H16_A1716/H16_A1719/H16_A1832/H16_A1885/H16_A1889/H16_A2138/H16_A2258/H16_A2979/H16_A3201/H16_A3311/H16_A3593/H16_A3594/H16_B0365/H16_B0382/H16_B0389/H16_B0402/H16_B0419/H16_B0420/H16_B0657/H16_B0659/H16_B0698/H16_B0724/H16_B0756/H16_B0848/H16_B0915/H16_B1188/H16_B1346/H16_B1439/H16_B1738/H16_B1741/H16_B1742/H16_B1773/H16_B1905/H16_B1914/H16_B2156/H16_B2478/	unknown/	3hpcoa <-> ppecoa + h2o	3hpcoa <-> ppecoa + h2o	3hpcoa <-> ppecoa + h2o

ACOADH2	Propanoate metabolism	acyl-CoA dehydrogenase	1.3.99.3	H16_A0101/H16_A0460/H16_A0816/H16_A0843/H16_A0863/H16_A1067/H16_A1068/H16_A1530/H16_A2458/H16_A2596/H16_B0014/H16_B0087/H16_B0356/H16_B0360/H16_B0379/H16_B0383/H16_B0384/H16_B0395/H16_B0396/H16_B0400/H16_B0580/H16_B0660/H16_B0661/H16_B0664/H16_B0665/H16_B0676/H16_B0683/H16_B0703/H16_B0704/H16_B0721/H16_B0722/H16_B0751/H16_B0849/H16_B0909/H16_B0913/H16_B0938/H16_B0975/H16_B1192/H16_B1332/H16_B1367/H16_B1481/H16_B1694/H16_B1826/H16_B2157/H16_B2158/	unknown/acaD/unknown/unknown/unknown/unknown/unknown/abmD/unknown/	fad + ppcoa <-> fadh2 + ppecoa	fad + ppcoa + h <-> fadh2 + ppecoa	fad + ppcoa + h <-> fadh2 + ppecoa
BKAR1	Propanoate metabolism	beta-ketoacyl-ACP reductase	1.3.1.-	H16_B0731/H16_B0734	unknown/unknown	ppcoa + nadp <-> ppecoa + nadph + 2 h	ppcoa + nadp <-> ppecoa + nadph + h	ppcoa + nadp <-> ppecoa + nadph + h
ACCSYN1	Propanoate metabolism	acetyl-CoA synthetase	6.2.1.1	H16_A1197/H16_A1616/H16_A2525/H16_B0386/H16_B0696/H16_B0834/H16_B1102/	unknown/unknown/acoE/unknown/unknown/unknown/unknown/	ppcoa + amp <-> ppald + coa	ppcoa + amp + h <-> ppald + coa	ppcoa + amp + h <-> ppald + coa
ACCSYN2	Propanoate metabolism	acetyl-CoA synthetase	6.2.1.1	H16_A1197/H16_A1616/H16_A2525/H16_B0386/H16_B0696/H16_B0834/H16_B1102/	unknown/unknown/acoE/unknown/unknown/unknown/unknown/	ppald + ppi <-> ppa + atp + h	ppald + ppi <-> ppa + atp	ppald + ppi <-> ppa + atp
PPCSYN1	Propanoate metabolism	propionyl-CoA synthetase	6.2.1.17	H16_A2462	prpE	ppcoa + amp <-> ppald + coa	ppcoa + amp + h <-> ppald + coa	ppcoa + amp + h <-> ppald + coa
PPCSYN2	Propanoate metabolism	propionyl-CoA synthetase	6.2.1.17	H16_A2462	prpE	ppald + ppi <-> ppa + atp + h	ppald + ppi <-> ppa + atp	ppald + ppi <-> ppa + atp
PPCCX	Propanoate metabolism	propionyl-CoA carboxylase	6.4.1.3	H16_A0177/H16_A1973/H16_A2144	unknown/unknown/pccB	atp + ppcoa + hco3 -> adp + pi + mmcoa-S + h	atp + ppcoa + hco3 -> adp + pi + mmcoa-S + h	atp + ppcoa + hco3 -> adp + pi + mmcoa-S + h
2MCITS	Propanoate metabolism	2-methylcitrate synthase	2.3.3.5	H16_A1906/H16_A2636	prpC1/prpC2	ppcoa + oaa + h2o <-> 2mcit + coa + h	ppcoa + oaa + h2o <-> 2mcit + coa + h	ppcoa + oaa + h2o <-> 2mcit + coa + h
MMSDH	Propanoate metabolism	methylmalonate-semialdehyde dehydrogenase	1.2.1.27	H16_A0273/H16_A3664/H16_B1191	mmsA1/mmsA2/mmsA3	mmsa + coa + nad -> ppcoa + co2 + nadh	mmsa + coa + nad -> ppcoa + co2 + nadh	mmsa + coa + nad -> ppcoa + co2 + nadh
L-LACD4	Propanoate metabolism	L-lactate dehydrogenase	1.1.1.27	H16_A0666	ldh	2hba + nad -> obut + nadh + h	2hba + nad -> obut + nadh + h	2hba + nad -> obut + nadh + h
ACPCD	Propanoate metabolism	1-aminocyclopropane-1-carboxylate deaminase	3.5.99.7	H16_B1365	acd	acpc + h2o <-> obut + nh4	acpc + h2o <-> obut + nh4	acpc + h2o <-> obut + nh4
PCT2	Propanoate metabolism	propionate CoA-transferase	2.8.3.1	H16_A2718	pct	lactcoa + ppa <-> llac + ppcoa	lactcoa + ppa <-> llac + ppcoa	lactcoa + ppa <-> llac + ppcoa
MCTOP	Propanoate metabolism	unclear reaction				malcoa + h2o + nadp <-> 3oppcoa + o2 + nadph + h	malcoa + h2o + nadp <-> 3oppcoa + o2 + nadph	malcoa + h2o + nadp <-> 3oppcoa + o2 + nadph
OPTHP	Propanoate metabolism	unclear reaction				3oppcoa + nadph + 2 h <-> 3hpcoa + nadp	3oppcoa + nadph + h <-> 3hpcoa + nadp	3oppcoa + nadph + h <-> 3hpcoa + nadp
ALHD16	Propanoate metabolism	aldehyde dehydrogenase (NAD+)	1.2.1.3	H16_A0232/H16_A0745/H16_A1114/H16_A1495/H16_B0212/H16_B0421/H16_B0737/H16_B0833/H16_B1534/H16_B1735/H16_B1751/H16_B1835/H16_B1960/H16_B2444	unknown/unknown/unknown/unknown/unknown/unknown/unknown/unknown/unknown/unknown/unknown/unknown/exaC/unknown/	2p1a + nad + h2o -> ppn + nadh + 2 h	2p1a + nad + h2o -> ppn + nadh + 2 h	2p1a + nad + h2o -> ppn + nadh + 2 h
2MCITDT	Propanoate metabolism	2-methylisocitrate dehydratase	4.2.1.99	H16_A1907	acnM	maco + h2o <-> micit	maco + h2o <-> micit	maco + h2o <-> micit
IMPCH	Puine metabolism	IMP cyclohydrolase	3.5.4.10	H16_A0501	purH	h2o + imp <-> fprica	h2o + imp <-> fprica	h2o + imp <-> fprica
PRAIZC2	Purine and Pyrimidine Biosynthesis	phosphoribosylamino imidazole carboxylase				cair <-> caiz	cair <-> caiz	cair <-> caiz
GARTFT	Purine and Pyrimidine Biosynthesis	GAR transformylase-T	2.1.2.-			atp + formate + gar -> adp + fgam + pi	atp + formate + gar -> adp + fgam + h + pi	atp + formate + gar -> adp + fgam + pi
GTPDPK	Purine metabolism	GTP diphosphokinase	2.7.6.5	H16_A0955/H16_A1337	spoT1/spoT2	atp + gtp -> amp + pppgpp	atp + gtp -> amp + pppgpp + 2 h	atp + gtp -> amp + pppgpp + h
G5TDPDP	Purine metabolism	guanosine-5'-triphosphate,3'-diphosphate diphosphatase	3.6.1.11	H16_A2436	ppx	pppgpp + h2o -> pi + ppgpp	pppgpp + h2o -> h + pi + ppgpp	pppgpp + h2o -> h + pi + ppgpp
URGLYCH	Purine metabolism	Ureidoglycolate hydrolase	3.5.3.19	H16_B2455	unknown	2 h + h2o + urdglyc -> co2 + glx + 2 nh4	2 h + h2o + urdglyc -> co2 + glx + 2 nh4	2 h + h2o + urdglyc -> co2 + glx + 2 nh4
XANDH	Purine metabolism	xanthine dehydrogenase	1.17.1.4	H16_A1016/H16_A1017/H16_A3371/H16_B1897/H16_B1898	xdhB1/xdhA1/coxL5/xdhA2/xdhB2	h2o + nad + xan -> h + nadh + urate	h2o + nad + xan -> h + nadh + urate	h2o + nad + xan -> 2 h + nadh + urate
RNDPR1	Purine metabolism	ribonucleoside-diphosphate reductase (ADP)	1.17.4.1	H16_A2390/(H16_A3234&H16_A3235)	nrdJ/(nrdB&nrdA)	adp + rthio -> dadp + h2o + othio	adp + rthio -> dadp + h2o + othio	adp + rthio -> dadp + h2o + othio
RNDPR2	Purine metabolism	ribonucleoside-diphosphate reductase (GDP)	1.17.4.1	H16_A2390/(H16_A3234&H16_A3235)	nrdJ/(nrdB&nrdA)	gdp + rthio -> dgdp + h2o + othio	gdp + rthio -> dgdp + h2o + othio	gdp + rthio -> dgdp + h2o + othio
RNDPR3	Purine metabolism	ribonucleoside-diphosphate reductase (CDP)	1.17.4.1	H16_A2390/(H16_A3234&H16_A3235)	nrdJ/(nrdB&nrdA)	cdp + rthio -> dcdp + h2o + othio	cdp + rthio -> dcdp + h2o + othio	cdp + rthio -> dcdp + h2o + othio
RNDPR4	Purine metabolism	ribonucleoside-diphosphate reductase (UDP)	1.17.4.1	H16_A2390/(H16_A3234&H16_A3235)	nrdJ/(nrdB&nrdA)	rthio + udp -> dudp + h2o + othio	rthio + udp -> dudp + h2o + othio	rthio + udp -> dudp + h2o + othio
PUNPP1	Purine metabolism	purine-nucleoside phosphorylase (Adenosine)	2.4.2.4	H16_A2012	deoA	adn + pi <-> ad + r1p	adn + pi <-> ad + r1p	adn + pi <-> ad + r1p
PUNPP2	Purine metabolism	purine-nucleoside phosphorylase (Deoxyadenosine)	2.4.2.4	H16_A2012	deoA	da + pi <-> dr1p + ad	da + pi <-> dr1p + ad	da + pi <-> dr1p + ad
PUNPP3	Purine metabolism	purine-nucleoside phosphorylase (Guanosine)	2.4.2.4	H16_A2012	deoA	gsn + pi <-> gn + r1p	gsn + pi <-> gn + r1p	gsn + pi <-> gn + r1p
PUNPP4	Purine metabolism	purine-nucleoside phosphorylase (Deoxyguanosine)	2.4.2.4	H16_A2012	deoA	dg + pi <-> dr1p + gn	dg + pi <-> dr1p + gn	dg + pi <-> dr1p + gn
PUNPP5	Purine metabolism	purine-nucleoside phosphorylase (Inosine)	2.4.2.4	H16_A2012	deoA	ins + pi <-> hyxn + r1p	ins + pi <-> hyxn + r1p	ins + pi <-> hyxn + r1p
PUNPP6	Purine metabolism	purine-nucleoside phosphorylase (Deoxyinosine)	2.4.2.4	H16_A2012	deoA	din + pi <-> dr1p + hyxn	din + pi <-> dr1p + hyxn	din + pi <-> dr1p + hyxn
PUNPP7	Purine metabolism	purine-nucleoside phosphorylase (Xanthosine)	2.4.2.4	H16_A2012	deoA	pi + xtsine <-> r1p + xan	pi + xtsine <-> r1p + xan	pi + xtsine <-> r1p + xan
XANPRT	Purine metabolism	xanthine phosphoribosyltransferase	2.4.2.8	H16_A3242	hprT	prpp + xan -> ppi + xmp	prpp + xan -> ppi + xmp + h	prpp + xan -> ppi + xmp + h
ADPRT1	Purine metabolism	adenine phosphoribosyltransferase	2.4.2.7	H16_A0395	apt	ad + prpp -> amp + ppi	ad + prpp -> amp + ppi + h	ad + prpp -> amp + ppi + h
GNPRT	Purine metabolism	guanine phosphoribosyltransferase	2.4.2.8	H16_A3242	hprT	gn + prpp -> gmp + ppi	gn + prpp -> gmp + ppi + h	gn + prpp -> gmp + ppi + h

HYXNPRT	Purine metabolism	hypoxanthine phosphoribosyltransferase (Hypoxanthine)	2.4.2.8	H16_A3242	hprT	hyxn + prpp -> imp + ppi	hyxn + prpp -> imp + ppi + h	hyxn + prpp -> imp + ppi + h
ADNK	Purine metabolism	adenosine kinase	2.7.1.20			adn + atp -> adp + amp	adn + atp -> adp + amp + h	adn + atp -> adp + amp + h
DADNK	Purine metabolism	deoxyadenylate kinase	2.7.4.3	H16_A0603	adk	atp + damp <-> adp + dadp	atp + damp + h <-> adp + dadp	atp + damp <-> adp + dadp
ADNK1	Purine metabolism	adenylate kinase	2.7.4.3	H16_A0603	adk	amp + atp <-> 2 adp	amp + atp + h <-> 2 adp	amp + atp <-> 2 adp
NUDPK1	Purine metabolism	nucleoside-diphosphate kinase (ATP:GDP)	2.7.4.6	H16_A2368	ndk	atp + gdp <-> adp + gtp	atp + gdp <-> adp + gtp	atp + gdp <-> adp + gtp
NUDPK5	Purine metabolism	nucleoside-diphosphate kinase (ATP:dGDP)	2.7.4.6	H16_A2368	ndk	atp + dgdp <-> adp + dgtp	atp + dgdp <-> adp + dgtp	atp + dgdp <-> adp + dgtp
NUDPK8	Purine metabolism	nucleoside-diphosphate kinase (ATP:dADP)	2.7.4.6	H16_A2368	ndk	atp + dadp <-> adp + datp	atp + dadp <-> adp + datp	atp + dadp <-> adp + datp
DGNK	Purine metabolism	deoxyguanylate kinase (dGMP:ATP)	2.7.4.8	H16_A0953	gmk	atp + dgmp <-> adp + dgdp	atp + dgmp + h <-> adp + dgdp	atp + dgmp <-> adp + dgdp
GKN	Purine metabolism	guanylate kinase (GMP:ATP)	2.7.4.8	H16_A0953	gmk	atp + gmp <-> adp + gdp	atp + gmp + h <-> adp + gdp	atp + gmp <-> adp + gdp
NUTD10	Purine metabolism	5'-nucleotidase (XMP)	3.1.3.5	H16_A2376	surE	h2o + xmp -> pi + xtsine	h2o + xmp -> pi + xtsine	h2o + xmp -> pi + xtsine
NUTD11	Purine metabolism	5'-nucleotidase (IMP)	3.1.3.5	H16_A2376	surE	h2o + imp -> ins + pi	h2o + imp -> ins + pi	h2o + imp -> ins + pi
NUTD6	Purine metabolism	5'-nucleotidase (dAMP)	3.1.3.5	H16_A2376	surE	damp + h2o -> da + pi	damp + h2o -> da + pi	damp + h2o -> da + pi
NUTD7	Purine metabolism	5'-nucleotidase (AMP)	3.1.3.5	H16_A2376	surE	amp + h2o -> adn + pi	amp + h2o -> adn + pi	amp + h2o -> adn + pi
NUTD8	Purine metabolism	5'-nucleotidase (dGMP)	3.1.3.5	H16_A2376	surE	dgmp + h2o -> dg + pi	dgmp + h2o -> dg + pi	dgmp + h2o -> dg + pi
NUTD9	Purine metabolism	5'-nucleotidase (GMP)	3.1.3.5	H16_A2376	surE	gmp + h2o -> gsn + pi	gmp + h2o -> gsn + pi	gmp + h2o -> gsn + pi
NUTPTP1	Purine metabolism	Nucleoside triphosphate tripolyhydrolase	3.1.5.1	H16_A3433	dgt	dgtp + h2o -> dg + pppi	dgtp + h2o -> dg + pppi	dgtp + h2o -> dg + pppi + h
NUTPTP2	Purine metabolism	Nucleoside triphosphate tripolyhydrolase	3.1.5.1	H16_A3433	dgt	gtp + h2o -> gsn + pppi	gtp + h2o -> gsn + pppi	gtp + h2o -> gsn + pppi + h
AMPNS	Purine metabolism	AMP nucleosidase	3.2.2.4	H16_B0070	amn	amp + h2o -> ad + r5p	amp + h2o -> ad + r5p	amp + h2o -> ad + r5p
GNDA	Purine metabolism	guanine deaminase	3.5.4.3	H16_A1013	guaD	gn + h + h2o -> nh4 + xan	gn + h + h2o -> nh4 + xan	gn + h + h2o -> nh4 + xan
ADNA	Purine metabolism	Adenosine deaminase	3.5.4.4	H16_A1014/H16_B2033	add/unknown	adn + h + h2o -> ins + nh4	adn + h + h2o -> ins + nh4	adn + h + h2o -> ins + nh4
ADPRDP	Purine metabolism	ADPribose diphosphatase	3.6.1.13	H16_A1404	unknown	adprib + h2o -> amp + r5p	adprib + h2o -> amp + r5p + 2 h	adprib + h2o -> amp + r5p + 2 h
NUTP1	Purine metabolism	nucleoside-triphosphatase (ATP)	3.6.1.15	H16_A0948	unknown	atp + h2o -> adp + pi	atp + h2o -> adp + h + pi	atp + h2o -> adp + h + pi
NUTP2	Purine metabolism	nucleoside-triphosphatase (GTP)	3.6.1.15	H16_A0948	unknown	gtp + h2o -> gdp + pi	gtp + h2o -> gdp + pi + h	gtp + h2o -> gdp + pi + h
ADNCYC	Purine metabolism	adenylate cyclase	4.6.1.1	H16_A0674/H16_A0827/H16_A1791/H16_A1809/H16_B0376	unknown/cycR1/unknown/unknown/cycR2	atp -> camp + ppi	atp -> camp + ppi + h	atp -> camp + ppi + h
DANDA	Purine metabolism	Deoxyadenosine deaminase	3.5.4.4	H16_A1014/H16_B2033	add/unknown	da + h + h2o -> din + nh4	da + h + h2o -> din + nh4	da + h + h2o -> din + nh4
BNTP	Purine metabolism	bis(5'-nucleosidyl)-tetraphosphatase	3.6.1.17	H16_A3406	unknown	gp4g + h2o -> gtp + gmp	gp4g + h2o -> gtp + gmp + h	gp4g + h2o -> gtp + gmp + 2 h
HYXND	Purine metabolism	hypoxanthine dehydrogenase	1.17.1.4	H16_A1016/H16_A1017/H16_A3371/H16_B1897/H16_B1898	xdhB1/xdhA1/coxL5/xdhA2/xdhB2	h2o + hyxn + nad -> h + nadh + xan	h2o + hyxn + nad -> h + nadh + xan	h2o + hyxn + nad -> h + nadh + xan
IMPDH	Purine metabolism	IMP dehydrogenase	1.1.1.205	H16_A2030	guaB	h2o + imp + nad -> h + nadh + xmp	h2o + imp + nad -> h + nadh + xmp	h2o + imp + nad -> h + nadh + xmp
PRGNFT	Purine metabolism	phosphoribosylglycinamide formyltransferase	2.1.2.2	H16_A3042	purN	fthf + gar <-> fgam + h + thf	fthf + gar <-> fgam + h + thf	fthf + gar <-> fgam + thf
PRAZCFT	Purine metabolism	phosphoribosylaminoimidazolecarboxamide formyltransferase	2.1.2.3	H16_A0501	purH	fthf + aicar <-> fprica + thf	fthf + aicar <-> fprica + thf	fthf + aicar <-> fprica + thf
GLUPRPAT	Purine metabolism	glutamine phosphoribosyldiphosphate amidotransferase	2.4.2.14	H16_A2607	purF	gln + h2o + prpp -> glu + ppi + pram	gln + h2o + prpp -> glu + ppi + pram + h	gln + h2o + prpp -> glu + ppi + pram + 2 h
ADSUCL1	Purine metabolism	adenylsuccinate lyase	4.3.2.2	H16_A3124	purB	asuc <-> amp + fum	asuc <-> amp + fum	asuc <-> amp + fum
ADSUCL2	Purine metabolism	adenylsuccinate lyase	4.3.2.2	H16_A3124	purB	saicar <-> aicar + fum	saicar <-> aicar + fum + h	saicar <-> aicar + fum + h
PRASUCS	Purine metabolism	phosphoribosylaminoimidazolesuccinocarboxamide synthase	6.3.2.6	H16_A0569	purC	cair + asp + atp -> saicar + adp + h + pi	cair + asp + atp -> saicar + adp + h + pi	cair + asp + atp -> saicar + adp + pi
PRAIZS	Purine metabolism	phosphoribosylaminoimidazole synthase	6.3.3.1	H16_A3077	purM	atp + fpram -> adp + air + pi	atp + fpram -> adp + air + h + pi	atp + fpram -> adp + air + h + pi
PRGCS	Purine metabolism	phosphoribosylglycinamide synthase	6.3.4.13	H16_A0915	purD	atp + gly + pram <-> adp + gar + pi	atp + gly + pram <-> adp + gar + h + pi	atp + gly + pram <-> adp + gar + h + pi
ADSUCS	Purine metabolism	adenylosuccinate synthase	6.3.4.4	H16_A2354/H16_B1994	purA1/purA2	asp + gtp + imp -> asuc + gdp + h + pi	asp + gtp + imp -> asuc + gdp + 2 h + pi	asp + gtp + imp -> asuc + gdp + 2 h + pi
GMPS	Purine metabolism	GMP synthase	6.3.5.2	H16_A2028	guaA	atp + gln + h2o + xmp -> amp + glu + gmp + h + ppi	atp + gln + h2o + xmp -> amp + glu + gmp + 3 h + ppi	atp + gln + h2o + xmp -> amp + glu + gmp + 3 h + ppi
PRFGAS	Purine metabolism	phosphoribosylformylglycinamidine synthase	6.3.5.3	H16_A1511	purL	atp + fgam + gln + h2o -> adp + fpram + glu + pi	atp + fgam + gln + h2o -> adp + fpram + glu + h + pi	atp + fgam + gln + h2o -> adp + fpram + glu + 2 h + pi
PRAIZC1	Purine metabolism	phosphoribosylaminoimidazole carboxylase	4.1.1.21	H16_A0570/H16_A0571	purE/purK	air + co2 <-> caiz + h	air + co2 <-> caiz + h	air + co2 <-> caiz + h
ADPRT2	Purine metabolism	adenine phosphoribosyltransferase	2.4.2.7	H16_A0395	apt	aicar + ppi <-> 5a4ic + prpp	aicar + ppi + h <-> 5a4ic + prpp	aicar + ppi + h <-> 5a4ic + prpp
ADPRT3	Purine metabolism	adenine phosphoribosyltransferase	2.4.2.7	H16_A0395	apt	gmp + ppi <-> gn + prpp	gmp + ppi + h <-> gn + prpp	gmp + ppi + h <-> gn + prpp
NUDPK9	Purine metabolism	nucleoside-diphosphate kinase (ATP:GDP)	2.7.4.6	H16_A2368	ndk	atp + didp <-> adp + ditp	atp + didp <-> adp + ditp	atp + didp <-> adp + ditp
NUDPK10	Purine metabolism	nucleoside-diphosphate kinase (ATP:GDP)	2.7.4.6	H16_A2368	ndk	atp + idp <-> adp + itp	atp + idp <-> adp + itp	atp + idp <-> adp + itp
ADPRT4	Purine metabolism	adenine phosphoribosyltransferase	2.4.2.8	H16_A3242	hprT	amp + ppi <-> ad + prpp	amp + ppi + h <-> ad + prpp	amp + ppi + h <-> ad + prpp
PYK1	Purine metabolism	pyruvate kinase	2.7.1.40	H16_A0567/H16_A3602/H16_B0961	pyk1/pyk2/pyk3	datp + pyr <-> dadp + pep	datp + pyr <-> dadp + pep	datp + pyr <-> dadp + pep
PYK2	Purine metabolism	pyruvate kinase	2.7.1.40	H16_A0567/H16_A3602/H16_B0961	pyk1/pyk2/pyk3	gtp + pyr <-> gdp + pep	gtp + pyr <-> gdp + pep	gtp + pyr <-> gdp + pep
PYK3	Purine metabolism	pyruvate kinase	2.7.1.40	H16_A0567/H16_A3602/H16_B0961	pyk1/pyk2/pyk3	dgtp + pyr <-> dgdp + pep	dgtp + pyr <-> dgdp + pep	dgtp + pyr <-> dgdp + pep
ADNK2	Purine metabolism	adenylate kinase	2.7.4.3	H16_A0603	adk	atp + damp <-> adp + dadp	atp + damp + h <-> adp + dadp	atp + damp <-> adp + dadp
BTPT1	Purine metabolism	bis(5'-nucleosidyl)-tetraphosphatase	3.6.1.17	H16_A3406	unknown	xp4g + h2o -> xtp + xmp	xp4g + h2o + h -> xtp + xmp	xp4g + h2o -> xtp + xmp
BTPT2	Purine metabolism	bis(5'-nucleosidyl)-tetraphosphatase	3.6.1.17	H16_A3406	unknown	appppa + h2o -> atp + amp	appppa + h2o -> atp + amp + h	appppa + h2o -> atp + amp + 2 h
ADNCYC1	Purine metabolism	adenylate cyclase	4.6.1.1	H16_A0674/H16_A0827/H16_A1791/H16_A1809/H16_B0376	unknown/cycR1/unknown/unknown/cycR2	gtp -> cgmp + ppi	gtp -> cgmp + ppi + h	gtp -> cgmp + ppi + h
AADT1	Purine metabolism	ATP adenylyltransferase	2.7.7.53	H16_A1656	unknown	aps + atp -> so4 + appppa + h	aps + atp -> so4 + appppa + h	aps + atp -> so4 + appppa

AADT2	Purine metabolism	ATP adenylyltransferase	2.7.7.53	H16_A1656	unknown	adp + atp -> pi + appppa	adp + atp -> pi + appppa + h	adp + atp + h -> pi + appppa
SADT1	Purine metabolism	sulfate adenylyltransferase	2.7.7.4	H16_A2995&H16_A2996&H16_B0626&H16_B0627	cysN1&cysD&cysN2&cysH2	atp + so4 + h -> ppi + aps	atp + so4 -> ppi + aps	atp + so4 -> ppi + aps
UREA	Purine metabolism	urease	3.5.1.5	H16_A1081/H16_A1083/H16_A1084	ureA/ureB/ureC	urea + h2o + 2 h -> co2 + 2 nh4	urea + h2o + 2 h -> co2 + 2 nh4	urea + h2o + 2 h -> co2 + 2 nh4
ALLTC	Purine metabolism	allantoicase	3.5.3.4	H16_B2460	unknown	alltt + h2o <-> urdglyc + urea	alltt + h2o <-> urdglyc + urea	alltt + h2o <-> urdglyc + urea
PAAD2	Purine metabolism	phenylacrylic acid decarboxylase	4.1.1.-	H16_B2447	unknown	a4ic + h -> amdz + co2	a4ic + h -> amdz + co2	a4ic + 2 h -> amdz + co2
ALLTNtr	Putative Transporters	allantoin transport in via proton symport				alltn_e + h_e <-> alltn + h	alltn_e + h_e <-> alltn + h	alltn_e + h_e <-> alltn + h
ARGORNt	Putative Transporters	arginine/ornithine antiporter				arg_e + orn <-> arg + orn_e	arg_e + orn <-> arg + orn_e	arg_e + orn <-> arg + orn_e
ACACt	Putative Transporters	acetoacetate transport via proton symport				acac_e + h_e <-> acac + h	acac_e + h_e <-> acac + h	acac_e + h_e <-> acac + h
BUTtr	Putative Transporters	Butyrate transport via proton symport, reversible				1boh_e + h_e <-> 1boh + h	1boh_e + h_e <-> 1boh + h	1boh_e + h_e <-> 1boh + h
CYNTt	Putative Transporters	Cyanate transport via proton symport				cynt_e + h_e -> cynt + h	cynt_e + h_e -> cynt + h	cynt_e + h_e -> cynt + h
GALCTtr	Putative Transporters	D-galactarte transport via proton symport, reversible				dgal_e + h_e <-> dgal + h	dgal_e + h_e <-> dgal + h	dgal_e + h_e <-> dgal + h
PPPNtr	Putative Transporters	3-phenylpropionate transport via proton symport, reversible				h_e + pppn_e <-> h + pppn	h_e + pppn_e <-> h + pppn	h_e + pppn_e <-> h + pppn
HPPPNtr	Putative Transporters	3-(3-hydroxyphenyl)propionate transport via proton symport, reversible				3hpppn_e + h_e <-> 3hpppn + h	3hpppn_e + h_e <-> 3hpppn + h	3hpppn_e + h_e <-> 3hpppn + h
HCINNMtr	Putative Transporters	3-hydroxycinnamic acid transport via proton symport, reversible				3hcinnm_e + h_e <-> 3hcinnm + h	3hcinnm_e + h_e <-> 3hcinnm + h	3hcinnm_e + h_e <-> 3hcinnm + h
GLUABUTt	Putative Transporters	4-aminobutyrate/glutamate antiport				gaba + glu_e <-> gaba_e + glu	gaba + glu_e <-> gaba_e + glu	gaba + glu_e <-> gaba_e + glu
ALAtr	Putative Transporters	L-alanine reversible transport via proton symport				ala_e + h_e <-> ala + h	ala_e + h_e <-> ala + h	ala_e + h_e <-> ala + h
URAtr	Putative Transporters	uracil transport in via proton symport, reversible				h_e + ura_e <-> h + ura	h_e + ura_e <-> h + ura	h_e + ura_e <-> h + ura
GLYBtr	Putative Transporters	Glycine betaine transport via proton symport, reversible				glyb_e + h_e <-> glyb + h	glyb_e + h_e <-> glyb + h	glyb_e + h_e <-> glyb + h
CHLabc	Putative Transporters	choline transport via ABC system				atp + choline_e + h2o -> adp + choline + pi	atp + choline_e + h2o -> adp + choline + h + pi	atp + choline_e + h2o -> adp + choline + h + pi
GLYBabc	Putative Transporters	Glycine betaine transport via ABC system				atp + glyb_e + h2o -> adp + glyb + pi	atp + glyb_e + h2o -> adp + glyb + h + pi	atp + glyb_e + h2o -> adp + glyb + h + pi
TARTRt	Putative Transporters	Tartrate/succinate antiporter				succ + tartr_e <-> succ_e + tartr	succ + tartr_e <-> succ_e + tartr	succ + tartr_e <-> succ_e + tartr
SUCCabc	Putative Transporters	Succinate transport via ABC system				atp + h2o + succ_e -> adp + pi + succ	atp + h2o + succ_e -> adp + h + pi + succ	atp + h2o + succ_e -> adp + h + pi + succ
GUAt2	Putative Transporters	guanine transport in via proton symport				gn_e + h_e -> gn + h	gn_e + h_e -> gn + h	gn_e + h_e -> gn + h
XANt2	Putative Transporters	xanthine transport in via proton symport				h_e + xan_e -> h + xan	h_e + xan_e -> h + xan	h_e + xan_e -> h + xan
THMDS	Pyrimidine metabolism	thymidylate synthase	2.1.1.45	H16_A2703	thyA	dump + mettthf -> dhf + dtmp	dump + metthf -> dhf + dtmp	dump + metthf -> dhf + dtmp
THMDPP	Pyrimidine metabolism	thymidine phosphorylase	2.4.2.4	H16_A2012	deoA	pi + thymd <-> dr1p + thym	pi + thymd <-> dr1p + thym	pi + thymd <-> dr1p + thym
URAPRT	Pyrimidine metabolism	uracil phosphoribosyltransferase	2.4.2.9	H16_A0918/H16_A2914	upp1/unknown	prpp + ura -> ppi + ump	prpp + ura -> ppi + ump + h	prpp + ura -> ppi + ump + h
CYTDK1	Pyrimidine metabolism	cytidylate kinase (CMP)	2.7.4.14	H16_A0797	cmk	atp + cmp <-> adp + cdp	atp + cmp + h <-> adp + cdp	atp + cmp <-> adp + cdp
CYTDK2	Pyrimidine metabolism	cytidylate kinase (dCMP)	2.7.4.14	H16_A0797	cmk	atp + dcmp <-> adp + dcdp	atp + dcmp + h <-> adp + dcdp	atp + dcmp <-> adp + dcdp
UMPK	Pyrimidine metabolism	UMP kinase	2.7.4.14	H16_A0797	cmk	atp + ump <-> adp + udp	atp + ump + h <-> adp + udp	atp + ump <-> adp + udp
NUDPK2	Pyrimidine metabolism	nucleoside-diphosphate kinase (ATP:UDP)	2.7.4.6	H16_A2368	ndk	atp + udp <-> adp + utp	atp + udp <-> adp + utp	atp + udp <-> adp + utp
NUDPK3	Pyrimidine metabolism	nucleoside-diphosphate kinase (ATP:CDP)	2.7.4.6	H16_A2368	ndk	atp + cdp <-> adp + ctp	atp + cdp <-> adp + ctp	atp + cdp <-> adp + ctp
NUDPK4	Pyrimidine metabolism	nucleoside-diphosphate kinase (ATP:dTDP)	2.7.4.6	H16_A2368	ndk	atp + dtdp <-> adp + dttp	atp + dtdp <-> adp + dttp	atp + dtdp <-> adp + dttp
NUDPK6	Pyrimidine metabolism	nucleoside-diphosphate kinase (ATP:dUDP)	2.7.4.6	H16_A2368	ndk	atp + dudp <-> adp + dutp	atp + dudp <-> adp + dutp	atp + dudp <-> adp + dutp
NUDPK7	Pyrimidine metabolism	nucleoside-diphosphate kinase (ATP:dCDP)	2.7.4.6	H16_A2368	ndk	atp + dcdp <-> adp + dctp	atp + dcdp <-> adp + dctp	atp + dcdp <-> adp + dctp
DTMPK	Pyrimidine metabolism	dTMP kinase	2.7.4.9	H16_A1569	tmk	atp + dtmp <-> adp + dtdp	atp + dtmp + h <-> adp + dtdp	atp + dtmp <-> adp + dtdp
NUTD1	Pyrimidine metabolism	5'-nucleotidase (dUMP)	3.1.3.5	H16_A2376	surE	dump + h2o -> du + pi	dump + h2o -> du + pi	dump + h2o -> du + pi
NUTD2	Pyrimidine metabolism	5'-nucleotidase (UMP)	3.1.3.5	H16_A2376	surE	h2o + ump -> pi + uri	h2o + ump -> pi + uri	h2o + ump -> pi + uri
NUTD3	Pyrimidine metabolism	5'-nucleotidase (dCMP)	3.1.3.5	H16_A2376	surE	dcmp + h2o -> dc + pi	dcmp + h2o -> dc + pi	dcmp + h2o -> dc + pi
NUTD4	Pyrimidine metabolism	5'-nucleotidase (CMP)	3.1.3.5	H16_A2376	surE	cmp + h2o -> cytd + pi	cmp + h2o -> cytd + pi	cmp + h2o -> cytd + pi
NUTD5	Pyrimidine metabolism	5'-nucleotidase (dTMP)	3.1.3.5	H16_A2376	surE	dtmp + h2o -> pi + thymd	dtmp + h2o -> pi + thymd	dtmp + h2o -> pi + thymd
CTDA	Pyrimidine metabolism	Cytosine deaminase	3.5.4.1	H16_B1593	codA	ct + h + h2o -> nh4 + ura	ct + h + h2o -> nh4 + ura	ct + h + h2o -> nh4 + ura
DCTPDA1	Pyrimidine metabolism	dCTP deaminase	3.5.4.13	H16_A2931	dcd	dctp + h + h2o -> dutp + nh4	dctp + h + h2o -> dutp + nh4	dctp + h + h2o -> dutp + nh4
DCTPDA2	Pyrimidine metabolism	dCTP deaminase	3.5.4.13	H16_A2931	dcd	ctp + h + h2o -> utp + nh4	ctp + h + h2o -> utp + nh4	ctp + h + h2o -> utp + nh4
DUTPDP	Pyrimidine metabolism	dUTP diphosphatase	3.6.1.23	H16_A3049	dut	dutp + h2o -> dump + ppi	dutp + h2o -> dump + ppi + 2 h	dutp + h2o -> dump + ppi + 2 h
DOURIP	Pyrimidine metabolism	deoxyuridine phosphorylase	2.4.2.4	H16_A2012	deoA	du + pi <-> dr1p + ura	du + pi <-> dr1p + ura	du + pi <-> dr1p + ura
URIDK1	Pyrimidine metabolism	uridylate kinase (dUMP)	2.7.4.9	H16_A1569	tmk	atp + dump <-> adp + dudp	atp + dump + h <-> adp + dudp	atp + dump <-> adp + dudp
DOROAD	Pyrimidine metabolism	dihydoorotic acid dehydrogenase	1.3.3.1	H16_A1401	pyrD	doroa + o2 -> oroa + h2o2	doroa + o2 -> oroa + h2o2	doroa + o2 -> oroa + h2o2
ASPCBT	Pyrimidine metabolism	aspartate carbamoyltransferase	2.1.3.2	H16_A2913	pyrB	asp + cap -> caasp + h + pi	asp + cap -> caasp + h + pi	asp + cap -> caasp + h + pi
OROPRT	Pyrimidine metabolism	orotate phosphoribosyltransferase	2.4.2.10	H16_A0224	pyrE	omp + ppi <-> oroa + prpp	omp + ppi + h <-> oroa + prpp	omp + ppi + h <-> oroa + prpp
DHORT	Pyrimidine metabolism	dihydroorotase	3.5.2.3	H16_A0479	pyrC	doroa + h2o <-> caasp + h	doroa + h2o <-> caasp + h	doroa + h2o <-> caasp + h

OMPDC	Pyrimidine metabolism	orotidine-5'-phosphate decarboxylase	4.1.1.23	H16_A3157	pyrF	h + omp -> co2 + ump	h + omp -> co2 + ump	h + omp -> co2 + ump
CTPS	Pyrimidine metabolism	CTP synthase (glutamine)	6.3.4.2			atp + gln + h2o + utp -> adp + ctp + glu + h + pi	atp + gln + h2o + utp -> adp + ctp + glu + 2 h + pi	atp + gln + h2o + utp -> adp + ctp + glu + 2 h + pi
URIDK2	Pyrimidine metabolism	uridylate kinase	2.7.4.22	H16_A2053	unknown	atp + ump <-> adp + udp	atp + ump + h <-> adp + udp	atp + ump <-> adp + udp
BTPT3	Pyrimidine metabolism	bis(5'-nucleosidyl)-tetraphosphatase	3.6.1.17	H16_A3406	unknown	up4g + h2o -> utp + ump	up4g + h2o -> utp + ump + h	up4g + h2o -> utp + ump + 2 h
PDRS	Pyrimidine metabolism	pseudouridylate synthase	4.2.1.70	H16_A3057	truA2	ura + r5p <-> puri5p + h2o	ura + r5p <-> puri5p + h2o	ura + r5p <-> puri5p + h2o
DHPM	Pyrimidine metabolism	dihydropyrimidinase	3.5.2.2	H16_A0068/H16_A3075	unknown/unknown	56dhu + h2o <-> 3urdpp + h	56dhu + h2o <-> 3urdpp + h	56dhu + h2o <-> 3urdpp + h
BUDPP1	Pyrimidine metabolism	beta-ureidopropionase	3.5.1.6	H16_A1475	unknown	3urdpp + h2o + 2 h -> bala + co2 + nh4	3urdpp + h2o + 2 h -> bala + co2 + nh4	3urdpp + h2o + 2 h -> bala + co2 + nh4
DCDA	Pyrimidine metabolism	dCMP deaminase	3.5.4.12	H16_B0797	unknown	dcmp + h2o + h -> dump + nh4	dcmp + h2o + h -> dump + nh4	dcmp + h2o + h -> dump + nh4
CYTDA	Pyrimidine metabolism	cytosine deaminase	3.5.4.1	H16_A0782/H16_B1593	ssnA/codA	5mc + h2o + h <-> thym + nh4	5mc + h2o + h <-> thym + nh4	5mc + h2o + h <-> thym + nh4
DHPMD	Pyrimidine metabolism	dihydropyrimidinase	3.5.2.2	H16_A0068/H16_A3075	unknown/unknown	56dht + h2o <-> 3udsb + h	56dht + h2o <-> 3udsb + h	56dht + h2o <-> 3udsb + h
BUDPP2	Pyrimidine metabolism	beta-ureidopropionase	3.5.1.6	H16_A1475	unknown	3udsb + h2o + 2 h -> 3aibt + co2 + nh4	3udsb + h2o + 2 h -> 3aibt + co2 + nh4	3udsb + h2o + 2 h -> 3aibt + co2 + nh4
ALHD1	Pyruvate Metabolism	aldehyde dehydrogenase (acetaldehyde, NAD)	1.2.1.3	H16_A0232/H16_A0745/H16_A1114/H16_A1495/H16_B0212/H16_B0421/H16_B0737/H16_B0833/H16_B1534/H16_B1735/H16_B1751/H16_B1835/H16_B1960/H16_B2444	unknown/unknown/unknown/unknown/unknown/unknown/unknown/unknown/unknown/unknown/unknown/unknown/exaC/unknown/	acal + h2o + nad -> ac + 2 h + nadh	acal + h2o + nad -> ac + 2 h + nadh	acal + h2o + nad -> ac + 2 h + nadh
PPS	Pyruvate Metabolism	phosphoenolpyruvate synthase	2.7.9.2	H16_A2038	ppsA	atp + h2o + pyr -> amp + pep + pi	atp + h2o + pyr -> amp + 2 h + pep + pi	atp + h2o + pyr -> amp + h + pep + pi
ALDRD1	Pyruvate Metabolism	aldehyde reductase	1.1.1.21	H16_A3186/H16_B2162	unknown/unknown	h + mtg + nadh -> dlald + nad	h + mtg + nadh -> dlald + nad	h + mtg + nadh -> dlald + nad
ALDRD2	Pyruvate Metabolism	aldehyde reductase	1.1.1.21	H16_A3186/H16_B2162	unknown/unknown	2 h + dlald + nadph <-> 12ppd-R + nadp	h + dlald + nadph <-> 12ppd-R + nadp	h + dlald + nadph <-> 12ppd-R + nadp
HAGTH	Pyruvate Metabolism	hydroxyacylglutathione hydrolase	3.1.2.6	H16_A0190/H16_A1980/H16_A2209/H16_A2464/H16_A3582/H16_A3623/	gloB1/unknown/unknown/gloB2/gloB3/unknown	h2o + ltg -> rgt + h + lac	h2o + ltg -> rgt + h + lac	h2o + ltg -> rgt + h + lac
MGXS	Pyruvate Metabolism	methylglyoxal synthase	4.2.3.3	H16_A0932	mgsA	dhap -> mtg + pi	dhap -> mtg + pi	dhap -> mtg + pi
LGTTHL	Pyruvate Metabolism	lactoylglutathione lyase	4.4.1.5	H16_A0274/H16_A0517/H16_A2179/H16_B1143/H16_B2255	unknown/gloA1/unknown/gloA2/gloA5	rgt + mtg -> ltg	rgt + mtg -> ltg	rgt + mtg -> ltg
ALCDet	Pyruvate Metabolism	alcohol dehydrogenase (ethanol)	1.1.1.1	H16_A0757/H16_A3330/H16_B0517/H16_B1433/H16_B1699/H16_B1745/H16_B1834/H16_B2470	adh/unknown/unknown/adhP/unknown/unknown/unknown/unknown	eth + nad <-> acal + h + nadh	eth + nad <-> acal + h + nadh	eth + nad <-> acal + h + nadh
DLDHD	Pyruvate Metabolism	D-lactate dehydrogenase	1.1.1.28	H16_A1681/H16_A1682	ldhA1/ldhA2	lac + nad <-> h + nadh + pyr	lac + nad <-> h + nadh + pyr	lac + nad <-> h + nadh + pyr
ACALDDH	Pyruvate Metabolism	acetaldehyde dehydrogenase (acetylating)	1.2.1.10	H16_A1806/H16_A2747/H16_B0551/H16_B0596	unknown/unknown/mhpF/unknown	acal + coa + nad <-> accoa + h + nadh	acal + coa + nad <-> accoa + h + nadh	acal + coa + nad <-> accoa + h + nadh
LCTAD2	Pyruvate Metabolism	lactaldehyde dehydrogenase	1.2.1.22	H16_A1919	unknown	mtg + nad + h2o <-> pyr + nadh + 2 h	mtg + nad + h2o <-> pyr + nadh + 2 h	mtg + nad + h2o <-> pyr + nadh + 2 h
PTA	Pyruvate Metabolism	phosphotransacetylase	2.3.1.8	H16_B1631/H16_B1871	pta1/pta2	accoa + pi <-> actp + coa + h	accoa + pi <-> actp + coa	accoa + pi <-> actp + coa
ACKA	Pyruvate Metabolism	acetate kinase	2.7.2.1	H16_A0670/H16_B1630	ackA2/ackA	ac + atp <-> actp + adp	ac + atp <-> actp + adp	ac + atp <-> actp + adp
PCT1	Pyruvate Metabolism	propionate CoA-transferase	2.8.3.1	H16_A2718	pct	accoa + ppa <-> ac + ppcoa	accoa + ppa <-> ac + ppcoa	accoa + ppa <-> ac + ppcoa
APPS2	Pyruvate Metabolism	acylphosphatase	3.6.1.7	H16_A3325	acyP	actp + h2o -> ac + pi	actp + h2o -> ac + pi + h	actp + h2o -> ac + pi + h
ACS	Pyruvate Metabolism	acetyl-CoA synthetase	6.2.1.1	H16_A1616/H16_A2525/H16_B0386/H16_B0591/H16_B0696/H16_B0834/H16_B1102	unknown/acoE/unknown/unknown/unknown/unknown/unknown	ac + atp + coa + h -> accoa + amp + ppi	ac + atp + coa -> accoa + amp + ppi + h	ac + atp + coa -> accoa + amp + ppi + h
PYC	Pyruvate metabolism	pyruvate carboxylase	6.4.1.1	H16_A1251/H16_A2142	pyc/accA2	atp + pyr + hco3 <-> adp + oaa + pi + h	atp + pyr + hco3 <-> adp + oaa + pi + h	atp + pyr + hco3 <-> adp + oaa + pi + h
PAAD1	Pyruvate Metabolism	phenylacrylic acid decarboxylase	4.1.1.-	H16_B2447	unknown	hedc + h -> pyr + co2	hedc + h -> pyr + co2	hedc + h -> pyr + co2
ALHD1p	Pyruvate Metabolism	aldehyde dehydrogenase (acetaldehyde, NAD)	1.2.1.3	H16_A0232/H16_A0745/H16_A1114/H16_A1495/H16_B0212/H16_B0421/H16_B0737/H16_B0833/H16_B1534/H16_B1735/H16_B1751/H16_B1835/H16_B1960/H16_B2444	unknown/unknown/unknown/unknown/unknown/unknown/unknown/unknown/unknown/unknown/unknown/unknown/exaC/unknown/	acal + h2o + nadp -> ac + 3 h + nadph	acal + h2o + nadp -> ac + 2 h + nadph	acal + h2o + nadp -> ac + 2 h + nadph
A6PRAUR	Rivoflavin metabolism	5-amino-6-(5-phosphoribosylamino)uracil reductase	1.1.1.193	H16_A2848	ribD	a6rp5p + 2 h + nadph -> a6rp5p2 + nadp	a6rp5p + h + nadph -> a6rp5p2 + nadp	a6rp5p + h + nadph -> a6rp5p2 + nadp
FLVRp	Rivoflavin Metabolism	flavin reductase	1.5.1.30	H16_A2352/H16_B0671	unknown/unknown	2 h + nadph + ribflav -> nadp + rbflvrd	2 h + nadph + ribflav -> nadp + rbflvrd	2 h + nadph + ribflav -> nadp + rbflvrd
FLVR	Rivoflavin Metabolism	flavin reductase (NAD)	1.5.1.30	H16_A2352/H16_B0671	unknown/unknown	h + nadh + ribflav -> nad + rbflvrd	2 h + nadh + ribflav -> nad + rbflvrd	2 h + nadh + ribflav -> nad + rbflvrd
RBFS1	Rivoflavin metabolism	riboflavin synthase	2.5.1.9	H16_A2855	ribH	a6rp + db4p -> dmlz + 2 h2o + pi	a6rp + db4p -> dmlz + h + 2 h2o + pi	a6rp + db4p -> dmlz + h + 2 h2o + pi
RBFS2	Rivoflavin metabolism	riboflavin synthase	2.5.1.9	H16_A2849	ribC	2 dmlz -> a6rp + ribflav	2 dmlz + h -> a6rp + ribflav	2 dmlz + h -> a6rp + ribflav
APPT	Rivoflavin metabolism	acid phosphatase	3.1.3.2	H16_A0767/H16_B1238	pgpB/unknown	fmn + h2o -> ribflav + pi	fmn + h2o -> ribflav + pi	fmn + h2o -> ribflav + pi
RBFK	Rivoflavin metabolism	riboflavin kinase	2.7.1.26	H16_A3045	unknown	atp + ribflav -> adp + fmn	atp + ribflav -> adp + fmn + h	atp + ribflav -> adp + fmn + h
FMNANT	Rivoflavin metabolism	FMN adenylyltransferase	2.7.7.2	H16_A3045	unknown	atp + fmn -> fad + ppi	atp + fmn -> fad + ppi	atp + fmn -> fad + ppi
DHPPRAP	Rivoflavin metabolism	diaminohydroxyphosphoribosylaminopryrimidine deaminase (25drapp)	3.5.4.26	H16_A2848	ribD	25drapp + h + h2o -> a6rp5p + nh4	25drapp + h + h2o -> a6rp5p + nh4	25drapp + h + h2o -> a6rp5p + nh4
DHB4PS	Rivoflavin metabolism	3,4-Dihydroxy-2-butanone-4-phosphate synthase	4.1.2.-	H16_A2854/H16_B0632/H16_B1223	ribBA/unknown/unknown	rl5p -> db4p + formate + h	rl5p -> db4p + formate + h	rl5p -> db4p + formate + h
GTPCHII	Rivoflavin metabolism	GTP cyclohydrolase II	3.5.4.25	H16_B1576	ribA	gtp + 3 h2o -> 25drapp + formate + ppi + h	gtp + 3 h2o -> 25drapp + formate + ppi + 3 h	gtp + 3 h2o -> 25drapp + formate + ppi + 3 h
NNDMBZPT	Rivoflavin metabolism	Nicotinate-nucleotide dimethylbenzimidazole phosphoribosyltransferase	2.4.2.21	H16_A2968	cobT1	dmbzid + nacn -> 5prdmbz + 2 h + nac	dmbzid + nacn -> 5prdmbz + h + nac	dmbzid + nacn -> 5prdmbz + h + nac
PMPP	Rivoflavin metabolism	pyrimidine phosphatase	3.1.3.-	H16_A0520/H16_A0786/H16_A2434/H16_A2577/H16_B0594/H16_B1063/H16_B2398	unknown/unknown/sixA/unknown/unknown/unknown/unknown	a6rp5p2 + h2o -> a6rp + pi	a6rp5p2 + h2o -> a6rp + pi	a6rp5p2 + h2o -> a6rp + pi
RZ5PP	Rivoflavin metabolism	alpha-ribazole 5-phosphate phosphatase		H16_A0520/H16_A0786/H16_A2434/H16_A2577/H16_B0594/H16_B1063/H16_B2398	unknown/unknown/sixA/unknown/unknown/unknown/unknown	5prdmbz + h2o -> pi + rdmbzi	5prdmbz + h2o -> pi + rdmbzi	5prdmbz + h2o -> pi + rdmbzi
SELNPS	Selenoamino acid metabolism	Selenophosphate synthase	2.7.9.3	H16_B0295	selD	atp + h2o + seld -> amp + pi + selnp	atp + h2o + seld -> amp + pi + selnp + 2 h	atp + h2o + seld -> amp + pi + selnp + h
CYSGS1	Selenoamino acid metabolism	cystathionine gamma-synthase	2.5.1.48	H16_A2606	metB	ahser + scys -> sllct + ac + h	ahser + scys -> sllct + ac + h	ahser + scys -> sllct + ac + h

CYSGS2	Selenoamino acid metabolism	cystathionine gamma-synthase	2.5.1.48	H16_A2606	metB	shser + scys -> sllct + succ + h	shser + scys -> sllct + succ + h	shser + scys -> sllct + succ + h
CYSTBL3	Selenoamino acid metabolism	cystathionine beta-lyase	4.4.1.8	H16_A1447	metC	sllct + h2o -> shcys + nh4 + pyr	sllct + h2o -> shcys + nh4 + pyr	sllct + h2o -> shcys + nh4 + pyr
ADHC2	Selenoamino acid metabolism	adenosylhomocysteinase	3.3.1.1	H16_A0244	ahcY	seadseh + h2o -> adn + shcys	seadseh + h2o -> adn + shcys	seadseh + h2o + h -> adn + shcys
SADMET	Selenoamino acid metabolism	S-adenosylmethionine synthetase	2.5.1.6	H16_A0230/H16_A1975	metK1/metK2	atp + smet + h2o + h -> pi + ppi + seasmet	atp + smet + h2o -> pi + ppi + seasmet + 2 h	atp + smet + h2o -> pi + ppi + seasmet + 3 h
METTRSS	Selenoamino acid metabolism	methionyl-tRNA synthetase	6.1.1.10	H16_A2945	metG	atp + smet + trnamet -> amp + ppi + selmtrna	atp + smet + trnamet -> amp + ppi + selmtrna	atp + smet + trnamet -> amp + ppi + selmtrna
CYSST4	Selenoamino acid metabolism	cysteine synthase	2.5.1.47	H16_A0807	cysK1	aser + seld -> scys + ac	aser + seld -> scys + ac	aser + seld -> scys + ac
SULFR	Selenoamino acid metabolism	sulfite reductase (NADPH)	1.8.1.2	H16_A1639&H16_A2999&H16_B2500	cysI2&cysI1&unknown	selt + 3 nadp + 7 h2 -> seld + 3 nadph + 3 h2o + 7 h	selt + 3 nadp + 7 h2 -> seld + 3 nadph + 3 h2o + 4 h	selt + 3 nadp + 7 h2 -> seld + 3 nadph + 3 h2o + 4 h
ADSLFK2	Selenoamino acid metabolism	adenylyl-sulfate kinase	2.7.1.25	H16_B0626	cysN2	atp + aselnt + h -> adp + ppadsel	atp + aselnt -> adp + ppadsel	atp + aselnt -> adp + ppadsel + h
G1PCYTF	Starch and sucrose metabolism	glucose-1-phosphate cytidylyltransferase	2.7.7.33	H16_A2893	rfbF	ctp + g1p <-> ppi + cdpglc	ctp + g1p <-> ppi + cdpglc	ctp + g1p <-> ppi + cdpglc
CDPGD	Starch and sucrose metabolism	CDP-glucose 4,6-dehydratase	4.2.1.45	H16_A2896	unknown	cdpglc -> cdpddglc + h2o	cdpglc -> cdpddglc + h2o	cdpglc -> cdpddglc + h2o
TRHPS	Starch and sucrose metabolism	alpha,alpha-trehalose-phosphate synthase (UDP-forming)	2.4.1.15	H16_A0430	unknown	g6p + udpg -> tre6p + udp	g6p + udpg -> tre6p + udp	g6p + udpg -> tre6p + udp + h
AMMALT1	Starch and sucrose metabolism	Amylomaltase (maltotriose)	2.4.1.25	H16_B1561	malQ	mlt + mlttr -> glc + mltttr	mlt + mlttr -> glc + mltttr	mlt + mlttr -> glc + mltttr
AMMALT2	Starch and sucrose metabolism	Amylomaltase (maltotetraose)	2.4.1.25	H16_B1561	malQ	mlt + mltttr -> glc + maltpt	mlt + mltttr -> glc + maltpt	mlt + mltttr -> glc + maltpt
AMMALT3	Starch and sucrose metabolism	Amylomaltase (maltopentaose)	2.4.1.25	H16_B1561	malQ	mlt + maltpt -> glc + mlthx	mlt + maltpt -> glc + mlthx	mlt + maltpt -> glc + mlthx
AMMALT4	Starch and sucrose metabolism	Amylomaltase (maltohexaose)	2.4.1.25	H16_B1561	malQ	mlt + mlthx -> glc + malthp	mlt + mlthx -> glc + malthp	mlt + mlthx -> glc + malthp
TRH6PP	Starch and sucrose metabolism	trehalose-phosphatase	3.1.3.12	H16_A0428	otsB	h2o + tre6p -> pi + tre	h2o + tre6p -> pi + tre	h2o + tre6p -> pi + tre
TREHL	Starch and sucrose metabolism	alpha,alpha-trehalase	3.2.1.28	H16_B2096	treA	h2o + tre -> 2 glc	h2o + tre -> 2 glc	h2o + tre -> 2 glc
GLCGP	Starch and sucrose metabolism	glycogen phosphorylase	2.4.1.1	H16_B1562	glgP	glycogen + pi -> g1p	glycogen + pi -> g1p	glycogen + pi -> g1p
GLCBRAN	Starch and sucrose metabolism	1,4-alpha-glucan branching enzyme (glycogen -> bglycogen)	2.4.1.18	H16_B1559	glgB	glycogen -> bglycogen	glycogen -> bglycogen	glycogen -> bglycogen
MLTGCT	Starch and sucrose metabolism	maltose alpha-D-glucosyltransferase	5.4.99.16	H16_B1558/H16_B1564	unknown/treY	tre_e <-> mlt_e	tre_e <-> mlt_e	tre_e <-> mlt_e
NITL4	Styrene degradation	nitrilase	3.5.5.1	H16_A1125	nit	pheacnit + 2 h2o -> pac + nh4	pheacnit + 2 h2o -> pac + nh4	pheacnit + 2 h2o -> pac + nh4
AMDS4	Styrene degradation	amidase	3.5.1.4	H16_A1469/H16_B1874/H16_B2459	unknown/unknown/aimE	pheact + h2o -> pac + nh4	pheact + h2o -> pac + nh4	pheact + h2o -> pac + nh4
PHEALDD	Styrene degradation	phenylacetaldehyde dehydrogenase	1.2.1.39	H16_B1358/H16_B1939	paaK2/feaB	pacald + nad + h2o -> pac + nadh + 2 h	pacald + nad + h2o -> pac + nadh + 2 h	pacald + nad + h2o -> pac + nadh + 2 h
NTPPD5	Styrene degradation	2-nitropropane dioxygenase	1.13.11.-	H16_A0633/H16_B0223/H16_B0757/H16_B1109/H16_B1420/H16_B1836	pcaH2/unknown/unknown/unknown/unknown/unknown	styrene + o2 + nadh + h -> strcg + nad	styrene + o2 + nadh + h -> strcg + nad	styrene + o2 + nadh + h -> strcg + nad
CATCHD3	Styrene degradation	catechol 2,3-dioxygenase	1.13.11.2	H16_B0546	unknown	3vcat + o2 -> 2h6ot + h	3vcat + o2 -> 2h6ot + h	3vcat + o2 -> 2h6ot + h
ALPNIT	Styrene degradation	aliphatic nitrilase	3.5.5.7	H16_A1956	unknown	aconit + 2 h2o -> propen + nh4	aconit + 2 h2o -> propen + nh4	aconit + 2 h2o -> propen + nh4
AMDS5	Styrene degradation	amidase	3.5.1.4	H16_A1469/H16_B1874/H16_B2459	unknown/unknown/aimE	aclm + h2o -> propen + nh4	aclm + h2o -> propen + nh4	aclm + h2o -> propen + nh4
PCT3	Styrene degradation	propionate CoA-transferase	2.8.3.1	H16_A2718	pct	lactcoa + ac -> llac + accoa	lactcoa + ac -> llac + accoa	lactcoa + ac -> llac + accoa
PAC2H	Styrene degradation	phenylacetate 2-hydroxylase	1.14.13.-			pac + o2 + nadh + h -> 2hpa + nad + h2o	pac + o2 + nadh + h -> 2hpa + nad + h2o	pac + o2 + nadh + h -> 2hpa + nad + h2o
2HPAC	Styrene degradation	2-hydroxy-phenylacetate hydroxylase	1.14.13.-			2hpa + o2 + nadh + h -> homogen + nad + h2o	2hpa + o2 + nadh + h -> homogen + nad + h2o	2hpa + o2 + nadh + h -> homogen + nad + h2o
SLFR	Sulfur Metabolism	sulfite reductase	1.8.1.2	H16_A1639&H16_A2999&H16_B2500	cysI2&cysI1&unknown	7 h + 3 nadph + so3 -> 3 h2o + h2s + 3 nadp	3 h + 3 nadph + so3 -> 3 h2o + h2s + 3 nadp	3 h + 3 nadph + so3 -> 3 h2o + h2s + 3 nadp
PASR1	Sulfur Metabolism	phosphoadenylyl-sulfate reductase (thioredoxin)	1.8.4.8	H16_A2997	cysH	paps + rthio -> pap + so3 + othio	paps + rthio -> pap + so3 + othio + h	paps + rthio -> pap + so3 + othio + h
PASR2	Sulfur Metabolism	phosphoadenylyl-sulfate reductase (glutaredoxin)	1.8.4.8	H16_A2997	cysH	grxrd + paps -> grxox + pap + so3	grxrd + paps -> grxox + pap + so3 + h	grxrd + paps -> grxox + pap + so3 + h
ADSLFK1	Sulfur Metabolism	adenylyl-sulfate kinase	2.7.1.25	H16_B0626	cysN2	aps + atp -> adp + paps	aps + atp -> adp + paps + h	aps + atp -> adp + paps + h
BPNT	Sulfur Metabolism	3',5'-bisphosphate nucleotidase	3.1.3.7			h2o + pap -> amp + pi	h2o + pap -> amp + pi	h2o + pap -> amp + pi
TAUDO	Taurine and Hypotaurine metabolism	Taurine dioxygenase	1.14.11.17	H16_A0037/H16_A0038/H16_A1263/H16_B0422/H16_B1004/H16_B1034/H16_B1533/H16_B2220/H16_B2227/H16_B2521	unknown/unknown/unknown/unknown/unknown/unknown/unknown/tauD1/tauD2/unknown	akg + o2 + taur -> aacald + co2 + so3 + succ	akg + o2 + taur -> aacald + co2 + so3 + succ	akg + o2 + taur -> aacald + co2 + so3 + succ + h
ALADH	Taurine and Hypotaurine metabolism	alanine dehydrogenase	1.4.1.1	H16_A2009	ald	ala + nad + h2o -> pyr + nh4 + nadh + h	ala + nad + h2o -> pyr + nh4 + nadh + h	ala + nad + h2o -> pyr + nh4 + nadh + h
SULALDAC	Taurine and Hypotaurine metabolism	sulfoacetaldehyde acetyltransferase	2.3.3.15	H16_B1870	xsc	sulald + pi -> actp + so3 + h	sulald + pi -> actp + so3	sulald + pi -> actp + so3
THMPDP	Thiamine Metabolism	thiamine-phosphate diphosphorylase	2.5.1.3	H16_A0239	thiE	ahmpp + thzp -> ppi + thmp	ahmpp + thzp -> ppi + thmp	ahmpp + thzp -> ppi + thmp
HMPMK	Thiamine Metabolism	hydroxymethylpyrimidine kinase (ATP)	2.7.1.49	H16_A0243	unknown	ahm + atp -> 4ampm + adp	ahm + atp -> 4ampm + adp + h	ahm + atp -> 4ampm + adp + h
HETHZK	Thiamine Metabolism	hydroxyethylthiazole kinase	2.7.1.50			4mhetz + atp -> thzp + adp	4mhetz + atp -> thzp + adp + h	4mhetz + atp -> thzp + adp + h
THMPK	Thiamine Metabolism	thiamine-phosphate kinase	2.7.4.16	H16_A3154	thiL	atp + thmp -> adp + thmpp	atp + thmp + h -> adp + thmpp	atp + thmp -> adp + thmpp
PMPMK	Thiamine Metabolism	phosphomethylpyrimidine kinase	2.7.4.7	H16_A024	unknown	4ampm + atp -> ahmpp + adp	4ampm + atp + h -> ahmpp + adp	4ampm + atp -> ahmpp + adp
THMPT	Thiamine Metabolism	phosphatase	3.1.3.-	H16_A0520/H16_A0786/H16_A2434/H16_A2577/H16_B0594/H16_B1063/H16_B2398	unknown/unknown/six/unknown/unknown/unknown/unknown	thiamin + pi <-> thmp + h2o	thiamin + pi <-> thmp + h2o	thiamin + pi <-> thmp + h2o
THMDP	Thiamine Metabolism	thiamin pyrophosphatase	3.6.1.15	H16_A0948	unknown	h2o + thmpp -> pi + thmp	h2o + thmpp -> 2 h + pi + thmp	h2o + thmpp -> h + pi + thmp
OXGTDC	Thiamine Metabolism	2-oxoglutarate decarboxylase	4.1.1.71			akg + h + thmpp -> co2 + ssaltpp	akg + h + thmpp -> co2 + ssaltpp	akg + 2 h + thmpp -> co2 + ssaltpp
THMB	Thiamine metabolism	thiamine biosynthesis protein ThiC	unclear reaction	H16_A0235	thiC	air -> ahm	air -> ahm	air -> ahm
GLYCOX	Thiamine metabolism	glycine oxidase	1.4.3.19	H16_A0236	thiO	gly -> imgly + h + h2	gly -> imgly + h + h2	gly -> imgly + h + h2
BZFORCL1	Toluene and Xylene degradation	benzoylformate carboxy-lyase	4.1.1.7	H16_A1113	unknown	aobzac + h -> bzald + co2	aobzac + h -> bzald + co2	aobzac + h -> bzald + co2
BZALDD1	Toluene and Xylene degradation	benzaldehyde dehydrogenase (NAD)	1.2.1.28	H16_A1772	unknown	bzald + nad + h2o -> benzot + nadh + 2 h	bzald + nad + h2o -> benzot + nadh + 2 h	bzald + nad + h2o -> benzot + nadh + 2 h
PHE2MO1	Toluene and Xylene degradation	phenol 2-monooxygenase	1.14.13.7	H16_B0539&H16_B0540&H16_B0541&H16_B0542&H16_B0543&H16_B0544	poxA&poxB&poxC&poxD&poxE&poxF	tolen + o2 + nadph + 2 h -> ocresol + nadp + h2o	tolen + o2 + nadph + h -> ocresol + nadp + h2o	tolen + o2 + nadph + h -> ocresol + nadp + h2o

PHE2MO2	Toluene and Xylene degradation	phenol 2-monooxygenase	1.14.13.7	H16_B0539&H16_B0540&H16_B0541&H16_B0542&H16_B0543&H16_B0544	poxA&poxB&poxC&poxD&poxE&poxF	ocresol + o2 + nadph + 2 h -> dhtolen + nadp + h2o	ocresol + o2 + nadph + h -> dhtolen + nadp + h2o	ocresol + o2 + nadph + h -> dhtolen + nadp + h2o
PHE2MO3	Toluene and Xylene degradation	phenol 2-monooxygenase	1.14.13.7	H16_B0539&H16_B0540&H16_B0541&H16_B0542&H16_B0543&H16_B0544	poxA&poxB&poxC&poxD&poxE&poxF	3cresol + o2 + nadph + 2 h -> dhtolen + nadp + h2o	3cresol + o2 + nadph + h -> dhtolen + nadp + h2o	3cresol + o2 + nadph + h -> dhtolen + nadp + h2o
CATCHD1	Toluene and Xylene degradation	catechol 2,3-dioxygenase	1.13.11.2	H16_B0546	unknown	dhtolen + o2 -> hkhdn + h	dhtolen + o2 -> hkhdn + h	dhtolen + o2 -> hkhdn + h
FBMO6	Toluene and Xylene degradation	flavin-binding monooxygenase	1.14.13.-	H16_A1145/H16_B0495/H16_B1480/H16_B2135	unknown/unknown/unknown/unknown	3cresol + o2 + nadh + h -> hbzal + nad + h2o	3cresol + o2 + nadh + h -> hbzal + nad + h2o	3cresol + o2 + nadh + h -> hbzal + nad + h2o
BZALDD2	Toluene and Xylene degradation	benzaldehyde dehydrogenase (NAD)	1.2.1.28	H16_A1772	unknown	3hbzald + nad + h2o -> 3hbenzot + nadh + 2 h	3hbzald + nad + h2o -> 3hbenzot + nadh + 2 h	3hbzald + nad + h2o -> 3hbenzot + nadh + 2 h
BZALDD3	Toluene and Xylene degradation	benzaldehyde dehydrogenase (NAD)	1.2.1.28	H16_A1772	unknown	mbzald + nadp + h2o -> otolat + nadph + 3 h	mbzald + nadp + h2o -> otolat + nadph + 2 h	mbzald + nadp + h2o -> otolat + nadph + 2 h
BZALDD4	Toluene and Xylene degradation	benzaldehyde dehydrogenase (NAD)	1.2.1.28	H16_A1772	unknown	mtolald + nadp + h2o -> mtolat + nadph + 3 h	mtolald + nadp + h2o -> mtolat + nadph + 2 h	mtolald + nadp + h2o -> mtolat + nadph + 2 h
BZALDD5	Toluene and Xylene degradation	benzaldehyde dehydrogenase (NAD)	1.2.1.28	H16_A1772	unknown	ptolald + nadp + h2o -> ptolat + nadph + 3 h	ptolald + nadp + h2o -> ptolat + nadph + 2 h	ptolald + nadp + h2o -> ptolat + nadph + 2 h
FBMO7	Toluene and Xylene degradation	flavin-binding monooxygenase	1.14.13.-	H16_A1145/H16_B0495/H16_B1480/H16_B2135	unknown/unknown/unknown/unknown	2 tol4sul + o2 + 2 h2 -> 2 4cresol + 2 so3	2 tol4sul + o2 + 2 h2 -> 2 4cresol + 2 so3	2 tol4sul + o2 + 2 h2 -> 2 4cresol + 2 so3
CATCHDG1	Toluene and Xylene degradation	catechol 1,2-dioxygenase	1.13.11.1	H16_A1964/H16_B0968	catA/pcpA	4mctch + o2 -> 3mhdd + 2 h	4mctch + o2 -> 3mhdd + 2 h	4mctch + o2 -> 3mhdd + 2 h
MCCIS1	Toluene and Xylene degradation	muconate cycloisomerase	5.5.1.1	H16_A1966/H16_B0536	catB3/catB4	3mhdd + h -> 4mmclac	3mhdd + h -> 4mmclac	3mhdd + h -> 4mmclac
CATCHD2	Toluene and Xylene degradation	catechol 2,3-dioxygenase	1.13.11.2	H16_B0546	unknown	4mctch + o2 -> hmcmsald + h	4mctch + o2 -> hmcmsald + h	4mctch + o2 -> hmcmsald + h
HMSALD2	Toluene and Xylene degradation	2-hydroxymuconic semialdehyde dehydrogenase	1.2.1.32	H16_B0547	unknown	hmcmsald + nad + h2o -> hmcmct + nadh + 2 h	hmcmsald + nad + h2o -> hmcmct + nadh + 2 h	hmcmsald + nad + h2o -> hmcmct + nadh + 2 h
KPENH	Toluene and Xylene degradation	2-keto-4-pentenoate hydratase	4.2.1.80	H16_B0548/H16_B0597/H16_B0884	mhpD1/bphH/mhpD3	hchdn + h2o -> hohx	hchdn + h2o -> hohx	hchdn + h2o -> hohx
OXCTD1	Toluene and Xylene degradation	4-oxalocrotonate decarboxylase	4.1.1.77	H16_B0549	unknown	omcmc + h -> hchdn + co2	omcmc + h -> hchdn + co2	omcmc + h -> hchdn + co2
4CRESOLD	Toluene and xylene degradation	4-cresol dehydrogenase (hydroxylating)	1.17.99.1			4cresol + fad -> 4hbzald + fadh2	4cresol + fad + h -> 4hbzald + fadh2	4cresol + fad -> 4hbzald + fadh2
NITRDT1	Trinitrotoluene degradation	nitroreductase	1.-.-.-	H16_A1789	unknown	tnittol + 2 nadh + 2 h -> 4hlmdnit + 2 nad + h2o	tnittol + 2 nadh + 2 h -> 4hlmdnit + 2 nad + h2o	tnittol + 2 nadh + 2 h -> 4hlmdnit + 2 nad + h2o
NITRDT2	Trinitrotoluene degradation	nitroreductase	1.-.-.-	H16_A1789	unknown	tnittol + 2 nadh + 2 h -> 2hlmdnit + 2 nad + h2o	tnittol + 2 nadh + 2 h -> 2hlmdnit + 2 nad + h2o	tnittol + 2 nadh + 2 h -> 2hlmdnit + 2 nad + h2o
AAATT	Trinitrotoluene degradation	arylamine N-acetyltransferase	2.3.1.5	H16_B1241	nhoA	24danit + accoa -> 4aanit + coa	24danit + accoa -> 4aanit + coa	24danit + accoa -> 4aanit + coa
NITRDT1p	Trinitrotoluene degradation	nitroreductase	1.-.-.-	H16_A1789	unknown	tnittol + 2 nadph + 4 h -> 4hlmdnit + 2 nadp + h2o	tnittol + 2 nadph + 2 h -> 4hlmdnit + 2 nadp + h2o	tnittol + 2 nadph + 2 h -> 4hlmdnit + 2 nadp + h2o
TRPPA	Tryptophan metabolism	Tryptophanase (L-tryptophan)	4.1.99.1			h2o + trp <-> indole + nh4 + pyr	h2o + trp <-> indole + nh4 + pyr	h2o + trp <-> indole + nh4 + pyr
MNAO10	Tryptophan metabolism	monoamine oxidase	1.4.3.4	H16_A0831	maoB	5hknm + o2 -> 46dhqn + nh4 + h2o2	5hknm + o2 -> 46dhqn + nh4 + h2o2	5hknm + o2 -> 46dhqn + nh4 + h2o2
ACFM2	Tryptophan metabolism	arylformamidase	3.5.1.9	H16_A3005/H16_B1997	unknown/unknown	5hnfkn + h2o -> 5hknn + formate + h	5hnfkn + h2o -> 5hknn + formate + h	5hnfkn + h2o -> 5hknn + formate + h
LAAO6	Tryptophan metabolism	L-amino-acid oxidase	1.4.3.2	H16_A0845/H16_A0856	lao1/lao2	trp + h2o + o2 -> idpyr + nh4 + h2o2	trp + h2o + o2 -> idpyr + nh4 + h2o2	trp + h2o + o2 -> idpyr + nh4 + h2o2
IDPD	Tryptophan metabolism	indolepyruvate decarboxylase	4.1.1.74	H16_B1399	ipdC	idpyr + h -> i3aa + co2	idpyr + h -> i3aa + co2	idpyr + h -> i3aa + co2
ALHD8	Tryptophan metabolism	aldehyde dehydrogenase (NAD+)	1.2.1.3	H16_A0232/H16_A0745/H16_A1114/H16_A1495/H16_B0212/H16_B0421/H16_B0737/H16_B0833/H16_B1534/H16_B1735/H16_B1751/H16_B1835/H16_B1960/H16_B2444	unknown/unknown/unknown/unknown/unknown/unknown/unknown/unknown/unknown/unknown/unknown/unknown/exaC/unknown/	i3aa + nad + h2o -> i3ac + nadh + 2 h	i3aa + nad + h2o -> i3ac + nadh + 2 h	i3aa + nad + h2o -> i3ac + nadh + 2 h
MNAO11	Tryptophan metabolism	monoamine oxidase	1.4.3.4	H16_A0831	maoB	sertn + h2o + o2 -> 5hiaa + nh4 + h2o2	sertn + h2o + o2 -> 5hiaa + nh4 + h2o2	sertn + h2o + o2 -> 5hiaa + nh4 + h2o2
MNAO12	Tryptophan metabolism	monoamine oxidase	1.4.3.4	H16_A0831	maoB	tyrpm + h2o + o2 -> i3aa + nh4 + h2o2	tyrpm + h2o + o2 -> i3aa + nh4 + h2o2	tyrpm + h2o + o2 -> i3aa + nh4 + h2o2
ALHD9	Tryptophan metabolism	aldehyde dehydrogenase (NAD+)	1.2.1.3	H16_A0232/H16_A0745/H16_A1114/H16_A1495/H16_B0212/H16_B0421/H16_B0737/H16_B0833/H16_B1534/H16_B1735/H16_B1751/H16_B1835/H16_B1960/H16_B2444	unknown/unknown/unknown/unknown/unknown/unknown/unknown/unknown/unknown/unknown/unknown/unknown/exaC/unknown/	5hiaa + nad + h2o -> 5hiac + nadh + 2 h	5hiaa + nad + h2o -> 5hiac + nadh + 2 h	5hiaa + nad + h2o -> 5hiac + nadh + 2 h
FBMO5	Tryptophan metabolism	flavin-binding monooxygenase	1.14.13.-	H16_A1145/H16_B0495/H16_B1480/H16_B2135	unknown/unknown/unknown/unknown	idlac + o2 + nadph + 2 h -> 6hidlac + nadp + h2o	idlac + o2 + nadph + h -> 6hidlac + nadp + h2o	idlac + o2 + nadph + h -> 6hidlac + nadp + h2o
TRPD	Tryptophan metabolism	tryptophan 2,3-dioxygenase	1.13.11.11	H16_A2816/H16_B1418	tdo1/tdo2	trp + o2 -> forkn	trp + o2 -> forkn	trp + o2 -> forkn
KYNRN1	Tryptophan metabolism	kynureninase	3.7.1.3	H16_A2815	kynU	forkn + h2o -> forant + ala + h	forkn + h2o -> forant + ala + h	forkn + h2o -> forant + ala + h
ACFM3	Tryptophan metabolism	arylformamidase	3.5.1.9	H16_A3005/H16_B1997	unknown/unknown	forant + h2o -> formate + an + h	forant + h2o -> formate + an + h	forant + h2o -> formate + an + h
ACFM4	Tryptophan metabolism	arylformamidase	3.5.1.9	H16_A3005/H16_B1997	unknown/unknown	forkn + h2o -> formate + kn + h	forkn + h2o -> formate + kn + h	forkn + h2o -> formate + kn + h
KYNRN2	Tryptophan metabolism	kynureninase	3.7.1.3	H16_A2815	kynU	kn + h2o -> an + ala + h	kn + h2o -> an + ala + h	kn + h2o -> an + ala + h
AMDS2	Tryptophan metabolism	amidase	3.5.1.4	H16_A1469/H16_B1874/H16_B2459	unknown/unknown/aimE	id3act + h2o -> i3ac + nh4	id3act + h2o -> i3ac + nh4	id3act + h2o -> i3ac + nh4
NITL1	Tryptophan metabolism	nitrilase	3.5.5.1	H16_A1125	nit	idactn + 2 h2o -> i3ac + nh4	idactn + 2 h2o -> i3ac + nh4	idactn + 2 h2o -> i3ac + nh4
CATL	Tryptophan metabolism	catalase	1.11.1.6	H16_A2777/H16_A3109/H16_B1428	katG/katE1/katE2	2 3han + 2 o2 -> cvn + 2 h2o2 + h2	2 3han + 2 o2 -> cvn + 2 h2o2 + h2	2 3han + 2 o2 -> cvn + 2 h2o2 + h2
KYNRN3	Tryptophan metabolism	kynureninase	3.7.1.3	H16_A2815	kynU	hlk + h2o -> 3han + ala + h	hlk + h2o -> 3han + ala + h	hlk + h2o -> 3han + ala + h
PAAD6	Tryptophan metabolism	phenylacrylic acid decarboxylase	4.1.1.-	H16_B2447	unknown	hlk + h -> hkn + co2	hlk + h -> hkn + co2	hlk + h -> hkn + co2
MNAO14	Tryptophan metabolism	monoamine oxidase	1.4.3.4	H16_A0831	maoB	hkn + o2 -> 48dhq + nh4 + h2o2	hkn + o2 -> 48dhq + nh4 + h2o2	hkn + o2 -> 48dhq + nh4 + h2o2
ACMSD	Tryptophan metabolism	aminocarboxymuconate-semialdehyde decarboxylase	4.1.1.45	H16_B0330	acmD	2a3cms + h -> 2amcs + co2	2a3cms + 2 h -> 2amcs + co2	2a3cms + h -> 2amcs + co2
HMSALD1	Tryptophan metabolism	2-hydroxymuconic semialdehyde dehydrogenase	1.2.1.32	H16_B0547	unknown	2amcs + nad + h2o -> 2amc + nadh + 2 h	2amcs + nad + h2o -> 2amc + nadh + 2 h	2amcs + nad + h2o -> 2amc + nadh + h
OGDH3	Tryptophan metabolism	2-oxoglutarate dehydrogenase E1 component	1.2.4.2	H16_A2325	odhA	2oad + coa + nad -> glutcoa + co2 + nadh	2oad + nad + coa -> glutcoa + co2 + nadh	2oad + coa + nad -> glutcoa + co2 + nadh
BKAR2	Tryptophan metabolism	beta-ketoacyl-ACP reductase	1.3.1.-	H16_B0731/H16_B0734	unknown/unknown	5co46d + nadph + h -> 5co46dp + nadp	5co46d + nadph + h -> 5co46dp + nadp	5co46d + nadph + h -> 5co46dp + nadp
KNTAPB	Tryptophan metabolism					knt + h2o <-> amphebut	knt + h2o <-> amphebut	knt + h2o <-> amphebut

KNAKGT	Tryptophan metabolism	kynurenine-oxoglutarate transaminase	2.6.1.7			amphebut + glu -> kn + akg	amphebut + glu -> kn + akg	amphebut + glu -> kn + akg
4HPHEA1	Tyrosine metabolism	4-hydroxyphenylacetate-3-hydroxylase	1.14.13.3	H16_B0496	unknown	hpheac + o2 + nadh + h -> 34dhpheac + nad + h2o	hpheac + o2 + nadh + h -> 34dhpheac + nad + h2o	hpheac + o2 + nadh + h -> 34dhpheac + nad + h2o
4HPHEA2	Tyrosine metabolism	4-hydroxyphenylacetate-3-hydroxylase	1.14.13.3	H16_B0496	unknown	4hpheac + o2 + nadh + h -> 34dhpheac + nad + h2o	4hpheac + o2 + nadh + h -> 34dhpheac + nad + h2o	4hpheac + o2 + nadh + h -> 34dhpheac + nad + h2o
MNAO3	Tyrosine metabolism	monoamine oxidase	1.4.3.4	H16_A0831	maoB	h2o + o2 + tym -> 4hac + h2o2 + nh4	h2o + o2 + tym -> 4hac + h2o2 + nh4	h2o + o2 + tym -> 4hac + h2o2 + nh4
FBMO2	Tyrosine metabolism	flavin-binding monooxygenase	1.14.13.-	H16_A1145/H16_B0495/H16_B1480/H16_B2135	unknown/unknown/unknown/unknown	tym + o2 + nadh + h -> dopa + nad + h2o	tym + o2 + nadh + h -> dopa + nad + h2o	tym + o2 + nadh + h -> dopa + nad + h2o
FBMO3	Tyrosine metabolism	flavin-binding monooxygenase	1.14.13.-	H16_A1145/H16_B0495/H16_B1480/H16_B2135	unknown/unknown/unknown/unknown	z4hphea + nadph + 2 h + o2 -> 4hmdn + nadp + 2 h2o	z4hphea + nadph + h + o2 -> 4hmdn + nadp + 2 h2o	z4hphea + nadph + h + o2 -> 4hmdn + nadp + 2 h2o
CARHM1	Tyrosine metabolism	5-carboxymethyl-2-hydroxymuconate isomerase	5.3.3.10	H16_A0624/H16_B1250	hpaF/unknown	5cm2hm -> 5c2o3e	5cm2hm -> 5c2o3e	5cm2hm -> 5c2o3e
ACTF2	Tyrosine metabolism	acetyltransferase	2.3.1.-	H16_A0039/H16_A0240/H16_A0269/H16_A0699/H16_A1315/H16_A1564/H16_A1683/H16_A1802/H16_A2759/H16_A3071/H16_A3093/H16_A3221/H16_A3529/H16_A3586/H16_B0018/H16_B0021/H16_B0032/H16_B0219/H16_B1278/H16_B1292/H16_B1407/H16_B1663/H16_B1899/H16_B2397/	unknown/unknown/unknown/unknown/unknown/unknown/unknown/unknown/unknown/unknown/unknown/unknown/unknown/pat/unknown/unknown/wbpD/unknown/unknown/phnT/unknown/unknown/unknown/unknown/	4hpheacoa + gly -> 4hphegly + coa + h	4hpheacoa + gly -> 4hphegly + coa + h	4hpheacoa + gly -> 4hphegly + coa + h
PAAD3	Tyrosine metabolism	phenylacrylic acid decarboxylase	4.1.1.-	H16_B2447	unknown	2 cddh + 2 h + o2 -> 2 dhid + 2 co2 + 2 h2o	2 cddh + 2 h + o2 -> 2 dhid + 2 co2 + 2 h2o	2 cddh + 2 h + o2 -> 2 dhid + 2 co2 + 2 h2o
PAAD4	Tyrosine metabolism	phenylacrylic acid decarboxylase	4.1.1.-	H16_B2447	unknown	dpcr + h -> dhid + co2	dpcr + h -> dhid + co2	dpcr + h -> dhid + co2
ACDH2	Tyrosine metabolism	acyl dehydratase	4.2.1.-	H16_A1069/H16_A1070/H16_A1289/H16_A2151/H16_A3307/H16_B0359/H16_B0706	unknown/unknown/unknown/unknown/unknown/unknown/unknown/	2hhpdd + h2o -> 4h2ohep	2hhpdd + h2o -> 4h2ohep	2hhpdd + h2o -> 4h2ohep
ACDH3	Tyrosine metabolism	acyl dehydratase	4.2.1.-	H16_A1069/H16_A1070/H16_A1289/H16_A2151/H16_A3307/H16_B0359/H16_B0706	unknown/unknown/unknown/unknown/unknown/unknown/unknown/	2o3e + h2o -> 4h2ohep	2o3e + h2o -> 4h2ohep	2o3e + h2o -> 4h2ohep
DHHED1	Tyrosine metabolism	2,4-dihydroxyhept-2-ene-1,7-dioic acid aldolase	4.1.2.-	H16_A0615/H16_B0632/H16_B1223	hpaI1/unknown/unknown	4h2ohep -> sucsal + pyr	4h2ohep -> sucsal + pyr	4h2ohep -> sucsal + pyr
MALAAC	Tyrosine metabolism	maleylacetoacetate isomerase	5.2.1.2	H16_A0362	unknown	4maac -> 4faac	4maac -> 4faac	4maac -> 4faac
FUMAAC	Tyrosine metabolism	fumarylacetoacetase	3.7.1.2	H16_B0324/H16_B1670	unknown/fahA	4faac + h2o -> acac + fum + h	4faac + h2o -> acac + fum + h	4faac + h2o -> acac + fum + h
HOMOGD	Tyrosine metabolism	homogentisate 1,2-dioxygenase	1.13.11.5	H16_B1671	hmgA	homogen + o2 -> 4maac + h	homogen + o2 -> 4maac + h	homogen + o2 -> 4maac + h
4HPHED1	Tyrosine metabolism	4-hydroxyphenylpyruvate dioxygenase	1.13.11.27	H16_B1083	hpd	4hpp + o2 -> homogen + co2	4hpp + o2 -> homogen + co2	4hpp + o2 -> homogen + co2
PAAD5	Tyrosine metabolism	phenylacrylic acid decarboxylase	4.1.1.-	H16_B2447	unknown	2 homogen + 3 o2 + 2 nadph + 6 h -> 2 gtah + 2 co2 + 2 nadp + 4 h2o	2 homogen + 3 o2 + 2 nadph + 4 h -> 2 gtah + 2 co2 + 2 nadp + 4 h2o	2 homogen + 3 o2 + 2 nadph + 4 h -> 2 gtah + 2 co2 + 2 nadp + 4 h2o
LAAO4	Tyrosine metabolism	L-amino-acid oxidase	1.4.3.2	H16_A0845/H16_A0856	lao1/lao2	tyr + h2o + o2 -> 4hpp + nh4 + h2o2	tyr + h2o + o2 -> 4hpp + nh4 + h2o2	tyr + h2o + o2 -> 4hpp + nh4 + h2o2
ASPAM5	Tyrosine metabolism	aspartate aminotransferase	2.6.1.1	H16_A2857	unknown	akg + tyr <-> 4hpp + glu	akg + tyr <-> 4hpp + glu	akg + tyr <-> 4hpp + glu
MNAO15	Tyrosine metabolism	monoamine oxidase	1.4.3.4	H16_A0831	maoB	dopa + h2o + o2 -> 34dhpac + nh4 + h2o2	dopa + h2o + o2 -> 34dhpac + nh4 + h2o2	dopa + h2o + o2 -> 34dhpac + nh4 + h2o2
MNAO4	Tyrosine metabolism	monoamine oxidase	1.4.3.4	H16_A0831	maoB	motym + h2o + o2 -> 3m4hpa + h2o2 + nh4	motym + h2o + o2 -> 3m4hpa + h2o2 + nh4	motym + h2o + o2 -> 3m4hpa + h2o2 + nh4
MNAO5	Tyrosine metabolism	monoamine oxidase	1.4.3.4	H16_A0831	maoB	norad + h2o + o2 -> 34dhma + nh4 + h2o2	norad + h2o + o2 -> 34dhma + nh4 + h2o2	norad + h2o + o2 -> 34dhma + nh4 + h2o2
MNAO6	Tyrosine metabolism	monoamine oxidase	1.4.3.4	H16_A0831	maoB	adrn + h2o + o2 -> 34dhma + methl + h2o2	adrn + h2o + o2 -> 34dhma + methl + h2o2	adrn + h2o + o2 -> 34dhma + methl + h2o2
MNAO7	Tyrosine metabolism	monoamine oxidase	1.4.3.4	H16_A0831	maoB	normp + h2o + o2 -> 3m4hpg + nh4 + h2o2	normp + h2o + o2 -> 3m4hpg + nh4 + h2o2	normp + h2o + o2 -> 3m4hpg + nh4 + h2o2
MNAO8	Tyrosine metabolism	monoamine oxidase	1.4.3.4	H16_A0831	maoB	metp + h2o + o2 -> 3m4hpg + h2o2 + methl	metp + h2o + o2 -> 3m4hpg + h2o2 + methl	metp + h2o + o2 -> 3m4hpg + h2o2 + methl
ALCDd	Tyrosine metabolism	alcohol dehydrogenase	1.1.1.1	H16_A0757/H16_A3330/H16_B0517/H16_B1433/H16_B1699/H16_B1745/H16_B1834/H16_B2470	adh/unknown/unknown/adhP/unknown/unknown/unknown/unknown	34dhma + nadh + h <-> 34dhpeg + nad	34dhma + nadh + h <-> 34dhpeg + nad	34dhma + nadh + h <-> 34dhpeg + nad
GENDO	Tyrosine metabolism	gentisate 1,2-dioxygenase	1.13.11.4	H16_B0873	unknown	gensa + o2 -> malpyr + h	gensa + o2 -> malpyr + h	gensa + o2 -> malpyr + h
MALPYRI	Tyrosine metabolism	maleylpyruvate isomerase	5.2.1.4	H16_B0875	unknown	malpyr -> fumpyr	malpyr -> fumpyr	malpyr -> fumpyr
ACPYRH	Tyrosine metabolism	acylpyruvate hydrolase	3.7.1.5	H16_B0428/H16_B0874	unknown/unknown	fumpyr + h2o -> fum + pyr + h	fumpyr + h2o -> fum + pyr + h	fumpyr + h2o -> fum + pyr + h
DHHED2	Tyrosine metabolism	2,4-dihydroxyhept-2-ene-1,7-dioic acid aldolase	4.1.2.-	H16_A0615/H16_B0632/H16_B1223	hpaI1/unknown/unknown	dhhedd -> sucsal + pyr	dhhedd -> sucsal + pyr	dhhedd -> sucsal + pyr
NADHDH	Ubiquinone	NADH dehydrogenase	1.6.5.3	H16_A0251/H16_A1050/H16_A1051/H16_A1052/H16_A1053/H16_A1054/H16_A1055/H16_A1056/H16_A1057/H16_A1058/H16_A1059/H16_A1060/H16_A1061/H16_A1062/H16_A1063/	unknown/nuoA/nuoB/nuoC/nuoD/nuoE/nuoF/nuoG/nuoH/nuoI/nuoJ/nuoK/nuoL/nuoM/nuoN/	uq + 2 h -> uqh2	uq + 2 h -> uqh2	uq + 2 h -> uqh2
NAPTS	Ubiquinone Biosynthesis	naphthoate synthase	4.1.3.36	H16_B1695	menB	sbzcoa -> coa + dhn	sbzcoa -> coa + dhn	sbzcoa -> coa + dhn
CHMPL	Ubiquinone Biosynthesis	Chorismate pyruvate lyase	4.1.3.40	H16_A3165	unknown	chor -> 4hb + pyr	chor -> 4hb + pyr	chor -> 4hb + pyr
DMUQMT	Ubiquinone Biosynthesis	3-Dimethylubiquinonol 3-methyltransferase	2.1.1.64	H16_A0787/H16_A1649	ubiG/unknown	2omhmbl + sam -> sah + h + uqh2	2omhmbl + sam -> sah + uq	2omhmbl + sam -> sah + h + uqh2
HBZOCPT	Ubiquinone Biosynthesis	Hydroxybenzoate octaprenyltransferase	2.5.1.-	H16_A3107	unknown	4hb + opp -> 3op4hb + ppi	4hb + opp -> 3op4hb + ppi + h	4hb + opp -> 3op4hb + ppi + h
OCPHPM	Ubiquinone Biosynthesis	2-octaprenyl-6-hydroxyphenol methylase	2.1.1.64	H16_A0787/H16_A1649	ubiG/unknown	2op6hp + sam -> 2opmp + sah + h	2op6hp + sam -> 2opmp + sah + h	2op6hp + sam -> 2opmp + sah + h
OCTMBZM	Ubiquinone Biosynthesis	2-Octaprenyl-6-methoxy-benzoquinol methylase	2.1.1.-	H16_A0445	ubiE1	2ombzl + sam -> 2ommbl + sah + h	2ombzl + sam -> 2ommbl + sah + h	2ombzl + sam -> 2ommbl + sah + h
OMMBZH	Ubiquinone Biosynthesis	2-octaprenyl-3-methyl-6-methoxy-1,4-benzoquinol hydroxylase	1.14.13.-			2ommbl + o2 + nadph + 2 h -> 2omhmbl + nadp + h2o	2ommbl + o2 + nadph + h -> 2omhmbl + nadp + h2o	2ommbl + o2 + nadph + h -> 2omhmbl + nadp + h2o
OCMPH1	Ubiquinone Biosynthesis	2-octaprenyl-6-methoxyphenol hydroxylase	1.14.13.-	H16_A0504	ubiH	2opmp + o2 + nadph + 2 h -> 2ombzl + nadp + h2o	2opmp + o2 + nadph + h -> 2ombzl + nadp + h2o	2opmp + o2 + nadph + h -> 2ombzl + nadp + h2o
OCMPH2	Ubiquinone Biosynthesis	2-octaprenyl-6-methoxyphenol hydroxylase	1.14.13.-	H16_A0504	ubiH	2 2opmp + o2 -> 2 2ombzl	2 2opmp + o2 -> 2 2ombzl	2 2opmp + o2 -> 2 2ombzl
OCMPH3	Ubiquinone Biosynthesis	2-octaprenyl-6-methoxyphenol hydroxylase (anaerobic)	1.14.13.-	H16_A0504	ubiH	2opmp + 2 atp + 3 h2o + nad -> 2ombzl + 2 adp + h + nadh + 2 pi	2opmp + 2 atp + 3 h2o + nad -> 2ombzl + 2 adp + 3 h + nadh + 2 pi	2opmp + 2 atp + 3 h2o + nad -> 2ombzl + 2 adp + 3 h + nadh + 2 pi

OCHBZDC	Ubiquinone Biosynthesis	Octaprenyl-hydroxybenzoate decarboxylase	4.1.1.-	H16_A2859/H16_A3344/ H16_A3366/H16_A3372	ubiD/ubiX1/ubiD2/ubiX2	3op4hb + h -> 2opp + co2	3op4hb + h -> 2opp + co2	3op4hb + h -> 2opp + co2
OCPPH2	Ubiquinone Biosynthesis	2-Octaprenylphenol hydroxylase	2.7.-.-	H16_A0448	ubiB	2opp + o2 + nadph + 2 h -> 2op6hp + nadp + h2o	2opp + o2 + nadph + h -> 2op6hp + nadp + h2o	2opp + o2 + nadph + h -> 2op6hp + nadp + h2o
OCPPH1	Ubiquinone Biosynthesis	2-Octaprenylphenol hydroxylase	0	H16_A0448	ubiB	2 2opp + o2 -> 2 2op6hp	2 2opp + o2 -> 2 2op6hp	2 2opp + o2 -> 2 2op6hp
OCPPH3	Ubiquinone Biosynthesis	2-Octaprenylphenol hydroxylase (anaerobic)	0	H16_A0448	ubiB	2opp + 2 atp + 3 h2o + nad -> 2op6hp + 2 adp + h + nadh + 2 pi	2opp + 2 atp + 3 h2o + nad -> 2op6hp + 2 adp + 3 h + nadh + 2 pi	2opp + 2 atp + 3 h2o + nad -> 2op6hp + 2 adp + 3 h + nadh + 2 pi
UMBM1	Ubiquinone Biosynthesis	ubiquinone/menaquinone biosynthesis methyltransferase	2.1.1.-	H16_A0445	ubiE1	2dmmq8 + sam -> mk + sah	2dmmq8 + sam -> mk + sah	2dmmq8 + sam -> mk + sah
UMBM2	Ubiquinone Biosynthesis	ubiquinone/menaquinone biosynthesis methyltransferase	2.1.1.-	H16_A0445	ubiE1	pnpq + sam -> pq + sah	pnpq + sam -> pq + sah	pnpq + sam -> pq + sah
UBQBM	Ubiquinone Biosynthesis	ubiquinone biosynthesis monooxygenase Coq7	1.14.13.-	H16_A3283	unknown	hpmmbq + o2 + nadph + h -> hpmhmbq + nadp + h2o	hpmmbq + o2 + nadph -> hpmhmbq + nadp + h2o	hpmmbq + o2 + nadph -> hpmhmbq + nadp + h2o
SPMS3	Ubiquinone Biosynthesis	spermidine synthase	2.5.1.16	H16_A1603/H16_A2204/ H16_A2643/H16_A2647/	unknown/unknown/ unknown/speE/	sama + sprmd -> 5mta + sprm + h	sama + sprmd -> 5mta + sprm + h	sama + sprmd -> 5mta + sprm + h
ALHD12	Ubiquinone Biosynthesis	aldehyde dehydrogenase (NAD+)	1.2.1.3	H16_A0232/H16_A0745/ H16_A1114/H16_A1495/ H16_B0212/H16_B0421/ H16_B0737/H16_B0833/ H16_B1534/H16_B1735/ H16_B1751/H16_B1835/ H16_B1960/H16_B2444	unknown/unknown/ unknown/unknown/ unknown/unknown/ unknown/unknown/ unknown/unknown/ unknown/unknown/ exaC/unknown/	bapa + nad + h2o -> bala + nadh + 2 h	bapa + nad + h2o -> bala + nadh + 2 h	bapa + nad + h2o -> bala + nadh + 2 h
OMMBZHx	Ubiquinone Biosynthesis	2-octaprenyl-6-methoxyphenol hydroxylase	1.14.13.-			2ommbl + 2 atp + 3 h2o + nad -> 2omhmbl + 2 adp + h + nadh + 2 pi	2ommbl + 2 atp + 3 h2o + nad -> 2omhmbl + 2 adp + 3 h + nadh + 2 pi	2ommbl + 2 atp + 3 h2o + nad -> 2omhmbl + 2 adp + 3 h + nadh + 2 pi
CAT	Unassigned	catalase	1.11.1.6			2 h2o2 -> 2 h2o + o2	2 h2o2 -> 2 h2o + o2	2 h2o2 -> 2 h2o + o2
MISRXN	unclear reaction	unclear reaction				g3p + pyr -> 4mhetz	g3p + pyr -> 4mhetz	g3p + pyr -> 4mhetz
ACGPR	Urea Cycle and Metabolism of amino groups	N-acetyl-g-glutamyl-phosphate reductase	1.2.1.38	H16_A0220/H16_B0337	argC1/argC2	naglus + nadp + pi <-> acg5p + 3 h + nadph	naglus + nadp + pi <-> acg5p + h + nadph	naglus + nadp + pi <-> acg5p + h + nadph
GLU5SD	Urea Cycle and Metabolism of amino groups	glutamate-5-semialdehyde dehydrogenase	1.2.1.41	H16_A3136	proA	glu5p + 3 h + nadph -> glugsal + nadp + pi	glu5p + h + nadph -> glugsal + nadp + pi	glu5p + h + nadph -> glugsal + nadp + pi
AGLUS	Urea Cycle and Metabolism of amino groups	N-acetylglutamate synthase	2.3.1.1	H16_A2343/H16_A3263	argAB/argJ	accoa + glu -> naglu + coa + h	accoa + glu -> naglu + coa + h	accoa + glu -> naglu + coa + h
SPMS1	Urea Cycle and Metabolism of amino groups	spermidine synthase	2.5.1.16	H16_A1603/H16_A2204/ H16_A2643/H16_A2647/	unknown/unknown/ unknown/speE/	sama + ptrc -> 5mta + h + sprmd	sama + ptrc -> 5mta + h + sprmd	sama + ptrc -> 5mta + h + sprmd
AORNT	Urea Cycle and Metabolism of amino groups	acetylornithine transaminase	2.6.1.11	H16_A3025	argD	naorn + akg <-> naglus + glu	naorn + akg <-> naglus + glu	naorn + akg <-> naglus + glu
GLU5K	Urea Cycle and Metabolism of amino groups	glutamate 5-kinase	2.7.2.11	H16_A3249	proB	atp + glu -> adp + glu5p	atp + glu -> adp + glu5p	atp + glu -> adp + glu5p
AGLUK	Urea Cycle and Metabolism of amino groups	acetylglutamate kinase	2.7.2.8	H16_A0208	argB	naglu + atp -> acg5p + adp	naglu + atp -> acg5p + adp	naglu + atp -> acg5p + adp
ACORND	Urea Cycle and Metabolism of amino groups	acetylornithine deacetylase	3.5.1.16	H16_A1454/H16_B0459	argE1/argE2	naorn + h2o -> ac + orn	naorn + h2o -> ac + orn	naorn + h2o -> ac + orn
AGMT	Urea Cycle and Metabolism of amino groups	agmatinase	3.5.3.11	H16_A0044	speB	agmatine + h2o -> ptrc + urea	agmatine + h2o -> ptrc + urea	agmatine + h2o -> ptrc + urea
ARGDC	Urea Cycle and Metabolism of amino groups	arginine decarboxylase	4.1.1.19	H16_A2930	ldcC	arg + h -> agmatine + co2	arg + h -> agmatine + co2	arg + h -> agmatine + co2
ACORND	Urea cycle and metabolism of amino groups	acetylornithine deacetylase	3.5.1.14	H16_B0491/H16_B1209	unknown/unknown	naorn + h2o -> ac + orn	naorn + h2o -> ac + orn	naorn + h2o -> ac + orn
GLUNAC	Urea cycle and metabolism of amino groups	glutamate N-acetyltransferase	2.3.1.35	H16_A3263	argJ	naorn + glu <-> orn + naglu	naorn + glu <-> orn + naglu	naorn + glu <-> orn + naglu
ALLPH	Urea cycle and metabolism of amino groups	allophanate hydrolase	3.5.1.54	H16_A0125/H16_B1758	unknown/alpH	u1car + h2o + 3 h -> 2 co2 + 2 nh4	u1car + h2o + 3 h -> 2 co2 + 2 nh4	u1car + h2o + 3 h -> 2 co2 + 2 nh4
MNAO13	Urea cycle and metabolism of amino groups	monoamine oxidase	1.4.3.4	H16_A0831	maoB	acputs + h2o + o2 -> n4aab + nh4 + h2o2	acputs + h2o + o2 -> n4aab + nh4 + h2o2	acputs + h2o + o2 -> n4aab + nh4 + h2o2
ALHD10	Urea cycle and metabolism of amino groups	aldehyde dehydrogenase (NAD+)	1.2.1.3	H16_A0232/H16_A0745/ H16_A1114/H16_A1495/ H16_B0212/H16_B0421/ H16_B0737/H16_B0833/ H16_B1534/H16_B1735/ H16_B1751/H16_B1835/ H16_B1960/H16_B2444	unknown/unknown/ unknown/unknown/ unknown/unknown/ unknown/unknown/ unknown/unknown/ unknown/unknown/ exaC/unknown/	n4aab + nad + h2o -> 4aabut + nadh + 2 h	n4aab + nad + h2o -> 4aabut + nadh + 2 h	n4aab + nad + h2o -> 4aabut + nadh + 2 h
ALHD11	Urea cycle and metabolism of amino groups	aldehyde dehydrogenase (NAD+)	1.2.1.3	H16_A0232/H16_A0745/ H16_A1114/H16_A1495/ H16_B0212/H16_B0421/ H16_B0737/H16_B0833/ H16_B1534/H16_B1735/ H16_B1751/H16_B1835/ H16_B1960/H16_B2444	unknown/unknown/ unknown/unknown/ unknown/unknown/ unknown/unknown/ unknown/unknown/ unknown/unknown/ exaC/unknown/	4ab + nadp + h2o -> gaba + nadph + 3 h	4ab + nadp + h2o -> gaba + nadph + 2 h	4ab + nadp + h2o -> gaba + nadph + 2 h
SPMS2	Urea cycle and metabolism of amino groups	spermidine synthase	2.5.1.16	H16_A1603/H16_A2204/ H16_A2643/H16_A2647/	unknown/unknown/ unknown/speE/	sama + ptrc -> 5mta + sprmd + h	sama + ptrc -> 5mta + sprmd + h	sama + ptrc -> 5mta + sprmd + h
AMDS3	Urea cycle and metabolism of amino groups	amidase	3.5.1.4	H16_A1469/H16_B1874/ H16_B2459	unknown/unknown/ aimE	guadbut + h2o -> guadbutn + nh4	guadbut + h2o -> guadbutn + nh4	guadbut + h2o -> guadbutn + nh4
LEUD3	Valine, Leucine and Isoleucine Biosynthesis	leucine dehydrogenase	1.4.1.9	H16_B0449	unknown	3mop + nh4 + nadh + h -> ile + nad + h2o	3mop + nh4 + nadh + h -> ile + nad + h2o	3mop + nh4 + nadh + h -> ile + nad + h2o
LEUD4	Valine, Leucine and Isoleucine Biosynthesis	leucine dehydrogenase	1.4.1.9	H16_B0449	unknown	3mob + nh4 + nadh + h -> val + h2o + nad	3mob + nh4 + nadh + h -> val + h2o + nad	3mob + nh4 + nadh + h -> val + h2o + nad
3IPPMD1	Valine, Leucine and Isoleucine Biosynthesis	3-isopropylmalate/(R)-2-methylmalate dehydratase	4.2.1.33	(H16_A1236/H16_A1549 /H16_A2621/H16_B0052 /H16_B2275)&(H16_A12 37/H16_A1550/H16_A26 20/H16_B0051/H16_B22 76)	(leuC1/leuC2/leuC3/l euC4/leu5)&(leuD1/l euD2/leuD3/leuD4/l euD5)	r2mm -> 2mm + h2o	r2mm -> 2mm + h2o	r2mm -> 2mm + h2o
3IPPMD2	Valine, Leucine and Isoleucine Biosynthesis	3-isopropylmalate/(R)-2-methylmalate dehydratase	4.2.1.33	(H16_A1236/H16_A1549 /H16_A2621/H16_B0052 /H16_B2275)&(H16_A12 37/H16_A1550/H16_A26 20/H16_B0051/H16_B22 76)	(leuC1/leuC2/leuC3/l euC4/leu5)&(leuD1/l euD2/leuD3/leuD4/l euD5)	2mm + h2o -> e3mm	2mm + h2o -> e3mm	2mm + h2o -> e3mm
IPPMD	Valine, Leucine and Isoleucine Biosynthesis	3-isopropylmalate dehydrogenase	1.1.1.85	H16_A2133/H16_A2619	leuB2/leuB3	e3mm + nad -> obut + co2 + nadh	e3mm + nad -> obut + co2 + nadh	e3mm + nad -> obut + co2 + nadh
LEUD1	Valine, Leucine and Isoleucine degradation	leucine dehydrogenase	1.4.1.9	H16_B0449	unknown	3rbleu + nad + h2o -> 3o4mp + nh4 + nadh + h	3rbleu + nad + h2o -> 3o4mp + nh4 + nadh + h	3rbleu + nad + h2o -> 3o4mp + nh4 + nadh + h

LEUD2	Valine, Leucine and Isoleucine degradation	leucine dehydrogenase	1.4.1.9	H16_B0449	unknown	leu + h2o + nad <-> 4mop + nh4 + nadh + h	leu + h2o + nad <-> 4mop + nh4 + nadh + h	leu + h2o + nad <-> 4mop + nh4 + nadh + h
LAAO3	Valine, Leucine and Isoleucine degradation	L-amino-acid oxidase	1.4.3.2	H16_A0845/H16_A0856	lao1/lao2	ile + h2o + o2 <-> 3mop + nh4 + h2o2	ile + h2o + o2 <-> 3mop + nh4 + h2o2	ile + h2o + o2 <-> 3mop + nh4 + h2o2
DHLLM1	Valine, Leucine and Isoleucine degradation	dihydrolipoyllysine-residue (2-methylpropanoyl)transferase	2.3.1.168	H16_B2235	bkdB	coa + s3mbdlipo -> 3mbcoa + dlipo	coa + s3mbdlipo -> 3mbcoa + dlipo	coa + s3mbdlipo -> 3mbcoa + dlipo
DHLLM2	Valine, Leucine and Isoleucine degradation	dihydrolipoyllysine-residue (2-methylpropanoyl)transferase	2.3.1.168	H16_B2235	bkdB	coa + s2mpdlipo -> 2mppacoa + dlipo	coa + s2mpdlipo -> 2mppacoa + dlipo	coa + s2mpdlipo -> 2mppacoa + dlipo
DHLLM3	Valine, Leucine and Isoleucine degradation	dihydrolipoyllysine-residue (2-methylpropanoyl)transferase	2.3.1.168	H16_B2235	bkdB	coa + s2mbdlipo -> 2mbcoa + dlipo	coa + s2mbdlipo -> 2mbcoa + dlipo	coa + s2mbdlipo -> 2mbcoa + dlipo
ISOVC	Valine, Leucine and Isoleucine degradation	isovaleryl-CoA dehydrogenase	1.3.99.10	H16_A0167/H16_A1291/H16_A1972	ivd1/unknown/ivd2	3mbcoa + fad -> 3mccoa + fadh2	3mbcoa + fad + h -> 3mccoa + fadh2	3mbcoa + fad + h -> 3mccoa + fadh2
ACOADH3	Valine, Leucine and Isoleucine degradation	acyl-CoA dehydrogenase	1.3.99.3	H16_A0101/H16_A0460/H16_A0816/H16_A0843/H16_A0863/H16_A1067/H16_A1068/H16_A1530/H16_A2458/H16_A2596/H16_B0014/H16_B0087/H16_B0356/H16_B0360/H16_B0379/H16_B0383/H16_B0384/H16_B0395/H16_B0396/H16_B0400/H16_B0580/H16_B0660/H16_B0661/H16_B0664/H16_B0665/H16_B0676/H16_B0683/H16_B0703/H16_B0704/H16_B0721/H16_B0722/H16_B0751/H16_B0849/H16_B0909/H16_B0913/H16_B0938/H16_B0975/H16_B1192/H16_B1332/H16_B1367/H16_B1481/H16_B1694/H16_B1826/H16_B2157/H16_B2158/	unknown/acaD/unknown/unknown/unknown/unknown/unknown/abmD/unknown/	3mbcoa + fad <-> 3mccoa + fadh2	3mbcoa + fad + h <-> 3mccoa + fadh2	3mbcoa + fad + h <-> 3mccoa + fadh2
ACOADH4	Valine, Leucine and Isoleucine degradation	acyl-CoA dehydrogenase	1.3.99.3	H16_A0101/H16_A0460/H16_A0816/H16_A0843/H16_A0863/H16_A1067/H16_A1068/H16_A1530/H16_A2458/H16_A2596/H16_B0014/H16_B0087/H16_B0356/H16_B0360/H16_B0379/H16_B0383/H16_B0384/H16_B0395/H16_B0396/H16_B0400/H16_B0580/H16_B0660/H16_B0661/H16_B0664/H16_B0665/H16_B0676/H16_B0683/H16_B0703/H16_B0704/H16_B0721/H16_B0722/H16_B0751/H16_B0849/H16_B0909/H16_B0913/H16_B0938/H16_B0975/H16_B1192/H16_B1332/H16_B1367/H16_B1481/H16_B1694/H16_B1826/H16_B2157/H16_B2158/	unknown/acaD/unknown/unknown/unknown/unknown/unknown/abmD/unknown/	2mppacoa + fad <-> 2mp2ecoa + fadh2	2mppacoa + fad + h <-> 2mp2ecoa + fadh2	2mppacoa + fad + h <-> 2mp2ecoa + fadh2
ACOADH5	Valine, Leucine and Isoleucine degradation	acyl-CoA dehydrogenase	1.3.99.3	H16_A0101/H16_A0460/H16_A0816/H16_A0843/H16_A0863/H16_A1067/H16_A1068/H16_A1530/H16_A2458/H16_A2596/H16_B0014/H16_B0087/H16_B0356/H16_B0360/H16_B0379/H16_B0383/H16_B0384/H16_B0395/H16_B0396/H16_B0400/H16_B0580/H16_B0660/H16_B0661/H16_B0664/H16_B0665/H16_B0676/H16_B0683/H16_B0703/H16_B0704/H16_B0721/H16_B0722/H16_B0751/H16_B0849/H16_B0909/H16_B0913/H16_B0938/H16_B0975/H16_B1192/H16_B1332/H16_B1367/H16_B1481/H16_B1694/H16_B1826/H16_B2157/H16_B2158/	unknown/acaD/unknown/unknown/unknown/unknown/unknown/abmD/unknown/	2mbcoa + fad <-> 2m2ecoa + fadh2	2mbcoa + fad + h <-> 2m2ecoa + fadh2	2mbcoa + fad + h <-> 2m2ecoa + fadh2
BTCOAD1	Valine, Leucine and Isoleucine degradation	butyryl-CoA dehydrogenase	1.3.99.2	H16_A0172/H16_B0485/H16_B0752/H16_B0850/H16_B1371	unknown/unknown/unknown/unknown/unknown/unknown	2 2mppacoa + o2 -> 2 2mp2ecoa + 2 h2o	2 2mppacoa + o2 -> 2 2mp2ecoa + 2 h2o	2 2mppacoa + o2 -> 2 2mp2ecoa + 2 h2o
BTCOAD2	Valine, Leucine and Isoleucine degradation	butyryl-CoA dehydrogenase	1.3.99.2	H16_A0172/H16_B0485/H16_B0752/H16_B0850/H16_B1371	unknown/unknown/unknown/unknown/unknown/unknown	2 2mbcoa + o2 -> 2 2m2ecoa + 2 h2o	2 2mbcoa + o2 -> 2 2m2ecoa + 2 h2o	2 2mbcoa + o2 -> 2 2m2ecoa + 2 h2o
HIBD	Valine, Leucine and Isoleucine degradation	3-hydroxyisobutyrate dehydrogenase	1.1.1.31	H16_A1239/H16_A1562/H16_B1190/H16_B1657/H16_B1750	unknown/unknown/unknown/unknown/unknown	hibut + nad <-> mmsa + nadh + h	hibut + nad <-> mmsa + nadh + h	hibut + nad <-> mmsa + nadh + h
ACCOAA1	Valine, Leucine and Isoleucine degradation	acetyl-CoA acyltransferase	2.3.1.16	H16_A0462/H16_A1290/H16_B0200	unknown/unknown/pcaF	coa + 2maaccoa -> ppcoa + accoa	coa + 2maaccoa -> ppcoa + accoa	coa + 2maaccoa -> ppcoa + accoa
BAPT2	Valine, Leucine and Isoleucine degradation	beta-alanine--pyruvate transaminase	2.6.1.18	H16_A0272	aptA	ala + mmsa <-> pyr + l3aibn	ala + mmsa <-> pyr + l3aibn	ala + mmsa <-> pyr + l3aibn
ALHD6	Valine, Leucine and Isoleucine degradation	aldehyde dehydrogenase (NAD+)	1.2.1.3	H16_A0232/H16_A0745/H16_A1114/H16_A1495/H16_B0212/H16_B0421/H16_B0737/H16_B0833/H16_B1534/H16_B1735/H16_B1751/H16_B1835/H16_B1960/H16_B2444	unknown/unknown/unknown/unknown/unknown/unknown/unknown/unknown/unknown/unknown/unknown/unknown/exaC/unknown/	mmsa + nad + h2o -> mm + nadh + h	mmsa + nad + h2o -> mm + nadh + 2 h	mmsa + nad + h2o -> mm + nadh + 2 h
MMCS	Valine, Leucine and Isoleucine degradation	unclear reaction	unclear reaction	unclear reaction	unclear reaction	mm + coa -> mmcoa-R + h2o	mm + coa + h -> mmcoa-R + h2o	mm + coa + h -> mmcoa-R + h2o
MMCE	Valine, Leucine, and Isoleucine Degradation	methylmalonyl-CoA epimerase	5.1.99.1	H16_A3551	unknown	mmcoa-R <-> mmcoa-S	mmcoa-R <-> mmcoa-S	mmcoa-R <-> mmcoa-S
MMCM	Valine, Leucine, and Isoleucine Degradation	Methylmalonyl-CoA mutase	5.4.99.2	(H16_A0280/H16_A1949)&H16_B1841&H16_B1842	(sbm1/sbm2)&unknown&unknown	succoa -> mmcoa-R	succoa -> mmcoa-R	succoa -> mmcoa-R
IPMALD	Valine, Leucine, and Isoleucine Metabolism	3-isopropylmalate dehydrogenase	1.1.1.85	H16_A0477/H16_A2133/H16_A2619/	leuB1/leuB2/leuB3/	3c2hmp + nad -> oicap + h + nadh	3c2hmp + nad -> oicap + h + nadh	3c2hmp + nad -> oicap + h + nadh

KARIS1	Valine, Leucine, and Isoleucine Metabolism	ketol-acid reductoisomerase	1.1.1.86	H16_A1037	ilvC	dhmva + nadp <-> alac-S + 2 h + nadph	dhmva + nadp <-> alac-S + h + nadph	dhmva + nadp <-> alac-S + h + nadph
KARIS2	Valine, Leucine, and Isoleucine Metabolism	ketol-acid reductoisomerase	1.1.1.86	H16_A1037	ilvC	abut + 2 h + nadph <-> dhmp + nadp	abut + h + nadph <-> dhmp + nadp	abut + h + nadph <-> dhmp + nadp
ILETA	Valine, Leucine, and Isoleucine Metabolism	isoleucine transaminase	2.6.1.42	H16_A0561	unknown	akg + ile <-> 3mop + glu	akg + ile <-> 3mop + glu	akg + ile <-> 3mop + glu
LEUTA	Valine, Leucine, and Isoleucine Metabolism	leucine transaminase	2.6.1.42	H16_A0561	unknown	4mop + glu -> akg + leu	4mop + glu -> akg + leu	4mop + glu -> akg + leu
VALTA	Valine, Leucine, and Isoleucine Metabolism	valine transaminase	2.6.1.42	H16_A0561	unknown	akg + val <-> 3mob + glu	akg + val <-> 3mob + glu	akg + val <-> 3mob + glu
IPPMS	Valine, Leucine, and Isoleucine Metabolism	2-isopropylmalate synthase	2.3.3.13	H16_A1041/H16_B0081	leuA1/leuA2	3mob + accoa + h2o -> 3c3hmp + coa + h	3mob + accoa + h2o -> 3c3hmp + coa + h	3mob + accoa + h2o -> 3c3hmp + coa + h
ACLACS	Valine, Leucine, and Isoleucine Metabolism	acetolactate synthase	2.2.1.6	H16_A1035/(H16_A1036&H16_A2231&H16_B0313&H16_B0735&H16_B2452)	ilvB/(ilvH&unknown&unknown&unknown&unknown)	h + 2 pyr -> alac-S + co2	h + 2 pyr -> alac-S + co2	h + 2 pyr -> alac-S + co2
IPMALD1	Valine, Leucine, and Isoleucine Metabolism	3-isopropylmalate dehydratase	4.2.1.33	(H16_A1236&H16_A1237)/(H16_A1549&H16_A1550)/(H16_A2620&H16_A2621)/(H16_B0051&H16_B0052)/(H16_B2275&H16_B2276)	(leuC1&leuD1)/(leuC2&leuD2)/(leuD3&leuC3)/(leuD4&leuC4)/(leuC5&leuD5)	3c2hmp <-> 2ippm + h2o	3c2hmp <-> 2ippm + h2o	3c2hmp <-> 2ippm + h2o
IPMALD2	Valine, Leucine, and Isoleucine Metabolism	3-isopropylmalate dehydratase	4.2.1.33	(H16_A1236&H16_A1237)/(H16_A1549&H16_A1550)/(H16_A2620&H16_A2621)/(H16_B0051&H16_B0052)/(H16_B2275&H16_B2276)	(leuC1&leuD1)/(leuC2&leuD2)/(leuD3&leuC3)/(leuD4&leuC4)/(leuC5&leuD5)	2ippm + h2o <-> 3c3hmp	2ippm + h2o <-> 3c3hmp	2ippm + h2o <-> 3c3hmp
DHADT1	Valine, Leucine, and Isoleucine Metabolism	dihydroxy-acid dehydratase	4.2.1.9	H16_A2987/H16_B0280	unknown/unknown	dhmva -> 3mob + h2o	dhmva -> 3mob + h2o	dhmva -> 3mob + h2o
ACHBUTS	Valine, Leucine, and Isoleucine Metabolism	2-aceto-2-hydroxybutanoate synthase	2.2.1.6	H16_A1035/(H16_A1036&H16_A2231&H16_B0313&H16_B0735&H16_B2452)	ilvB/(ilvH&unknown&unknown&unknown&unknown)	obut + h + pyr -> abut + co2	obut + h + pyr -> abut + co2	obut + h + pyr -> abut + co2
DHADT2	Valine, Leucine, and Isoleucine Metabolism	ihydroxy-acid dehydratase	4.2.1.9	H16_A2987/H16_B0280	unknown/unknown	dhmp -> 3mop + h2o	dhmp -> 3mop + h2o	dhmp -> 3mop + h2o
OMCDC	Valine, Leucine, and Isoleucine Metabolism	2-Oxo-4-methyl-3-carboxypentanoate decarboxylation	spontaneous	spontaneous	spontaneous	oicap + h -> 4mop + co2	oicap + h -> 4mop + co2	oicap + h -> 4mop + co2
PDX5PO	Vitamine B6 metabolism	pyridoxine 5'-phosphate oxidase	1.4.3.5	H16_A2802	pdxH	o2 + pdx5p -> h2o2 + pydx5p	o2 + pdx5p -> h2o2 + pydx5p	o2 + pdx5p -> h2o2 + pydx5p + h
PYAM5PO	Vitamine B6 metabolism	pyridoxamine 5'-phosphate oxidase	1.4.3.5	H16_A2802	pdxH	h2o + o2 + pyam5p -> h2o2 + nh4 + pydx5p	h2o + o2 + pyam5p -> h2o2 + nh4 + pydx5p	h2o + o2 + pyam5p -> h2o2 + nh4 + pydx5p + h
OHPBAKGT	Vitamine B6 metabolism	O-Phospho-4-hydroxy-L-threonine:2-oxoglutarate aminotransferase	2.6.1.52	H16_A0791	serC	glu + ohpb <-> akg + pht	glu + ohpb <-> akg + pht	glu + ohpb <-> akg + pht
HTHRS	Vitamine B6 metabolism	4-Hydroxy-L-threonine synthase	4.2.3.1	H16_A2265	thrC	h2o + pht -> 4hlt + pi	h2o + pht -> 4hlt + pi	h2o + pht -> 4hlt + pi
DALATA	Vitamine B6 metabolism	D-alanine transaminase	2.6.1.54			dala + pydx5p -> pyam5p + pyr	dala + pydx5p -> pyam5p + pyr	dala + pydx5p + h -> pyam5p + pyr
E4PDH	Vitamine B6 metabolism	Erythrose 4-phosphate dehydrogenase	1.2.1.72			e4p + h2o + nad <-> er4p + 2 h + nadh	e4p + h2o + nad <-> er4p + 2 h + nadh	e4p + h2o + nad <-> er4p + 2 h + nadh
P5PPR	Vitamine B6 metabolism	pyridoxal-5'-phosphate phosphohydrolase	3.1.3.74			h2o + pyam5p -> pi + pdla	h2o + pyam5p -> pi + pdla	h2o + pyam5p -> pi + pdla
PX5PS	Vitamine B6 metabolism	Pyridoxine 5'-phosphate synthase	1.1.1.262/2.6.99.2	(H16_A0513/H16_B0216/H16_B0319)/(H16_A2552)	(pdxA/pdxA/pdxA)/(pdxJ)	dx5p + nad + pht -> co2 + h + 2 h2o + nadh + pdx5p + pi	dx5p + nad + pht -> co2 + h + 2 h2o + nadh + pdx5p + pi	dx5p + nad + pht -> co2 + h + 2 h2o + nadh + pdx5p + pi
ER4PD	Vitamine B6 metabolism	Erythronate 4-phosphate (4per) dehydrogenase	1.1.1.290			er4p + nad <-> h + nadh + ohpb	er4p + nad <-> h + nadh + ohpb	er4p + nad <-> h + nadh + ohpb
PYR5OXM	Vitamine B6 metabolism	pyridoxamine 5'-phosphate oxidase	1.4.3.5	H16_A2802	pdxH	pdla + h2o + o2 <-> pl + nh4 + h2o2	pdla + h2o + o2 <-> pl + nh4 + h2o2	pdla + h2o + o2 <-> pl + nh4 + h2o2 + h
PYR5OXX	Vitamine B6 metabolism	pyridoxamine 5'-phosphate oxidase	1.4.3.5	H16_A2802	pdxH	pydxn + o2 <-> pl + h2o2	pydxn + o2 <-> pl + h2o2	pydxn + o2 <-> pl + h2o2 + h
HTHRPD	Vitamine B6 metabolism	4-hydroxythreonine-4-phosphate dehydrogenase	1.1.1.262	H16_A0513/H16_B0216/H16_B0319	pdxA1/pdxA2/pdxA3	pht + nad -> ao4pob + nadh + h	pht + nad -> ao4pob + nadh + h	pht + nad -> ao4pob + nadh + 2 h
SPOTN	Vitamine B6 metabolism	spontaneous				ao4pob + h -> 3a2op + co2	ao4pob + h -> 3a2op + co2	ao4pob + h -> 3a2op + co2
12PPDt	Transport, Extracellular	S-Propane-1,2-diol facilitated transport				12ppd-S_e <-> 12ppd-S	12ppd-S_e <-> 12ppd-S	12ppd-S_e <-> 12ppd-S
NMNt7	Transport, Extracellular	NMN transport via NMN glycohydrolase				h2o + namn_e -> h + nam + r5p	h2o + namn_e -> h + nam + r5p	h2o + namn_e -> h + nam + r5p
ACALDt	Transport, Extracellular	acetaldehyde reversible transport				acal_e <-> acal	acal_e <-> acal	acal_e <-> acal
GUAt1	Transport, Extracellular	Guanine transport				gn_e <-> gn	gn_e <-> gn	gn_e <-> gn
HYXNt	Transport, Extracellular	Hypoxanthine transport				hyxn_e <-> hyxn	hyxn_e <-> hyxn	hyxn_e <-> hyxn
XANt1	Transport, Extracellular	xanthine reversible transport				xan_e <-> xan	xan_e <-> xan	xan_e <-> xan
NACUP	Transport, Extracellular	Nicotinic acid uptake				nac_e -> nac	nac_e -> nac	nac_e -> nac
ASNabc	Transport, Extracellular	L-asparagine transport via ABC system				asn_e + atp + h2o -> adp + asn + pi	asn_e + atp + h2o -> adp + asn + h + pi	asn_e + atp + h2o -> adp + asn + h + pi
ASNtr	Transport, Extracellular	L-asparagine reversible transport via proton symport				asn_e + h_e <-> asn + h	asn_e + h_e <-> asn + h	asn_e + h_e <-> asn + h
DAPabc	Transport, Extracellular	M-diaminopimelic acid ABC transport				26dap-M_e + atp + h2o -> 26dap-M + adp + pi	26dap-M_e + atp + h2o -> 26dap-M + adp + h + pi	26dap-M_e + atp + h2o -> 26dap-M + adp + h + pi
CYSabc	Transport, Extracellular	L-cysteine transport via ABC system				atp + cys_e + h2o -> adp + cys + pi	atp + cys_e + h2o -> adp + cys + h + pi	atp + cys_e + h2o -> adp + cys + h + pi
ACtr	Transport, Extracellular	acetate reversible transport via proton symport				ac_e + h_e <-> ac + h	ac_e + h_e <-> ac + h	ac_e + h_e <-> ac + h
ETOHtr	Transport, Extracellular	ethanol reversible transport via proton symport				eth + h -> eth_e + h_e	eth + h -> eth_e + h_e	eth + h -> eth_e + h_e
PYRtr	Transport, Extracellular	pyruvate reversible transport via proton symport				h_e + pyr_e <-> h + pyr	h_e + pyr_e <-> h + pyr	h_e + pyr_e <-> h + pyr
O2t	Transport, Extracellular	o2 transport (diffusion)				o2_e <-> o2	o2_e <-> o2	o2_e <-> o2
CO2t	Transport, Extracellular	CO2 transporter via diffusion				co2_e <-> co2	co2_e <-> co2	co2_e <-> co2
H2Ot	Transport, Extracellular	H2O transport via diffusion				h2o_e <-> h2o	h2o_e <-> h2o	h2o_e <-> h2o
DHAt	Transport, Extracellular	Dihydroxyacetone transport via facilitated diffusion				glyn_e <-> glyn	glyn_e <-> glyn	glyn_e <-> glyn
NH3t	Transport, Extracellular	ammonia reversible transport				nh4_e <-> nh4	nh4_e <-> nh4	nh4_e <-> nh4

ARBtr	Transport, Extracellular	L-arabinose transport via proton symport	larabinose_e + h_e <-> larabinose + h	larabinose_e + h_e <-> larabinose + h	larabinose_e + h_e <-> larabinose + h
ARBabc	Transport, Extracellular	L-arabinose transport via ABC system	larabinose_e + atp + h2o -> adp + larabinose + pi	larabinose_e + atp + h2o -> adp + larabinose + h + pi	larabinose_e + atp + h2o -> adp + larabinose + h + pi
HIStr	Transport, Extracellular	L-histidine reversible transport via proton symport	h_e + his_e <-> h + his	h_e + his_e <-> h + his	h_e + his_e <-> h + his
PHEtr	Transport, Extracellular	L-phenylalanine reversible transport via proton symport	h_e + phe_e <-> h + phe	h_e + phe_e <-> h + phe	h_e + phe_e <-> h + phe
LEUtr	Transport, Extracellular	L-leucine reversible transport via proton symport	h_e + leu_e <-> h + leu	h_e + leu_e <-> h + leu	h_e + leu_e <-> h + leu
VALtr	Transport, Extracellular	L-valine reversible transport via proton symport	h_e + val_e <-> h + val	h_e + val_e <-> h + val	h_e + val_e <-> h + val
ILEtr	Transport, Extracellular	L-isoleucine reversible transport via proton symport	h_e + ile_e <-> h + ile	h_e + ile_e <-> h + ile	h_e + ile_e <-> h + ile
CBL1abc	Transport, Extracellular	Cob(1)alamin transport via ABC system	atp + cbl1_e + h2o -> adp + cbl1 + pi	atp + cbl1_e + h2o -> adp + cbl1 + h + pi	atp + cbl1_e + h2o -> adp + cbl1 + h + pi
CADVt	Transport, Extracellular	Lysine/Cadaverine antiporter	15dap + h_e + lys_e -> 15dap_e + h + lys	15dap + h_e + lys_e -> 15dap_e + h + lys	15dap + h_e + lys_e -> 15dap_e + h + lys
CRNt7	Transport, Extracellular	Carnitine/butyrobetaine antiporter	crn_e + gbbtn -> crn + gbbtn_e	crn_e + gbbtn -> crn + gbbtn_e	crn_e + gbbtn -> crn + gbbtn_e
NAt_1	Transport, Extracellular	sodium proton antiporter (H:NA is 1:1)	h_e + na <-> h + na_e	h_e + na <-> h + na_e	h_e + na <-> h + na_e
CITtsuc	Transport, Extracellular	Citrate transport via succinate antiport	cit_e + succ -> cit + succ_e	cit_e + succ -> cit + succ_e	cit_e + succ -> cit + succ_e
CSNt2	Transport, Extracellular	cytosine transport in via proton symport	ct_e + h_e -> ct + h	ct_e + h_e -> ct + h	ct_e + h_e -> ct + h
ACGApts	Transport, Extracellular	N-Acetyl-D-glucosamine transport via PEP:Pyr PTS	naga_e + pep -> naga6p + pyr	naga_e + pep -> naga6p + pyr + h	naga_e + pep -> naga6p + pyr + h
DALAtr	Transport, Extracellular	D-alanine transport via proton symport	dala_e + h_e <-> dala + h	dala_e + h_e <-> dala + h	dala_e + h_e <-> dala + h
DSERtr	Transport, Extracellular	D-serine transport via proton symport	h_e + dser_e <-> h + dser	h_e + dser_e <-> h + dser	h_e + dser_e <-> h + dser
GLYtr	Transport, Extracellular	glycine reversible transport via proton symport	gly_e + h_e <-> gly + h	gly_e + h_e <-> gly + h	gly_e + h_e <-> gly + h
SULabc	Transport, Extracellular	sulfate transport via ABC system	atp + h2o + so4_e -> adp + pi + so4	atp + h2o + so4_e -> adp + h + pi + so4	atp + h2o + so4_e -> adp + h + pi + so4
ASPt_2	Transport, Extracellular	Aspartate transport via proton symport (2 H)	asp_e + 2 h_e -> asp + 2 h	asp_e + 2 h_e -> asp + 2 h	asp_e + 2 h_e -> asp + 2 h
FUMt_2	Transport, Extracellular	Fumarate transport via proton symport (2 H)	fum_e + 2 h_e -> fum + 2 h	fum_e + 2 h_e -> fum + 2 h	fum_e + 2 h_e -> fum + 2 h
MALt_2	Transport, Extracellular	Malate transport via proton symport (2 H)	2 h_e + mal_e -> 2 h + mal	2 h_e + mal_e -> 2 h + mal	2 h_e + mal_e -> 2 h + mal
SUCCt_2	Transport, Extracellular	succinate transport via proton symport (2 H)	2 h_e + succ_e -> 2 h + succ	2 h_e + succ_e -> 2 h + succ	2 h_e + succ_e -> 2 h + succ
ASPt_3	Transport, Extracellular	L-asparate transport via proton symport (3 H)	asp_e + 3 h_e -> asp + 3 h	asp_e + 3 h_e -> asp + 3 h	asp_e + 3 h_e -> asp + 3 h
MALt_3	Transport, Extracellular	Malate transport via proton symport (3 H)	3 h_e + mal_e -> 3 h + mal	3 h_e + mal_e -> 3 h + mal	3 h_e + mal_e -> 3 h + mal
SUCCt_3	Transport, Extracellular	Succintate transport via proton symport (3 H)	3 h_e + succ_e -> 3 h + succ	3 h_e + succ_e -> 3 h + succ	3 h_e + succ_e -> 3 h + succ
SUCCet	Transport, Extracellular	Succinate efflux via proton symport	h + succ -> h_e + succ_e	h + succ -> h_e + succ_e	h + succ -> h_e + succ_e
FUMt_3	Transport, Extracellular	Fumarate transport via proton symport (3 H)	fum_e + 3 h_e -> fum + 3 h	fum_e + 3 h_e -> fum + 3 h	fum_e + 3 h_e -> fum + 3 h
SUCFUMt	Transport, Extracellular	succinate:fumarate antiporter	fum_e + succ <-> fum + succ_e	fum_e + succ <-> fum + succ_e	fum_e + succ <-> fum + succ_e
GALCTNtr	Transport, Extracellular	D-galactonate transport via proton symport, reversible	dgalctn_e + h_e <-> dgalctn + h	dgalctn_e + h_e <-> dgalctn + h	dgalctn_e + h_e <-> dgalctn + h
GLCURtr	Transport, Extracellular	D-glucuronate transport via proton symport, reversible	dgluc_e + h_e <-> dgluc + h	dgluc_e + h_e <-> dgluc + h	dgluc_e + h_e <-> dgluc + h
OCDCAt	Transport, Extracellular	Octadecanoate transport via proton symport	h_e + c180_e -> h + c180	h_e + c180_e -> h + c180	h_e + c180_e -> h + c180
HDCAt	Transport, Extracellular	Hexadecanoate transport via proton symport	h_e + c160_e -> h + c160	h_e + c160_e -> h + c160	h_e + c160_e -> h + c160
TTDCAt	Transport, Extracellular	Tetradecanoate transport via proton symport	h_e + c140_e -> h + c140	h_e + c140_e -> h + c140	h_e + c140_e -> h + c140
FE2abc	Transport, Extracellular	iron (II) transport via ABC system	atp + fe2_e + h2o -> adp + fe2 + pi	atp + fe2_e + h2o -> adp + fe2 + h + pi	atp + fe2_e + h2o -> adp + fe2 + h + pi
FORt	Transport, Extracellular	formate transport via diffusion	formate_e <-> formate	formate_e <-> formate	formate_e <-> formate
FUCt	Transport, Extracellular	L-fucose transport via proton symport	fuc_e + h_e <-> fuc + h	fuc_e + h_e <-> fuc + h	fuc_e + h_e <-> fuc + h
ABUTt	Transport, Extracellular	4-aminobutyrate transport in via proton symport	gaba_e + h_e -> gaba + h	gaba_e + h_e -> gaba + h	gaba_e + h_e -> gaba + h
GALTpts	Transport, Extracellular	Galactitol transport via PEP:Pyr PTS	galt_e + pep -> galt1p + pyr	galt_e + pep -> galt1p + pyr + h	galt_e + pep -> galt1p + pyr + h
GLNabc	Transport, Extracellular	L-glutamine transport via ABC system	atp + gln_e + h2o -> adp + gln + pi	atp + gln_e + h2o -> adp + gln + h + pi	atp + gln_e + h2o -> adp + gln + h + pi
GLYCt	Transport, Extracellular	glycerol transport via channel	gl <-> gl_e	gl <-> gl_e	gl <-> gl_e
GLYALDt	Transport, Extracellular	Glyceraldehyde facilitated diffusion	t3_e <-> t3	t3_e <-> t3	t3_e <-> t3
UREAt	Transport, Extracellular	Urea transport via facilitate diffusion	urea_e <-> urea	urea_e <-> urea	urea_e <-> urea
GLYC3Pt	Transport, Extracellular	Glycerol-3-phosphate : phosphate antiporter	glyc3p_e + pi -> glyc3p + pi_e	glyc3p_e + pi -> glyc3p + pi_e	glyc3p_e + pi -> glyc3p + pi_e
ASPabc	Transport, Extracellular	L-aspartate transport via ABC system	asp_e + atp + h2o -> adp + asp + pi	asp_e + atp + h2o -> adp + asp + h + pi	asp_e + atp + h2o -> adp + asp + h + pi
GLUabc	Transport, Extracellular	L-glutamate transport via ABC system	atp + glu_e + h2o -> adp + glu + pi	atp + glu_e + h2o -> adp + glu + h + pi	atp + glu_e + h2o -> adp + glu + h + pi
ASPt	Transport, Extracellular	L-aspartate transport in via proton symport	asp_e + h_e -> asp + h	asp_e + h_e -> asp + h	asp_e + h_e -> asp + h
GLUtr	Transport, Extracellular	L-glutamate transport via proton symport, reversible	glu_e + h_e <-> glu + h	glu_e + h_e <-> glu + h	glu_e + h_e <-> glu + h

GLUt	Transport, Extracellular	Na+/glutamate symport	glu_e + na_e -> glu + na	glu_e + na_e -> glu + na	glu_e + na_e -> glu + na
ORNabc	Transport, Extracellular	ornithine transport via ABC system	atp + h2o + orn_e -> adp + orn + pi	atp + h2o + orn_e -> adp + h + orn + pi	atp + h2o + orn_e -> adp + h + orn + pi
ARGabc	Transport, Extracellular	L-arginine transport via ABC system	arg_e + atp + h2o -> adp + arg + pi	arg_e + atp + h2o -> adp + arg + h + pi	arg_e + atp + h2o -> adp + arg + h + pi
HISabc	Transport, Extracellular	L-histidine transport via ABC system	atp + h2o + his_e -> adp + his + pi	atp + h2o + his_e -> adp + h + his + pi	atp + h2o + his_e -> adp + h + his + pi
LYSabc	Transport, Extracellular	L-lysine transport via ABC system	atp + h2o + lys_e -> adp + lys + pi	atp + h2o + lys_e -> adp + h + lys + pi	atp + h2o + lys_e -> adp + h + lys + pi
IDONtr	Transport, Extracellular	L-idonate transport via proton symport, reversible	h_e + idon_e <-> h + idon	h_e + idon_e <-> h + idon	h_e + idon_e <-> h + idon
GLCNtr	Transport, Extracellular	D-gluconate transport via proton symport, reversible	gluc_e + h_e <-> gluc + h	gluc_e + h_e <-> gluc + h	gluc_e + h_e <-> gluc + h
DDGLCNtr	Transport, Extracellular	2-dehydro-3-deoxy-D-gluconate transport via proton symport, reversible	kdg_e + h_e <-> kdg + h	kdg_e + h_e <-> kdg + h	kdg_e + h_e <-> kdg + h
Kabc	Transport, Extracellular	Potassium ABC transporter	atp + h2o + k_e -> adp + k + pi	atp + h2o + k_e -> adp + h + k + pi	atp + h2o + k_e -> adp + h + k + pi
LCTSt	Transport, Extracellular	Lactose transport via proton symport	h_e + lactose_e <-> h + lactose	h_e + lactose_e <-> h + lactose	h_e + lactose_e <-> h + lactose
ILEabc	Transport, Extracellular	L-isoleucine transport via ABC system	atp + h2o + ile_e -> adp + ile + pi	atp + h2o + ile_e -> adp + h + ile + pi	atp + h2o + ile_e -> adp + h + ile + pi
THRabc	Transport, Extracellular	L-threonine transport via ABC system	atp + h2o + thr_e -> adp + pi + thr	atp + h2o + thr_e -> adp + h + pi + thr	atp + h2o + thr_e -> adp + h + pi + thr
ALAabc	Transport, Extracellular	L-alanine transport via ABC system	ala_e + atp + h2o -> adp + ala + pi	ala_e + atp + h2o -> adp + ala + h + pi	ala_e + atp + h2o -> adp + ala + h + pi
VALabc	Transport, Extracellular	L-valine transport via ABC system	atp + h2o + val_e -> adp + pi + val	atp + h2o + val_e -> adp + h + pi + val	atp + h2o + val_e -> adp + h + pi + val
LEUabc	Transport, Extracellular	L-leucine transport via ABC system	atp + h2o + leu_e -> adp + leu + pi	atp + h2o + leu_e -> adp + h + leu + pi	atp + h2o + leu_e -> adp + h + leu + pi
DLACt	Transport, Extracellular	D-lactate transport via proton symport	h_e + lac_e <-> h + lac	h_e + lac_e <-> h + lac	h_e + lac_e <-> h + lac
GLYCLTtr	Transport, Extracellular	glycolate transport via proton symport, reversible	glycolate_e + h_e <-> glycolate + h	glycolate_e + h_e <-> glycolate + h	glycolate_e + h_e <-> glycolate + h
LLACtr	Transport, Extracellular	L-lactate reversible transport via proton symport	h_e + llac_e <-> h + llac	h_e + llac_e <-> h + llac	h_e + llac_e <-> h + llac
LYStr	Transport, Extracellular	L-lysine reversible transport via proton symport	h_e + lys_e <-> h + lys	h_e + lys_e <-> h + lys	h_e + lys_e <-> h + lys
MALTPTabc	Transport, Extracellular	maltopentaose transport via ABC system	atp + h2o + maltpt_e -> adp + maltpt + pi	atp + h2o + maltpt_e -> adp + h + maltpt + pi	atp + h2o + maltpt_e -> adp + h + maltpt + pi
MALTTTRabc	Transport, Extracellular	maltotetraose transport via ABC system	atp + h2o + mltttr_e -> adp + mltttr + pi	atp + h2o + mltttr_e -> adp + h + mltttr + pi	atp + h2o + mltttr_e -> adp + h + mltttr + pi
MALTHXabc	Transport, Extracellular	maltohexaose transport via ABC system	atp + h2o + mlthx_e -> adp + mlthx + pi	atp + h2o + mlthx_e -> adp + h + mlthx + pi	atp + h2o + mlthx_e -> adp + h + mlthx + pi
MALTTRabc	Transport, Extracellular	Maltotriose transport via ABC system	atp + h2o + mlttr_e -> adp + mlttr + pi	atp + h2o + mlttr_e -> adp + h + mlttr + pi	atp + h2o + mlttr_e -> adp + h + mlttr + pi
FRUpts2	Transport, Extracellular	Fructose transport via PEP:Pyr PTS (f6p generating)	fru_e + pep -> f6p + pyr	fru_e + pep -> f6p + pyr + h	fru_e + pep -> f6p + pyr + h
MANpts	Transport, Extracellular	D-mannose transport via PEP:Pyr PTS	man_e + pep -> man6p + pyr	man_e + pep -> man6p + pyr + h	man_e + pep -> man6p + pyr + h
GAMpts	Transport, Extracellular	D-glucosamine transport via PEP:Pyr PTS	gam_e + pep -> ga6p + pyr	gam_e + pep -> ga6p + pyr + h	gam_e + pep -> ga6p + pyr + h
MELIBt	Transport, Extracellular	melibiose transport in via symport	h_e + meli_e -> h + meli	h_e + meli_e -> h + meli	h_e + meli_e -> h + meli
METabc	Transport, Extracellular	L-methionine transport via ABC system	atp + h2o + met_e -> adp + met + pi	atp + h2o + met_e -> adp + h + met + pi	atp + h2o + met_e -> adp + h + met + pi
METDabc	Transport, Extracellular	D-methionine transport via ABC system	atp + h2o + dmet_e -> adp + dmet + pi	atp + h2o + dmet_e -> adp + h + dmet + pi	atp + h2o + dmet_e -> adp + h + dmet + pi
INDOLEtr	Transport, Extracellular	Indole transport via proton symport, reversible	h_e + indole_e <-> h + indole	h_e + indole_e <-> h + indole	h_e + indole_e <-> h + indole
ACNAMt	Transport, Extracellular	N-acetylneuraminate proton symport	naneu_e + h_e -> naneu + h	naneu_e + h_e -> naneu + h	naneu_e + h_e -> naneu + h
NO3t	Transport, Extracellular	nitrate transport in via nitrite antiport	no2 + no3_e -> no2_e + no3	no2 + no3_e -> no2_e + no3	no2 + no3_e -> no2_e + no3
NO2tr	Transport, Extracellular	nitrite transport in via proton symport, reversible	h_e + no2_e <-> h + no2	h_e + no2_e <-> h + no2	h_e + no2_e <-> h + no2
NAt_2	Transport, Extracellular	sodium proton antiporter (H:NA is 2)	2 h_e + na -> 2 h + na_e	2 h_e + na -> 2 h + na_e	2 h_e + na -> 2 h + na_e
NAt_1.5	Transport, Extracellular	sodium proton antiporter (H:NA is 1.5)	3 h_e + 2 na -> 3 h + 2 na_e	3 h_e + 2 na -> 3 h + 2 na_e	3 h_e + 2 na -> 3 h + 2 na_e
GSNt	Transport, Extracellular	guanosine transport in via proton symport	gsn_e + h_e -> gsn + h	gsn_e + h_e -> gsn + h	gsn_e + h_e -> gsn + h
DGSNt	Transport, Extracellular	deoxyguanosine transport in via proton symport	dg_e + h_e -> dg + h	dg_e + h_e -> dg + h	dg_e + h_e -> dg + h
INSt	Transport, Extracellular	inosine transport in via proton symport	h_e + ins_e -> h + ins	h_e + ins_e -> h + ins	h_e + ins_e -> h + ins
DINSt	Transport, Extracellular	deoxyinosine transport in via proton symport	din_e + h_e -> din + h	din_e + h_e -> din + h	din_e + h_e -> din + h
ADNt	Transport, Extracellular	adenosine transport in via proton symport	adn_e + h_e -> adn + h	adn_e + h_e -> adn + h	adn_e + h_e -> adn + h
URIt	Transport, Extracellular	uridine transport in via proton symport	h_e + uri_e -> h + uri	h_e + uri_e -> h + uri	h_e + uri_e -> h + uri
CYTDt	Transport, Extracellular	cytidine transport in via proton symport	cytd_e + h_e -> cytd + h	cytd_e + h_e -> cytd + h	cytd_e + h_e -> cytd + h
DCYTt	Transport, Extracellular	deoxycytidine transport in via proton symport	dc_e + h_e -> dc + h	dc_e + h_e -> dc + h	dc_e + h_e -> dc + h
DURIt	Transport, Extracellular	deoxyuridine transport in via proton symport	du_e + h_e -> du + h	du_e + h_e -> du + h	du_e + h_e -> du + h
DADNt	Transport, Extracellular	deoxyadenosine transport in via proton symport	da_e + h_e -> da + h	da_e + h_e -> da + h	da_e + h_e -> da + h
THMDt	Transport, Extracellular	thymidine transport in via proton symport	h_e + thymd_e -> h + thymd	h_e + thymd_e -> h + thymd	h_e + thymd_e -> h + thymd
PNTOt	Transport, Extracellular	Pantothenate sodium symporter	na_e + pnto_e -> na + pnto	na_e + pnto_e -> na + pnto	na_e + pnto_e -> na + pnto

PItr	Transport, Extracellular	phosphate reversible transport via symport	h_e + pi_e <-> h + pi	h_e + pi_e <-> h + pi	h_e + pi_e <-> h + pi
NMNP	Transport, Extracellular	NMN permease	namn_e -> namn	namn_e -> namn	namn_e -> namn
PTRCabc	Transport, Extracellular	putrescine transport via ABC system	atp + h2o + ptrc_e -> adp + pi + ptrc	atp + h2o + ptrc_e -> adp + h + pi + ptrc	atp + h2o + ptrc_e -> adp + h + pi + ptrc
SPMDabc	Transport, Extracellular	spermidine transport via ABC system	atp + h2o + sprmd_e -> adp + pi + sprmd	atp + h2o + sprmd_e -> adp + h + pi + sprmd	atp + h2o + sprmd_e -> adp + h + pi + sprmd
PTRCORNt	Transport, Extracellular	putrescine/ornithine antiporter	orn + ptrc_e <-> orn_e + ptrc	orn + ptrc_e <-> orn_e + ptrc	orn + ptrc_e <-> orn_e + ptrc
PTRCtr	Transport, Extracellular	putrescine transport in via proton symport, reversible	h_e + ptrc_e <-> h + ptrc	h_e + ptrc_e <-> h + ptrc	h_e + ptrc_e <-> h + ptrc
PROtr	Transport, Extracellular	L-proline reversible transport via proton symport	h_e + pro_e <-> h + pro	h_e + pro_e <-> h + pro	h_e + pro_e <-> h + pro
PROabc	Transport, Extracellular	L-proline transport via ABC system	atp + h2o + pro_e -> adp + pi + pro	atp + h2o + pro_e -> adp + h + pi + pro	atp + h2o + pro_e -> adp + h + pi + pro
PIabc	Transport, Extracellular	phosphate transport via ABC system	atp + h2o + pi_e -> adp + 2 pi	atp + h2o + pi_e -> adp + h + 2 pi	atp + h2o + pi_e -> adp + h + 2 pi
ACMANApts	Transport, Extracellular	N-acetyl-D-mannosamine transport via PTS	nadma_e + pep -> nadma6p + pyr	nadma_e + pep -> nadma6p + pyr + h	nadma_e + pep -> nadma6p + pyr + h
MNLpts	Transport, Extracellular	mannitol transport via PEP:Pyr PTS	mnt_e + pep -> mnt1p + pyr	mnt_e + pep -> mnt1p + pyr + h	mnt_e + pep -> mnt1p + pyr + h
FRUpts	Transport, Extracellular	D-fructose transport via PEP:Pyr PTS	fru_e + pep -> f1p + pyr	fru_e + pep -> f1p + pyr + h	fru_e + pep -> f1p + pyr + h
FRUabc	Transport, Extracellular	D-fructose transport via ABC system	atp + h2o + fru_e -> adp + pi + fru	atp + h2o + fru_e -> adp + h + pi + fru	atp + h2o + fru_e -> adp + h + pi + fru
PROt	Transport, Extracellular	Na+/Proline-L symporter	na_e + pro_e -> na + pro	na_e + pro_e -> na + pro	na_e + pro_e -> na + pro
RMNt	Transport, Extracellular	L-rhamnose transport via proton symport	h_e + rmn_e -> h + rmn	h_e + rmn_e -> h + rmn	h_e + rmn_e -> h + rmn
TSULabc	Transport, Extracellular	thiosulfate transport via ABC system	atp + h2o + tsul_e -> adp + pi + tsul	atp + h2o + tsul_e -> adp + h + pi + tsul	atp + h2o + tsul_e -> adp + h + pi + tsul
SERtr	Transport, Extracellular	L-serine reversible transport via proton symport	h_e + ser_e <-> h + ser	h_e + ser_e <-> h + ser	h_e + ser_e <-> h + ser
THMabc	Transport, Extracellular	thiamine transport via ABC system	atp + h2o + thiamin_e -> adp + pi + thiamin	atp + h2o + thiamin_e -> adp + h + pi + thiamin	atp + h2o + thiamin_e -> adp + h + pi + thiamin
SBTpts	Transport, Extracellular	D-sorbitol transport via PEP:Pyr PTS	pep + sot_e -> pyr + sbt6p	pep + sot_e -> pyr + sbt6p + h	pep + sot_e -> pyr + sbt6p + h
SERt	Transport, Extracellular	L-serine via sodium symport	na_e + ser_e -> na + ser	na_e + ser_e -> na + ser	na_e + ser_e -> na + ser
THRt	Transport, Extracellular	L-threonine via sodium symport	na_e + thr_e -> na + thr	na_e + thr_e -> na + thr	na_e + thr_e -> na + thr
TAURabc	Transport, Extracellular	taurine transport via ABC system	atp + h2o + taur_e -> adp + pi + taur	atp + h2o + taur_e -> adp + h + pi + taur	atp + h2o + taur_e -> adp + h + pi + taur
THRtr	Transport, Extracellular	L-threonine reversible transport via proton symport	h_e + thr_e <-> h + thr	h_e + thr_e <-> h + thr	h_e + thr_e <-> h + thr
TRPtr	Transport, Extracellular	L-tryptophan reversible transport via proton symport	h_e + trp_e <-> h + trp	h_e + trp_e <-> h + trp	h_e + trp_e <-> h + trp
Ktr	Transport, Extracellular	potassium reversible transport via proton symport	h_e + k_e <-> h + k	h_e + k_e <-> h + k	h_e + k_e <-> h + k
TYRtr	Transport, Extracellular	L-tyrosine reversible transport via proton symport	h_e + tyr_e <-> h + tyr	h_e + tyr_e <-> h + tyr	h_e + tyr_e <-> h + tyr
GLYC3Pabc	Transport, Extracellular	sn-Glycerol 3-phosphate transport via ABC system	atp + glyc3p_e + h2o -> adp + glyc3p + pi	atp + glyc3p_e + h2o -> adp + glyc3p + h + pi	atp + glyc3p_e + h2o -> adp + glyc3p + h + pi
MAN6Pt_2	Transport, Extracellular	Mannose-6-phosphate transport via phosphate antiport	man6p_e + 2 pi -> man6p + 2 pi_e	man6p_e + 2 pi -> man6p + 2 pi_e	man6p_e + 2 pi -> man6p + 2 pi_e
G6Pt_2	Transport, Extracellular	Glucose-6-phosphate transport via phosphate antiport	g6p_e + 2 pi -> g6p + 2 pi_e	g6p_e + 2 pi -> g6p + 2 pi_e	g6p_e + 2 pi -> g6p + 2 pi_e
FUCPt_2	Transport, Extracellular	Fucose 1-phosphate transport via phosphate antiport	fuc1p_e + 2 pi -> fuc1p + 2 pi_e	fuc1p_e + 2 pi -> fuc1p + 2 pi_e	fuc1p_e + 2 pi -> fuc1p + 2 pi_e
URAt	Transport, Extracellular	uracil transport in via proton symport	h_e + ura_e -> h + ura	h_e + ura_e -> h + ura	h_e + ura_e -> h + ura
XTSNtr	Transport, Extracellular	Xanthosine transport via proton symport	h_e + xtsine_e <-> h + xtsine	h_e + xtsine_e <-> h + xtsine	h_e + xtsine_e <-> h + xtsine
INStr	Transport, Extracellular	inosine transport in via proton symport, reversible	h_e + ins_e <-> h + ins	h_e + ins_e <-> h + ins	h_e + ins_e <-> h + ins
ADNtr	Transport, Extracellular	adenosine transport in via proton symport, reversible	adn_e + h_e <-> adn + h	adn_e + h_e <-> adn + h	adn_e + h_e <-> adn + h
CYTDtr	Transport, Extracellular	cytidine transport in via proton symport, reversible	cytd_e + h_e <-> cytd + h	cytd_e + h_e <-> cytd + h	cytd_e + h_e <-> cytd + h
THMDtr	Transport, Extracellular	thymidine transport in via proton symport, reversible	h_e + thymd_e <-> h + thymd	h_e + thymd_e <-> h + thymd	h_e + thymd_e <-> h + thymd
URItr	Transport, Extracellular	uridine transport in via proton symport, reversible	h_e + uri_e <-> h + uri	h_e + uri_e <-> h + uri	h_e + uri_e <-> h + uri
CHLtr	Transport, Extracellular	choline transport via proton symport, reversible	choline_e + h_e <-> choline + h	choline_e + h_e <-> choline + h	choline_e + h_e <-> choline + h
ADEtr	Transport, Extracellular	adenine transport via proton symport (reversible)	ad_e + h_e <-> ad + h	ad_e + h_e <-> ad + h	ad_e + h_e <-> ad + h
RIBabc	Transport, Extracellular	D-ribose transport via ABC system	atp + h2o + rib_e -> adp + pi + rib	atp + h2o + rib_e -> adp + h + pi + rib	atp + h2o + rib_e -> adp + h + pi + rib
FEabc	Transport, Extracellular		atp + fe3_e + h2o <-> fe3 + adp + pi	atp + fe3_e + h2o <-> fe3 + adp + h + pi	atp + fe3_e + h2o <-> fe3 + adp + h + pi
CRNabc	Transport, Extracellular		atp + h2o + crn_e <-> adp + crn + pi	atp + h2o + crn_e <-> adp + crn + h + pi	atp + h2o + crn_e <-> adp + crn + h + pi
MOBDabc	Transport, Extracellular		atp + mobd_e + h2o <-> adp + pi + mobd	atp + mobd_e + h2o <-> adp + h + pi + mobd	atp + mobd_e + h2o <-> adp + h + pi + mobd
TOLabc	Transport, Extracellular		atp + h2o + tolen_e <-> tolen + adp + pi	atp + h2o + tolen_e <-> tolen + adp + h + pi	atp + h2o + tolen_e <-> tolen + adp + h + pi
ASO3t1	Transport, Extracellular		aso3 <-> aso3_e	aso3 <-> aso3_e	aso3 <-> aso3_e
MG2t	Transport, Extracellular		mg2_e <-> mg2	mg2_e <-> mg2	mg2_e <-> mg2
COBTt	Transport, Extracellular		cobalt2 <-> cobalt2_e	cobalt2 <-> cobalt2_e	cobalt2 <-> cobalt2_e
ASPALAt	Transport, Extracellular		asp_e + ala <-> asp + ala_e	asp_e + ala <-> asp + ala_e	asp_e + ala <-> asp + ala_e
ASO3t2	Transport, Extracellular		aso3 -> aso3_e	aso3 -> aso3_e	aso3 -> aso3_e
BENZOTt	Transport, Extracellular		benzot_e + h_e -> benzot + h	benzot_e + h_e -> benzot + h	benzot_e + h_e -> benzot + h
NA1t	Transport, Extracellular		na_e + h <-> na + h_e	na_e + h <-> na + h_e	na_e + h <-> na + h_e

NA1t2	Transport, Extracellular					na + h_e <-> na_e + h	na + h_e <-> na_e + h	na + h_e <-> na_e + h
SUCCt	Transport, Extracellular					succ_e + h_e -> succ + h	succ_e + h_e -> succ + h	succ_e + h_e -> succ + h
SUCCtr	Transport, Extracellular					na_e + succ_e -> na + succ	na_e + succ_e -> na + succ	na_e + succ_e -> na + succ
SO4t	Transport, Extracellular					so4_e + na_e -> so4 + na	so4_e + na_e -> so4 + na	so4_e + na_e -> so4 + na
LYSt	Transport, Extracellular					lys + h_e -> lys_e + h	lys + h_e -> lys_e + h	lys + h_e -> lys_e + h
CITt	Transport, Extracellular					cit_e + h_e <-> cit + h	cit_e + h_e <-> cit + h	cit_e + h_e <-> cit + h
4HBZt	Transport, Extracellular					4hb_e + h_e <-> 4hb + h	4hb_e + h_e <-> 4hb + h	4hb_e + h_e <-> 4hb + h
3HBZt	Transport, Extracellular					3hbenzot_e + h_e <-> 3hbenzot + h	3hbenzot_e + h_e <-> 3hbenzot + h	3hbenzot_e + h_e <-> 3hbenzot + h
BENZt	Transport, Extracellular					benzot_e + h_e <-> benzot + h	benzot_e + h_e <-> benzot + h	benzot_e + h_e <-> benzot + h
PHBt	Transport, Extracellular					pbhb + h -> pbhb_e + h_e	pbhb + h -> pbhb_e + h_e	pbhb + h -> pbhb_e + h_e
PHENOLt	Transport, Extracellular					phenol_e + h_e -> phenol + h	phenol_e + h_e -> phenol + h	phenol_e + h_e -> phenol + h
PPAt	Transport, Extracellular					ppa_e + h_e -> ppa + h	ppa_e + h_e -> ppa + h	ppa_e + h_e -> ppa + h
C181t	Transport, Extracellular					c181_e + h_e -> c181 + h	c181_e + h_e -> c181 + h	c181_e + h_e -> c181 + h
BUTNt	Transport, Extracellular					butn_e + h_e -> butn + h	butn_e + h_e -> butn + h	butn_e + h_e -> butn + h
GENt	Transport, Extracellular					gensa_e + h_e -> gensa + h	gensa_e + h_e -> gensa + h	gensa_e + h_e -> gensa + h
2PGt	Transport, Extracellular					2pg_e + h_e -> 2pg + h	2pg_e + h_e -> 2pg + h	2pg_e + h_e -> 2pg + h
3PGt	Transport, Extracellular					3pg_e + h_e -> 3pg + h	3pg_e + h_e -> 3pg + h	3pg_e + h_e -> 3pg + h
4CRESOLt	Transport, Extracellular					4cresol_e + h_e -> 4cresol + h	4cresol_e + h_e -> 4cresol + h	4cresol_e + h_e -> 4cresol + h
6CHQt	Transport, Extracellular					6chq_e + h_e -> 6chq + h	6chq_e + h_e -> 6chq + h	6chq_e + h_e -> 6chq + h
CATECHt	Transport, Extracellular					catech_e + h_e -> catech + h	catech_e + h_e -> catech + h	catech_e + h_e -> catech + h
CLt	Transport, Extracellular					cl_e <-> cl	cl_e <-> cl	cl_e <-> cl
BP23Dt	Transport, Extracellular					bp23d_e + h_e -> bp23d + h	bp23d_e + h_e -> bp23d + h	bp23d_e + h_e -> bp23d + h
GLXt	Transport, Extracellular					glx_e + h_e -> glx + h	glx_e + h_e -> glx + h	glx_e + h_e -> glx + h
ICITt	Transport, Extracellular					icit_e + h_e <-> icit + h	icit_e + h_e <-> icit + h	icit_e + h_e <-> icit + h
ADIPt	Transport, Extracellular					adip_e + h_e <-> adip + h	adip_e + h_e <-> adip + h	adip_e + h_e <-> adip + h
PACt	Transport, Extracellular					pac_e + h_e <-> pac + h	pac_e + h_e <-> pac + h	pac_e + h_e <-> pac + h
CCMUCt	Transport, Extracellular					ccmuc_e + h_e <-> ccmuc + h	ccmuc_e + h_e <-> ccmuc + h	ccmuc_e + h_e <-> ccmuc + h
MCLACTt	Transport, Extracellular					mclact_e + h_e <-> mclact + h	mclact_e + h_e <-> mclact + h	mclact_e + h_e <-> mclact + h
34DHBt	Transport, Extracellular					34dhb_e + h_e -> 34dhb + h	34dhb_e + h_e -> 34dhb + h	34dhb_e + h_e -> 34dhb + h
KNTt	Transport, Extracellular					knt_e + h_e <-> knt + h	knt_e + h_e <-> knt + h	knt_e + h_e <-> knt + h
ANt	Transport, Extracellular					an_e + h_e <-> an + h	an_e + h_e <-> an + h	an_e + h_e <-> an + h
KNt	Transport, Extracellular					kn_e + h_e -> kn + h	kn_e + h_e -> kn + h	kn_e + h_e -> kn + h
AOBZACt	Transport, Extracellular					aobzac_e + h_e -> aobzac + h	aobzac_e + h_e -> aobzac + h	aobzac_e + h_e -> aobzac + h
ACONCt	Transport, Extracellular					acon-C_e + h_e -> acon-C + h	acon-C_e + h_e -> acon-C + h	acon-C_e + h_e -> acon-C + h
UROCANt	Transport, Extracellular					urocan_e + h_e <-> urocan + h	urocan_e + h_e <-> urocan + h	urocan_e + h_e <-> urocan + h
2HBAt	Transport, Extracellular					2hba_e + h_e -> 2hba + h	2hba_e + h_e -> 2hba + h	2hba_e + h_e -> 2hba + h
4HBTt	Transport, Extracellular					4hbt_e + h_e -> 4hbt + h	4hbt_e + h_e -> 4hbt + h	4hbt_e + h_e -> 4hbt + h
4HPHEACt	Transport, Extracellular					4hpheac_e + h_e -> 4hpheac + h	4hpheac_e + h_e -> 4hpheac + h	4hpheac_e + h_e -> 4hpheac + h
4HPHEAO	Transport, Extracellular					4hpheac + o2 + nadh + h -> homogen + nad + h2o	4hpheac + o2 + nadh + h -> homogen + nad + h2o	4hpheac + o2 + nadh + h -> homogen + nad + h2o
OBUTt	Transport, Extracellular					obut_e + h_e -> obut + h	obut_e + h_e -> obut + h	obut_e + h_e -> obut + h
R3HBNt	Transport, Extracellular					r3hbn_e + h_e -> r3hbn + h	r3hbn_e + h_e -> r3hbn + h	r3hbn_e + h_e -> r3hbn + h
BZALDt	Transport, Extracellular					bzald_e + h_e -> bzald + h	bzald_e + h_e -> bzald + h	bzald_e + h_e -> bzald + h
4FLRBZt	Transport, Extracellular					4flrbz_e + h_e -> 4flrbz + h	4flrbz_e + h_e -> 4flrbz + h	4flrbz_e + h_e -> 4flrbz + h
4FLTHRt	Transport, Extracellular					4flthr + h -> 4flthr_e + h_e	4flthr + h -> 4flthr_e + h_e	4flthr + h -> 4flthr_e + h_e
ASCBpts	Transport, Inner Membrane	L-ascorbate transport via PEP:Pyr PTS (periplasm)	2.7.1.69	H16_A0324	fruA	ascb_e + pep -> ascb6p + pyr	ascb_e + pep -> ascb6p + pyr + h	ascb_e + pep -> ascb6p + pyr + h
IMLTAP	Transport, Outer Membrane	ATPase (isomaltose)	3.6.1.-	H16_A2683/H16_A3646/ H16_B0538/H16_B1613	unknown/parA2/pox R/unknown	imal_e + atp + h2o -> imal + pi + adp	atp + h2o + imal_e -> imal + pi + adp + h	imal_e + atp + h2o -> imal + pi + adp + h
Maintenance						atp + h2o -> adp + pi	atp + h2o -> adp + pi + h	atp + h2o -> adp + pi + h
LPS	Lipopolysaccharide Biosynthesis / Recycling	Lipopolysaccharide biosynthesis				0.14 lipa + 0.42 adphep + 0.28 udpg + 0.28 cdpetn + 0.42 ckdo -> 0.42 adp + 0.28 udp + 0.42 cmp + 0.28 cdp + LPS	0.14 lipa + 0.42 adphep + 0.28 udpg + 0.28 cdpetn + 0.42 ckdo -> 0.42 adp + 0.28 udp + 0.42 cmp + 0.28 cdp + LPS	0.14 lipa + 0.42 adphep + 0.28 udpg + 0.28 cdpetn + 0.42 ckdo -> 0.42 adp + 0.28 udp + 0.42 cmp + 0.28 cdp + LPS
Protein						1.211 ala + 0.456 arg + 0.369 asn + 0.369 asp + 0.115 cys + 0.512 gln + 0.512 glu + 1.135 gly + 0.223 his + 0.306 ile + 0.522 leu + 0.189 lys + 0.159 met + 0.43 phe + 0.997 pro + 0.421 ser + 0.764 thr + 0.008 trp + 0.222 tyr + 0.687 val + 40 atp -> 40 adp + PROTEIN	1.211 ala + 0.456 arg + 0.369 asn + 0.369 asp + 0.115 cys + 0.512 gln + 0.512 glu + 1.135 gly + 0.223 his + 0.306 ile + 0.522 leu + 0.189 lys + 0.159 met + 0.43 phe + 0.997 pro + 0.421 ser + 0.764 thr + 0.008 trp + 0.222 tyr + 0.687 val + 40 atp -> 40 adp + PROTEIN	1.211 ala + 0.456 arg + 0.369 asn + 0.369 asp + 0.115 cys + 0.512 gln + 0.512 glu + 1.135 gly + 0.223 his + 0.306 ile + 0.522 leu + 0.189 lys + 0.159 met + 0.43 phe + 0.997 pro + 0.421 ser + 0.764 thr + 0.008 trp + 0.222 tyr + 0.687 val + 40 atp -> 40 adp + PROTEIN
DNA			2.7.7.7			0.564 datp + 1.054 dctp + 0.564 dttp + 1.054 dgtp + 4.4 atp -> 4.4 adp + 4.4 pi + DNA	0.564 datp + 1.054 dctp + 0.564 dttp + 1.054 dgtp + 4.4 atp -> 4.4 adp + 4.4 pi + DNA	0.564 datp + 1.054 dctp + 0.564 dttp + 1.054 dgtp + 4.4 atp -> 4.4 adp + 4.4 pi + DNA
RNA			2.7.7.6			0.631 atp + 0.75 gtp + 0.998 ctp + 0.747 utp -> 1.25 adp + 1.25 pi + RNA	0.631 atp + 0.75 gtp + 0.998 ctp + 0.747 utp -> 1.25 adp + 1.25 pi + RNA	0.631 atp + 0.75 gtp + 0.998 ctp + 0.747 utp -> 1.25 adp + 1.25 pi + RNA
Phospholipid						0.927 pe + 0.283 pg + 0.093 clpn -> PHOSPHOLIPID	0.927 pe + 0.283 pg + 0.093 clpn -> PHOSPHOLIPID	0.927 pe + 0.283 pg + 0.093 clpn -> PHOSPHOLIPID
Cofactors and vitamins (CAV)						0.656 pydxn + 0.145 coa + 0.141 fad + 0.243 fmn + 0.14 uq + 0.167 nad + 0.149 nadp + 0.249 thf + 0.418 thiamin -> CAV	0.656 pydxn + 0.145 coa + 0.141 fad + 0.243 fmn + 0.14 uq + 0.167 nad + 0.149 nadp + 0.249 thf + 0.418 thiamin -> CAV	0.656 pydxn + 0.145 coa + 0.141 fad + 0.243 fmn + 0.14 uq + 0.167 nad + 0.149 nadp + 0.249 thf + 0.418 thiamin -> CAV
Carbohydrate (CARBO)						3.937 udpnag + 0.984 udpacgal -> 4.921 udp + CARBO	3.937 udpnag + 0.984 udpacgal -> 4.921 udp + CARBO	3.937 udpnag + 0.984 udpacgal -> 4.921 udp + CARBO
Biomass						0.68 PROTEIN + 0.031 DNA + 0.06 RNA + 0.06 PEPTIDO + 0.055 CARBO + 0.03 CAV + 0.034 LPS + 0.0495 PHOSPHOLIPID + 15.3 atp -> BIOMASS + 15.3 adp + 15.3 pi	0.68 PROTEIN + 0.031 DNA + 0.06 RNA + 0.06 PEPTIDO + 0.055 CARBO + 0.03 CAV + 0.034 LPS + 0.0495 PHOSPHOLIPID + 15.3 atp -> BIOMASS + 15.3 adp + 15.3 pi	0.68 PROTEIN + 0.031 DNA + 0.06 RNA + 0.06 PEPTIDO + 0.055 CARBO + 0.03 CAV + 0.034 LPS + 0.0495 PHOSPHOLIPID + 15.3 atp -> BIOMASS + 15.3 adp + 15.3 pi
