# Supplementary material for: Genome-scale reconstruction and in silico analysis of the Ralstonia eutropha H16 for polyhydroxyalkanoate synthesis, lithoautotrophic growth, and 2-methyl citric acid production
Source: BMC Syst Biol. 2011 Jun 28;5:101. doi: 10.1186/1752-0509-5-101 (PMC3154180; doi:10.1186/1752-0509-5-101)
Supplement: Additional file 2 — List of metabolites in the genome-scale metabolic model of Ralstonia eutropha H16 [file 1752-0509-5-101-S2.PDF]

Additional file 2. List of metabolites in the genome-scale metabolic model of *Ralstonia eutropha* H16

abbreviation	Name	Name(sub)	Formula	Charge		
				pH 6	pH 7	pH 8
12anthcd	1,2-Anthracenediol		C14H10O2	0	0	0
12bhpp	1,2-Bis(4-hydroxyphenyl)-2-propanol		C15H16O3	0	0	0
12dhflu	1,2-Dihydroxyfluorene		C13H10O2	0	0	0
12ppd-R	(R)-Propane-1,2-diol	(R)-1,2-Propanediol/(R)-Propylene glycol	C3H8O2	0	0	0
12ppd-S	(S)-Propane-1,2-diol	(S)-1,2-Propanediol/(S)-Propylene glycol	C3H8O2	0	0	0
13pdg	3-Phospho-D-glyceroyl phosphate	1,3-Bisphospho-D-glycerate/(R)-2-Hydroxy-3-(phosphonooxy)-1-monoanhydride with phosphoric propanoic acid	C3H8O10P2	-3	-4	-4
14hpeth	1-(4'-Hydroxyphenyl)ethanol	4-Hydroxy-alpha-methyl-benzenemethanol	C8H10O2	0	0	0
15dap	1,5-Diaminopentane	Cadaverine/1,5-Pentanediamine/Pentamethylenediamine	C5H14N2	2	2	2
16dh24checc	1,6-Dihydroxy-cis-2,4-cyclohexadiene-1-carboxylic acid	cis-1,2-Dihydroxycyclohexa-3,5-diene-1-carboxylate	C7H8O4	-1	-1	-1
1boh	1-Butanol	n-Butanol	C4H10O	-1	-1	-1
22bhpp	2,2-Bis(4-hydroxyphenyl)-1-propanol		C15H16O3	0	0	0
23bhpp	2,3-Bis(4-hydroxyphenyl)-1,2-propanediol		C15H16O4	0	0	0
23doddt	2,3-Dihydroxy 1,1,1-Trichloro-2,2-bis(4'-chlorophenyl)ethane	2,3-Dihydroxy DDT	C14H9Cl5O2	0	0	0
246tchph	2,4,6-Trichlorophenol		C6H3Cl3O	-1	-1	-1
24dab	L-2,4-Diaminobutanoate	alpha,gamma-Diaminobutyrate/L-2,4-Diaminobutyrate	C4H10N2O2	1	1	1
24danit	2,4-Diamino-6-nitrotoluene		C7H9N3O2	0	0	0
25dop	2,5-Dioxopentanoate	2-Oxoglutarate semialdehyde	C5H6O4	-1	-1	-1
25drapp	2,5-Diamino-6-(5'-phosphoribosylamino)-4-pyrimidineone		C9H16N5O8P	-1	-2	-2
26dap-LL	LL-2,6-Diaminoheptanedioate	LL-2,6-Diaminopimelate/LL-2,6-Diaminopimelic acid	C7H14N2O4	0	0	0
26dap-M	meso-2,6-Diaminoheptanedioate	meso-2,6-Diaminopimelate/meso-2,6-Diaminopimelic acid/meso-Diaminoheptanedioate	C7H14N2O4	0	0	0
26dchhq	2,6-Dichlorohydroquinone		C6H4Cl2O2	0	-1	-1
26dchp	2,6-Dichlorophenol		C6H4Cl2O	0	-1	-1
26dhccoa	2,6-Dihydroxycyclohexane-1-carboxyl-CoA		C28H46N7O19P3S	-3	-4	-4
2a3cms	2-Amino-3-carboxymuconate semialdehyde	2-Amino-3-(3-oxoprop-1-enyl)-but-2-enedioate/2-Amino-3-(3-oxoprop-1-en-1-yl)but-2-enedioate	C7H7NO5	-1	-2	-2
2aa	2-Aminoacrylate	Dehydroalanine	C3H5NO2	0	0	0
2ag3pc	2-Acyl-sn-glycero-3-phosphocholine	2-Acylglycero-3-phosphocholine/1-Lysophosphatidylcholine/1-Lysolecithin/3-Lysolecithin	C9H20NO7PR	-1	-2	-2
2ag3pe	2-Acyl-sn-glycero-3-phosphoethanolamine	L-1-Lysophosphatidylethanolamine/O-(2-Acyl-sn-glycero-3-phospho)-ethanolamine/2-Acyl-sn-glycero-3-phosphoethanolamine	C6H13NO7PR	0	0	0
2ag3ps	2-Acyl-sn-glycero-3-phosphoserine		C8H13NO10PR	-2	-2	-2
2amc	2-Aminomuconate	2-Aminomuconic acid/o-Aminomuconate	C6H7NO4	-1	-1	-1
2amcs	2-Aminomuconate semialdehyde	2-Aminomuconate 6-semialdehyde	C6H7NO3	0	0	-1
2amoeccoa	2-Amino-5-oxocyclohex-1-enecarbonyl-CoA	2-Amino-5-oxo-cyclohex-1-enecarboxy-CoA	C28H43N8O18P3S	-3	-4	-4
2aobut	L-2-Amino-3-oxobutanoate	L-2-Amino-3-oxobutanoic acid/L-2-Amino-acetoacetate/(S)-2-Amino-3-oxobutanoic acid	C4H7NO3	0	0	-1
2bromoac	2-Bromomaleylacetate		C6H5BrO5	-2	-2	-2
2c4h6at	2-Chloro-4-hydroxy-6-amino-1,3,5-triazine		C3H3ClN4O	0	0	0
2chmac	2-Chloromaleylacetate		C6H5ClO5	-2	-2	-2
2cpr5p	1-(2-Carboxyphenylamino)-1'-deoxy-D-ribulose 5'-phosphate		C12H16NO9P	-2	-3	-3
2dhglcn6p	2-Dehydro-D-gluconate 6-phosphate	6-phospho-2-dehydro-D-gluconate	C6H11O10P	-2	-3	-3
2dmmq8	2-Demethylmenaquinone		C15H14O2(C5H8)n	0	0	0
2e5mhdcoa	(2E)-5-Methylhexa-2,4-dienoyl-CoA		C28H44N7O17P3S	-3	-4	-4
2fchdc	2-Fluorocyclohexadiene-cis,cis-1,2-diol-1-carboxylate		C7H7FO4	-1	-1	-1
2flcmuc	2-Fluoro-cis,cis-muconate		C6H5FO4	-2	-2	-2
2flrbz	2-Fluorobenzoate	2-Fluorobenzoic acid	C7H5FO2	-1	-1	-1
2h2hpd	2-Hydroxy-2-hydropyrone-4,6-dicarboxylate		C7H6O6	-2	-2	-2
2h3cbzpyr	2-Hydroxy-3-carboxybenzalpyruvate	3-(3-Carboxy-3-oxo-1-propenyl)-2-hydroxy-benzoic acid	C11H8O6	-2	-2	-2
2h3mbzpyr	2-Hydroxy-3-methylbenzalpyruvate		C11H10O4	-1	-1	-1
2h4hmbnpyr	2-Hydroxy-4-hydroxymethylbenzalpyruvate		C11H10O5	-1	-1	-1
2h6ot	2-Hydroxy-6-oxoocta-2,4,7-trienoate		C8H8O4	-1	-1	-1
2hba	2-Hydroxybutanoic acid	2-Hydroxybutyrate/2-Hydroxybutyric acid	C4H8O3	-1	-1	-1
2hhpdd	2-Hydroxyhepta-2,4-dienedioate	2-Hydroxyhepta-2,4-diene-1,7-dioate	C7H8O5	-2	-2	-2
2hlmdnit	2-Hydroxylamino-4,6-dinitrotoluene		C7H7N3O5	0	0	0
2hmuc	2-Hydroxymuconate		C6H6O5	-2	-2	-2
2hmucsald	2-Hydroxymuconate semialdehyde	2-Hydroxymuconic semialdehyde	C6H6O4	-1	-1	-1

2hohphd	2-Hydroxy-6-oxo-6-(2-hydroxyphenyl)-hexa-2,4-dienoate		C12H10O5		-1	-1	-1
2hohphod	2-Hydroxy-6-oxo-6-(2-hydroxyphenoxy)-hexa-2,4-dienoate		C12H10O6		-1	-1	-1
2hpa	2-Hydroxyphenylacetate		C8H8O3		-1	-1	-1
2ippm	2-Isopropylmaleate	beta-Isopropylmaleate	C7H10O4		-2	-2	-2
2kchccoa	2-Ketocyclohexane-1-carboxyl-CoA	2-Oxocyclohexane-1-carbonyl-CoA	C28H44N7O18P3S		-3	-4	-4
2kmb	4-Methylthio-2-oxobutanoate	4-Methylthio-2-oxobutanoic acid	C5H8O3S		-1	-1	-1
2m2ecoa	2-Methylbut-2-enoyl-CoATiglyl-CoA	(E)-2-Methylcrotonoyl-CoA/Methylcrotonoyl-CoA/Methylcrotonyl-CoA/Tigloyl-CoA/2-Methylcrotanoyl-CoA	C26H42N7O17P3S		-3	-4	-4
2maaccoa	2-Methylacetoacetyl-CoA	2-Methyl-3-acetoacetyl-CoA	C26H42N7O18P3S		-3	-4	-4
2mac	2-Maleylacetate	4-Oxohex-2-enedioate/Maleylacetate	C6H6O5		-2	-2	-2
2mbcoa	(S)-2-Methylbutanoyl-CoA		C26H44N7O17P3S		-3	-4	-4
2mcit	2-Methylcitrate	2-Hydroxybutane-1,2,3-tricarboxylate/(2R,3S)-2-Hydroxybutane-1,2,3-tricarboxylate	C7H10O7		-3	-3	-3
2mm	2-Methylmaleate	Citraconate/Citraconic acid/Methylmaleic acid	C5H6O4		-2	-2	-2
2mnapth	2-Methylnaphthalene	beta-Methylnaphthalene	C11H10		0	0	-1
2mp2ecoa	2-Methylprop-2-enoyl-CoA	Methacrylyl-CoA/Methylacrylyl-CoA	C25H40N7O17P3S		-3	-4	-4
2mppacoa	2-Methylpropanoyl-CoA	2-Methylpropionyl-CoA/Isobutyryl-CoA	C25H42N7O17P3S		-3	-4	-4
2napald	2-Naphthaldehyde	2-Naphthalenecarboxaldehyde	C11H8O		0	0	0
2naptcoa	2-Naphthoyl-CoA		C32H42N7O17P3S		-3	-4	-4
2naptha	2-Naphthoic acid	2-Naphthalenecarboxylic acid/beta-Naphthoic acid	C11H8O2		-1	-1	-1
2napthm	(2-Naphthyl)methanol	2-Naphthalenemethanol/2-Hydroxymethylnaphthalene	C11H10O		0	0	0
2o3e	2-Oxohept-3-enedioate	2-Oxohept-3-ene-1,7-dioate	C7H8O5		-2	-2	-2
2oad	2-Oxoadipate	2-Oxoadipic acid	C6H8O5		-2	-2	-2
2odhfac	2-Oxo-2,3-dihydrofuran-5-acetate	3-Oxoadipate enol-lactone/4,5-Dihydro-5-oxofuran-2-acetate/5-Oxo-4,5-dihydrofuran-2-acetate	C6H6O4		-1	-1	-1
2ombzl	2-Octaprenyl-6-methoxy-1,4-benzoquinol			0	0	0	0
2omhmbl	2-Octaprenyl-3-methyl-5-hydroxy-6-methoxy-1,4-benzoquinone		C48H72O4		0	0	0
2ommbl	2-Octaprenyl-3-methyl-6-methoxy-1,4-benzoquinone		C48H72O3		0	0	0
2op6hp	2-Octaprenyl-6-hydroxyphenol		C46H70O2		0	0	0
2opmp	2-Octaprenyl-6-methoxyphenol		C47H72O2		0	0	0
2opp	2-Octaprenylphenol		C46H70O		0	0	0
2p1a	2-Propyn-1-al		C3H2O		0	0	0
2pcdpmde	2-phospho-4-(cytidine 5'-diphospho)-2-C-methyl-D-erythritol		C14H26N3O17P3		-3	-4	-4
2pg	D-Glycerate 2-phosphate	2-Phospho-D-glycerate	C3H7O7P		-2	-3	-3
2ppg	2-Phosphoglycolate	Phosphoglycolic acid	C2H5O6P		-2	-3	-3
2py46dc	2-Pyrone-4,6-dicarboxylate		C7H4O6		-2	-2	-2
34dhb	3,4-Dihydroxybenzoate	3,4-Dihydroxybenzoic acid/Protocatechuate/Protocatechuic acid	C7H6O4		-1	-1	-1
34dhflu	3,4-Dihydroxyfluorene		C13H10O2		0	0	0
34dhma	3,4-Dihydroxymandelaldehyde		C8H8O4		0	0	0
34dhpac	3,4-Dihydroxyphenylacetaldehyde	Protocatechuatealdehyde	C8H8O3		0	0	0
34dhpeg	3,4-Dihydroxyphenylethyleneglycol		C8H10O4		0	0	0
34dhpheac	3,4-Dihydroxyphenylacetate	3,4-Dihydroxyphenylacetic acid/3,4-Dihydroxyphenyl acetate/3,4-Dihydroxyphenyl acetic acid/Homoprotocatechuate	C8H8O4		-1	-1	-1
3a2op	3-Amino-2-oxopropyl phosphate	1-Amino-3-(phosphohydroxy)propan-2-one	C3H8NO5P		0	-1	-2
3aibt	3-Aminoisobutanoate	3-Amino-2-methylpropanoate	C4H9NO2		0	0	0
3b1a	3-Butyn-1-al		C4H4O		0	0	0
3btecoa	3-Butenoyl-CoA	Vinylacetyl-CoA	C25H40N7O17P3S		-3	-4	-4
3but	3-Butynoate		C4H4O2		-1	-1	-1
3c2hmp	3-Carboxy-2-hydroxy-4-methylpentanoate	(2R,3S)-3-Isopropylmalate/3-Isopropylmalate/2-D-threo-Hydroxy-3-carboxy-isocaproate	C7H12O5		-2	-2	-2
3c3hmp	3-Carboxy-3-hydroxy-4-methylpentanoate	(2S)-2-Isopropylmalate/2-Isopropylmalate/2-Isopropylmalic acid/3-Carboxy-3-hydroxy-isocaproate/3-Carboxy-3-hydroxyisocaproate/2-Hydroxy-2-isopropylbutanedioate/3-Hydroxy-4-methyl-3-carboxypentanoate	C7H12O5		-2	-2	-2
3chccm	3-Chloro-cis,cis-muconate		C6H5ClO4		-2	-2	-2
3chroald	3-Chloroallyl aldehyde	trans-3-Chloroallyl aldehyde	C3H3ClO		0	0	0
3cresol	3-Cresol	m-Cresol/3-Hydroxytoluene	C7H8O		0	0	0
3ddah7p	2-Dehydro-3-deoxy-D-arabino-heptonate 7-phosphate	3-Deoxy-D-arabino-hept-2-ulosonate 7-phosphate/3-Deoxy-arabino-heptulonate 7-phosphate/3-Deoxy-D-arabino-heptulosonic acid 7-phosphate/DAHP/2-Dahp	C7H13O10P		-2	-3	-3
3fchdc	3-Fluorocyclohexadiene-cis,cis-1,2-diol-1-carboxylate		C7H7FO4		-1	-1	-1
3flcmuc	3-Fluoro-cis,cis-muconate		C6H5FO4		-2	-2	-2
3flrbz	3-Fluorobenzoate	3-Fluorobenzoic acid	C7H5FO2		-1	-1	-1
3flrcatech	3-Fluorocatechol		C6H5FO2		0	0	0
3h2napth	3-Hydroxy-2-naphthoate		C11H8O3		-1	-1	-1

3h3mgcoa	(S)-3-Hydroxy-3-methylglutaryl-CoA	Hydroxymethylglutaryl-CoA/Hydroxymethylglutaroyl coenzyme A/HMG-CoA/3-Hydroxy-3-methylglutaryl-CoA	C27H44N7O20P3S	-4	-5	-5
3h5m4ecoa	3-Hydroxy-5-methylhex-4-enoyl-CoA		C28H46N7O18P3S	-3	-4	-4
3h5ohcoa	3-Hydroxy-5-oxohexanoyl-CoA		C27H44N7O19P3S	-3	-4	-4
3han	3-Hydroxyanthranilate	3-Hydroxyanthranilic acid	C7H7NO3	-1	-1	-1
3hbcoa	(S)-3-Hydroxybutanoyl-CoA	(S)-3-Hydroxybutyryl-CoA	C25H42N7O18P3S	-3	-4	-4
3hbenzot	3-Hydroxybenzoate	m-Hydroxybenzoic acid	C7H6O3	-1	-1	-1
3hbzald	3-Hydroxybenzaldehyde		C7H6O2	0	0	0
3hcinnm	trans-3-Hydroxycinnamate	3-Coumaric acid	C9H8O3	-1	-1	-1
3hibcoa	(S)-3-Hydroxyisobutyryl-CoA		C25H42N7O18P3S	-3	-4	-4
3hivcoa	3-Hydroxyisovaleryl-CoA	3-Hydroxyisovaleryl coenzyme A	C26H44N7O18P3S	-3	-4	-4
3hmrsACP	(3R)-3-Hydroxytetradecanoyl-[acyl-carrier protein]	(R)-3-Hydroxytetradecanoyl-[acyl-carrier protein]/beta-Hydroxymyristyl-[acyl-carrier protein]/HMA	C14H27O2SR	-1	-1	-1
3hpcoa	3-Hydroxypropionyl-CoA	3-Hydroxypropionyl coenzyme A/3-Hydroxypropanoyl-CoA/3-Hydroxypropanoyl coenzymeA	C24H40N7O18P3S	-3	-4	-4
3hpp	3-Hydroxypropanoate	3-Hydroxypropanoic acid/3-Hydroxypropionate/3-Hydroxypropionic acid/Hydracrylic acid	C3H6O3	-1	-1	-1
3hpppn	3-(3-Hydroxy-phenyl)-propanoic acid	Dihydro-3-coumaric acid/3-Hydroxyphenylpropanoate	C9H10O3	-1	-1	-1
3ig3p	C1-(3-Indolyl)-glycerol 3-phosphate	Indoleglycerol phosphate/1-C-(Indol-3-yl)glycerol 3-phosphate/(3-Indolyl)-glycerol phosphate/(1S,2R)-1-C-(Indol-3-yl)glycerol 3-phosphate/Indole-3-glycerol phosphate	C11H14NO6P	-1	-2	-2
3m4hpa	3-Methoxy-4-hydroxyphenylacetaldehyde		C9H10O3	0	0	0
3m4hpg	3-Methoxy-4-hydroxyphenylglycolaldehyde		C9H10O4	0	0	0
3mbcoa	3-Methylbutanoyl-CoA	Isovaleryl-CoA	C26H44N7O17P3S	-3	-4	-4
3mccoa	3-Methylcrotonyl-CoA	3-Methylbut-2-enoyl-CoA/3-Methylcrotonoyl-CoA/Dimethylacryloyl-CoA	C26H42N7O17P3S	-3	-4	-4
3mhdd	3-Methyl-cis,cis-hexadienedioate	3-Methyl-cis,cis-muconate	C7H8O4	-2	-2	-2
3mlac	3-Mercaptolactate		C3H5O3S	-1	-1	-1
3mob	3-Methyl-2-oxobutanoate	3-Methyl-2-oxobutanoic acid/3-Methyl-2-oxobutyric acid/2-Oxo-3-methylbutanoate/2-Oxoisovalerate/2-Oxoisopentanoate/alpha-Ketovaline/2-Ketovaline/2-Keto-3-methylbutyric acid	C5H8O3	-1	-1	-1
3mop	(S)-3-Methyl-2-oxopentanoate	(S)-3-Methyl-2-oxopentanoic acid/(3S)-3-Methyl-2-oxopentanoic acid/(3S)-3-Methyl-2-oxopentanoate	C6H10O3	-1	-1	-1
3o4mp	3-Oxo-4-methylpentanoic acid	3-Oxo-4-methylpentanoate/(R)-4-Methyl-3-oxopentanoate	C6H10O3	-1	-1	-1
3op4hb	3-Octaprenyl-4-hydroxybenzoate		C47H70O3	-1	-1	-1
3opp	3-Oxopropanoate	Malonate semialdehyde	C3H4O3	-1	-1	-1
3oppcoa	3-Oxopropionyl-CoA		C24H38N7O18P3S	-3	-4	-4
3pg	3-Phospho-D-glycerate	D-Glycerate 3-phosphate/3-Phospho-(R)-glycerate	C3H7O7P	-2	-3	-3
3php	3-Phosphohydroxypyruvate	3-Phosphonooxypyruvate/3-Phosphonooxypyruvic acid/3-Phosphohydroxypyruvic acid	C3H5O7P	-2	-3	-3
3psme	5-O-(1-Carboxyvinyl)-3-phosphoshikimate	O5-(1-Carboxyvinyl)-3-phosphoshikimate	C10H13O10P	-3	-4	-4
3rbleu	(3R)-beta-Leucine	(3R)-beta-2-Amino-4-methylvaleric acid/L-beta-Leucine	C6H13NO2	0	0	0
3sfpyr	3-Sulfinylpyruvate	3-Sulfinopyruvate	C3H4O5S	-1	-1	-2
3slala	3-Sulfino-L-alanine	L-Cysteinesulfinic acid/3-Sulphino-L-alanine/3-Sulfinoalanine	C3H7NO4S	-1	-1	-1
3spyr	3-Sulfopyruvate	3-Sulfopyruvic acid	C3H4O6S	-2	-2	-2
3sulmc	3-Sulfomuconate		C6H6O7S	-3	-3	-3
3udsb	3-Ureidoisobutyrate		C5H10N2O3	-1	-1	-1
3urdpp	3-Ureidopropionate	3-Ureidopropanoate/beta-Ureidopropionic acid/N-Carbamoyl-beta-alanine	C4H8N2O3	-1	-1	-1
3vcat	3-Vinylcatechol		C8H8O2	0	0	0
46dhqn	4,6-Dihydroxyquinoline	Quinoline-4,6-diol	C9H7NO2	0	0	0
48dhq	4,8-Dihydroxyquinoline	Quinoline-4,8-diol	C9H7NO2	0	0	0
4aabut	4-Acetamidobutanoate	N4-Acetylaminobutanoate	C6H11NO3	-1	-1	-1
4aanit	4-Acetamido-2-amino-6-nitrotoluene		C9H11N3O3	0	0	0
4ab	4-Aminobutanal	4-Aminobutyraldehyde/Butyraldehyde, 4-amino-	C4H9NO	1	1	1
4ampm	4-Amino-2-methyl-5-phosphomethylpyrimidine	4-Amino-5-phosphomethyl-2-methylpyrimidine	C6H10N3O4P	0	-2	-2
4c2hhd	4-Carboxy-2-hydroxyhexa-2,4-dienedioate		C7H6O7	-3	-3	-3
4c2hmucsah	4-Carboxy-2-hydroxymuconate semialdehyde	4-Carboxy-2-hydroxy-cis,cis-muconate 6-semialdehyde	C7H6O6	-2	-2	-2
4c2o4pent	4-Carboxy-2-oxo-4-pentanoate		C6H6O5	-2	-2	-2
4c4h2oadip	4-Carboxy-4-hydroxy-2-oxoadipate	4-Hydroxy-4-carboxymethyl-2-oxoglutarate/2-Hydroxy-4-oxobutane-1,2,4-tricarboxylate	C7H8O8	-3	-3	-3
4chbenz	4-Chlorobenzoate	4-Chlorobenzoic acid	C7H5ClO2	-1	-1	-1

4chcat	4-Chlorocatechol		C6H5ClO2	0	0	0
4chphac	4-Chlorophenylacetate	4-Chlorophenylacetic acid/4-Chlorophenyl acetate/4-Chlorophenyl acetic acid	C8H7ClO2	-1	-1	-1
4cresol	4-Cresol	p-Cresol/4-Hydroxytoluene/4-Methylphenol	C7H8O	0	0	0
4ethp	4-Ethylphenol		C8H10O	0	0	0
4faac	4-Fumarylacetoacetate	4-Fumarylacetoacetic acid/Fumarylacetoacetate	C8H8O6	-2	-2	-2
4fchcd	4-Fluorocyclohexadiene-cis,cis-1,2-diol		C6H7FO2	0	0	0
4fchdc	4-Fluorocyclohexadiene-cis,cis-1,2-diol-1-carboxylate		C7H7FO4	-1	-1	-1
4flrbz	4-Fluorobenzoate	4-Fluorobenzoic acid	C7H5FO2	-1	-1	-1
4flrcatech	4-Fluorocatechol		C6H5FO2	0	0	0
4flthr	4-Fluoro-L-threonine		C4H8FNO3	0	0	0
4fmuclac	4-Fluoromuconolactone		C6H5FO4	-1	-1	-1
4h2ohep	4-Hydroxy-2-oxo-heptanedioate	4-Hydroxy-2-ketopimelate/4-Hydroxy-2-oxoheptanedioic acid	C7H10O6	-2	-2	-2
4hac	4-Hydroxyphenylacetaldehyde	2-(4-Hydroxyphenyl)acetaldehyde	C8H8O2	0	0	0
4hactph	4'-Hydroxyacetophenone	(4-Hydroxyphenyl)ethan-1-one	C8H8O2	0	0	-1
4hb	4-Hydroxybenzoate	Hydroxybenzoic acid/4-Hydroxybenzoic acid/Hydroxybenzenecarboxylic acid	C7H6O3	-1	-1	-1
4hbt	4-Hydroxybutanoic acid	4-Hydroxybutanoate/4-Hydroxybutyric acid	C4H8O3	-1	-1	-1
4hbzald	4-Hydroxybenzaldehyde	p-Hydroxybenzaldehyde	C7H6O2	0	0	-1
4hbzcoa	4-Hydroxybenzoyl-CoA		C28H40N7O18P3S	-3	-4	-4
4hglusa	L-4-Hydroxyglutamate semialdehyde		C5H9NO4	0	0	0
4hlmdnit	4-Hydroxylamino-2,6-dinitrotoluene		C7H7N3O5	0	0	0
4hlt	4-Hydroxy-L-threonine		C4H9NO4	0	0	0
4hmcatech	4-Hydroxymethylcatechol		C7H8O3	0	0	0
4hmdn	4-Hydroxymandelonitrile		C8H7NO2	0	0	0
4hmsalc	4-Hydroxymethylsalicylate	2-Hydroxy-4-hydroxymethylbenzoic acid	C8H8O4	-1	-1	-1
4hmscald	4-Hydroxymethylsalicylaldehyde	2-Hydroxy-4-hydroxymethyl-benzaldehyde	C8H8O3	0	0	0
4hpheac	4-Hydroxyphenylacetate	4-Hydroxyphenylacetic acid	C8H8O3	-1	-1	-1
4hpheacoa	4-Hydroxyphenylacetyl-CoA		C29H42N7O18P3S	-3	-4	-4
4hphegly	4-Hydroxyphenylacetylglycine	p-Hydroxyphenylacetylglycine	C10H11NO4	-1	-1	-1
4hpheglyx	4-Hydroxyphenylglyoxylate	4-Hydroxybenzoylformate	C8H6O4	-1	-1	-2
4hpp	3-(4-Hydroxyphenyl)pyruvate	4-Hydroxyphenylpyruvate, p-Hydroxyphenylpyruvic acid	C9H8O4	-1	-1	-1
4hpro	trans-4-Hydroxy-L-proline		C5H9NO3	0	0	0
4i5p	4-Imidazolone-5-propanoate	4-Imidazolone-5-propionic acid/4,5-Dihydro-4-oxo-5-imidazolepropanoate	C6H8N2O3	-1	-2	-2
4maac	4-Maleylacetoacetate	4-Maleylacetoacetic acid	C8H8O6	-2	-2	-2
4mctch	4-Methylcatechol	3,4-Dihydroxytoluene/1,2-Dihydroxy-4-methylbenzene/4-Methyl-1,2-benzenediol	C7H8O2	0	0	0
4mhetz	5-(2-Hydroxyethyl)-4-methylthiazole	4-Methyl-5-(2'-hydroxyethyl)-thiazole/4-Methyl-5-(2-hydroxyethyl)-thiazole	C6H9NOS	0	0	0
4mmclac	4-Methylmuconolactone	4-Carboxymethyl-4-methylbut-2-en-1,4-olide	C7H8O4	-1	-1	-1
4mop	4-Methyl-2-oxopentanoate	2-Oxoisocaproate	C6H10O3	-1	-1	-1
4obtc	(E)-4-Oxobut-1-ene-1,2,4-tricarboxylate	4-Oxalomesaconate/4-Oxalmesaconic acid	C7H6O7	-3	-3	-3
4ppcys	N-[(R)-4'-Phosphopantothenoyl]-L-cysteine	(R)-4'-Phosphopantothenoyl-L-cysteine	C12H23N2O9PS	-2	-3	-3
4ppnte	Pantetheine 4'-phosphate	4'-Phosphopantetheine/Phosphopantetheine/D-Pantetheine 4'-phosphate	C11H23N2O7PS	-1	-2	-2
4ppnto	D-4'-Phosphopantothenate	(R)-4'-Phosphopantothenate	C9H18NO8P	-2	-3	-3
4sulcat	4-Sulfocatechol		C6H6O5S	-1	-1	-1
4sullac	4-Sulfolactone		C6H6O7S	-2	-2	-2
56dht	5,6-Dihydrothymine	Dihydrothymine/5,6-Dihydro-5-methyluracil	C5H8N2O2	0	0	0
56dhu	5,6-Dihydrouracil	2,4(1H,3H)-Pyrimidinedione, dihydro-/Dihydrouracile/Dihydrouracil/5,6-Dihydro-2,4-dihydroxypyrimidine/Hydrouracil	C4H6N2O2	0	0	0
5a4ic	5-Amino-4-imidazolecarboxyamide		C4H6N4O	0	0	0
5aop	5-Amino-4-oxopentanoate	5-Aminolevulinate/5-Amino-4-oxovaleric acid	C5H9NO3	0	0	-1
5c2o3e	5-Carboxy-2-oxohept-3-enedioate	5-Oxopent-3-ene-1,2,5-tricarboxylate	C8H8O7	-3	-3	-3
5ch2hmcs	5-Chloro-2-hydroxymuconic semialdehyde		C6H5ClO4	-1	-1	-1
5cm2hm	5-Carboxymethyl-2-hydroxymuconate		C8H8O7	-3	-3	-3
5co46d	5-(3'-Carboxy-3'-oxopropenyl)-4,6-dihydroxypicolinate		C10H7NO7	-2	-3	-3
5co46dp	5-(3'-Carboxy-3'-oxopropyl)-4,6-dihydroxypicolinate	5-(gamma-Carboxy-gamma-oxopropyl)-4,6-dihydroxypicolinate	C10H9NO7	-3	-3	-3
5fchdc	5-Fluorocyclohexadiene-cis,cis-1,2-diol-1-carboxylate		C7H7FO4	-1	-1	-1
5fmuclac	5-Fluoromuconolactone		C6H5FO4	-1	-1	-1
5fthf	5-Formyltetrahydrofolate	L(-)-5-Formyl-5,6,7,8-tetrahydrofolic acid/Folinic acid	C20H23N7O7	-2	-2	-2
5hiaa	5-Hydroxyindoleacetaldehyde		C10H9NO2	0	0	0
5hiac	5-Hydroxyindoleacetate		C10H9NO3	-1	-1	-1
5hknm	5-Hydroxykynurenamine		C9H12N2O2	1	1	1
5hknn	5-Hydroxykynurenine		C10H12N2O4	0	0	0
5hnfkn	5-Hydroxy-N-formylkynurenine		C11H12N2O5	0	0	0
5m3o4hcoa	5-Methyl-3-oxo-4-hexenoyl-CoA		C28H44N7O18P3S	-3	-4	-4
5mc	5-Methylcytosine		C5H7N3O	0	0	0

5mh4ecoa	5-Methylhex-4-enoyl-CoA		C28H46N7O17P3S	-3	-4	-4
5mta	5'-Methylthioadenosine	Methylthioadenosine/S-Methyl-5'-thioadenosine/5-Methylthioadenosine/5'-Deoxy-5'-(methylthio)adenosine/Thiomethyladenosine/MTA	C11H15N5O3S	0	0	0
5mtglu	5-Methyltetrahydropteroyltri-L-glutamate		C30H39N9O12	-4	-4	-4
5prdmbz	N1-(5-Phospho-alpha-D-ribosyl)-5,6-dimethylbenzimidazole	alpha-Ribazole 5'-phosphate	C14H19N2O7P	-1	-2	-2
6checoa	6-Carboxyhex-2-enoyl-CoA	2,3-Didehydro-pimeloyl-CoA	C28H44N7O19P3S	-4	-5	-5
6chq	6-Chlorohydroxyquinol	6-Chlorobenzene-1,2,4-triol	C6H5ClO3	0	0	0
6fchdc	6-Fluorocyclohexadiene-cis,cis-1,2-diol-1-carboxylate		C7H7FO4	-1	-1	-1
6hcecoa	6-Hydroxycyclohex-1-enecarbonyl-CoA	6-Hydroxycyclohex-1-ene-1-carboxyl-CoA	C28H44N7O18P3S	-3	-4	-4
6hhn	6-Hydroxyhexanoic acid	6-Hydroxyhexanoate	C6H12O3	-1	-1	-1
6hidlac	6-Hydroxyindolelactate		C11H11NO4	-1	-1	-1
6hnld	6-Hexanolide	1-Oxa-2-oxocycloheptane/epsilon-Caprolactone/2-Oxepanone/hexano-6-lactone	C6H10O2	0	0	0
6pgl	6-phospho-D-glucono-1,5-lactone	D-Glucono-1,5-lactone 6-phosphate	C6H11O9P	-1	-2	-2
7m3o6ocoa	7-Methyl-3-oxo-6-octenoyl-CoA		C30H48N7O18P3S	-3	-4	-4
a4ic	5-Amino-4-imidazole carboxylate	4-Amino-5-imidazolecarboxylic acid	C4H5N3O2	0	0	-1
a5p	D-Arabinose 5-phosphate		C5H11O8P	-1	-2	-2
a6rp	4-(1-D-Ribitylamino)-5-aminouracil	4-(1-D-Ribitylamino)-5-amino-2,6-dihydroxypyrimidine	C9H16N4O6	0	0	0
a6rp5p	5-Amino-6-(5'-phosphoribosylamino)uracil	5-Amino-6-(ribosylamino)-2,4-(1H,3H)-pyrimidinedione 5'-phosphate/5-Amino-6-(5-phosphoribosylamino)uracil	C9H15N4O9P	-1	-2	-2
a6rp5p2	5-Amino-6-(5'-phosphoribitylamino)uracil	5-Amino-2,6-dioxy-4-(5'-phosphoribitylamino)pyrimidine/5-Amino-6-(5-phosphoribitylamino)uracil	C9H17N4O9P	-1	-2	-2
aacald	Aminoacetaldehyde		C2H5NO	1	1	0
aacoa	Acetoacetyl-CoA	Acetoacetyl coenzyme A/3-Acetoacetyl-CoA	C25H40N7O18P3S	-3	-4	-4
aact	Aminoacetone	1-Amino-2-propanone	C3H7NO	1	1	0
aagcya	alpha-Amino-gamma-cyanobutanoate	2-Amino-4-cyanobutanoic acid	C5H8N2O2	0	0	0
aamppn	alpha-Aminopropiononitrile		C3H6N2	1	0	0
abut	(S)-2-Aceto-2-hydroxybutanoate	(S)-2-Hydroxy-2-ethyl-3-oxobutanoate	C6H10O4	-1	-1	-1
ac	Acetate	Acetic acid/Ethanoic acid/Glacial acetic acid	C2H4O2	-1	-1	-1
acac	Acetoacetate	3-Oxobutanoic acid/beta-Ketobutyric acid/Acetoacetic acid	C4H6O3	-1	-1	-1
acACP	Acetyl-ACP		C13H23N2O8PRS	-1	-1	-1
acal	Acetaldehyde	Ethanal	C2H4O	0	0	0
acala	N-Acetylmuramoyl-Ala	N-Acetyl-D-muramoyl-L-alanine	C14H24N2O9	-1	-1	-1
accoa	Acetyl-CoA	Acetyl coenzyme A	C23H38N7O17P3S	-3	-4	-4
acda	Adenosyl cobyrinate a,c diamide	Adenosyl cobyrinate diamide/Adenosylcob(Ⅲ)yrinic acid a,c-diamide/Adenosylcobyrinic acid a,c-diamide	C55H73CoN11O15	-5	-5	-5
acetol	Acetol	Hydroxyacetone/1-Hydroxy-2-propanone/2-Ketopropyl alcohol/Acetone alcohol/Pyruvinalcohol/Pyruvic alcohol/Methylketol	C3H6O2	0	0	0
acg5p	N-Acetyl-L-glutamyl 5-phosphate	N-Acetyl-L-glutamate 5-phosphate	C7H12NO8P	-3	-3	-3
acha	Adenosyl cobyrinate hexaamide	Adenosylcobyric acid	C55H77CoN15O11	0	0	0
aclm	Acrylamide	2-Propenamide	C3H5NO	0	0	0
acmur	N-Acetyl-D-muramoate		C11H19NO8	-1	-1	-1
acon-C	cis-Aconitate	cis-Aconitic acid	C6H6O6	-3	-3	-3
aconit	Acrylonitrile	Propenenitrile/Vinyl cyanide	C3H3N	0	0	0
ACP	Acyl-carrier protein	ACP/[Acyl-carrier protein]/Holo-[acyl-carrie-protein]	HSR	-1	-1	-1
acpc	1-Aminocyclopropane-1-carboxylate	1-Aminocyclopropane-1-carboxylic acid	C4H7NO2	0	0	0
acputs	N-Acetylputrescine		C6H14N2O	1	1	1
actACP	Acetoacetyl-[acp]	Acetoacetyl-[acyl-carrier protein]	C4H5O2SR	-1	-1	-1
actp	Acetyl phosphate		C2H5O5P	-2	-2	-2
ad	Adenine	6-Aminopurine	C5H5N5	0	0	0
adcba	Adenosyl cobinamide		C58H84CoN16O11	0	0	0
adcbap	Adenosyl cobinamide phosphate		C58H85CoN16O14P	-1	-2	-2
adchor	4-amino-4-deoxychorismate	ADC	C10H11NO5	-1	-1	-1
adip	Adipate	Hexanedioate/Hexan-1,6-dicarboxylate	C6H10O4	-2	-2	-2
adipcoa	Adipyl-CoA	5-Carboxypentanoyl-CoA	C27H44N7O19P3S	-4	-5	-5
adlipo	S-Acetyldihydrolipoamide-E	[Dihydrolipoyllysine-residue acetyltransferase] S-acetyldihydrolipoyllysine	C10H18NO2S2R	0	0	0
adn	Adenosine		C10H13N5O4	0	0	0

adocbl	Adenosylcobalamin	Cobamide coenzyme/Deoxyadenosylcobalamin/Cobamamide/Vitamin B12 coenzyme/5,6-Dimethylbenzimidazolyl-5-deoxyadenosyl-cobamide/(5'-Deoxy-5'-adenosyl)cobamide coenzyme/(5,6-Dimethylbenzimidazolyl)cobamide coenzyme/alpha-(5,6-Dimethylbenzimidazolyl)cobamide coenzyme/5'-Deoxy-5'-adenosylcobalamin/5'-Deoxy-5'-adenosyl vitamin B12/5'-Deoxy-5'-adenosyl-5,6-dimethylbenzimidazolylcobamide/5,6-Dimethylbenzimidazolyl-Co-5'-deoxy-5'-adenosylcobamide/Calomide/Cobalamin coenzyme/Coenzyme B12/DMBC coenzyme/Dibencozide/Funacomide	C72H100CoN18O17P	0	-1	-1
adp	ADP	Adenosine 5'-diphosphate	C10H15N5O10P2	-2	-2	-3
adpdgdmhep	ADP-D-glycero-D-manno-heptose		C17H27N5O16P2	-2	-2	-2
adphep	ADP-L-glycero-D-manno-heptose		C17H27N5O16P2	-2	-2	-2
adprib	ADP-D-ribose		C15H23N5O14P2	-2	-2	-2
adrn	L-Adrenaline	(R)-(-)-Adrenaline/(R)-(-)-Epinephrine/(R)-(-)-Epirenamine/(R)-(-)-Adnephrine/4-[(1R)-1-Hydroxy-2-(methylamino)ethyl]-1,2-benzenediol	C9H13NO3	1	1	1
ag	L-Arogenate	L-Arogenic acid/Pretyrosine	C10H13NO5	-1	-1	-1
agdpcba	Adenosine-GDP-cobinamide	Adenosylcobinamide-GDP	C68H97CoN21O21P2	-2	-2	-2
agl	1-Acylglycerol	Glyceride/Monoglyceride/Monoacylglycerol/1-Monoacylglycerol	C4H7O4R	0	0	0
agl3p	1-Acyl-sn-glycerol 3-phosphate		C4H8O7PR	-1	-2	-2
agmatine	Agmatine	(4-Aminobutyl) guanidine	C5H14N4	2	2	2
ahdt	2-Amino-4-hydroxy-6-(erythro-1,2,3-trihydroxypropyl)dihydropteridine triphosphate	6-(L-erythro-1,2-Dihydroxypropyl 3-triphosphate)-7,8-dihydropterin/6-[(1S,2R)-1,2-Dihydroxy-3-triphosphooxypropyl]-7,8-dihydropterin	C9H16N5O13P3	-3	-3	-4
ahhmd	2-Amino-7,8-dihydro-4-hydroxy-6-(diphosphooxymethyl)pteridine	2-Amino-4-hydroxy-6-hydroxymethyl-7,8-dihydropteridine diphosphate/7,8-Dihydropterin pyrophosphate	C7H11N5O8P2	-2	-2	-3
ahhmp	2-Amino-4-hydroxy-6-hydroxymethyl-7,8-dihydropteridine		C7H9N5O2	0	0	0
ahm	4-Amino-5-hydroxymethyl-2-methylpyrimidine	Toxopyrimidine/4-Amino-2-methyl-5-pyrimidinemethanol	C6H9N3O	1	0	0
ahmpp	2-Methyl-4-amino-5-hydroxymethylpyrimidine diphosphate	4-Amino-2-methyl-5-diphosphomethylpyrimidine	C6H11N3O7P2	-1	-2	-3
ahser	O-Acetylhomoserine		C6H11NO4	0	0	0
aicar	1-(5'-Phosphoribosyl)-5-amino-4-imidazolecarboxamide	5'-Phosphoribosyl-5-amino-4-imidazolecarboxamide/5'-Phospho-ribosyl-5-amino-4-imidazole carboxamide/AICAR/5-Aminoimidazole-4-carboxamide ribotide/5-Phosphoribosyl-4-carbamoyl-5-aminoimidazole/5-Amino-1-(5-phospho-D-ribosyl)imidazole-4-carboxamide	C9H15N4O8P	-1	-2	-2
air	Aminoimidazole ribotide	AIR/1-(5'-Phosphoribosyl)-5-aminoimidazole/5'-Phosphoribosyl-5-aminoimidazole/1-(5-Phospho-D-ribosyl)-5-aminoimidazole/5-Amino-1-(5-phospho-D-ribosyl)imidazole	C8H14N3O7P	0	-1	-2
akg	2-Oxoglutarate	Oxoglutaric acid/2-Ketoglutaric acid/alpha-Ketoglutaric acid	C5H6O5	-2	-2	-2
ala	L-Alanine	L-2-Aminopropionic acid/L-alpha-Alanine	C3H7NO2	0	0	0
alaala	D-Alanyl-D-alanine	D-Ala-D-Ala	C6H12N2O3	0	0	-1
alac-S	(S)-2-Acetolactate	(S)-2-Hydroxy-2-methyl-3-oxobutanoate	C5H8O4	-1	-1	-1
alatrna	L-Alanyl-tRNA	L-Alanyl-tRNA(Ala)	C13H22NO11PR2(C5H8O6PR)n	1	1	1
alltn	Allantoin	5-Ureidohydantoin/Glyoxyldiureide	C4H6N4O3	0	0	-1
alltt	Allantoate	Allantoic acid	C4H8N4O4	-1	-1	-1
amdz	5-Aminoimidazole	Aminoimidazole/4-Aminoimidazole	C3H5N3	1	1	1
ammld	Ammelide	2,4-Dihydroxy-6-amino-1,3,5-triazine	C3H4N4O2	0	0	0
amp	AMP	Adenosine 5'-monophosphate/Adenylic acid/Adenylate/5'-AMP/5'-Adenylic acid/5'-Adenosine monophosphate/Adenosine 5'-phosphate	C10H14N5O7P	-1	-2	-2
amphebut	4-(2-Aminophenyl)-2,4-dioxobutanoate		C10H9NO4	-1	-1	-1
amppo	(R)-1-Aminopropan-2-ol	(R)-1-Amino-2-propanol	C3H9NO	1	1	1
an	Anthranilate	Anthranilic acid/o-Aminobenzoic acid/Vitamin L1/2-Aminobenzoate	C7H7NO2	-1	-1	-1
aniline	Aniline	Phenylamine/Benzenamine/Arylamine	C6H7N	0	0	0
anthcoa	Anthranilyl-CoA	2-Aminobenzoyl-CoA	C28H41N8O17P3S	-3	-4	-4
anthr910d	Anthracene-9,10-dihydrodiol		C14H12O2	0	0	0
anthrc	Anthracene		C14H10	0	0	0
ao4pob	2-Amino-3-oxo-4-phosphonooxybutyrate	L-2-Amino-3-oxo-4-phosphonooxybutyrate/(2S)-2-Amino-3-oxo-4-phosphonooxybutanoate	C4H8NO7P	-1	-2	-3

aobzac	alpha-Oxo-benzeneacetic acid	Benzoylformate/Benzoylformic acid/Phenylglyoxylic acid/Phenylglyoxylate/2-Oxo-2-phenylacetate	C8H6O3	-1	-1	-1
aona	8-Amino-7-oxononanoate	8-Amino-7-oxononanoic acid	C9H17NO3	0	0	0
apoACP	apoprotein [acyl carrier protein]		RHO	0	0	0
appppa	P1,P4-Bis(5'-adenosyl) tetraphosphate	AppppA	C20H28N10O19P4	-4	-4	-4
aps	Adenylyl sulfate	Adenosine 5'-phosphosulfate/APS/5'-Adenylyl sulfate	C10H14N5O10PS	-2	-2	-2
arbt	L-Arabitol	L-Arabinol/L-Arabinitol/L-Lyxitol	C5H12O5	0	0	0
arg	L-Arginine	(S)-2-Amino-5-guanidinovaleric acid	C6H14N4O2	1	1	1
argsucc	N-(L-Arginino)succinate	N(omega)-(L-Arginino)succinate/L-Argininosuccinate/L-Argininosuccinic acid/L-Arginosuccinic acid	C10H18N4O6	-1	-1	-1
argtrna	L-Arginyl-tRNA(Arg)	L-Arginyl-tRNA	C21H33N9O11PR(C5H8O6PR)n	2	2	2
ascb	L-Ascorbate	Ascorbate/Ascorbic acid/L-Ascorbic acid/Vitamin C	C6H8O6	-1	-1	-1
ascb6p	L-Ascorbate 6-phosphate		C6H9O9P	-2	-3	-3
aselnt	Adenylylselenate	Adenosine-5'-phosphoselenate	C10H14N5O10PSe	-1	-1	-1
aser	O-Acetyl-L-serine	O3-Acetyl-L-serine	C5H9NO4	0	0	0
asn	L-Asparagine	2-Aminosuccinamic acid	C4H8N2O3	0	0	0
asntrna	L-Asparaginyl-tRNA(Asn)	Asn-tRNA(Asn)/Asparaginyl-tRNA(Asn)	C14H23N2O12PR2(C5H8O6PR)n	0	0	0
aso3	arsenite		AsO3	0	-1	-1
asp	L-Aspartate	L-Aspartic acid/2-Aminosuccinic acid	C4H7NO4	-1	-1	-1
asp-D	D-Aspartate	D-Aspartic acid	C4H7NO4	-1	-1	-1
aspsa	L-Aspartate 4-semialdehyde	Aspartate beta-semialdehyde/L-Aspartic 4-semialdehyde	C4H7NO3	0	0	0
asptrna	L-Aspartyl-tRNA(Asp)		C14H22NO13PR2(C5H8O6PR)n	0	0	0
asuc	N6-(1,2-Dicarboxyethyl)-AMP	Adenylosuccinate/Adenylosuccinic acid	C14H18N5O11P	-3	-4	-4
athr	L-Allo-threonine	L-allo-Threonine	C4H9NO3	0	0	0
atp	ATP	Adenosine 5'-triphosphate	C10H16N5O13P3	-3	-3	-4
b5amp	Biotinyl-5'-AMP		C20H28N7O9PS	-1	-1	-1
bal	Betaine aldehyde		C5H12NO	1	1	1
bala	beta-Alanine	3-Aminopropionic acid/3-Aminopropanoate	C3H7NO2	0	0	0
bapa	beta-Aminopropion aldehyde		C3H7NO	1	1	1
basp	4-Phospho-L-aspartate	L-4-Aspartyl phosphate	C4H8NO7P	-2	-2	-2
bbtcoa	gamma-butyrobetainyl-CoA		C28H46N8O17P3S	-3	-4	-4
benzot	Benzoate	Benzoic acid/Benzenecarboxylic acid/Phenylformic acid/Dracylic acid	C7H6O2	-1	-1	-1
bf26p	beta-D-Fructose 2,6-bisphosphate	D-Fructose 2,6-bisphosphate	C6H14O12P2	-2	-4	-4
bf2p	beta-D-Fructose 2-phosphate	beta-D-Fructofuranose 2-phosphate	C6H13O9P	-1	-2	-2
bglycogen	branching glycogen		(C6H10O5)n	0	0	0
BIOMASS	biomass			0	0	0
bisphenol	Bisphenol A	2,2-Bis(4-Hydroxyphenyl)propane	C15H16O2	0	0	0
bp23d	Biphenyl-2,3-diol	2,3-Dihydroxybiphenyl/3-Phenylcatechol	C12H10O2	0	0	0
br	Bromide	Br-	Br	-1	-1	-1
bt	Biotin	D-Biotin/Vitamin H/Coenzyme R	C10H16N2O3S	-1	-1	-1
butanal	Butanal	Butyraldehyde	C4H8O	0	0	0
butn	Butanoic acid	Butanoate/Butyrate/Butyric acid	C4H8O2	-1	-1	-1
bzaccoa	Benzoyl acetyl-CoA	Benzoyl acetyl coenzyme A	C30H42N7O18P3S	-3	-4	-4
bzald	Benzaldehyde	Benzoic aldehyde	C7H6O	0	0	0
bzamid	Benzamide		C7H7NO	0	0	0
bzocoa	Benzoyl-CoA	S-Benzoate coenzyme A	C28H40N7O17P3S	-3	-4	-4
bzonit	Benzonitrile	Phenyl cyanide/Cyanobenzene	C7H5N	0	0	0
bzop	Benzoyl phosphate		C7H7O5P	-2	-2	-2
c040coa	Butanoyl-CoA	Butyryl-CoA	C25H42N7O17P3S	-3	-4	-4
c120	Dodecanoate	Dodecanoic acid/Dodecylcarboxylate/Lauric acid	C12H24O2	-1	-1	-1
c120ACP	Dodecanoyl-[acyl-carrier protein]	Dodecanoyl-[acp]/Lauroyl-[acyl-carrier protein]	C12H23OSR	-1	-1	-1
c140	Tetradecanoic acid	Tetradecanoate/Myristic acid	C14H28O2	-1	-1	-1
c140ACP	Myristoyl-[acyl-carrier protein]	Tetradecanoyl-[acp]/Tetradecanoyl-[acyl-carrier protein]	C14H27OSR	-1	-1	-1
c141	Myristoleic acid	(9Z)-Tetradecenoic acid/9-Tetradecenoic acid/(Z)-Tetradec-9-enoic acid	C14H26O2	-1	-1	-1
c141ACP	cis-tetradec-7-enoyl-[acyl-carrier protein] (n-C14:1)		C25H45N2O8PRS	-1	-1	-1
c150	pentadecanoic acid (C15:0)	Pentadecylic acid/n-Pentadecanoic acid	C15H30O2	-1	-1	-1
c150ACP	pentadecanoyl-ACP (C15:0 ACP)		C26H49N2O8PRS	-1	-1	-1
c151	pentadecenoic acid (C15:1)		C15H28O2	-1	-1	-1
c151ACP	pentadecenoyl-ACP (C15:1 ACP)		C26H47N2O8PRS	-1	-1	-1
c160	Hexadecanoate	Hexadecanoic acid/Hexadecylic acid/Palmitic acid/Palmitate/Cetylic acid	C16H32O2	-1	-1	-1
c160ACP	Palmitoyl-ACP (n-C16:0ACP)	Hexadecanoyl-[acp]/Hexadecanoyl-[acyl-carrier protein]	C16H31OSR	-1	-1	-1
c161	hexadecenoate (n-C16:1)	(9Z)-Hexadecenoic acid/ cis-9-Hexadecenoic acid/ Palmitoleic acid	C16H30O2	-1	-1	-1
c161ACP	cis-hexadec-9-enoyl-[acyl-carrier protein] (n-C16:1)		C27H49N2O8PRS	-1	-1	-1
c170	heptadecanoic acid (C17:0)		C17H34O2	-1	-1	-1

c170ACP	heptadecanoyl-ACP (C17:0 ACP)		C28H53N2O8PRS		-1	-1	-1
c171	Heptadecenoic acid (C17:1)		C17H32O2		-1	-1	-1
c171ACP	heptadecenoyl-ACP (C17:1 ACP)		C28H51N2O8PRS		-1	-1	-1
c180	Octadecanoic acid	Stearate/Stearic acid	C18H36O2		-1	-1	-1
c180ACP	Octadecanoyl-ACP (n-C18:0ACP)		C29H55N2O8PRS		-1	-1	-1
c181	(9Z)-Octadecenoic acid	(Z)-Octadec-9-enoic acid/Oleate/Oleic acid	C18H34O2		-1	-1	-1
c181ACP	cis-octadec-11-enoyl-[acyl-carrier protein] (n-C18:1)		C29H53N2O8PRS		-1	-1	-1
c190	nonadecanoic acid (C19:0)		C19H38O2		-1	-1	-1
c190ACP	nonadecanoyl-ACP (C19:0 ACP)		C30H57N2O8PRS		-1	-1	-1
c23dhddt	cis-2,3-Dihydrodiol 1,1,1-Trichloro-2,2-bis(4'-chlorophenyl)ethane	cis-2,3-Dihydrodiol DDT	C14H11Cl5O2		0	0	0
c2ch4cmo	cis-2-Chloro-4-carboxymethylenebut-2-en-1,4-olide	cis-2-Chlorodienelactone	C6H3ClO4		-1	-1	-1
c34dhdhf	(+)-(3S,4R)-cis-3,4-Dihydroxy-3,4-dihydrofluorene	(+)-(1R,2S)-cis-3,4-Dihydroxy-3,4-dihyd	C13H12O2		0	0	0
c34dhphe	(+)-cis-3,4-Dihydrophenanthrene-3,4-diol	cis-3,4-Dihydroxy-3,4-dihydrophenanthrene	C14H12O2		0	0	0
c3chp	cis-3-Chloro-2-propene-1-ol		C3H5ClO		0	0	0
c3chroald	cis-3-Chloroallyl aldehyde		C3H3ClO		0	0	0
c3chroc	cis-3-Chloroacrylic acid		C3H3ClO2		-1	-1	-1
c4cmbo	cis-4-Carboxymethylenebut-2-en-4-olide	4-Carboxymethylenebut-2-en-4-olide	C6H4O4		-1	-1	-1
caasp	N-Carbamoyl-L-aspartate		C5H8N2O5		-2	-2	-2
cabm	Carbamate	Carbamic acid/Aminoformic acid	CH3NO2		-1	-1	-1
cacac	cis-Acetylacrylate		C5H6O3		-1	-1	-1
cair	1-(5-Phospho-D-ribosyl)-5-amino-4-imidazolecarboxylate	1-(5'-Phosphoribosyl)-5-amino-4-imidazolecarboxylate/1-(5'-Phosphoribosyl)-5-amino-4-carboxyimidazole/5'-Phosphoribosyl-5-amino-4-imidazolecarboxylate/1-(5'-Phosphoribosyl)-4-carboxy-5-aminoimidazole/5'-Phosphoribosyl-4-carboxy-5-aminoimidazole/5-Amino-1-(5-phospho-D-ribosyl)imidazole-4-carboxylate	C9H14N3O9P		-1	-2	-3
caiz	5-carboxyamino-1-(5-phospho-D-ribosyl)imidazole		C9H14N3O9P		-1	-2	-3
camp	cAMP	3',5'-Cyclic AMP/Cyclic adenylic acid/Cyclic AMP/Adenosine 3',5'-phosphate	C10H12N5O6P		-1	-1	-1
cap	Carbamoyl phosphate		CH4NO5P		-1	-2	-2
CARBO	carbohydrate			0	0	0	0
carbs	N-Carbamoylsarcosine		C4H8N2O3		-1	-1	-1
carccm	3-Carboxy-cis,cis-muconate	beta-Carboxy-cis,cis-muconate/cis,cis-Butadiene-1,2,4-tricarboxylate	C7H6O6		-3	-3	-3
carpcoa	5-Carboxy-2-pentenoyl-CoA		C27H42N7O19P3S		-4	-5	-5
carvnapc	3-(2-Carboxyvinyl)naphthalene-2-carboxylic acid		C14H10O4		-2	-2	-2
catech	Catechol	1,2-Benzenediol/o-Benzenediol/1,2-Dihydroxybenzene/Brenzcatechin/Pyrocatechol	C6H6O2		0	0	0
CAV	cofactors and vitamines			0	0	0	0
cbi	Cobinamide		C48H72CoN11O8		0	0	0
cbl1	Cob(I)alamin	Cbl/Vitamin B12s	C62H89CoN13O14P		0	-1	-1
cchc	Cyclohexane-1-carboxylate	Hexahydrobenzoic acid	C7H12O2		-1	-1	-1
cchccoa	Cyclohexane-1-carboxyl-CoA		C28H46N7O17P3S		-3	-4	-4
cchexo	Cyclohexanone		C6H10O		0	0	0
cchfor	N-Cyclohexylformamide		C7H13NO		0	0	0
cchlam	Cyclohexylamine	Cyclohexanamine	C6H13N		1	1	1
ccmuc	cis,cis-Muconate	cis,cis-Hexadienedioate/cis,cis-2,4-Hexadienedioic acid	C6H6O4		-2	-2	-2
ccoa	Crotonoyl-CoA	Crotonyl-CoA/2-Butenoyl-CoA/trans-But-2-enoyl-CoA/But-2-enoyl-CoA	C25H40N7O17P3S		-3	-4	-4
ccpam	N-Cyclopropylammeline	2-Hydroxy-4-amino-6-(cyclopropylamino)-1,3,5-triazine	C6H9N5O		0	0	0
ccpamd	N-Cyclopropylammelide	2,4-Dihydroxy-6-(cyclopropylamino)-1,3,5-triazine	C6H8N4O2		0	0	0
ccppam	Cyclopropylamine		C3H7N		1	1	1
ccppc	Cyclopropanecarboxylate		C4H6O2		-1	-1	-1
ccppccoa	Cyclopropanecarboxyl-CoA		C25H40N7O17P3S		-3	-4	-4
cddh	2-Carboxy-2,3-dihydro-5,6-dihydroxyindole	Leucodopachrome	C8H7NO2		-1	-1	-1
cdp	CDP	Cytidine 5'-diphosphate/Cytidine diphosphate	C9H15N3O11P2		-2	-2	-3
cdpddglc	CDP-4-dehydro-6-deoxy-D-glucose		C15H23N3O15P2		-2	-2	-2
cdpdg	CDP-diacylglycerol	CDP-1,2-diacylglycerol/1,2-Diacyl-sn-glycero-3-cytidine-5'-diphosphate	C14H19N3O15P2R2		-2	-2	-2
cdpetn	CDP-ethanolamine		C11H20N4O11P2		-1	-1	-1
cdpglc	CDP-glucose	CDP-D-Glucose	C15H25N3O16P2		-2	-2	-2
cdpmde	4-(Cytidine 5'-diphospho)-2-C-methyl-D-erythritol		C14H25N3O14P2		-2	-2	-2
cechddd	cis-3-(Carboxy-ethyl)-3,5-cyclo-hexadiene-1,2-diol	cis-3-(2-Carboxy-ethyl)-3,5-cyclo-hexadiene-1,2-diol	C9H12O4		-1	-1	-1

cenchddd	cis-3-(3-Carboxyethenyl)-3,5-cyclohexadiene-1,2-diol		C9H10O4	-1	-1	-1
cgmp	3',5'-Cyclic GMP	Guanosine 3',5'-cyclic monophosphate/Guanosine 3',5'-cyclic phosphate/Cyclic GMP/cGMP	C10H12N5O7P	-1	-1	-1
chac	Chloroacetic acid	Chloroethanoic acid	C2H3ClO2	-1	-1	-1
chacald	Chloroacetaldehyde	2-Chloroethanal	C2H3ClO	0	0	0
chcatol	3-Chlorocatechol		C6H5ClO2	0	0	0
chccm	2-Chloro-cis,cis-muconate		C6H5ClO4	-2	-2	-2
chhmsald	3-Chloro-2-hydroxymuconic semialdehyde		C6H5ClO4	-1	-1	-1
choline	Choline	Bilineurine	C5H14NO	1	1	1
cholp	Choline phosphate	Phosphorylcholine/Phosphocholine/O-Phosphocholine	C5H15NO4P	0	-1	-1
chor	chorismate	Chorismic acid	C10H10O6	-2	-2	-2
cinnm	trans-Cinnamate	trans-Cinnamic acid/(E)-Cinnamate	C9H8O2	-1	-1	-1
cit	Citrate	Citric acid/2-Hydroxy-1,2,3-propanetricarboxylic acid/2-Hydroxytricarballylic acid	C6H8O7	-3	-3	-3
citnl	Citronellate	Citronellic acid/3,7-Dimethyl-6-octenoic acid	C10H18O2	-1	-1	-1
citnlcoa	Citronellyl-CoA		C31H52N7O17P3S	-3	-4	-4
citr	L-Citrulline	2-Amino-5-ureidovaleric acid/Citrulline	C6H13N3O3	0	0	0
ckdo	CMP-3-deoxy-D-manno-octulosonate	CMP-KDO	C17H26N3O15P	-2	-2	-2
cl	Chloride		Cl	-1	-1	-1
clpn	cardiolipin	Cardiolipin/Diphosphatidylglycerol/1',3'-Bis(1,2-diacyl-sn-glycero-3-phospho)-sn-glycerol	C13H18O17P2R4	-2	-2	-2
cmcdopp	3-[6-(Carboxymethylene)cyclohexa-2,4-dien-1-ylidene]-2-oxopropanate		C11H8O5	-2	-2	-2
cmp	CMP	Cytidine-5'-monophosphate/Cytidylic acid	C9H14N3O8P	-1	-2	-2
cn	Cyanide	Prussiate/CN-/Cyano	CN	0	0	0
co2	CO2	Carbon dioxide	CO2	0	0	0
coa	Coenzyme A	CoA/CoA-SH	C21H36N7O16P3S	-3	-4	-4
coadip	2-Chloro-3-oxoadipate		C6H7ClO5	-2	-2	-2
cobacd	Cob(I)yrinate a,c diamide	Cob(I)yrinate diamide/Cob(I)yrinic acid a,c-diamide	C45H61CoN6O12	-5	-5	-5
cobalt2	Co2+	Cobalt	Co	2	2	2
cobtpc	Cobalt-precorrin 2	Cobalt-sirohydrochlorin	C42H46CoN4O16	-8	-8	-8
cpp	Coproporphyrinogen III		C36H44N4O8	-4	-4	-4
cppi	Coproporphyrinogen I		C36H44N4O8	-4	-4	-4
creatine	Creatine	alpha-Methylguanidino acetic acid/Methylglycocyamine	C4H9N3O2	0	0	0
cretn	Creatinine	1-Methylglycocyamidine	C4H7N3O	1	0	0
crn	L-Carnitine	L-gamma-Trimethyl-beta-hydroxybutyrobetaine/Vitamin BT/3-Carboxy-2-hydroxy-N,N,N-trimethyl-1-propanaminium hydroxide, inner salt/Levocarnitine/(R)-Carnitine	C7H15NO3	0	0	0
crncoa	L-Carnitinyl-CoA		C28H46N8O18P3S	-3	-4	-4
ct	Cytosine		C4H5N3O	0	0	0
ctbt	4-(Trimethylammonio)but-2-enoate		C7H14NO2	0	0	0
ctbtcoa	crotonobetainyl-CoA		C28H44N8O17P3S	-3	-4	-4
ctp	CTP	Cytidine 5'-triphosphate/Cytidine triphosphate	C9H16N3O14P3	-3	-3	-4
cvn	Cinnavalininate		C14H8N2O6	-2	-2	-2
cyaala	3-Cyano-L-alanine	L-3-Cyanoalanine/L-beta-Cyanoalanine	C4H6N2O2	0	0	-1
cyanr	Cyanuric acid		C3H3N3O3	0	0	0
cynt	Cyanate	Cyanic acid	CHNO	-1	-1	-1
cyromz	Cyromazine	2-Cyclopropylamino-4,6-diamino-s-triazine/Cyclopropylmelamine	C6H10N6	1	0	0
cys	L-Cysteine	L-2-Amino-3-mercaptopropionic acid	C3H7NO2S	0	0	0
cysgly	Cys-Gly	L-Cysteinylglycine	C5H10N2O3S	0	0	-1
cyst	L-Cystine	L-Dicysteine/L-alpha-Diamino-beta-dithiolactic acid	C6H12N2O4S2	0	0	0
cysteate	L-Cysteate	L-Cysteic acid/3-Sulfoalanine/2-Amino-3-sulfopropionic acid	C3H7NO5S	-1	-1	-1
cystrna	L-Cysteinyl-tRNA(Cys)		C18H26N6O11PSR(C5H8O6PR)n	1	1	1
cytd	Cytidine		C9H13N3O5	0	0	0
d1ap2oop	D-1-Aminopropan-2-ol O-phosphate	(R)-1-Aminopropan-2-yl phosphate	C3H10NO4P	0	-1	-1
d3dg	2-Dehydro-3-deoxy-D-glucarate		C6H8O7	-2	-2	-2
d4dg	5-Dehydro-4-deoxy-D-glucarate		C6H8O7	-2	-2	-2
d6pgc	6-Phospho-D-gluconate		C6H13O10P	-2	-3	-3
da	Deoxyadenosine	2'-Deoxyadenosine	C10H13N5O3	0	0	0
da-5	5'-Deoxyadenosine		C10H13N5O3	0	0	0
dadp	dADP	2'-Deoxyadenosine 5'-diphosphate	C10H15N5O9P2	-2	-2	-3
dala	D-Alanine	D-2-Aminopropionic acid/D-Ala	C3H7NO2	0	0	0
dalt	D-Altronate		C6H12O7	-1	-1	-1
damp	dAMP	2'-Deoxyadenosine 5'-phosphate/2'-Deoxyadenosine 5'-monophosphate/Deoxyadenylic acid/Deoxyadenosine monophosphate	C10H14N5O6P	-1	-2	-2
danna	7,8-Diaminononanoate		C9H20N2O2	1	1	0

datp	dATP	2'-Deoxyadenosine 5'-triphosphate/Deoxyadenosine 5'-triphosphate/Deoxyadenosine triphosphate	C10H16N5O12P3	-3	-3	-4
db4p	3,4-dihydroxy-2-butanone 4-phosphate		C4H9O6P	-1	-2	-2
dc	Deoxycytidine	2'-Deoxycytidine	C9H13N3O4	0	0	0
dcdp	dCDP	2'-Deoxycytidine diphosphate/2'-Deoxycytidine 5'-diphosphate	C9H15N3O10P2	-2	-2	-3
dchdchche	1,1-Dichloro-2-(dihydroxy-4'-chlorophenyl)-2-(4'-chlorophenyl)ethylene		C14H8Cl4O2	0	0	-1
dchrocmo	2,5-Dichloro-carboxymethylenebut-2-en-4-olide		C6H2Cl2O4	-1	-1	-1
dchrooe	2,5-Dichloro-4-oxohex-2-enedioate		C6H4Cl2O5	-2	-2	-2
dcmp	dCMP	Deoxycytidylic acid/Deoxycytidine monophosphate/Deoxycytidylate/2'-Deoxycytidine 5'-monophosphate	C9H14N3O7P	-1	-2	-2
dctp	dCTP	Deoxycytidine 5'-triphosphate/Deoxycytidine triphosphate/2'-Deoxycytidine 5'-triphosphate	C9H16N3O13P3	-3	-3	-4
dda	Bis(4'-chlorophenyl)acetate	DDA	C14H10Cl2O2	-1	-1	-1
ddm	Bis(4'-chlorophenyl)methane	DDM	C13H10Cl2	0	0	0
dg	Deoxyguanosine	2'-Deoxyguanosine	C10H13N5O4	0	0	0
dgal	D-Galactarate	D-Mucic acid/D-Galactaric acid	C6H10O8	-2	-2	-2
dgalac	D-Galacturonate	D-Galacturonic acid	C6H10O7	-1	-1	-1
dgalctn	D-Galactonate	D-Galactonic acid	C6H12O7	-1	-1	-1
dgdmh17bp	D-Glycero-D-manno-heptose 1,7-bisphosphate		C7H16O13P2	-2	-4	-4
dgdmh1p	D-Glycero-D-manno-heptose 1-phosphate		C7H15O10P	-1	-2	-2
dgdmh7p	D-Glycero-D-manno-heptose 7-phosphate		C7H15O10P	-1	-2	-2
dgdp	dGDP	2'-Deoxyguanosine 5'-diphosphate	C10H15N5O10P2	-2	-2	-3
dgln	D-Glutamine	D-2-Aminoglutaramic acid	C5H10N2O3	0	0	0
dglu	D-Glutamate	D-Glutamic acid/D-Glutaminic acid/D-2-Aminoglutaric acid	C5H9NO4	-1	-1	-1
dgluc	D-Glucuronate	Glucuronic acid/Glucuronate	C6H10O7	-1	-1	-1
dgluca	D-Glucarate	D-Glucaric acid/L-Gularic acid/d-Saccharic acid/D-Glucosaccharic acid	C6H10O8	-2	-2	-2
dglucl	D-Glucuronolactone	Glucurone/Glucofuranurono-6,3-lactone/D-Glucurono-3,6-lactone/D-Glucurone	C6H8O6	0	0	0
dgmp	dGMP	2'-Deoxyguanosine 5'-monophosphate/2'-Deoxyguanosine 5'-phosphate/Deoxyguanylic acid/Deoxyguanosine monophosphate	C10H14N5O7P	-1	-2	-2
dgr	1,2-Diacyl-sn-glycerol	1,2-Diacylglycerol/D-1,2-Diacylglycerol	C5H6O5R2	0	0	0
dgtp	dGTP	2'-Deoxyguanosine 5'-triphosphate/Deoxyguanosine 5'-triphosphate/Deoxyguanosine triphosphate	C10H16N5O13P3	-3	-3	-4
dhap	Glycerone phosphate	Dihydroxyacetone phosphate	C3H7O6P	-1	-2	-2
dhbpt	Dihydrobiopterin	6,7-Dihydrobiopterin/Quinoid-dihydrobiopterin/(6R)-6-(L-erythro-1,2-Dihydroxypropyl)-7,8-dihydro-6H-pterin	C9H13N5O3	0	0	0
dhchph	2,3-Dihydroxy-4'-chlorobiphenyl		C12H9ClO2	0	0	0
dhcinnm	trans-2,3-Dihydroxycinnamate		C9H8O4	-1	-1	-1
dhdp	2,3-Dihydrodipicolinate	L-2,3-Dihydrodipicolinate/Dihydrodipicolinic acid/Dihydrodipicolinate	C7H7NO4	-2	-2	-2
dhf	Dihydrofolate	Dihydrofolic acid/7,8-Dihydrofolate/7,8-Dihydrofolic acid/7,8-Dihydropteroylglutamate	C19H21N7O6	-2	-2	-2
dhhedd	2,4-Dihydroxyhept-2-enedioate	2,4-Dihydroxyhept-2-1,7-dioate/2,4-Dihydroxyhept-2-enedioic acid	C7H10O6	-2	-2	-2
dhid	5,6-Dihydroxyindole	DHI	C8H7NO2	0	0	0
dhmp	(R)-2,3-Dihydroxy-3-methylpentanoate	(R)-2,3-Dihydroxy-3-methylvalerate/(2R,3R)-2,3-Dihydroxy-3-methylpentanoate	C6H12O4	-1	-1	-1
dhmva	(R)-2,3-Dihydroxy-3-methylbutanoate	(R)-2,3-Dihydroxy-isovalerate/(R)-2,3-Dihydroxy-isovaleric acid/(2R)-2,3-Dihydroxy-3-methylbutanoate	C5H10O4	-1	-1	-1
dhn	1,4-Dihydroxy-2-naphthoate		C11H8O4	-1	-1	-1
dhnapthsul	1,2-Dihydroxynaphthalene-6-sulfonate		C10H8O5S	-1	-1	-1
dhnpt	Dihydroneopterin	2-Amino-4-hydroxy-6-(D-erythro-1,2,3-trihydroxypropyl)-7,8-dihydropteridine	C9H13N5O4	0	0	0
dhpant	2-Dehydropantoate		C6H10O4	-1	-1	-1
dhpmp	Dihydroneopterin phosphate	2-Amino-4-hydroxy-6-(erythro-1,2,3-trihydroxypropyl)dihydropteridine phosphate	C9H14N5O7P	-1	-2	-2
dhpppn	3-(2,3-Dihydroxyphenyl)propanoate	2,3-Dihydroxyphenylpropanoate	C9H10O4	-1	-1	-1
dhpt	Dihydropteroate	7,8-Dihydropteroate	C14H14N6O3	-1	-1	-1
dhsk	3-Dehydroshikimate		C7H8O5	-1	-1	-1
dhtolen	2,3-Dihydroxytoluene	3-Methylcatechol	C7H8O2	0	0	0
didp	dIDP	2'-Deoxyinosine-5'-diphosphate/2'-Deoxyinosine 5'-diphosphate	C10H14N4O10P2	-2	-2	-3
dimgly	N,N-Dimethylglycine	Dimethylglycine	C4H9NO2	0	0	0

dimgp	D-erythro-1-(Imidazol-4-yl)glycerol 3-phosphate	D-erythro-Imidazole-glycerol 3-phosphate/D-erythro-Imidazole-glycerol phosphate	C6H11N2O6P		-1	-2	-2
din	Deoxyinosine		C10H12N4O4		0	0	0
ditp	dITP	2'-Deoxyinosine-5'-triphosphate/2'-Deoxyinosine 5'-triphosphate	C10H15N4O13P3		-3	-3	-4
dlald	D-Lactaldehyde	(R)-Lactaldehyde/D-2-Hydroxypropionaldehyde	C3H6O2		0	0	0
dlipo	Dihydrolipoamide-E	Enzyme N6-(dihydrolipoyl)lysine	C8H16NOS2R		0	0	0
dlipoe	Dihydrolipoamide	Dihydrothioctamide	C8H17NOS2		0	0	0
dlipop	Dihydrolipoylprotein	[Protein]-dihydrolipoyllysine	C8H16NOS2R		0	0	0
dmal	D-Malate	(R)-Malate/D-Malic acid	C4H6O5		-4	-5	-5
dmbzid	5,6-Dimethylbenzimidazole	Dimethylbenzimidazole	C9H10N2		1	0	0
dmet	D-Methionine	D-2-Amino-4-(methylthio)butyric acid	C5H11NO2S		0	0	0
dmi1p	Inositol 1-phosphate	myo-Inositol 1-phosphate/1D-myo-Inositol 1-phosphate/D-myo-Inositol 1-phosphate/1D-myo-Inositol 1-monophosphate	C6H13O9P		-1	-2	-2
dmlz	6,7-Dimethyl-8-(1-D-ribityl)lumazine		C13H18N4O6		0	-1	-1
dmpp	Dimethylallyl diphosphate	Prenyl diphosphate/2-Isopentenyl diphosphate/delta2-Isopentenyl diphosphate/delta-Prenyl diphosphate/DMAPP	C5H12O7P2		-2	-2	-3
DNA	DNA			0	0	0	0
dnad	Deamino-NAD+	Deamido-NAD+/Deamido-NAD	C21H27N6O15P2		-3	-3	-3
dopa	Dopamine	4-(2-Aminoethyl)-1,2-benzenediol/4-(2-Aminoethyl)benzene-1,2-diol/3,4-Dihydroxyphenethylamine/2-(3,4-Dihydroxyphenyl)ethylamine	C8H11NO2		1	1	1
doroa	(S)-Dihydroorotate	(S)-4,5-Dihydroorotate/L-Dihydroorotate/L-Dihydroorotic acid/Dihydro-L-orotic acid	C5H6N2O4		-1	-1	-1
dpcoa	Dephospho-CoA		C21H35N7O13P2S		-2	-2	-2
dpcr	L-Dopachrome	2-L-Carboxy-2,3-dihydroindole-5,6-quinone	C9H7NO4		-1	-1	-1
dphe	D-Phenylalanine	D-alpha-Amino-beta-phenylpropionic acid	C9H11NO2		0	0	0
dqt	3-Dehydroquinate	5-Dehydroquinate/3-Dehydroquinic acid/5-Dehydroquinic acid	C7H10O6		-1	-1	-1
dr1p	2-Deoxy-D-ribose 1-phosphate	2-Deoxy-alpha-D-ribose 1-phosphate	C5H11O7P		-1	-2	-2
dscl	dihydrosirohydrochlorin	Precorrin 2	C42H48N4O16		-7	-7	-7
dser	D-Serine		C3H7NO3		0	0	0
dtb	Dethiobiotin	Desthiobiotin	C10H18N2O3		-1	-1	-1
dtdp	dTDP	Deoxythymidine 5'-diphosphate	C10H16N2O11P2		-2	-2	-3
dtdp4d6dg	dTDP-4-dehydro-6-deoxy-alpha-D-glucose	dTDP-4-oxo-6-deoxy-alpha-D-glucose	C16H24N2O15P2		-2	-2	-2
dtdp4d6dm	dTDP-4-dehydro-6-deoxy-L-mannose	dTDP-4-oxo-6-deoxy-L-mannose/dTDP-4-oxo-L-rhamnose	C16H24N2O15P2		-2	-2	-2
dtdpgal	dTDP-galactose	dTDP-D-galactose	C16H26N2O16P2		-2	-2	-2
dtdpglu	dTDP-glucose	dTDP-D-glucose	C16H26N2O16P2		-2	-2	-2
dtdprmn	dTDP-L-rhamnose	dTDP-6-deoxy-L-mannose	C16H26N2O15P2		-2	-2	-2
dtmp	dTMP	Thymidine 5'-phosphate/Deoxythymidine 5'-phosphate/Thymidylic acid/5'-Thymidylic acid/Thymidine monophosphate/Deoxythymidylic acid/Thymidylate	C10H15N2O8P		-1	-2	-2
dttp	dTTP	Deoxythymidine triphosphate/Deoxythymidine 5'-triphosphate/TTP	C10H17N2O14P3		-3	-3	-4
du	Deoxyuridine	2-Deoxyuridine/2'-Deoxyuridine	C9H12N2O5		0	0	0
dudp	dUDP	2'-Deoxyuridine 5'-diphosphate	C9H14N2O11P2		-2	-2	-3
dump	dUMP	Deoxyuridylic acid/Deoxyuridine monophosphate/Deoxyuridine 5'-phosphate/2'-Deoxyuridine 5'-phosphate	C9H13N2O8P		-1	-2	-2
dutp	dUTP	2'-Deoxyuridine 5'-triphosphate	C9H15N2O14P3		-3	-3	-4
dx5p	1-Deoxy-D-xylulose 5-phosphate		C5H11O7P		-1	-2	-2
e3mm	D-erythro-3-Methylmalate		C5H8O5		-2	-2	-2
e4hglu	L-erythro-4-Hydroxyglutamate		C5H9NO5		-1	-1	-1
e4p	D-Erythrose 4-phosphate		C4H9O7P		-1	-2	-2
er4p	4-Phospho-D-erythronate	4-Phosphoerythronate	C4H9O8P		-2	-3	-3
eth	Ethanol	Ethyl alcohol/Methylcarbinol/Dehydrated ethanol	C2H6O		0	0	0
etha	Ethanolamine	Aminoethanol/2-Hydroxyethylamine	C2H7NO		1	1	1
ethap	Ethanolamine phosphate	O-Phosphorylethanolamine/Phosphoethanolamine/O-Phosphoethanolamine	C2H8NO4P		0	-1	-1
ethlam	Ethylamine		C2H7N		1	1	1
f1p	D-Fructose 1-phosphate		C6H13O9P		-1	-2	-2
f6p	D-Fructose 6-phosphate	D-Fructose 6-phosphoric acid/Neuberg ester	C6H13O9P		-1	-2	-2
fa	Formamide	Methanamide	CH3NO		0	0	0
fad	FAD	Flavin adenine dinucleotide	C27H33N9O15P2		-2	-3	-3
fadh2	FADH2		C27H35N9O15P2		-2	-2	-2
fdp	D-Fructose 1,6-bisphosphate		C6H14O12P2		-2	-4	-4
fe2	Fe2+	Fe(II)/Ferrous ion/Iron(2+)	Fe		2	2	2
fe3	Fe3+	Fe(III)/Ferric ion/Iron(3+)	Fe		3	3	3
fgam	N2-Formyl-N1-(5-phospho-D-ribosyl)glycinamide	5'-Phosphoribosyl-N-formylglycinamide/N-Formyl-GAR/N-Formylglycinamide ribonucleotide	C8H15N2O9P		-1	-2	-2
fl	Folate	Pteroylglutamic acid/Folic acid	C19H19N7O6		-2	-2	-2

flac	Fluoroacetate	Fluoroacetic acid/Cymonic acid/Gifblaar poison/HFA/UN 2642	C2H3FO2	-1	-1	-1
flald	Fluoroacetaldehyde		C2H3FO	0	0	0
fmettrna	N-Formylmethionyl-tRNA		C16H26NO12PSR2(C5H8O6PR)n	0	0	0
fmgt	S-Formylglutathione		C11H17N3O7S	-1	-1	-1
fmn	FMN	Riboflavin-5-phosphate/Flavin mononucleotide	C17H21N4O9P	-1	-3	-3
forant	Formylanthranilate	N-Formylanthranilate/2-(Formylamino)-benzoic acid	C8H7NO3	-1	-1	-1
forglu	N-Formyl-L-glutamate		C6H9NO5	-2	-2	-2
forkn	L-Formylkynurenine	N-Formyl-L-kynurenine/N-Formylkynurenine	C11H12N2O4	0	0	0
formate	Formate	Methanoic acid/Formic acid	CH2O2	-1	-1	-1
formt	N-Formyl-L-methionine		C6H11NO3S	-1	-1	-1
fpram	2-(Formamido)-N1-(5-phospho-D-ribosyl)acetamidine	2-(Formamido)-N1-(5'-phosphoribosyl)acetamidine/1-(5'-Phosphoribosyl)-N-formylglycinamidine/5'-Phosphoribosyl-N-formylglycinamidine/5'-Phosphoribosylformylglycinamidine	C8H16N3O8P	0	-1	-2
fprica	5-Formamido-1-(5-phospho-D-ribosyl)imidazole-4-carboxamide	1-(5'-Phosphoribosyl)-5-formamido-4-imidazolecarboxamide/5'-Phosphoribosyl-5-formamido-4-imidazolecarboxamide/5-Formamido-1-(5-phosphoribosyl)imidazole-4-carboxamide	C10H15N4O9P	-1	-2	-2
frdp	Farnesyl diphosphate	trans,trans-Farnesyl diphosphate/Farnesyl pyrophosphate/2-trans,6-trans-Farnesyl diphosphate	C15H28O7P2	-2	-2	-3
fru	D-Fructose	Levulose/Fruit sugar/D-arabino-Hexulose	C6H12O6	0	0	0
fsalac	3-Formylsalicylic acid	2-Hydroxyisophthalaldehydic acid	C8H6O4	-1	-1	-1
fthf	10-Formyltetrahydrofolate	10-Formyl-THF	C20H23N7O7	-2	-2	-2
fuc	L-Fucose	6-Deoxy-L-galactose	C6H12O5	0	0	0
fuc1p	L-Fucose 1-phosphate	6-Deoxy-L-galactose 1-phosphate/beta-L-Fucose 1-phosphate	C6H13O8P	-1	-2	-2
fum	Fumarate	Fumaric acid/trans-Butenedioic acid	C4H4O4	-2	-2	-2
fumpyr	3-Fumarylpyruvate		C7H6O6	-2	-2	-2
g14l	L-Gulono-1,4-lactone	L-Gulono-gamma-lactone/gamma-Gulonolactone/L-Gulonic acid gamma-lactone/L-Gulonolactone	C6H10O6	0	0	0
g15l	D-Glucono-1,5-lactone	Gluconic lactone/Gluconic acid lactone/1,5-Gluconolactone/delta-Gluconolactone/D-Gluconolactone/Gluconolactone/D-Aldonolactone	C6H10O6	0	0	0
g1p	D-Glucose 1-phosphate	alpha-D-Glucose 1-phosphate/Cori ester/D-Glucose alpha-1-phosphate	C6H13O9P	-1	-2	-2
g3p	D-Glyceraldehyde 3-phosphate	(2R)-2-Hydroxy-3-(phosphonooxy)-propanal	C3H7O6P	-1	-2	-2
g3pc	sn-Glycero-3-phosphocholine	Glycerophosphocholine	C8H21NO6P	0	0	0
g3pe	sn-Glycero-3-phosphoethanolamine	Glycerophosphoethanolamine	C5H14NO6P	0	0	0
g3pg	Glycerophosphoglycerol		C6H15O8P	-1	-1	-1
g3pi	sn-Glycero-3-phospho-1-inositol	1-(sn-glycero-3-Phospho)-1D-myo-inositol	C9H19O11P	-1	-1	-1
g3ps	Glycerophosphoserine		C6H13NO8P	-1	-1	-1
g6p	D-Glucose 6-phosphate	Glucose 6-phosphate/Robison ester	C6H13O9P	-1	-2	-2
ga1p	D-Glucosamine 1-phosphate	alpha-D-Glucosamine 1-phosphate	C6H14NO8P	0	-1	-2
ga6p	D-Glucosamine 6-phosphate	D-Glucosamine phosphate	C6H14NO8P	0	-1	-1
gaba	4-Aminobutanoate	4-Aminobutanoic acid/4-Aminobutyrate/4-Aminobutyric acid/gamma-Aminobutyric acid/4-Aminobutylate	C4H9NO2	0	0	0
gagcya	gamma-Amino-gamma-cyanobutanoate	4-Amino-4-cyanobutanoic acid	C5H8N2O2	0	-1	-1
gallate	Gallate	Gallic acid/3,4,5-Trihydroxybenzoic acid/3,4,5-Trihydroxybenzoate/Pyrogallol-5-carboxylic acid	C7H6O5	-1	-1	-1
galt	Galactitol	Dulcitol/Dulcose	C6H14O6	0	0	0
galt1p	Galactitol 1-phosphate	D-Galactitol 1-phosphate/L-Galactitol 6-phosphate	C6H15O9P	-1	-2	-2
gam	D-Glucosamine	Chitosamine/2-Amino-2-deoxy-D-glucose	C6H13NO5	1	1	1
gar	5'-Phosphoribosylglycinamide	GAR/N1-(5-Phospho-D-ribosyl)glycinamide/Glycinamide ribonucleotide	C7H15N2O8P	0	-1	-2
gbbtn	gamma-butyrobetaine		C7H15NO2	0	0	0
gcarmclc	gamma-Carboxymuconolactone	5-Carboxy-2,5-dihydro-2-oxofuran-5-acetate/4-Carboxymuconolactone/2-Carboxy-2,5-dihydro-5-oxofuran-2-acetate	C7H6O6	-2	-2	-2
gcys	gamma-L-Glutamyl-L-cysteine	L-gamma-Glutamylcysteine/5-L-Glutamyl-L-cysteine/gamma-Glutamylcysteine	C8H14N2O5S	-1	-1	-1
gdp	GDP	Guanosine 5'-diphosphate/Guanosine diphosphate	C10H15N5O11P2	-2	-2	-3
gdpddman	GDP-4-dehydro-6-deoxy-D-mannose	GDP-4-dehydro-6-deoxy-D-talose/GDP-4-oxo-6-deoxy-D-mannose/GDP-4-dehydro-D-rhamnose/GDP-4-keto-6-deoxy-D-mannose	C16H23N5O15P2	-2	-2	-2
gdpmann	GDP-D-mannose	GDP-mannose	C16H25N5O16P2	-2	-2	-2
gensa	2,5-Dihydroxybenzoate	Gentisic acid/Hydroquinonecarboxylic acid/Gentisate	C7H6O4	-1	-1	-1
gerana	Geranic acid	3,7-Dimethylocta-2,6-dienoate	C10H16O2	-1	-1	-1

ggbap	gamma-Glutamyl-beta-aminopropiononitrile	gamma-Glutamyl-3-aminopropiononitrile	C8H13N3O3	0	0	0
ggbcya	gamma-Glutamyl-beta-cyanoalanine		C9H13N3O5	-1	-1	-1
gl	Glycerol	Glycerin/1,2,3-Trihydroxypropane/1,2,3-Propanetriol	C3H8O3	0	0	0
gl1coa	Glutaconyl-1-CoA	4-Carboxybut-2-enoyl-CoA	C26H40N7O19P3S	-4	-5	-5
glac	D-Galactose		C6H12O6	0	0	0
glal	Glycolaldehyde	Hydroxyacetaldehyde	C2H4O2	0	0	0
glc	D-Glucose	Grape sugar/Dextrose	C6H12O6	0	0	0
gln	L-Glutamine	L-2-Aminoglutaramic acid	C5H10N2O3	0	0	0
glntrna	Glutaminyl-tRNA	L-Glutaminyl-tRNA(Gln)/Glutaminyl-tRNA(Gln)/Gln-tRNA(Gln)	C20H29N7O12PR(C5H8O6PR)n	1	1	1
glu	L-Glutamate	L-Glutamic acid/L-Glutaminic acid	C5H9NO4	-1	-1	-1
glu1sa	L-Glutamate 1-semialdehyde	(S)-4-Amino-5-oxopentanoate	C5H9NO3	0	0	0
glu5p	L-Glutamate 5-phosphate	L-Glutamyl 5-phosphate	C5H10NO7P	-2	-2	-2
gluc	D-Gluconate	D-Gluconic acid/D-gluco-Hexonic acid	C6H12O7	-1	-1	-1
glugsal	L-Glutamate 5-semialdehyde	L-Glutamate gamma-semialdehyde	C5H9NO3	0	0	0
glutcoa	Glutaryl-CoA		C26H42N7O19P3S	-4	-5	-5
glutrna	L-Glutamyl-tRNA(Glu)		C20H28N6O13PR(C5H8O6PR)n	0	0	0
glx	Glyoxylate	Glyoxalate/Glyoxylic acid	C2H2O3	-1	-1	-1
gly	Glycine	Aminoacetic acid/Gly	C2H5NO2	0	0	0
glyb	Glycine betaine	Betaine/Trimethylaminoacetate/N,N,N-Trimethylglycine/Trimethylammonioacetate	C5H11NO2	0	0	0
glyc3p	Glycerol 3-phosphate	sn-Glycerol 3-phosphate/Glycerophosphoric acid/sn-Gro-1-P	C3H9O6P	-1	-2	-2
glycogen	Glycogen		C24H42O21	0	0	0
glycolate	Glycolate	Glycolic acid/Hydroxyacetic acid	C2H4O3	-1	-1	-1
glyc-R	(R)-Glycerate	D-Glycerate/Glycerate/Glyceric acid	C3H6O4	-1	-1	-1
glyn	Glycerone	Dihydroxyacetone/1,3-Dihydroxyacetone/1,3-Dihydroxy-2-propanone/1,3-Dihydroxypropan-2-one	C3H6O3	0	0	0
glytrna	Glycyl-tRNA(Gly)		C12H20NO11PR2(C5H8O6PR)n	1	1	1
gmp	GMP	Guanosine 5'-phosphate/Guanosine monophosphate/Guanosine 5'-monophosphate/Guanylic acid	C10H14N5O8P	-1	-2	-2
gn	Guanine	2-Amino-6-hydroxypurine	C5H5N5O	0	0	0
gp4g	P1,P4-Bis(5'-guanosyl) tetraphosphate	GppppG/Bis(5'-guanosyl) tetraphosphate	C20H28N10O21P4	-4	-4	-4
gpp	Geranyl diphosphate		C10H20O7P2	-2	-2	-3
grxox	Thioredoxin disulfide	Oxidized thioredoxin/Thioredoxin sulfide	C10H12N4O4S2R4	0	0	0
grxrd	Thioredoxin	Reduced thioredoxin	C10H14N4O4S2R4	0	0	0
gsn	Guanosine		C10H13N5O5	0	0	0
gtah	Gentisate aldehyde		C7H6O3	0	0	0
gtp	GTP	Guanosine 5'-triphosphate	C10H16N5O14P3	-3	-3	-4
gtspmd	Glutathionylspermidine	N1-(gamma-L-Glutamyl-L-cysteinyl-glycyl)-spermidine	C17H34N6O5S	2	2	2
guadbut	4-Guanidinobutanamide		C5H12N4O	1	1	1
guadbutn	4-Guanidinobutanoate		C5H11N3O2	0	0	0
guln	L-Gulonate	L-Gulonic acid/Gulonate/Gulonic acid	C6H12O7	-1	-1	-1
h	H+		H	1	1	1
h2	H2		H2	0	0	0
h2o	H2O	Water	H2O	0	0	0
h2o2	H2O2	Hydrogen peroxide/Oxydol	H2O2	0	0	0
h2s	Hydrogen sulfide	Hydrogen-sulfide/H2S	H2S	0	-1	-1
h3op	2-Hydroxy-3-oxopropanoate	Tartronate semialdehyde	C3H4O4	-1	-1	-1
hadpcoa	(3S)-3-Hydroxyadipyl-CoA		C27H44N7O20P3S	-4	-5	-5
hatraz	Hydroxyatrazine	4-(Ethylamino)-2-hydroxy-6-(isopropylamino)-1,3,5-triazine	C8H15N5O	0	0	0
hbzal	3-Hydroxybenzyl alcohol	3-Hydroxybenzenemethanol	C7H8O2	0	0	0
hcchccoa	2-Hydroxycyclohexane-1-carboxyl-CoA		C28H46N7O18P3S	-3	-4	-4
hccho	2-Hydroxycyclohexan-1-one		C6H10O2	0	0	0
hchdn	2-Hydroxy-cis-hex-2,4-dienoate		C6H8O3	-1	-1	-1
hcl	hcl		HCl	-1	-1	-1
hco3	Bicarbonate	HCO3-/Hydrogencarbonate/Acid carbonate	HCO3	0	-1	-1
hcys	L-Homocysteine	L-2-Amino-4-mercaptobutyric acid	C4H9NO2S	0	0	0
hcyst	Homocystine	4,4'-Dithiobis(2-aminobutyric acid)	C8H16N2O4S2	0	0	0
hedc	2-Hydroxyethylenedicarboxylate	enol-Oxaloacetate/enol-Oxaloacetic acid/2-Hydroxybut-2-enedioic acid	C4H4O5	-2	-2	-2
hemeA	Heme A		C49H56FeN4O6	0	0	0
hemeO	Heme O		C49H58FeN4O5	0	0	0
heppp	all-trans-Heptaprenyl diphosphate		C35H60O7P2	-2	-2	-3
hf	Hydrofluoric acid		HF	-1	-1	-1
hgcoa	2-Hydroxyglutaryl-CoA		C26H42N7O20P3S	-4	-5	-5
hibut	(S)-3-Hydroxyisobutyrate		C4H8O3	-1	-1	-1
his	L-Histidine	(S)-alpha-Amino-1H-imidazole-4-propionic acid	C6H9N3O2	1	0	0
hisol	L-Histidinol		C6H11N3O	2	1	1
hisolp	L-Histidinol phosphate		C6H12N3O4P	1	-1	-1

histrna	L-Histidyl-tRNA(His)		C16H24N3O11PR2(C5H8O6PR)n	1	1	1
hkhdn	2-Hydroxy-6-keto-2,4-heptadienoate	2-Hydroxy-6-oxo-hept-2,4-dienoate	C7H8O4	-1	-1	-1
hkn	3-Hydroxykynurenamine		C9H12N2O2	1	1	1
hlk	3-Hydroxy-L-kynurenine		C10H12N2O4	0	0	0
hmb	Hydroxymethylbilane		C40H46N4O17	-8	-8	-8
hmb4pp	1-Hydroxy-2-methyl-2-butenyl 4-diphosphate	(E)-4-Hydroxy-3-methylbut-2-en-1-yl diphosphate	C5H12O8P2	-2	-2	-3
hmcmct	2-Hydroxy-5-methyl-cis,cis-muconate		C7H8O5	-2	-2	-2
hmcmsald	2-Hydroxy-5-methyl-cis,cis-muconic semialdehyde	(2E,4Z)-2-Hydroxy-5-methyl-6-oxohexa-2,4-dienoate	C7H8O4	-1	-1	-1
hmgth	S-(Hydroxymethyl)glutathione		C11H19N3O7S	-1	-1	-1
hmnapth	1-Hydroxymethylnaphthalene	1-Naphthalenemethanol	C11H10O	0	0	0
hnapoxen	4-(3-Hydroxy-2-naphthyl)-2-oxobut-3-enoic acid		C14H10O4	-1	-1	-1
hnaptho	1-Hydroxy-2-naphthoate	1-Hydroxy-2-naphthoic acid/1-Naphthol-2-carboxylic acid	C11H8O3	-1	-1	-1
hochphhd	2-Hydroxy-6-oxo-6-(4'-chlorophenyl)-hexa-2,4-dienoate		C12H9ClO4	-1	-1	-1
hodhiyb2e	2-Hydroxy-4-(2-oxo-1,3-dihydro-2H-inden-1-ylidene)but-2-enoic acid		C13H10O4	-1	-1	-1
hodhiybe	2-Hydroxy-4-(1-oxo-1,3-dihydro-2H-inden-2-ylidene)-but-2-enoic acid		C13H10O4	-1	-1	-1
hohx	4-Hydroxy-2-oxohexanoic acid	4-Hydroxy-2-oxohexanoate	C6H10O4	-1	-1	-1
homogen	Homogentisate	Homogentisic acid/2,5-Dihydroxyphenylacetic acid/2,5-Dihydroxyphenylacetate	C8H8O4	-1	-1	-1
hophd	2-Hydroxy-6-oxo-6-phenylhexa-2,4-dienoate	2,6-Dioxo-6-phenylhexa-3-enoate	C12H10O4	-1	-1	-1
hopt	4-Hydroxy-2-oxopentanoate	4-Hydroxy-2-oxovalerate	C5H8O4	-1	-1	-1
hpheac	3-Hydroxyphenylacetate		C8H8O3	-1	-1	-1
hpimcoa	3-Hydroxypimeloyl-CoA	3-Hydroxypimelyl-CoA	C28H46N7O20P3S	-4	-5	-5
hpmhmbq	2-Hexaprenyl-3-methyl-5-hydroxy-6-methoxy-1,4-benzoquinone		C38H56O4	-1	-1	-1
hpmmbq	2-Hexaprenyl-3-methyl-6-methoxy-1,4-benzoquinone		C38H56O3	0	0	0
hppp	all-trans-Hexaprenyl diphosphate		C30H52O7P2	-2	-2	-3
hppr	Hippurate	Hippuric acid/N-Benzoylglycine/Benzoylaminoacetic acid	C9H9NO3	-1	-1	-1
hpyr	Hydroxypyruvate	Hydroxypyruvic acid/3-Hydroxypyruvate/3-Hydroxypyruvic acid	C3H4O4	-1	-1	-1
hqn	p-Benzenediol	Hydroquinone/1,4-Benzenediol/1,4-Dihydroxybenzene/Benzene-1,4-diol/Quinol/4-Hydroxyphenol	C6H6O2	0	0	0
hser	L-Homoserine	2-Amino-4-hydroxybutyric acid	C4H9NO3	0	0	0
hsulpob	(Z)-4-(2-Hydroxy-5-sulfonatophenyl)-2-oxo-3-butenoate		C10H8O7S	-2	-2	-2
ht5p	Hydantoin-5-propionate	Hydantoin-propionate	C6H8N2O4	-1	-1	-1
hxsp	2-Hydroxyisophthalic acid	2-Hydroxy-1,3-benzenedicarboxylic acid	C8H6O5	-2	-2	-2
hydroxyakg	D-4-Hydroxy-2-oxoglutarate		C5H6O6	-2	-2	-2
hyxn	Hypoxanthine	Purine-6-ol	C5H4N4O	0	0	0
i3aa	Indole-3-acetaldehyde	2-(Indol-3-yl)acetaldehyde/Indoleacetaldehyde	C10H9NO	0	0	0
i3ac	Indole-3-acetate	Indole-3-acetic acid/(Indol-3-yl)acetate/Indoleacetate/Indoleacetic acid	C10H9NO2	-1	-1	-1
i4aa	Imidazole-4-acetaldehyde	Imidazole acetaldehyde	C5H6N2O	1	0	0
i4ac	Imidazole-4-acetate	Imidazoleacetic acid/4-Imidazoleacetate	C5H6N2O2	0	-1	-1
iasp	Iminoaspartate		C4H5NO4	-2	-2	-2
icit	Isocitrate	Isocitric acid/1-Hydroxytricarballylic acid/1-Hydroxypropane-1,2,3-tricarboxylic acid	C6H8O7	-3	-3	-3
id3act	Indole-3-acetamide		C10H10N2O	0	0	0
idactn	3-Indoleacetonitrile	Indol-3-ylacetonitrile/Indole-3-acetonitrile/(Indol-3-yl)acetonitrile	C10H8N2	0	0	0
idlac	Indolelactate		C11H11NO3	-1	-1	-1
idon	L-Idonate		C6H12O7	-1	-1	-1
idp	IDP	Inosine 5'-diphosphate/Inosine diphosphate	C10H14N4O11P2	-2	-2	-3
idpyr	Indolepyruvate	Indolepyruvic acid/(Indol-3-yl)pyruvate/Indole-3-pyruvate/3-(Indol-3-yl)pyruvate	C11H9NO3	-1	-1	-1
ile	L-Isoleucine	2-Amino-3-methylvaleric acid	C6H13NO2	0	0	0
iletrna	L-Isoleucyl-tRNA(Ile)		C21H32N6O11PR(C5H8O6PR)n	1	1	1
imACP	3-(Imidazol-4-yl)-2-oxopropyl phosphate	Imidazole-acetol phosphate	C6H9N2O5P	0	-2	-2
imal	Isomaltose	Brachiose	C12H22O11	0	0	0
imgly	Iminoglycine	Iminoacetic acid	C2H3NO2	-1	-1	-1
imp	IMP	Inosinic acid/Inosine monophosphate/Inosine 5'-monophosphate/Inosine 5'-phosphate/5'-Inosinate/5'-Inosinic acid/5'-Inosine /onophosphate/5'-IMP	C10H13N4O8P	-1	-2	-2
indole	Indole	2,3-Benzopyrrole	C8H7N	0	0	0
ins	Inosine		C10H12N4O5	0	0	0

ipp	Isopentenyl diphosphate	delta3-Isopentenyl diphosphate/delta3-Methyl-3-butenyl diphosphate	C5H12O7P2	-2	-2	-3
isoppam	N-Isopropylammelide		C6H10N4O2	0	0	0
itcn	Itaconate	Itaconic acid/Methylenesuccinic acid	C5H6O4	-2	-2	-2
itcncoa	Itaconyl-CoA		C26H40N7O19P3S	-4	-5	-5
itp	ITP	Inosine 5'-triphosphate/Inosine triphosphate/Inosine tripolyphosphate	C10H15N4O14P3	-3	-3	-4
k	potassium	K+	K	1	1	1
k2lipiv	Di[3-deoxy-D-manno-octulosonyl]-lipid IV(A)	KDO2-lipid IV(A)	C84H154N2O37P2	-4	-6	-6
kdg	2-Dehydro-3-deoxy-D-gluconate		C6H10O6	-1	-1	-1
kdo	3-Deoxy-D-manno-octulosonate	KDO/2-Dehydro-3-deoxy-D-octonate/3-Deoxy-D-manno-2-octulosonate/3-Deoxyoctulosonic acid	C8H14O8	-1	-1	-1
kdo8p	3-Deoxy-D-manno-octulosonate 8-phosphate	2-Dehydro-3-deoxy-D-octonate 8-phosphate	C8H15O11P	-2	-3	-3
kdolipid4	3-Deoxy-D-manno-octulosonyl-lipid IV(A)	KDO-lipid IV(A)/3-Deoxy-D-manno-octulosonyl-2',3',2',3'-tetrakis(beta-hydroxymyristoyl)-D-glucosaminyl-1,6-beta-D-glucosamine 1,4'-bisphosphate	0	-3	-5	-5
kdpg	2-Dehydro-3-deoxy-6-phospho-D-gluconate	6-Phospho-2-dehydro-3-deoxy-D-gluconate/2-Keto-3-deoxy-6-phosphogluconate/2-Dehydro-3-deoxy-D-gluconate 6-phosphate	C6H11O9P	-2	-3	-3
kn	L-Kynurenine	3-Anthraniloyl-L-alanine	C10H12N2O3	0	0	0
knt	Kynurenate	4-Hydroxy-2-quinolinecarboxylic acid/Kynurenic acid	C10H7NO3	-1	-1	-1
l1p3h5c	L-1-Pyrroline-3-hydroxy-5-carboxylate	3-Hydroxy-L-1-pyrroline-5-carboxylate	C5H7NO3	-1	-1	-1
l3aibn	L-3-Amino-isobutanoate	(S)-3-Amino-isobutyrate/L-3-Amino-isobutyrate/(S)-3-Amino-isobutanoate/(S)-3-Amino-2-methylpropanoate	C4H9NO2	0	0	0
lac	D-Lactate	(R)-Lactate/D-Lactic acid/D-2-Hydroxypropanoic acid/D-2-Hydroxypropionic acid	C3H6O3	-1	-1	-1
lactcoa	Lactoyl-CoA		C24H40N7O18P3S	-3	-4	-4
lactose	Lactose	1-beta-D-Galactopyranosyl-4-alpha-D-glucopyranose/Milk sugar/alpha-Lactose/Anhydrous lactose	C12H22O11	0	0	0
larabinose	L-Arabinose	L-Arabinopyranose	C5H10O5	0	0	0
leu	L-Leucine	2-Amino-4-methylvaleric acid/(2S)-alpha-2-Amino-4-methylvaleric acid/(2S)-alpha-Leucine	C6H13NO2	0	0	0
leutrna	L-Leucyl-tRNA(Leu)	L-Leucyl-tRNA	C21H32N6O11PR(C5H8O6PR)n	1	1	1
lipa	Di[3-deoxy-D-manno-octulosonyl]-lipid A	KDO2-lipid (A)	C110H202N2O39P2	-4	-6	-6
lipidA	2,3,2'3'-Tetrakis(3-hydroxytetradecanoyl)-D-glucosaminyl-1,6-beta-D-glucosamine 1,4'-bisphosphate	2,3,2'3'-Tetrakis(beta-hydroxymyristoyl)-D-glucosaminyl-1,6-beta-D-glucosamine 1,4'-bisphosphate/Lipid A disaccharide bisphosphate/Lipid IV(A)	C68H130N2O23P2	-2	-4	-4
lipidAds	2,3,2',3'-Tetrakis(3-hydroxytetradecanoyl)-D-glucosaminyl-1,6-beta-D-glucosamine 1-phosphate	2,3-Bis(3-hydroxytetradecanoyl)-D-glucosaminyl-1,6-beta-D-2,3-bis(3-hydroxytetradecanoyl)-beta-D-glucosaminyl 1-phosphate/2,3-Bis-(beta-hydroxymyristoyl)-D-glucosaminyl-(beta-D-1,6)-2,3-bis(beta-hydroxymyristoyl)-D-glucosaminyl beta-phosphate/2,3-Bis-(3-hydroxytetradecanoyl)-D-glucosaminyl-(beta-D-1,6)-2,3-bis(3-hydroxy-tetradecanoyl)-D-glucosaminyl beta-phosphate/Lipid A disaccharide	C68H129N2O20P	-1	-1	-1
lipidX	2,3-Bis(3-hydroxytetradecanoyl)-beta-D-glucosaminyl 1-phosphate	2,3-Bis(beta-hydoroxymyristoyl)-beta-D-glucosaminyl 1-phosphate/Lipid X	C34H66NO12P	-1	-2	-2
lipo	Lipoamide-E	Enzyme N6-(lipoyl)lysine	C8H14NOS2R	0	0	0
lipoe	Lipoamide	Thioctic acid amide	C8H15NOS2	0	0	0
lipop	Lipoylprotein	H-Protein-lipoyllysine	C8H14NOS2R	0	0	0
lk2lipiv	Lauroyl-KDO2-lipid IV(A)		C96H176N2O38P2	-4	-6	-6
llac	D-Lactate	(S)-Lactate/L-Lactic acid	C3H6O3	-1	-1	-1
llald	L-Lactaldehyde	(S)-Lactaldehyde/L-2-Hydroxypropionaldehyde	C3H6O2	0	0	0
llct	L-Cystathionine		C7H14N2O4S	0	0	0
LPS	lipopolisaccharide		0	0	0	0
ltg	(R)-S-Lactoylglutathione		C13H21N3O8S	-1	-1	-1
lys	L-Lysine	Lysine acid/2,6-Diaminohexanoic acid	C6H14N2O2	1	1	1
lystrna	L-Lysine-tRNA (Lys)	L-Lysyl-tRNA	C16H29N2O11PR2(C5H8O6PR)n	2	2	2
maco	cis-2-Methylaconitate	Z)-But-2-ene-1,2,3-tricarboxylate	C7H8O6	-3	-3	-3
mal	L-Malate	(S)-Malate/L-Apple acid/L-Malic acid/L-2-Hydroxybutanedioic acid	C4H6O5	-2	-2	-2
malACP	Malonyl-[acyl-carrier protein]		C3H3O3SR	-2	-2	-2
malcoa	Malonyl-CoA	Malonyl coenzyme A	C24H38N7O19P3S	-4	-5	-5
malpyr	Maleylpyruvate	Maleylpyruvic acid/3-Maleylpyruvate	C7H6O6	-2	-2	-2
malte	maleate	maleic acid/cis-Butenedioic acid	C4H4O4	-2	-2	-2
malthp	Celloheptaose		C42H72O36	0	0	0
maltpt	Cellopentaose		C30H52O26	0	0	0

man	D-Mannose	Mannose/Seminose/Carubinose	C6H12O6	0	0	0
man1p	D-Mannose 1-phosphate	alpha-D-Mannose 1-phosphate	C6H13O9P	-1	-2	-2
man6p	D-Mannose 6-phosphate		C6H13O9P	-1	-2	-2
mbzald	2-Methylbenzaldehyde	o-Toluic aldehyde/2-Formyltoluene/o-Tolualdehyde	C8H8O	0	0	0
mclact	(S)-5-Oxo-2,5-dihydrofuran-2-acetate	(+)-Muconolactone	C6H6O4	-1	-1	-1
mde4p	2-C-methyl-D-erythritol 4-phosphate		C5H13O7P	-1	-2	-2
mdecpp	2-C-methyl-D-erythritol 2,4-cyclodiphosphate	3-Methyl-1,2,3,4-tetrahydroxybutane-1,3-cyclic bisphosphate	C5H12O9P2	-2	-2	-2
meli	Melibiose	6-O-(alpha-D-Galactopyranosyl)-D-glucopyranose/D-Gal-alpha1->6D-Glucose	C12H22O11	0	0	0
met	L-Methionine	Methionine/L-2-Amino-4methylthiobutyric acid	C5H11NO2S	0	0	0
methf	5,10-Methenyltetrahydrofolate		C20H22N7O6	-1	-1	-1
methl	Methylamine	Methanamine	CH5N	1	1	1
metp	L-Metanephrine		C10H15NO3	1	1	1
metthf	5,10-Methylenetetrahydrofolate	(6R)-5,10-Methylenetetrahydrofolate/5,10-Methylene-THF	C20H23N7O6	-2	-2	-2
mettrna	L-Methionyl-tRNA (Met)	L-Methionyl-tRNA	C20H30N6O11PSR(C5H8O6PR)n	1	1	1
mg2	Magnesium	Mg2+	Mg	2	2	2
mi	myo-Inositol	D-myo-Inositol/1D-myo-Inositol/L-myo-Inositol/1L-myo-Inositol/meso-Inositol/Inositol/Dambose/Cyclohexitol/Meat sugar/Bios I	C6H12O6	0	0	0
micit	methylisocitrate	(2S,3R)-3-Hydroxybutane-1,2,3-tricarboxylate/Methylisocitric acid	C7H10O7	-3	-3	-3
mk	Menaquinone	Menatetrenone	C16H16O2(C5H8)n	0	0	0
mlt	Maltose	Malt sugar/1-alpha-D-Glucopyranosyl-4-alpha-D-glucopyranose	C12H22O11	0	0	0
mlthx	Maltohexaose		C36H62O31	0	0	0
mlttr	Maltotriose	Amylotriose	C18H32O16	0	0	0
mltttr	Maltotetraose		C24H42O21	0	0	0
mlzac	Methylimidazole acetaldehyde	1-Methylimidazole-4-acetaldehyde/Methylimidazoleacetaldehyde	C6H8N2O	1	0	0
mm	Methylmalonate	Methylmalonic acid	C4H6O4	-1	-2	-2
mmcoa-R	(R)-Methylmalonyl-CoA	(R)-2-Methyl-3-oxopropanoyl-CoA/(R)-2-Methyl-3-oxopropionyl-CoA/(R)-3-Oxo-2-methylpropanoyl-CoA	C25H40N7O19P3S	-4	-5	-5
mmcoa-S	(S)-Methylmalonyl-CoA	(S)-2-Methyl-3-oxopropanoyl-CoA/(S)-2-Methyl-3-oxopropionyl-CoA/(S)-Methylmalonyl-coenzyme A/(S)-3-Oxo-2-methylpropanoyl-CoA	C25H40N7O19P3S	-4	-5	-5
mmsa	(S)-Methylmalonate semialdehyde		C4H6O3	-1	-1	-1
mnapth	1-Methylnaphthalene	alpha-Methylnaphthalene	C11H10	0	0	0
mnt	D-Mannitol	Mannitol	C6H14O6	0	0	0
mnt1p	D-Mannitol 1-phosphate		C6H15O9P	-1	-2	-2
mobd	Molybdate	Sodium molybdate/Sodium molybdate(VI)	MoO4. 2Na	-2	-2	-2
motym	3-Methoxytyramine		C9H13NO2	1	1	1
mpyr	Mercaptopyruvate	3-Mercaptopyruvate	C3H4O3S	-1	-1	-1
msalc	3-Methylsalicylate		C8H8O3	-1	-1	-1
msalc4	4-Methylsalicylate	m-Cresotic acid/2-Hydroxy-4-methylbenzoic acid	C8H8O3	-1	-1	-1
msalcah	3-Methylsalicylaldehyde	2-Hydroxy-3-methylbenzaldehyde	C8H8O2	0	0	0
mtg	Methylglyoxal	Pyruvaldehyde/Pyruvic aldehyde/2-Ketopropionaldehyde/2-Oxopropanal	C3H4O2	0	0	0
mthf	5-Methyltetrahydrofolate		C20H25N7O6	-2	-2	-2
mtolald	3-Methylbenzaldehyde	m-Tolualdehyde	C8H8O	0	0	0
mtolat	m-Methylbenzoate	m-Toluic Acid/beta-Bethylbenzoic acid/m-Toluylic acid/m-Toluate	C8H8O2	-1	-1	-1
n2	nitrogen	N2	N2	0	0	0
n2o	Nitrous oxide	Dinitrogen monoxide/Dinitrogen oxide	N2O	0	0	0
n4aab	N4-Acetylaminobutanal		C6H11NO2	0	0	0
na	Sodium	Na+	Na	1	1	1
nac	Nicotinate	Nicotinic acid/Niacin/3-Pyridinecarboxylic acid	C6H5NO2	-1	-1	-1
nacd	Nicotinate D-ribonucleoside		C11H14NO6	0	0	0
nacn	Nicotinate D-ribonucleotide	beta-Nicotinate D-ribonucleotide/Nicotinate ribonucleotide/Nicotinic acid ribonucleotide	C11H15NO9P	-1	-2	-2
nad	Nicotinamide adenine dinucleotide	NAD+/NAD/DPN/Diphosphopyridine nucleotide/Nadide	C21H28N7O14P2	-1	-1	-1
nadh	Nicotinamide adenine dinucleotide - reduced	NADH/DPNH	C21H29N7O14P2	-2	-2	-2
nadma	N-Acetyl-D-mannosamine	2-Acetamido-2-deoxy-D-mannose	C8H15NO6	0	0	0
nadma6p	N-Acetyl-D-mannosamine 6-phosphate	N-Acetylmannosamine 6-phosphate	C8H16NO9P	-1	-2	-2
nadp	Nicotinamide adenine dinucleotide phosphate	NADP+/NADP/beta-Nicotinamide adenine dinucleotide phosphate/TPN/Triphosphopyridine nucleotide	C21H29N7O17P3	-2	-3	-3
nadph	Nicotinamide adenine dinucleotide phosphate - reduced	NADPH/TPNH	C21H30N7O17P3	-4	-4	-4
naga	N-Acetyl-D-glucosamine	N-Acetylchitosamine/2-Acetamido-2-deoxy-D-glucose/GlcNAc	C8H15NO6	0	0	0
naga1p	N-Acetyl-D-glucosamine 1-phosphate		C8H16NO9P	-1	-2	-2

naga6p	N-Acetyl-D-glucosamine 6-phosphate		C8H16NO9P		-1	-2	-2
naglu	N-Acetyl-L-glutamate	N-Acetyl-L-glutamic acid	C7H11NO5		-2	-2	-2
naglus	N-Acetyl-L-glutamate 5-semialdehyde	2-Acetamido-5-oxopentanoate	C7H11NO4		-1	-1	-1
nam	Nicotinamide	Nicotinic acid amide/Niacinamide/Vitamin PP	C6H6N2O		0	0	0
namd	N-Ribosylnicotinamide	1-(beta-D-Ribofuranosyl)nicotinamide	C11H15N2O5		1	1	1
namn	Nicotinamide D-ribonucleotide	NMN/Nicotinamide mononucleotide/Nicotinamide ribonucleotide/Nicotinamide nucleotide/beta-Nicotinamide D-ribonucleotide/beta-Nicotinamide ribonucleotide/beta-Nicotinamide mononucleotide	C11H15N2O8P		0	-1	-1
naneu	N-Acetylneuraminate	N-Acetylneuraminic acid/5-Acetamido-3,5-dideoxy-D-glycero-D-galacto-2-nonulosonic acid/Neu5Ac	C11H19NO9		-1	-1	-1
naorn	N2-Acetyl-L-ornithine	N-Acetylornithine	C7H14N2O3		0	0	0
nap2msuccoa	Naphthyl-2-methylene-succinyl-CoA		C36H46N7O19P3S		-4	-5	-5
naphmsuccoa	Naphthyl-2-hydroxymethyl-succinyl CoA		C36H48N7O20P3S		-4	-5	-5
napmsccoa	Naphthyl-2-methyl-succinyl-CoA		C36H48N7O19P3S		-4	-5	-5
napomsuccoa	Naphthyl-2-oxomethyl-succinyl-CoA		C36H46N7O20P3S		-4	-5	-5
napth12d	Naphthalene-1,2-diol	1,2-Naphthalenediol/beta-Naphthohydroquinone/1,2-Dihydroxynaphthalene	C10H8O2		0	0	0
napth2ms	Naphthyl-2-methyl-succinic acid		C15H14O4		-2	-2	-2
napthah	1-Naphthaldehyde	1-Formylnaphthalene	C11H8O		0	0	0
nfglu	N-Formimino-L-glutamate	N-Formimidoyl-L-glutamate	C6H10N2O4		-1	-1	-1
nh4	Ammonium		NH4		1	1	1
nicatol	4-Nitrocatechol		C6H5NO4		0	0	-1
nitbz	Nitrobenzene	Nitrobenzol	C6H5NO2		0	0	0
nmhis	N-Methylhistamine	1-Methylhistamine/1-Methyl-4-(2-aminoethyl)imidazole/Ntau-Methylhistamine	C6H11N3		2	1	1
no	Nitric oxide	NO/Nitrogen monoxide	NO		0	0	0
no2	Nitrite		HNO2		-1	-1	-1
no3	Nitrate	Nitric acid	HNO3		-1	-1	-1
norad	L-Noradrenaline	Noradrenaline/Norepinephrine/Arterenol/4-[(1R)-2-Amino-1-hydroxyethyl]-1,2-benzenediol	C8H11NO3		1	1	1
normp	L-Normetanephrine		C9H13NO3		1	1	1
npran	N-(5-Phospho-D-ribosyl)anthranilate	N-(5-Phospho-beta-D-ribosyl)anthranilate/N-(5-Phosphoribosyl)anthranilic acid	C12H16NO9P		-2	-3	-3
ntphp	4-Nitrophenyl phosphate		C6H6NO6P		-1	-2	-2
o2	Oxygen	O2	O2		0	0	0
oaa	Oxaloacetate	Oxalacetic acid/Oxaloacetic acid/2-Oxobutanedioic acid/Oxosuccinic acid/keto-Oxaloacetate	C4H4O5		-2	-2	-2
oadip	3-Oxoadipate	3-Oxoadipic acid/3-Keto-adipate	C6H8O5		-2	-2	-2
oahser	O-Acetyl-L-homoserine		C6H11NO4		0	0	0
obut	2-Oxobutanoate	2-Ketobutyric acid/2-Oxobutyric acid/2-Oxobutyrate/2-Oxobutanoic acid/alpha-Ketobutyric acid/alpha-Ketobutyrate	C4H6O3		-1	-1	-1
ocresol	o-Cresol	2-Hydroxytoluene/o-Methylphenol	C7H8O		0	0	0
ogt	Oxidized glutathione	Glutathione disulfide/GSSG/Oxiglutatione	C20H32N6O12S2		-2	-2	-4
ohchtcht	6-Oxo-2-hydroxy-7-(4'-chlorophenyl)-3,8,8-trichloroocta-2E,4E,7E-trienoate		C14H8Cl4O4		-1	-1	-1
ohpb	2-Oxo-3-hydroxy-4-phosphobutanoate	alpha-Keto-3-hydroxy-4-phosphobutyrate/(3R)-3-Hydroxy-2-oxo-4-phosphonooxybutanoate	C4H7O8P		-2	-3	-3
ohtchod	6-Oxo-2-hydroxy-7-(4'-chlorophenyl)-3,8,8,8-tetrachloroocta-2E,4E-dienoate		C14H9Cl5O4		-1	-1	-1
oicap	3-Carboxy-4-methyl-2-oxopentanoate	(2S)-2-Isopropyl-3-oxosuccinate/2-Oxo-4-methyl-3-carboxypentanoate	C7H10O5		-2	-2	-2
omcmc	2-Oxo-5-methyl-cis-muconate		C7H8O5		-2	-2	-2
omp	Orotidine 5'-phosphate	Orotidylic ac	C10H13N2O11P		-2	-3	-3
ooadpcoa	3-Oxoadipyl-CoA		C27H42N7O20P3S		-4	-5	-5
oohoch	1-Oxa-2-oxo-3-hydroxycycloheptane		C6H10O3		0	0	0
op4en	2-Oxopent-4-enoate	2-Hydroxy-2,4-pentadienoate/cis-2-Hydroxypenta-2,4-dienoate/Oxopent-4-enoate/2-Hydroxypenta-2,4-dienoate	C5H6O3		-1	-1	-1
opimcoa	3-Oxopimeloyl-CoA	3-Ketopimelyl-CoA	C28H44N7O20P3S		-4	-5	-5
opp	all-trans-Octaprenyl diphosphate	Farnesylfarnesylgeraniol	C40H68O7P2		-2	-2	-3
orn	L-Ornithine	(S)-2,5-Diaminovaleric acid/(S)-2,5-Diaminopentanoic acid/(S)-2,5-Diaminopentanoate	C5H12N2O2		1	1	1
oroa	Orotate	Orotic acid/Uracil-6-carboxylic acid	C5H4N2O4		-1	-1	-1
oslhser	O-Succinyl-L-homoserine		C8H13NO6		-1	-1	-1
othio	Oxidized thioredoxin	Thioredoxin disulfide/Thioredoxin sulfide	C6H7NO2S2R2		0	0	0
otolat	o-Toluate	o-Methylbenzoate/o-Toluic Acid/2-Methylbenzoic acid	C8H8O2		-1	-1	-1
p5c	1-Pyrroline-5-carboxylate	(S)-1-Pyrroline-5-carboxylate/L-1-Pyrroline-5-carboxylate	C5H7NO2		0	-1	-1
pa	Phosphatidate			0	-1	-2	-2
paba	4-Aminobenzoate	ABEE/4-Aminobenzoic acid/p-Aminobenzoate	C7H7NO2		-1	-1	-1
pac	Phenylacetic acid	Benzylformic acid/Phenylacetate/Benzeneacetiic acid	C8H8O2		-1	-1	-1
pacald	Phenylacetaldehyde	alpha-Tolualdehyde	C8H8O		0	0	0

pant	(R)-Pantoate	Pantoate/Pantoic acid	C6H12O4		-1	-1	-1
pantcys	N-((R)-Pantothenoyl)-L-cysteine	D-Pantothenoyl-L-cysteine/N-Pantothenoylcysteine	C12H22N2O6S		-1	-1	-1
pap	Adenosine 3',5'-bisphosphate	PAP/3'-Phosphoadenylate/Phosphoadenosine phosphate	C10H15N5O10P2		-2	-4	-4
paps	3'-Phosphoadenylyl sulfate	3'-Phosphoadenosine 5'-phosphosulfate/3'-Phospho-5'-adenylyl sulfate/PAPS	C10H15N5O13P2S		-3	-4	-4
parat	Parathion	O,O-Diethyl O-p-nitrophenyl phosphorothioate/Thiophos/DNTP	C10H14NO5PS		0	0	0
parax	Paraoxon	O,O-Diethyl-O-p-nitrophenylphosphoric acid	C10H14NO6P		0	0	0
pbg	Porphobilinogen		C10H14N2O4		-1	-1	-1
pbhb	Poly-beta-hydroxybutyrate		(C4H6O2)n		0	0	0
pbzq	p-Benzoquinone	Quinone/Chinone/2,5-Cyclohexadiene-1,4-dione	C6H4O2		0	0	0
pc	phosphatidylcholine	Lecithin/Phosphatidyl-N-trimethylethanolamine/1,2-Diacyl-sn-glycero-3-phosphocholine/Choline phosphatide/3-sn-Phosphatidylcholine	C10H18NO8PR2		0	0	0
pdla	Pyridoxamine	PM	C8H12N2O2		1	1	1
pdx5p	Pyridoxine 5'-phosphate	Pyridoxine phosphate/Pyridoxine 5-phosphate	C8H12NO6P		-1	-2	-2
pe	Phosphatidylethanolamine	(3-Phosphatidyl)ethanolamine/(3-Phosphatidyl)-ethanolamine/Cephalin/O-(1-beta-Acyl-2-acyl-sn-glycero-3-phospho)ethanolamine/1-Acyl-2-acyl-sn-glycero-3-phosphoethanolamine	C7H12NO8PR2		0	0	0
peamn	Phenethylamine	2-Phenylethylamine/beta-Phenylethylamine/Phenylethylamine	C8H11N		1	1	1
pep	Phosphoenolpyruvate	Phosphoenolpyruvic acid/PEP	C3H5O6P		-2	-2	-2
PEPTIDO	peptidoglycan			0	0	0	0
pg	Phosphatidylglycerol	3-(3-sn-Phosphatidyl)glycerol/3(3-Phosphatidyl-)glycerol/PtdGro	C8H13O10PR2		-1	-1	-1
pgp	Phosphatidylglycerophosphate	3(3-sn-Phosphatidyl)-sn-glycerol 1-phosphate/3(3-Phosphatidyl-)L-glycerol 1-phosphate/1,2-Diacyl-sn-glycero-3-phospho-sn-glycerol 3'-phosphate	C8H14O13P2R2		-2	-3	-3
phaccoa	Phenylacetyl-CoA		C29H42N7O17P3S		-3	-4	-4
phatol	1-Phenanthrol		C14H10O		0	0	0
phe	L-Phenylalanine	(S)-alpha-Amino-beta-phenylpropionic acid	C9H11NO2		0	0	0
pheacgly	Phenylacetylglycine		C10H11NO3		-1	-1	-1
pheacnit	Phenylacetonitrile	Benzyl cyanide	C8H7N		0	0	0
pheact	2-Phenylacetamide		C8H9NO		0	0	0
pheborn	Phenylboronic acid	Benzeneboronic acid	C6H7BO2		0	0	0
phen	Prephenate	Prephenic acid	C10H10O6		-2	-2	-2
phenol	Phenol	Benzenol/Hydroxybenzene/Phenic acid/Phenylic acid	C6H6O		0	0	0
phentrc	Phenanthracene	Phenanthrene	C14H10		0	0	0
pheth12o	Phenanthrene-1,2-oxide		C14H10O		0	0	0
pheth910o	Phenanthrene-9,10-oxide		C14H10O		0	0	0
phetrna	L-Phenylalanyl-tRNA(Phe)		C19H26NO11PR2(C5H8O6PR)n		1	1	1
PHOSPHOLIPID	phospholipid			0	0	0	0
phpyr	Phenylpyruvate	Phenylpyruvic acid/alpha-Ketohydrocinnamic acid/keto-Phenylpyruvate/3-Phenyl-2-oxopropanoate	C9H8O3		-1	-1	-1
phser	O-Phospho-L-homoserine		C4H10NO6P		-1	-2	-2
pht	O-Phospho-4-hydroxy-L-threonine	4-(Phosphonooxy)-threonine/4-(Phosphonooxy)-L-threonine	C4H10NO7P		-1	-2	-2
pi	Phosphate	Orthophosphate/Phosphoric acid/Orthophosphoric acid	H3PO4		-1	-2	-2
pimlt	6-Carboxyhexanoate	Pimelate/Pimelic acid/Heptanedioic acid	C7H12O4		-2	-2	-2
pl	Pyridoxal		C8H9NO3		0	0	-1
pmcoa	Pimeloyl-CoA	6-Carboxyhexanoyl-CoA	C28H46N7O19P3S		-3	-4	-5
pnp	4-Nitrophenol	p-Nitrophenol/PNP/Niphen/4-Hydroxynitrobenzene	C6H5NO3		0	0	-1
pnpq	2-Phytyl-1,4-naphthoquinone	Demethylphylloquinone	C30H44O2		0	0	0
pnto	(R)-Pantothenate	Pantothenate/Pantothenic acid	C9H17NO5		-1	-1	-1
ppa	Propionate	Propanoate/Propanoic acid/Propionic acid	C3H6O2		-1	-1	-1
ppacp	propionyl-[acyl carrier protein]		C14H26N2O8PRS		-1	-1	-1
ppadsel	3'-Phosphoadenylylselenate	3'-Phosphoadenosine-5'-phosphoselanate	C10H16N5O13P2Se		-1	-2	-3
ppald	Propinol adenylate	Propionyladenylate	C13H18N5O8P		-1	-1	-1
ppap	Propanoyl phosphate	Propionyl phosphate	C3H7O5P		-2	-2	-2
ppcoa	Propanoyl-CoA	Propionyl-CoA/Propionyl coenzyme A	C24H40N7O17P3S		-3	-4	-4
ppecoa	Propenoyl-CoA	Acryloyl-CoA/Acrylyl-CoA	C24H38N7O17P3S		-3	-4	-4
ppeptido	peptidoglycan precursor			0	0	0	0
ppgpp	Guanosine 3',5'-bis(diphosphate)	Guanosine 3'-diphosphate 5'-diphosphate/Guanosine 5'-diphosphate,3'-diphosphate	C10H17N5O17P4		-4	-5	-6
pphg	Protoporphyrinogen IX		C34H40N4O4		-2	-2	-2
ppi	Diphosphate	Pyrophosphate/Pyrophosphoric acid/PPi	P2H4O7		-2	-3	-4
ppix	Protoporphyrin	Protoporphyrin IX/Porphyrinogen IX	C34H34N4O4		-2	-2	-2
ppn	Propynoate	Propiolic acid/Acetylenecarboxylic acid/Acetylenemonocarboxylate	C3H2O2		-1	-1	-1

pppgpp	Guanosine 3'-diphosphate 5'-triphosphate	Guanosine 5'-triphosphate,3'-diphosphate	C10H18N5O20P5		-5	-6	-7
pppi	Inorganic triphosphate		P3H5O10		-3	-3	-5
pppn	Phenylpropanoate	3-Phenyl-propionic acid/3-Phenylpropanoic acid	C9H10O2		-1	-1	-1
ppyr	Parapyruvate	4-Hydroxy-4-methyl-2-oxoglutarate	C6H8O6		-2	-2	-2
pq	Phylloquinone	Vitamin K1/Phytonadione/2-Methyl-3-phytyl-1,4-naphthoquinone	C31H46O2		0	0	0
pqppi	Presqualene diphosphate		C30H52O7P2		-2	-2	-3
pqq	PQQ	Pyrrolo-quinoline quinone/Pyrroloquinoline-quinone/Pyrroloquinoline quinone/4,5-Dioxo-4,5-dihydro-1H-pyrrolo[2,3-f]quinoline-2,7,9-tricarboxylate	C14H6N2O8		-3	-3	-3
pqqh2	PQQH2	Reduced pyrroloquinoline-quinone	C14H8N2O8		-3	-3	-3
pram	5-Phosphoribosylamine	5-Phospho-beta-D-ribosylamine/5-Phospho-D-ribosylamine/5-Phosphoribosyl-1-amine	C5H12NO7P		0	-1	-2
prbamp	1-(5-Phosphoribosyl)-AMP	Phosphoribosyl-AMP/N1-(5-Phospho-D-ribosyl)-AMP	C15H23N5O14P2		-2	-3	-4
prbatp	1-(5-Phosphoribosyl)-ATP	Phosphoribosyl-ATP/N1-(5-Phospho-D-ribosyl)-ATP		0	-4	-5	-6
prfp	1-(5-Phosphoribosyl)-5-[(5-phosphoribosylamino)methylideneamino]imidazole-4-carboxamide	-(5-Phospho-D-ribosylaminoformimino)-1-(5-phosphoribosyl)-imidazole-4-carboxamide/N-(5'-Phospho-D-ribosylformimino)-5-amino-1-(5''-phospho-D-ribosyl)-4-imidazolecarboxamide/N-(5'-Phosphoribosylformimino)-5-amino-1-(5''-phosphoribosyl)-4-imidazolecarboxamide Phosphoribosyl-formimino-AICAR-phosphate	C15H25N5O15P2		-1	-3	-4
prlp	5-[(5-Phospho-1-deoxyribulos-1-ylamino)methylideneamino]-1-(5-phosphoribosyl)imidazole-4-carboxamide	N-(5'-Phospho-D-1'-ribulosylformimino)-5-amino-1-(5''-phospho-D-ribosyl)-4-imidazolecarboxamide/Phosphoribulosyl-formimino-AICAR-phosphate	C15H25N5O15P2		-1	-3	-4
pro	L-Proline	2-Pyrrolidinecarboxylic acid	C5H9NO2		0	0	0
progly	L-Prolinylglycine		C7H12N2O3		0	0	0
propen	Propenoate	Acrylic acid/Acrylate/2-Propenoic acid/Vinylformic acid	C3H4O2		-1	-1	-1
PROTEIN	protein			0	0	0	0
protmn	Protoanemonin	4-Methylenebut-2-en-4-olide/cis-4-Methylenebut-2-en-4-olide	C5H4O2		0	0	0
protrna	L-Prolyl-tRNA(Pro)		C15H24NO11PR2(C5H8O6PR)n		1	1	1
prpp	5-Phospho-alpha-D-ribose 1-diphosphate	5-Phosphoribosyl diphosphate/5-Phosphoribosyl 1-pyrophosphate/PRPP	C5H13O14P3		-3	-4	-5
ps	Phosphatidylserine	Phosphatidyl-L-serine/1,2-Diacyl-sn-glycerol 3-phospho-L-serine/3-O-sn-Phosphatidyl-L-serine/O3-Phosphatidyl-L-serine	C8H12NO10PR2		-2	-2	-2
pser	O-Phospho-L-serine	L-O-Phosphoserine/3-Phosphoserine	C3H8NO6P		-1	-2	-2
pth	Protoheme	Heme/Haem/Heme B/Protoheme IX	C34H32FeN4O4		-2	-2	-2
ptolald	p-Tolualdehyde	p-Methylbenzaldehyde/4-Toluylaldehyde/p-Formyltoluene	C8H8O		0	0	0
ptolat	Toluate	p-Toluate/p-Toluic acid/4-Methylbenzoic acid/Toluenecarboxylic acid/Crithminic acid	C8H8O2		-1	-1	-1
ptrc	Putrescine	1,4-Butanediamine/1,4-Diaminobutane/Tetramethylenediamine	C4H12N2		2	2	2
ptt	Pantetheine	(R)-Pantetheine	C11H22N2O4S		0	0	0
puri5p	Pseudouridine 5'-phosphate		C9H13N2O9P		-1	-2	-2
pyam5p	Pyridoxamine 5'-phosphate	Pyridoxamine phosphate/Pyridoxamine 5-phosphate	C8H13N2O5P		0	-1	-1
pydx5p	Pyridoxal 5'-phosphate	Pyridoxal 5-phosphate/Pyridoxal phosphate	C8H10NO6P		-1	-2	-3
pydxn	Pyridoxine	Pyridoxol	C8H11NO3		0	0	0
pyr	Pyruvate	Pyruvic acid/2-Oxopropanoate/2-Oxopropanoic acid/Pyroracemic acid	C3H4O3		-1	-1	-1
pythp	6-Pyruvoyltetrahydropterin	6-(1,2-Dioxopropyl)-5,6,7,8-tetrahydropterin/6-Pyruvoyl-5,6,7,8-tetrahydropterin	C9H11N5O3		0	0	0
qa	Quinolinate	Pyridine-2,3-dicarboxylate/Quinolinic acid/2,3-Pyridinedicarboxylic acid	C7H5NO4		-2	-2	-2
qt	Quinate	Quinic acid/Kinic acid/Chinic acid/L-Quinic acid/L-Quinate/(-)-Quinic acid	C7H12O6		-1	-1	-1
r15bp	D-Ribose 1,5-bisphosphate	Ribose 1,5-bisphosphate	C5H12O11P2		-2	-4	-4
r1p	alpha-D-Ribose 1-phosphate	Ribose 1-phosphate/D-Ribose 1-phosphate	C5H11O8P		-1	-2	-2
r2mm	(R)-2-Methylmalate	(R)-2-Methylmalic acid/(3R)-Citramalate/(3R)-Citramalic acid/(3R)-alpha-Hydroxypyrotartaric acid/D-Citramalate/D-Citramalic acid/D-alpha-Hydroxypyrotartaric acid/(2R)-2-Hydroxy-2-methylbutanedioate	C5H8O5		-2	-2	-2
r3hbcoa	(R)-3-Hydroxybutanoyl-CoA	(3R)-3-Hydroxybutanoyl-CoA	C25H42N7O18P3S		-3	-4	-4
r3hbn	(R)-3-Hydroxybutanoate	(R)-3-Hydroxybutanoic acid/(R)-3-Hydroxybutyric acid	C4H8O3		-1	-1	-1
r3hbn2	((R)-3-Hydroxybutanoyl)(n-2)		(C4H6O2)n		0	0	0

r3rhbb	(R)-3-((R)-3-Hydroxybutanoyloxy)butanoate		C8H14O5	-1	-1	-1
r5p	alpha-D-Ribose 5-phosphate		C5H11O8P	-1	-2	-2
rbflvrd	Reduced riboflavin		C17H22N4O6	0	0	0
rdmbzi	N1-(alpha-D-ribosyl)-5,6-dimethylbenzimidazole	alpha-Ribazole	C14H18N2O4	0	0	0
resoc	Resorcinol	Resorcin/1,3-Benzenediol/1,3-Dihydroxybenzene/m-Hydroquinone	C6H6O2	0	0	0
rgt	Reduced glutathione	Glutathione/5-L-Glutamyl-L-cysteinylglycine/N-(N-gamma-L-Glutamyl-L-cysteinyl)glycine/gamma-L-Glutamyl-L-cysteinyl-glycine/GSH	C10H17N3O6S	-1	-1	-1
rib	D-Ribose		C5H10O5	0	0	0
ribflav	Riboflavin	Lactoflavin/7,8-Dimethyl-10-ribitylisoalloxazine/Vitamin B2	C17H20N4O6	0	-1	-1
rl5p	D-Ribulose 5-phosphate		C5H11O8P	-1	-2	-2
rmn	L-Rhamnose	6-Deoxy-L-mannose/L-Mannomethylose	C6H12O5	0	0	0
RNA	RNA		0	0	0	0
rthio	Reduced thioredoxin	Thioredoxin	C6H9NO2S2R2	0	0	0
s	Sulfur	S/Sulfur, precipitated	S	0	0	0
s2mbdlipo	S-(2-Methylbutanoyl)-dihydrolipoamide-E	[Dihydrolipoyllysine-residue (2-methylpropanoyl)transferase] S-(2-methylbutanoyl)dihydrolipoyllysine	C13H24NO2S2R	0	0	0
s2mpdlipo	S-(2-Methylpropionyl)-dihydrolipoamide-E	[Dihydrolipoyllysine-residue (2-methylpropanoyl)transferase] S-(2-methylpropanoyl)dihydrolipoyllysine/S-(2-Methylpropanoyl)-dihydrolipoamide-E	C12H22NO2S2R	0	0	0
s3h2mbcoa	(S)-3-Hydroxy-2-methylbutyryl-CoA	(2S,3S)-3-Hydroxy-2-methylbutanoyl-CoA	C26H44N7O18P3S	-3	-4	-4
s3mbdlipo	S-(3-Methylbutanoyl)-dihydrolipoamide-E	[Dihydrolipoyllysine-residue (2-methylpropanoyl)transferase] S-(3-methylbutanoyl)dihydrolipoyllysine	C13H24NO2S2R	0	0	0
s7p	Sedoheptulose 7-phosphate	altro-Heptulose 7-phosphate	C7H15O10P	-1	-2	-2
sah	S-Adenosyl-L-homocysteine	S-Adenosylhomocysteine	C14H20N6O5S	0	0	-1
saicar	1-(5'-Phosphoribosyl)-5-amino-4-(N-succinocarboxamide)-imidazole	1-(5'-Phosphoribosyl)-4-(N-succinocarboxamide)-5-aminoimidazole/5'-Phosphoribosyl-4-(N-succinocarboxamide)-5-aminoimidazole/(S)-2-[5-Amino-1-(5-phospho-D-ribosyl)imidazole-4-carboxamido]succinate/SAICAR	C13H19N4O12P	-3	-3	-3
salcyl	Salicylate	o-Hydroxybenzoic acid/Salicylic acid	C7H6O3	-1	-1	-1
sam	S-Adenosyl-L-methionine	S-Adenosylmethionine/Acylcarnitine	C15H23N6O5S	0	0	-1
sama	S-Adenosylmethioninamine	(5-Deoxy-5-adenosyl)(3-aminopropyl)methylsulfonium salt	C14H23N6O3S	2	2	2
samob	S-Adenosyl-4-methylthio-2-oxobutanoate		C15H20N5O6S	-1	-1	-1
sap	S-Aminomethyldihydrolipoylprotein	[Protein]-S8-aminomethyldihydrolipoyllysine/H-Protein-S-aminomethyldihydrolipoyllysine	C9H19N2OS2R	0	0	0
sarcs	Sarcosine	N-Methylglycine	C3H7NO2	0	0	0
sb1p	Sorbose 1-phosphate	L-Sorbose 1P/L-xylo-Hexulose 1-phosphate/L-Sorbose 1-phosphate	C6H13O9P	-1	-2	-2
sbt6p	D-Sorbitol 6-phosphate	D-Sorbitol 6-phosphate	C6H15O9P	-1	-2	-2
sbzcoa	O-Succinylbenzoyl-CoA	2-Succinylbenzoyl-CoA/Succinylbenzoyl-CoA	C32H44N7O20P3S	-4	-5	-5
scys	Selenocysteine		C3H7NO2Se	0	0	0
seadseh	Se-Adenosylselenohomocysteine		C14H20N6O5Se	0	0	-1
seasmet	Se-Adenosylselenomethionine		C15H24N6O5Se	0	0	0
seld	Selenide	Hydrogen selenide	H2Se	-1	-1	-1
selmtrna	Selenomethionyl-tRNA(Met)		C20H30N6O11PSeR(C5H8O6PR)n	0	0	0
selnp	Selenophosphate		PH3SeO3	-2	-2	-2
selnt	Selenate	Selenic acid	H2SeO4	0	0	0
selt	Selenite		SeO3	0	0	0
ser	L-Serine	L-2-Amino-3-hydroxypropionic acid/L-3-Hydroxy-alanine	C3H7NO3	0	0	0
sertn	3-(2-Aminoethyl)-1H-indol-5-ol	Serotonin/5-Hydroxytryptamine/Enteramine	C10H12N2O	1	1	1
sertrna	L-Seryl-tRNA(Ser)		C13H22NO12PR2(C5H8O6PR)n	0	0	0
sgdhl	S-Glutaryldihydrolipoamide		C13H23NO4S2	-1	-1	-1
shcl	Sirohydrochlorin		C42H46N4O16	-8	-8	-8
shcys	Selenohomocysteine		C4H9NO2Se	0	0	0
shser	O-Succinylhomoserine		C8H13NO6	-1	-1	-1
skm5p	Shikimate 5-phosphate	Shikimate 3-phosphate	C7H11O8P	-2	-3	-3
sl26da	N-Succinyl-LL-2,6-diaminoheptanedioate	N-Succinyl-LL-2,6-diaminopimelate/N-Succinyl-L-2,6-diaminoheptanedioate/N-Succinyl-L-2,6-diaminopimelate	C11H18N2O7	-2	-2	-2
sl2a6o	N-Succinyl-2-L-amino-6-oxoheptanedioate	N-Succinyl-L-2-amino-6-oxoheptanedioate/N-Succinyl-L-2-amino-6-oxopimelate/N-Succinyl-2-amino-6-oxo-L-pimelic acid/N-Succinyl-epsilon-keto-L-aminopimelic acid/(S)-2-(Succinylamino)-6-oxoheptanedioate	C11H15NO8	-3	-3	-3
sllct	Selenocystathionine		C7H14N2O4Se	0	0	0

sme	Shikimate	Shikimic acid/3,4,5-Trihydroxy-1-cyclohexenecarboxylic acid	C7H10O5	-1	-1	-1
smet	Selenomethionine		C5H11NO2Se	0	0	0
so2	sulfur dioxide		SO2	0	0	0
so3	Sulfite		H2SO3	-1	-1	-1
so4	Sulfate	Sulfuric acid	H2SO4	-2	-2	-2
sot	D-Sorbitol	D-Glucitol/L-Gulitol/Sorbitol	C6H14O6	0	0	0
sprm	Spermine	N,N'-Bis(3-aminopropyl)-1,4-butanediamine	C10H26N4	4	4	4
sprmd	Spermidine	N-(3-Aminopropyl)-1,4-butane-diamine	C7H19N3	3	3	3
sql	Squalene	Spinacene/Supraene	C30H50	0	0	0
srlh	S-Ribosyl-L-homocysteine	S-D-Ribosyl-L-homocysteine/Ribose-5-S-homocysteine/S-Ribosylhomocysteine/S-(5-Deoxy-D-ribos-5-yl)-L-homocysteine	C9H17NO6S	0	0	0
ssaltpp	Succinate semialdehyde-thiamin diphosphate anion		C16H21N4O10P2S	-2	-2	-2
sslcys	S-Sulfo-L-cysteine		C3H7NO5S2	-1	-1	-1
strcg	Styrene cis-glycol		C8H10O2	0	0	0
styrene	Styrene	Phenylethylene	C8H8	0	0	0
succ	Succinate	Succinic acid/Butanedionic acid/Ethylenesuccinic acid	C4H6O4	-2	-2	-2
succoa	Succinyl-CoA	Succinyl coenzyme A	C25H40N7O19P3S	-4	-5	-5
sucsal	Succinic semialdehyde	Succinate semialdehyde	C4H6O3	-1	-1	-1
sulald	Sulfoacetaldehyde	2-Sulfoacetaldehyde	C2H4O4S	-1	-1	-1
sulcatech	3-Sulfocatechol	2,3-Dihydroxybenzenesulfonate	C6H6O5S	-1	-1	-1
t3	D-Glyceraldehyde		C3H6O3	0	0	0
t3chc	trans-3-Chloroacrylic acid		C3H3ClO2	-1	-1	-1
t3chp	trans-3-Chloro-2-propene-1-ol		C3H5ClO	0	0	0
t6p	D-Tagatose 6-phosphate		C6H13O9P	-1	-2	-2
tartr	L-Tartaric acid	(R,R)-Tartaric acid/(R,R)-Tartrate/Tartaric acid/Tartrate/2,3-Dihydroxybutanedioic acid/(2R,3R)-Tartaric acid/(+)-Tartaric acid	C4H6O6	-2	-2	-2
taur	Taurine	2-Aminoethanesulfonic acid/Aminoethylsulfonic acid	C2H7NO3S	0	0	0
tchccm	2,3,5-Trichloro-cis,cis-muconate		C6H3Cl3O4	-2	-2	-2
tchrocat	3,4,6-Trichlorocatechol		C6H3Cl3O2	0	-1	-1
tcmbo	trans-4-Carboxymethylenebut-2-en-4-olide		C6H4O4	-1	-1	-1
tcynt	Thiocyanate	Thiocyanic acid	CHNS	-1	-1	-1
tcys	Thiocysteine		C3H7NO2S2	0	0	0
tdhdp	2,3,4,5-Tetrahydrodipicolinate	delta1-Piperidine-2,6-dicarboxylate/L-2,3,4,5-Tetrahydrodipicolinate/(S)-2,3,4,5-Tetrahydropyridine-2,6-dicarboxylate	C7H9NO4	-2	-2	-2
tethbp	Tetrahydrobiopterin	5,6,7,8-Tetrahydrobiopterin/2-Amino-6-(1,2-dihydroxypropyl)-5,6,7,8-tetrahydoro-4(1H)-pteridinone	C9H15N5O3	0	0	0
tgercoa	trans-Geranyl-CoA		C31H50N7O17P3S	-3	-4	-4
tgl	Triacylglycerol	Triglyceride	C6H5O6R3	0	0	0
tglu	Tetrahydropteroyltri-L-glutamate		C29H37N9O12	-4	-4	-4
thbn	Benzene-1,2,4-triol	Hydroxyhydroquinone/1,2,4-Benzenetriol/1,2,4-Trihydroxybenzene/Hydroxyquinol	C6H6O3	0	0	0
thf	5,6,7,8-Tetrahydrofolate	Tetrahydrofolate/Tetrahydrofolic acid/THF/(6S)-Tetrahydrofolate/(6S)-Tetrahydrofolic acid/(6S)-THFA	C19H23N7O6	-2	-2	-2
thfglu	THF-L-glutamate	Tetrahydrofolyl-[Glu](2)	C24H30N8O9	-3	-3	-3
thiamin	Thiamin	Thiamine/Vitamin B1/Aneurin/Antiberiberi factor	C12H17N4OS	1	1	1
thmp	Thiamin monophosphate	Thiamine monophosphate/Thiamin phosphate/Thiamine phosphate/TMP	C12H18N4O4PS	0	-1	-1
thmpp	Thiamine diphosphate	Thiamine diphosphate/Thiamin pyrophosphate/TPP/ThPP	C12H19N4O7P2S	-1	-1	-2
thr	L-Threonine	2-Amino-3-hydroxybutyric acid	C4H9NO3	0	0	0
thrtrna	L-Threonyl-tRNA(Thr)		C14H24NO12PR2(C5H8O6PR)n	0	0	0
thym	Thymine	5-Methyluracil	C5H6N2O2	0	0	0
thymd	Thymidine	Deoxythymidine	C10H14N2O5	0	0	0
thzp	4-Methyl-5-(2-phosphoethyl)-thiazole	4-Methyl-5-(2-phosphono-oxyethyl)-thiazole	C6H10NO4PS	-1	-2	-2
tnittol	Trinitrotoluene	2,4,6-Trinitrotoluene	C7H5N3O6	0	0	0
tol4sul	Toluene-4-sulfonate	Tosylate	C7H8O3S	-1	-1	-1
tolen	Toluene	Methylbenzene/Toluol	C7H8	0	0	0
tre	Trehalose	alpha,alpha-Trehalose/alpha,alpha'-Trehalose	C12H22O11	0	0	0
tre6p	alpha,alpha'-Trehalose 6-phosphate	Trehalose 6-phosphate	C12H23O14P	-1	-2	-2
trnaala	tRNA(Ala)		C10H17O10PR2(C5H8O6PR)n	0	0	0
trnaarg	tRNA(Arg)		C15H21N5O10PR(C5H8O6PR)n	0	0	0
trnaasp	tRNA(Asp)		C10H17O10PR2(C5H8O6PR)n	0	0	0
trnacys	tRNA(Cys)		C15H21N5O10PR(C5H8O6PR)n	0	0	0

trnagln	tRNA(Gln)		C15H21N5O10PR(C5H8O6PR)n	0	0	0
trnaglu	tRNA(Glu)		C15H21N5O10PR(C5H8O6PR)n	0	0	0
trnagly	tRNA(Gly)		C10H17O10PR2(C5H8O6PR)n	0	0	0
trnahis	tRNA(His)		C10H17O10PR2(C5H8O6PR)n	0	0	0
trnaile	tRNA(Ile)		C15H21N5O10PR(C5H8O6PR)n	0	0	0
trnaleu	tRNA(Leu)		C15H21N5O10PR(C5H8O6PR)n	0	0	0
trnalys	tRNA(Lys)		C10H17O10PR2(C5H8O6PR)n	0	0	0
trnamet	tRNA(Met)		C15H21N5O10PR(C5H8O6PR)n	0	0	0
trnaphe	tRNA(Phe)		C10H17O10PR2(C5H8O6PR)n	0	0	0
trnapro	tRNA(Pro)		C10H17O10PR2(C5H8O6PR)n	0	0	0
trnaser	tRNA(Ser)		C10H17O10PR2(C5H8O6PR)n	0	0	0
trnathr	tRNA(Thr)		C10H17O10PR2(C5H8O6PR)n	0	0	0
trnatrp	tRNA(Trp)		C15H21N5O10PR(C5H8O6PR)n	0	0	0
trnatyr	tRNA(Tyr)		C15H21N5O10PR(C5H8O6PR)n	0	0	0
trnaval	tRNA(Val)		C15H21N5O10PR(C5H8O6PR)n	0	0	0
trp	L-Tryptophan	Tryptophan/(S)-alpha-Amino-beta-(3-indolyl)-propionic acid	C11H12N2O2	0	0	0
trptrna	L-Tryptophanyl-tRNA(Trp)		C26H31N7O11PR(C5H8O6PR)n	1	1	1
tsul	Thiosulfate	Hyposulfite	HS2O3	-1	-1	-1
tym	Tyramine	2-(p-Hydroxyphenyl)ethylamine	C8H11NO	1	1	1
tyr	L-Tyrosine	(S)-3-(p-Hydroxyphenyl)alanine/(S)-2-Amino-3-(p-hydroxyphenyl)propionic acid	C9H11NO3	0	0	0
tyrpm	Tryptamine	3-(2-Aminoethyl)indole	C10H12N2	1	1	1
tyrtrna	L-Tyrosyl-tRNA(Tyr)		C24H30N6O12PR(C5H8O6PR)n	1	1	1
u1car	Urea-1-carboxylate	Allophanate/Allophanic acid	C2H4N2O3	-1	-1	-1
u3hga	UDP-3-O-(3-hydroxytetradecanoyl)-D-glucosamine	UDP-3-O-(beta-hydroxymyristoyl)-D-glucosamine	C29H51N3O18P2	-1	-1	-2
uaagmda	Undecaprenyl-diphospho-N-acetylmuramoyl-(N-acetylglucosamine)-L-alanyl-D-glutamyl-meso-2,6-diaminopimeloyl-D-alanyl-D-alanine		C95H156N8O28P2	-4	-4	-5
uaagmm5da	Undecaprenyl-diphospho-N-acetylmuramoyl-(N-acetylglucosamine)-L-alanyl-D-glutaminyl-meso-2,6-diaminopimeloyl-(glycyl)5-D-alanyl-D-alanine		C105H172N14O32P2	-3	-3	-4
uaagmmda	Undecaprenyl-diphospho-N-acetylmuramoyl-(N-acetylglucosamine)-L-alanyl-D-glutaminyl-meso-2,6-diaminopimeloyl-D-alanyl-D-alanine		C95H157N9O27P2	-3	-3	-4
uaccg	UDP-N-acetyl-3-(1-carboxyvinyl)-D-glucosamine	UDP-N-acetyl-3-O-(1-carboxyvinyl)-D-glucosamine/UDP-N-acetylglucosamine-3-O-pyruvateether/UDP-N-acetylglucosamine enolpyruvate	C20H29N3O19P2	-3	-3	-3
uagmda	Undecaprenyl-diphospho-N-acetylmuramoyl-L-alanyl-D-glutamyl-meso-2,6-diaminopimeloyl-D-alanyl-D-alanine		C87H143N7O23P2	-4	-4	-5
uama	UDP-N-acetylmuramoyl-L-alanine		C23H36N4O20P2	-3	-3	-3
uamag	UDP-N-acetylmuramoyl-L-alanyl-D-glutamate		C28H43N5O23P2	-4	-4	-4
udcpdp	Undecaprenyl diphosphate		C55H92O7P2	-2	-2	-3
udcpp	Undecaprenyl phosphate		C55H91O4P	-1	-2	-2
udp	UDP	Uridine 5'-diphosphate	C9H14N2O12P2	-2	-2	-3
udpacgal	UDP-N-acetyl-D-galactosamine		C17H27N3O17P2	-2	-2	-2
udpg	UDP-glucose	UDPglucose/UDP-D-glucose/Uridine diphosphate glucose/UDP-alpha-D-glucose	C15H24N2O17P2	-2	-2	-2
udpg23a	UDP-2,3-bis(3-hydroxytetradecanoyl)glucosamine	UDP-2,3-bis(beta-hydroxymyristoyl)-D-glucosamine/UDP-2,3-bis(3-hydroxytetradecanoyl)-D-glucosamine	C43H77N3O20P2	-2	-2	-2
udpg2aa	UDP-3-O-(3-hydroxytetradecanoyl)-N-acetylglucosamine	UDP-3-O-(beta-hydroxymyristoyl)-N-acetylglucosamine	C31H53N3O19P2	-2	-2	-2

udpgal	UDP-D-galactose	UDP-galactose/UDP-D-galactopyranose	C15H24N2O17P2	-2	-2	-2
udpglcur	UDP-D-glucuronate	UDP-glucuronate/UDPglucuronate/UDP-alpha-D-glucuronate	C15H22N2O18P2	-3	-3	-3
udpnag	UDP-N-acetyl-D-glucosamine	UDP-N-acetylglucosamine	C17H27N3O17P2	-2	-2	-2
udpnam	UDP-N-acetylmuramate	UDP-N-acetylmuramic acid/UDP-MurNAc	C20H31N3O19P2	-3	-3	-3
ugmd	UDP-N-acetylmuramoyl-L-alanyl-D-gamma-glutamyl-meso-2,6-diaminopimelate	UDP-N-acetylmuramoyl-L-alanyl-D-gamma-glutamyl-meso-2,6-diamino-heptanedioate	C35H55N7O26P2	-4	-4	-5
ugmda	UDP-N-acetylmuramoyl-L-alanyl-D-glutamyl-6-carboxy-L-lysyl-D-alanyl-D-alanine	UDP-N-acetylmuramoyl-L-alanyl-D-glutamyl-meso-2,6-diaminopimeloyl-D-alanyl-D-alanine	C41H65N9O28P2	-4	-4	-5
ump	UMP	Uridylic acid/Uridine monophosphate/Uridine 5'-monophosphate/5'Uridylic acid	C9H13N2O9P	-1	-2	-2
up4g	P1,P4-Bis(5'-uridyl) tetraphosphate	UppppU	C18H26N4O23P4	-4	-4	-4
uppg1	Uroporphyrinogen I		C40H44N4O16	-8	-8	-8
uppg3	Uroporphyrinogen Ⅲ		C40H44N4O16	-8	-8	-8
uq	Ubiquinone	Coenzyme Q/CoQ/Q	C14H18O4(C5H8)n	0	0	0
uqh2	Ubiquinol	QH2/CoQH2	C14H20O4(C5H8)n	0	0	0
ura	Uracil		C4H4N2O2	0	0	0
urate	Urate	Uric acid	C5H4N4O3	0	0	-1
urdglyc	(-)-Ureidoglycolate	(S)-Ureidoglycolate	C3H6N2O4	-1	-1	-1
urea	Urea	Carbamide	CH4N2O	0	0	0
uri	Uridine		C9H12N2O6	0	0	0
urocan	Urocanate	Urocanic acid	C6H6N2O2	0	-1	-1
utp	UTP	Uridine 5'-triphosphate/Uridine triphosphate	C9H15N2O15P3	-3	-3	-4
val	L-Valine	2-Amino-3-methylbutyric acid	C5H11NO2	0	0	0
valtrna	L-Valyl-tRNA(Val)		C20H30N6O11PR(C5H8O6PR)n	1	1	1
xan	Xanthine		C5H4N4O2	0	0	0
xmp	Xanthosine 5'-phosphate	Xanthylic acid/XMP/(9-D-Ribosylxanthine)-5'-phosphate	C10H13N4O9P	-1	-2	-2
xp4g	P1,P4-Bis(5'-xanthosyl) tetraphosphate	XppppX	C20H26N8O23P4	-4	-6	-6
xtp	XTP		C10H15N4O15P3	-3	-3	-4
xtsine	Xanthosine		C10H12N4O6	0	0	0
xu5p	D-Xylulose 5-phosphate		C5H11O8P	-1	-2	-2
xyl	D-Xylose	Wood sugar	C5H10O5	0	0	0
xylt	Xylitol		C5H12O5	0	0	0
z4hphea	(Z)-4-Hydroxyphenylacetaldehyde-oxime		C8H9NO2	0	0	0
zoe	(Z)-5-Oxohex-2-enedioate	gamma-Oxalocrotonate/4-Oxalocrotonate	C6H6O5	-2	-2	-2
