# Supplementary material for: Genome-scale reconstruction and in silico analysis of the Ralstonia eutropha H16 for polyhydroxyalkanoate synthesis, lithoautotrophic growth, and 2-methyl citric acid production
Source: BMC Syst Biol. 2011 Jun 28;5:101. doi: 10.1186/1752-0509-5-101 (PMC3154180; doi:10.1186/1752-0509-5-101)
Supplement: Additional file 3 — Biomass composition of Ralstonia eutropha H16 [file 1752-0509-5-101-S3.PDF]

Additional file 3. Biomass composition of *Ralstonia eutropha H16*.

Table 3-1. Macromolecular composition[a]

Macromolecular composition

Component	Composition (g/g DCW)	Comments
**Protein**	0.680	Taken from Srinivasan et al. (2002)
**DNA**	0.031	Taken from Neidhardt et al. (1996)
**RNA**	0.060	Taken from Brown (2002)
**Phospholipid**	0.050	Taken from Gmeiner et al. (1980)
**Cofactors and vitamins**	0.030	Assumption (Small molecules compose less than 3% of cell dry weight) (Ingraham et al., 1983)
**Cell wall**	0.150	
Lipopolysaccharide	0.034	Taken from Neidhardt et al. (1996)
Carbohydrate	0.055	Determined in this study
Peptidoglycan	0.060	Carbohydrates made about 5.5% of the cell wall in this study. The rest was assumed to be peptidoglycan.
**Ash**	0.000	Assumption (not included in this model)

[a]Calculated for an average macromolecular composition of *R.eutropha* H16 in MR minimal medium with D-fructose. Biomass composition was experimentally measured at the exponential growth phase of aerobic batch cultivation (specific growth rate: 0.2 h^{-1}), which is the average of three samples. MW of 1 water was subtracted from MW of each molecule to account for esterification or peptide bonding.

Table 3-2. Protein composition

Protein composition

The amino acid composition was determined by a Waters HPLC systems (Water Corporation, Milford, MA). Absorbance at 354 nm was measured.

Amino acids	mmol/g protein
Alanine	1.211
Arginine	0.456
Asparagine	0.369
Aspartate	0.369
Cysteine	0.115
Glutamate	0.512
Glutamine	0.512
Glycine	1.135
Histidine	0.223
Isoleucine	0.306
Leucine	0.522
lysine	0.189
Methionine	0.159
Phenylalanine	0.430
Proline	0.997
Serine	0.421
Threonine	0.764
Tryptophane	0.008
Tyrosine	0.222
Valine	0.687

Table 3-3. DNA composition

DNA analysis

The DNA composition was determined from the genomic sequence of *R. eutropha*. GC content of *R. eutropha* is 65.2%.

Nucleotide	mol/mol, DNA	MW, g/mol	mmol/g DNA
dAMP	0.174	313.200	0.564
dCMP	0.326	289.200	1.054
dTMP	0.174	304.200	0.564
dGMP	0.326	329.200	1.054

Table 3-4. RNA composition

RNA composition

It was assumed that mRNA makes up 5% and rRNA 80% of the total RNA. The rest was assumed to be tRNA (Brown TA (2002) Genomes 2nd, Wiley-Liss, New York).

Nucleotide	mol/mol RNA			MW, g/mol	mol/mol RNA	mmol/g RNA
	mRNA	rRNA	tRNA			
	0.050	0.800	0.150			
AMP	0.174	0.202	0.207	329.200	0.202	0.631
GMP	0.326	0.225	0.274	345.200	0.240	0.750
CMP	0.326	0.316	0.328	305.200	0.319	0.998
UMP	0.174	0.256	0.191	306.200	0.239	0.747

Table 3-5. Phospholipids composition

Phospholipids composition

The composition of phospholipids was taken from Galbraith el al. (1999) (Galbraith L, Jonsson MH, Rudhe LC, Wilkinson SG (1999) Lipids and fatty acids of*Burkholderia* and *Ralstonia* species. FEMS Microbiology Letters 173:359-364).

Component	g/g phospholipids	mmol/g phospholipids
Phosphatidylethanolamine	0.660	0.927
Phosphatidylglycerol	0.210	0.283
Diphosphatidylglycerol (Cardiolipin)	0.130	0.093

Table 5.1 Molecular weights of phospholipids components:

Constituent	MW, g/mol		
	backbone	# of fatty acids residues	total
Phosphatidylethanolamine	181.128	2	711.74
Phosphatidylglycerol	212.139	2	742.75
Cardiolipin	332.183	4	1393.40

Table 3-6. Composition of fatty acids in phospholipids

Composition of fatty acids in phospholipids

Kalacheva GS and Volova TG (2007) Fatty acid composition of *Wautersia eutropha* lipids under conditions of active polyhydroxyalkanoates synthesis. Mikrobiologiia 76:608-14

Complete medium

Fatty acid	g/g total fatty acids	MW, g/mol	mmol/g total fatty acids	mol/mol total fatty acids
c12	0.006	214.947	0.027	0.007
c14:1	0.000	240.000	0.001	0.000
c14	0.399	252.477	1.581	0.419
c15:1	0.000	254.000	0.000	0.000
c15	0.006	256.000	0.025	0.007
c16:1	0.177	268.000	0.660	0.175
c16	0.258	270.258	0.956	0.254
c17:1	0.007	282.000	0.026	0.007
c17	0.006	282.669	0.021	0.006
c18:1	0.125	296.000	0.422	0.112
c18	0.015	298.000	0.050	0.013
c19	0.000	310.000	0.000	0.000

Nitrogen-free medium

Fatty acid	g/g total fatty acids	MW, g/mol	mmol/g total fatty acids	mol/mol total fatty acids
c12	0.002	214.947	0.007	0.002
c14:1	0.001	240.000	0.004	0.001
c14	0.391	252.477	1.550	0.414
c15:1	0.010	254.000	0.038	0.010
c15	0.027	256.000	0.104	0.028
c16:1	0.059	268.000	0.220	0.060
c16	0.259	270.258	0.957	0.255
c17:1	0.000	282.000	0.000	0.000
c17	0.112	282.669	0.395	0.105
c18:1	0.117	296.000	0.395	0.105
c18	0.009	298.000	0.032	0.008
c19	0.014	310.000	0.046	0.012

Table 3-7. Cofactors and vitamins incorporated in the biomass

Cofactors and vitamins incorporated in the biomass

Cofactors and vitamins are assumed to be same ratio (w/w)

Molecule	MW, g/mol	g/g cofactors and vitamins	mmol/g cofactors and small molecules
Pyridoxine	169.178	0.111	0.656
Coenzyme A	767.535	0.111	0.145
Flavin adenine dinucleotide	785.550	0.111	0.141
Flavin mononucleotide	456.344	0.111	0.243
Ubiquinone	794.623	0.111	0.140
NAD	664.433	0.111	0.167
NADP	744.413	0.111	0.149
Tetrahydrofolate	445.430	0.111	0.249
Thiamin	265.356	0.111	0.418

Table 3-8. Carbohydrate composition

Carbohydrate composition

The carbohydrates composition was determined by CarboPac PA1(4.5 x 250 mm) and CarboPac PA1 cartridge (4.5 x 50 mm) with Bio-LC DX-600 (Dionex, Sunnyvale, CA).

Component	Molar ratio	MW, g/mol	mmol/g carbohydrate
N-acetylglucosamine	4.000	203.194	3.937
N-	1.000	203.194	0.984

Table 3-9. Lipopolysaccharide composition

Lipopolysaccharide composition

The composition of lipopolysaccharide was assumed to be the same as in *Escherichia coli* (Neidhardt FC, Curtiss R, Ingraham JL, Lin ECC, Low KB, Magasanik B, Reznikoff WS, Riley M, Schaechter M, Umbarger HE (1996) Escherichia coli and Salmonella, ASM press, Washington D.C.).

Component	Molar ratio	MW, g/mol	mmol/g LPS
KDO(2)-lipid A	1.000	1624.910	0.140
ADP-L-glycero-D-manno-heptose	3.000	619.370	0.420
UDPglucose	2.000	566.050	0.280
CDP-Ethanolamine	2.000	446.06	0.280
CMP-2-keto-3-deoxyoctanoate	3.000	543.109	0.420

Reference

Brown TA: *Genomes 2nd*. New York: Wiley-Liss; 2002.
Galbraith L, Jonsson MH, Rudhe LC, Wilkinson SG: **Lipids and fatty acids of *Burkholderia* and *Ralstonia* species**. *FEMS Microbiol Lett* 1999, **173:**359-364.
Gmeiner J, Schlecht S: **Molecular composition of the outer membrane of *Escherichia coli* and the importance of protein-lipopolysaccharide interactions**. *Arch Microbiol* 1980, **127:**81-86.
Ingraham JL, Maalee O, Neidhardt FC: *Growth of the Bacterial Cell*. Sunderland: Sinauer Associates; 1983.
Kalacheva GS and Volova TG: **Fatty acid composition of *Wautersia eutropha* lipids under conditions of active polyhydroxyalkanoates synthesis** *Mikrobiologiia* 2007, **76:**608-614.
Neidhardt FC, Curtiss R, Ingraham JL, Lin ECC, Low KB, Magasanik B, Reznikoff WS, Riley M, Schaechter M, Umbarger HE: *Escherichia coli and Salmonella*, Washington D.C.: ASM press; 1996.
Srinivasan S, Barnard GC, Gerngross TU: **A novel high-cell-density protein expression system based on *Ralstonia eutropha*.** *Appl Environ Microbiol* 2002, **68:**5925-5932.
