# Supplementary material for: Genome-scale reconstruction and in silico analysis of the Ralstonia eutropha H16 for polyhydroxyalkanoate synthesis, lithoautotrophic growth, and 2-methyl citric acid production
Source: BMC Syst Biol. 2011 Jun 28;5:101. doi: 10.1186/1752-0509-5-101 (PMC3154180; doi:10.1186/1752-0509-5-101)
Supplement: Additional file 4 — Batch and chemostat culture profile of Ralstonia eutropha H16 in minimal medium [file 1752-0509-5-101-S4.PDF]

Additional file 4. Batch and chemostat culture profile of *Ralstonia eutropha* H16 in minimal medium.

Table 4-1. The components of complete and nitrogen-limited minimal medium.

/100 mL	Complete	Nitrogen-limited	
$(NH_4)_2HPO_4$	4 g·L^{-1}	0 g·L^{-1}	
Na_2HPO_4	0 g·L^{-1}	4 g·L^{-1}	
NH_4Cl	0 g·L^{-1}	1.8 g·L^{-1}	
KH_2PO_4	6.67 g·L^{-1}	6.67 g·L^{-1}	
Citric acid	0.8 g·L^{-1}	0.8 g·L^{-1}	
$MgSO_4·7H_2O$	0.8 g·L^{-1}	0.8 g·L^{-1}	
Trace metal solution	5 mL	5 mL	Lee and Lee, 1996

Table 4-2. The time profile of aerobic culture of *R. eutropha* for complete and nitrogen-limited minimal medium.

Complete minimal medium			Nitrogen-limited medium		
Time (h)	biomass (g·L^{-1})	D-fructose (g·L^{-1})	Time (h)	biomass (g·L^{-1})	D-fructose (g·L^{-1})
0	0.279104	21.3435	0	0.17024	19.97
2	0.52416	20.9596	9	0.34496	20.51
4	0.89376	20.9715	16	0.44128	20.34
7	2.10112	18.7423	23.25	0.5376	20.36
10	4.4688	14.481	33	0.92512	19.51
17.5	15.0528	0.117	40	2.28928	16.22
21.5	14.784	0	43	4.3008	11.61
			48.4	6.944	7.36
			56.5	10.752	2.75
			60.7	13.0368	0.00

Table 4-3. The specific growth rate and D-fructose uptake rate for complete and nitrogen-limited minimal medium..

	Complete	Nitrogen-limited
Growth rate (h^{-1})	0.25±0.08	0.15±0.03
D-Fructose (mmol·gDCW^{-1}·h^{-1})	2.6±0.9	1.7±0.5
O_2 (mmol·gDCW^{-1}·h^{-1})	4.6±0.3	3.46±0.3

Table 4-5. The time profile of aerobic chemostat culture of *R. eutropha.*

Dilution (h^{-1})	D-fructose uptake (mmol·gDCW^{-1}·h^{-1})
0.05	0.45
0.07	0.75
0.10	1.21

Figure 4-1. The time profile of aerobic culture of *R. eutropha.*

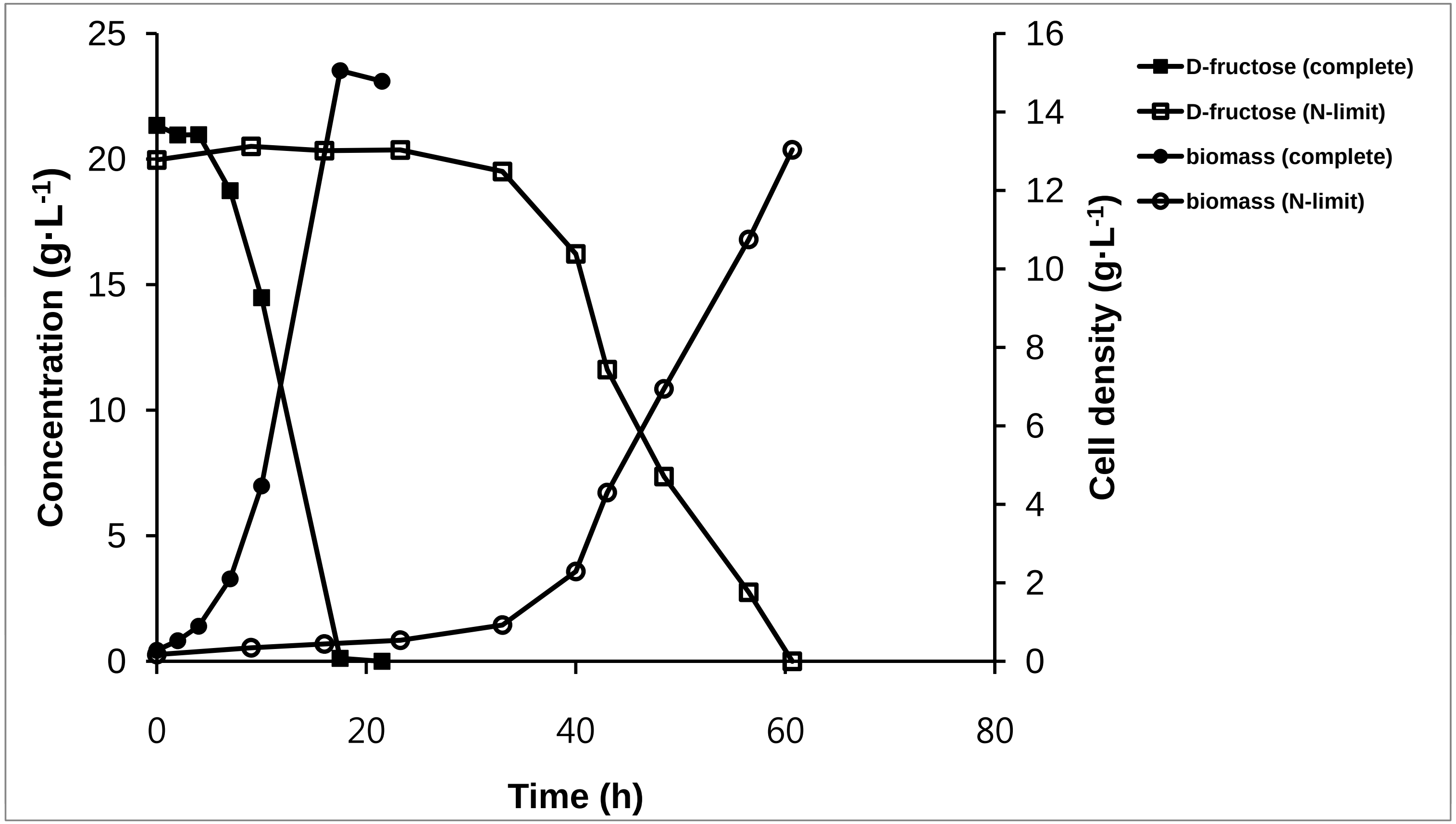

Reference

Lee Y, Lee SY: **Enhanced production of poly(3-hydroxybutyrate) by filamentation-suppressed recombinant *Escherichia coli* in a defined medium.** *J Environ Polymer Degrad* 1996, 4:131-134.

Srinivasan S, Barnard GC, Gerngross TU: **A novel high-cell-density protein expression system based on Ralstonia eutropha.** *Appl Environ Microbiol* 2002, **68:**5925-5932
