# Supplementary material for: Genome-scale reconstruction and in silico analysis of the Ralstonia eutropha H16 for polyhydroxyalkanoate synthesis, lithoautotrophic growth, and 2-methyl citric acid production
Source: BMC Syst Biol. 2011 Jun 28;5:101. doi: 10.1186/1752-0509-5-101 (PMC3154180; doi:10.1186/1752-0509-5-101)

Additional file 5. HPLC and GC analysis results in culture of *Ralstonia eutropha* H16 in minimal medium.

Figure 5-1. The HPLC results for the detection of D-fructose and organic acid concentration in the cuture of *R. eutropha* in minimal medium.

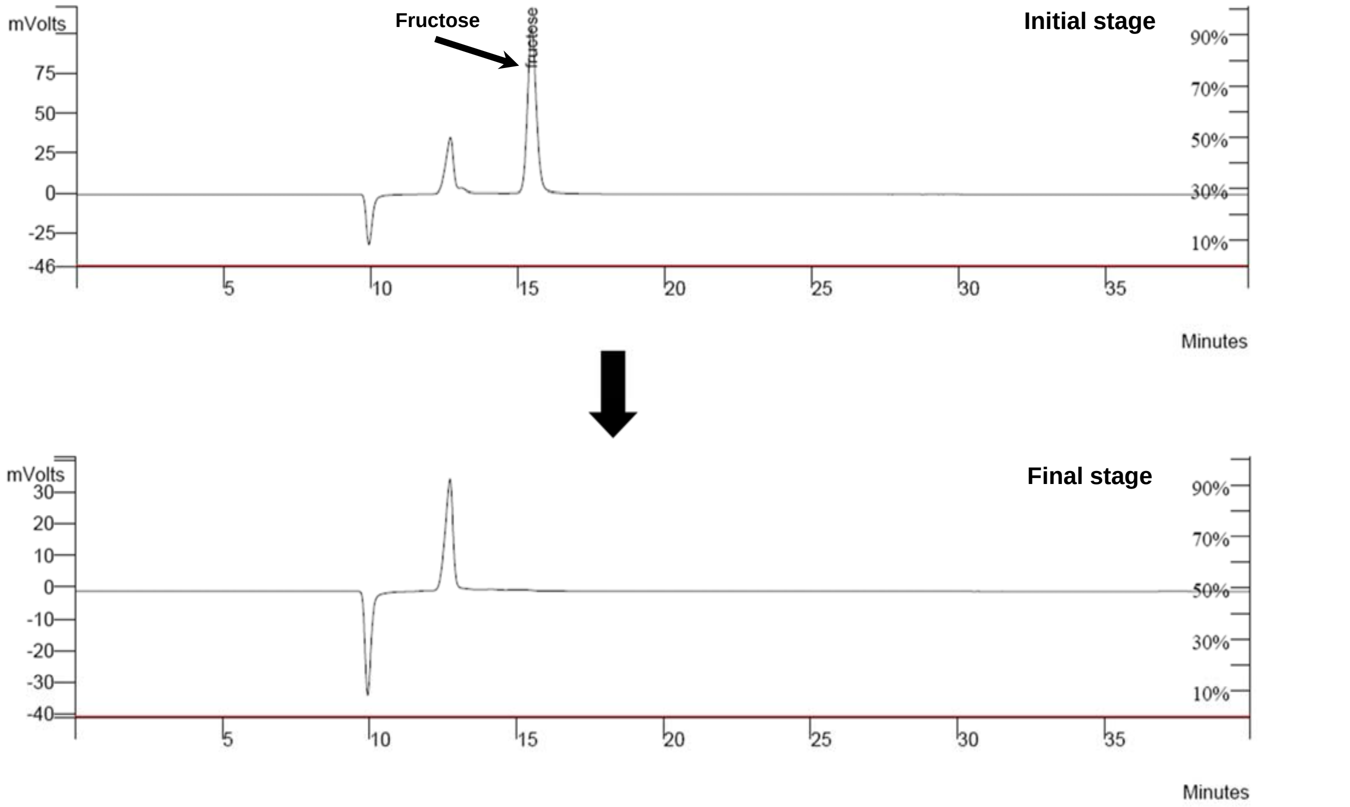

Figure 5-2. The GC results for the detection of PHB monomer in the cuture of *R. eutropha* in minimal medium.

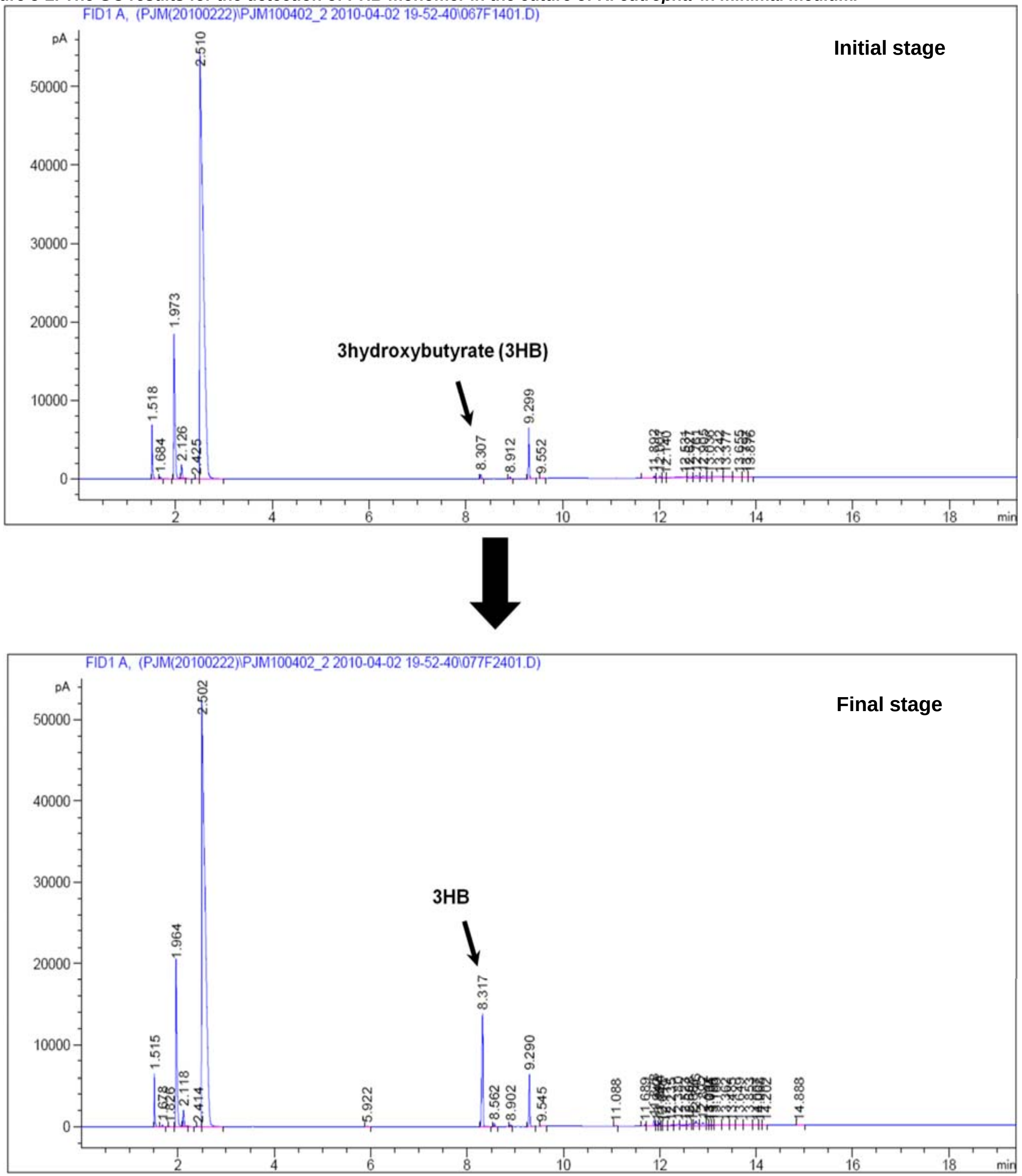

Supplement: Additional file 5 — HPLC and GC analysis results in culture of Ralstonia eutropha H16 in minimal medium [file 1752-0509-5-101-S5.PDF]
