# Supplementary material for: Genome-scale reconstruction and in silico analysis of the Ralstonia eutropha H16 for polyhydroxyalkanoate synthesis, lithoautotrophic growth, and 2-methyl citric acid production
Source: BMC Syst Biol. 2011 Jun 28;5:101. doi: 10.1186/1752-0509-5-101 (PMC3154180; doi:10.1186/1752-0509-5-101)
Supplement: Additional file 6 — Carbon source utilization of Ralstonia eutropha H16 [file 1752-0509-5-101-S6.PDF]

Additional file 6. Carbon source utilization of *R. eutorpha*.

	Experiment	Simulation
Malate	O	O
2,3-butanediol	X	X
2-aminoethanol	X	X
2-oxoglutarate	X	X
2-phospho-D-glycerate	O	O
3-hydroxy-benzoate	O	O
3-hydroxybutyrate	O	O
3-phospho-D-glycerate	O	O
4-Cresol	O	O
4-Fluorobenzoate	O	O
4-hydroxy benzoate	O	O
5-keto-gluconate	X	X
6-Chlorohydroxyquinol	O	O
Acetate	O	O
Adipate	O	O
adonitol	X	X
Trehalose	X	X
alpha-cyclodextrin	X	X
alpha-D-glucose	X	X
alpha-d-lactose	X	X
2-Hydroxybutyric acid	O	O
2-Ketobutyric acid	O	O
Anthranilate	O	O
Benzoate	O	O
Benzoylformate	O	O
Bezaldehyde	O	O
3-hydroxybutyric acid	O	O
Biphenyl-2,3-diol	O	O
b-methyl-d-glucoside	X	X
Butyric acid	O	O
catechol	O	O
cellobiose	X	X
cis, cis-muconate	O	O
cis-Aconitic acid	O	O
Citrate	O	O
D-alanine	O	O
d-arabitol	X	X
Decanoate	X	X
dextrin	X	X
D-Fructose	O	O
D-galactonate	X	X
D-galactonic acid lactone	X	X
D-galactose	X	X
D-galacturonic acid	X	X
D-gluconic acid	O	O
d-glucosaminic acid	X	X
D-glucuronic acid	X	X
dl-alpha-glycerol phosphate	X	X
dl-carnitine	X	X
dl-lactic acid	O	O
D-mannitol	X	X
D-mannose	X	X
d-melibiose	X	X
d-raffinose	X	X
D-Saccharic acid	X	X
D-serine	X	X
d-sorbitol	X	X
ethanol	X	X
Formic acid	O	O
Fumarate	O	O
gamma-aminobutyric acid	X	X
4-Hydroxybutyric acid	O	O
gentiobiose	X	X
Gentisate	O	O
glucose-1-phosphate	X	X
glucose-6-phosphate	X	X
Glycerol	O	O
Glycogen	X	X
Glycolate	O	O
glycyl-l-aspartic acid	X	X
glyoxylate	O	O
hydroxy-l-proline	X	X
i-erythritol	X	X
inosine	X	X
isocitrate	O	O
Isomaltose	X	X
Itaconic acid	X	X
Kynurenate	O	O
Kynurenine	O	O
lactate	O	O
Lactose	X	X
lactulose	X	X
L-alanine	O	O

L-arabinose	X	X
l-asparagine	O	O
l-aspartic acid	O	O
L-fucose	X	X
l-glutamic acid	O	O
L-histidine	O	O
L-leucine	O	X
L-Mandelate	X	X
l-ornithine	X	X
L-phenylalanine	O	O
l-proline	O	O
Rhamnose	X	X
L-serine	O	O
L-Threonine	O	O
L-tryptophan	O	O
Malonic acid	X	X
Maltose	X	X
3-Cresol	X	X
Melibiose	X	X
Inositol	X	X
Muconolactone	O	O
myristate	O	O
N-Acetyl-d-galactosamine	X	X
N-Acetyl-d-glucosamine	X	O
Nicotinate	O	O
o-cresol	X	X
Oleic acid	O	O
palmitate	O	O
Phenol	O	O
Phenylacetate	O	O
Phenylethylamine	X	X
4-Hydroxyphenylacetic acid	O	O
propionate	O	O
Protocatechuate	O	O
putrescine	X	X
pyruvate	O	O
Quinate	X	X
Ribose	X	X
Salicin	X	X
Shikimate	X	X
Succinate	O	O
Sucrose	X	X
thymidine	X	X
turanose	X	X
uridine	X	X
Urocanic acid	O	O
xylitol	X	X
Xylose	X	X

Reference

1. Yabuuchi E, Kawamuta Y, Ezaki T: *Ralstonia Yabuuchi, Kosako, Yano, Hotta and Nishiuchi 1996, 625 VP (Effective publication: Yabuuchi, Kosako, Yano, Hotta and Nishiuchi 1995, 902).* US: Springer; 1996.

2. Moissenet D, Goujon CP, Garbarg-chenon A, Vu-thien H: **CDC Group IV c-2: a New *Ralstonia* Species Close to *Ralstonia eutropha*.** *J Clin Microbiol* 1999,

3. Johnson BF, Stanier RY: **Dissimilation of Aromatic Compounds by *Alcaligenes eutrophus*.** *J Bacteriol* 1971, **107:**468-475.

4. Dennis D, McCoy M, Stangl A, Valentin HE, Wu Z: **Formation of poly(3-hydroxybutyrate-co-3-hydroxyhexanoate) by PHA synthase from *Ralstonia eutropha*.** *J Bacteriol 1998,* **64:**177-186.

5. Wang Z, Bramer C, Steinbuchel A: **Two phenotypically compensating isocitrate dehydrogenases in *Ralstonia eutropha*.** *FEMS Microbiol Lett* 2003, **227:**9-16.

6. Raberg M, Reinecke F, Reichelt R, Malkus U, König S, Pötter M, Fricke WF, Pohlmann A, Voigt B, Hecker M, Friedrich B, Bowien B, Steinbüchel A: ***Ralstonia eutropha* H16 flagellation changes according to nutrient supply and state of poly(3-hydroxybutyrate) accumulation.** *Appl Environ Microbiol* 2008, **74:**4477-[illegible]

7. Zarnt G, Schräder T, Andreesen JR: **Degradation of tetrahydrofurfuryl alcohol by *Ralstonia eutropha* is initiated by an inducible pyrroloquinoline quinone-dependent alcohol dehydrogenase.** *Appl Environ Microbiol* 1997, **63:**4891-4898.

8. Lee JN, Shin HD, Lee YH: **Metabolic engineering of pentose phosphate pathway in *Ralstonia eutropha* for enhanced biosynthesis of poly-beta-hydroxybutyrate.** *Biotechnol Prog* 2003, **19:**1444-1449.

9. Ewering C, Heuser F, Benolken JK, Bramer CO, Steinbuchel A: **Metabolic engineering of strains of *Ralstonia eutropha* and *Pseudomonas* putida for biotechnological production of 2-methylcitric acid.** *Metabolic engineering* 2006, **8:**587-602.

10. Ampe F, Leonard D, Lindley ND: **Growth performance and pathway flux determine substrate preference of *Alcaligenes eutrophus* during growth on acetate plus aromatic compound mixtures.** *Appl Microbiol Biotechnol* 1996, **46:** 562-569.

11. Ampe F, Leonard D, Lendley ND: **Repression of Phenol Catabolism by Organic Acids in *Ralstonia eutropha*.** *Appl Environ Microbiol* 1998, **64:**1-6.

12. Kimura H, Ohura T, Takeishi M, Nakamura S, Doi Y: **Effective microbial production of poly(4-hydroxybutyrate) homopolymer by *Ralstonia eutropha* H16.** *Polym Int* 1999, **48:**1073-1079.

13. Yu ST, Lin CC, Too JR: **PHBV production by *Ralstonia eutropha* in a continuous stirred tank reactor.** *Process Biochem* 2005, **40:**2729-2734.

14. Yu J, Si Y: **Metabolic Carbon Fluxes and Biosynthesis of Polyhydroxyalkanoates in *Ralstonia eutropha* on Short Chain Fatty Acids.** *Biotechnol Prog* 2004,

15. Madison LL, Huisman GW: **Metabolic Engineering of Poly(3-Hydroxyalkanoates): From DNA to Plastic.** *Microbiol Mol Biol Reviews* 1999, **63:**21-53.

16. Pohlmann A, Fricke WF, Reinecke F, Kusian B, Liesegang H, Cramm R, Eitinger T, Ewering C, Potter M, Schwartz E, Strittmatter A, Voss I, Gottschalk G, Steinbuchel A, Friedrich B, Bowien B: **Genome sequence of the bioplastic-producing "*Knallgas*" bacterium *Ralstonia eutropha* H16.** *Nature biotech* 2006, **24:**1257-1262.
